# Cardamonin Inhibits the Nuclear Translocation and DNA Binding of RelA in the Tumor Necrosis Factor-α-Induced NF-κB Signaling Pathway in Human Lung Adenocarcinoma A549 Cells

**DOI:** 10.3390/molecules30224324

**Published:** 2025-11-07

**Authors:** Nhat Thi Vu, Quy Van Vu, Nghia Trong Vo, Riho Tanigaki, Hue Tu Quach, Yasunobu Miyake, Tomoo Shiba, Takao Kataoka

**Affiliations:** 1 Department of Applied Biology, Kyoto Institute of Technology, Matsugasaki, Sakyo-ku, Kyoto 606-8585, Japan; 2 Division of Molecular and Cellular Immunoscience, Department of Biomolecular Sciences, Faculty of Medicine, Saga University, Saga 849-8501, Japan; 3 Center for Social and Biomedical Engineering, Kyoto Institute of Technology, Matsugasaki, Sakyo-ku, Kyoto 606-8585, Japan

**Keywords:** cardamonin, 4′-hydroxychalcone, isoliquiritigenin, xanthohumol, chalcone, TNF-α, NF-κB, RelA, ICAM-1

## Abstract

Tumor necrosis factor α (TNF-α) activates the nuclear factor κB (NF-κB) signaling pathway, which promotes the expression of NF-κB-responsive genes, including intercellular adhesion molecule 1 (ICAM-1). We previously reported that cardamonin, a chalcone-type flavonoid, inhibited TNF-α-induced ICAM-1 expression in human lung adenocarcinoma A549 cells. However, the mechanisms by which cardamonin inhibits the TNF-α-induced NF-κB signaling pathway have yet to be elucidated. Therefore, we herein investigated the effects of cardamonin on TNF-α-induced gene expression and the NF-κB-dependent signaling pathway. Cardamonin reduced TNF-α-induced ICAM-1 mRNA expression and NF-κB reporter activity. It did not affect the inhibitor of NF-κB α (IκBα) degradation, but prevented RelA nuclear translocation and binding to the ICAM-1 promoter. Consistent with this result, three other chalcone derivatives (4′-hydroxychalcone, isoliquiritigenin, and xanthohumol) did not affect the degradation of IκBα, but inhibited nuclear RelA translocation. Cardamonin exhibited the same inhibitory profiles in human breast cancer MCF-7 cells and human fibrosarcoma HT-1080 cells. Cysteine 38 (C38) of RelA was not a primary target site of cardamonin because cardamonin inhibited the nuclear translocation of the RelA C38S mutant. An *in silico* molecular docking analysis confirmed that cardamonin was not positioned close enough to RelA C38 to mediate covalent binding, and also that cardamonin interacted with RelA at different sites. Mutations in these interaction sites abrogated the nuclear translocation of RelA in response to a TNF-α stimulation. The present results demonstrate that cardamonin inhibited the nuclear translocation of RelA and its DNA binding in the NF-κB signaling pathway in response to a TNF-α stimulation.

## 1. Introduction

Proinflammatory cytokines are mainly produced by macrophages in peripheral tissues during inflammation [1,2]. These cytokines are capable of stimulating nearby endothelial cells to up-regulate the expression of cell adhesion molecules, such as intercellular adhesion molecule 1 (ICAM-1), which then mediates the recruitment of circulating leukocytes and their transmigration to sites of inflammation [3,4]. Therefore, adhesion molecules play an essential role in the inflammatory responses induced by proinflammatory cytokines. Proinflammatory cytokines are also released and promote tumorigenesis in the tumor microenvironment [5,6,7]. These proinflammatory cytokines up-regulate the expression of many genes in various types of cancer and promote tumor initiation, epithelial–mesenchymal transition, angiogenesis, and metastasis [5,6,7]. In addition, adhesion molecules, such as ICAM-1, play a role in metastasis in various types of cancer [8,9,10].

Tumor necrosis factor (TNF)-α, a member of the TNF superfamily, is a proinflammatory cytokine that binds to TNF receptors 1 and 2 [11,12,13]. TNF-α plays a role in the pathogenesis of inflammatory diseases as well as in carcinogenesis [14,15]. TNF receptor 1 is widely expressed in tissues, whereas TNF receptor 2 expression is more restricted [16,17]. TNF receptor 1 primarily activates the nuclear factor κB (NF-κB) signaling pathway [16,17]. NF-κB transcription factors form dimers from five subunits [18,19]. In unstimulated cells, NF-κB dimers associate with the inhibitor of NF-κB (IκB), which sequesters them in the cytoplasm [20,21,22]. Upon engagement with TNF-α, TNF receptor 1 recruits a set of adaptor proteins, including receptor-interacting protein kinase 1 (RIPK1), TNF receptor-associated death domain protein (TRADD), and TNF receptor-associated factor (TRAF) 2, to its cytoplasmic domain, which is a prerequisite for the activation of the IκB kinase complex [20,21,22]. The IκB kinase complex then phosphorylates IκB, allowing its ubiquitination and subsequent degradation by the proteasome [23,24]. This process releases and translocates NF-κB dimers, including the RelA and p50 heterodimer, to the nucleus, which promotes the transcriptional activation of many genes, including those regulating inflammation and cancer progression [25,26,27].

**Figure 1:**
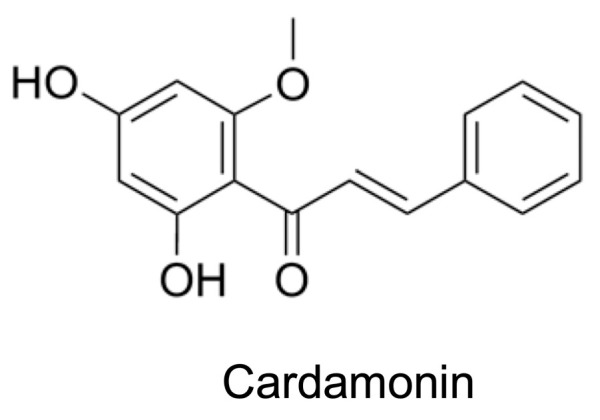
Structure of cardamonin.

Chalcones (1,3-diaryl-2-propen-1-ones) are a type of flavonoid, and their derivatives have been shown to exert various biological effects, including anti-inflammatory and anticancer activities [28,29]. Cardamonin (Figure 1) is a chalcone that is present in cardamon seeds, which are used as a spice, and it is also a component of many other plant species, some of which are edible [30]. Cardamonin is assumed to interfere with multiple signaling pathways associated with chronic diseases, including inflammatory diseases and cancers [30,31,32,33]. In relation to anti-inflammatory activity, cardamonin has been shown to inhibit the NF-κB signaling pathway upstream of IκBα phosphorylation and degradation in lipopolysaccharide (LPS)-stimulated macrophages [34,35,36,37,38,39]. In terms of anticancer activities, cardamonin inhibited the growth of cancer cells by regulating the cell cycle, promoting cell death, and targeting various signaling pathways, including the NF-κB signaling pathway [31,32,33]. Furthermore, cardamonin was found to inhibit TNF-α-induced NF-κB activation in cancer cell lines [40,41,42]. However, the mechanisms by which cardamonin exerts its inhibitory effects have yet to be elucidated.

During our screening-based research, we identified various natural and synthetic compounds targeting NF-κB activation in human lung adenocarcinoma A549 cells and human umbilical vein endothelial cells. We previously reported that three chalcone derivatives (cardamonin, 4-hydroxypanduratin A, and isopanduratin A) inhibited ICAM-1 expression at similar concentrations in TNF-α-stimulated A549 cells [43]. Moreover, isopanduratin A down-regulated TNF receptor 1 expression and blocked its downstream signaling events [44]. Therefore, we investigated the mechanisms by which cardamonin inhibits the NF-κB signaling pathway upon TNF-α stimulation. The results obtained herein demonstrated that cardamonin inhibited the NF-κB signaling pathway in TNF-α-stimulated A549 cells in a manner that was distinct from 4-hydroxypanduratin A and isopanduratin A.

## 2. Results

### 2.1. Cardamonin Inhibited TNF-α-Induced ICAM-1 mRNA Expression in A549 Cells

**Figure 2:**
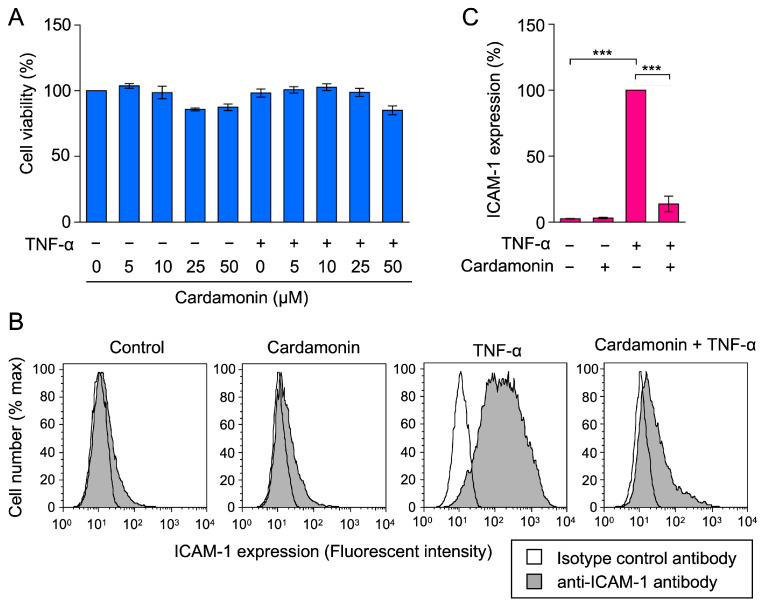
Cardamonin inhibited TNF-α-induced ICAM-1 protein expression. (**A**) A549 cells were treated with cardamonin for 1 h, followed by a 6-h stimulation with (+) or without (−) TNF-α (2.5 ng/mL) in the presence or absence of cardamonin (5–50 µM). Cell viability (%) (blue bars) is presented as the mean ± standard error (*n* = 3). No significant differences were observed. (**B**,**C**) A549 cells were treated with cardamonin for 1 h, followed by a 6-h stimulation with (+) or without (−) TNF-α (2.5 ng/mL) or cardamonin (50 µM). Histograms represent ICAM-1 expression from three independent experiments and are presented as the isotype control antibody (empty area) or anti-ICAM-1 antibody (gray area) (**B**). ICAM-1 expression (%) (magenta bars) is presented as the mean ± standard error (*n* = 3) (**C**). *** *p* < 0.001.

Human lung adenocarcinoma A549 cells are adherent epithelial cells that are stimulated to activate the NF-κB pathway and express adhesion molecules in response to proinflammatory cytokines. In our previous studies [43,44,45,46], we used A549 cells as an immortalized model that exhibits phenotypes similar to those of human endothelial cells, including NF-κB activation and adhesion molecule expression. These cells may be used to examine the effects of anti-inflammatory and anticancer compounds. A549 cells were treated with serial dilutions of cardamonin for 1 h and were then stimulated with TNF-α for 6 h. Crystal violet staining showed that cardamonin did not affect the viability of A549 cells at concentrations up to 50 µM during a 7-h incubation period (Figure 2A). We previously demonstrated that cardamonin inhibited TNF-α-induced ICAM-1 expression at concentrations of 10–50 µM using a cell enzyme-linked immunosorbent assay (ELISA) [43]. In these experiments, the stock solution of cardamonin (50 mM) in dimethyl sulfoxide (DMSO) was diluted by the culture medium to concentrations lower than 0.1%. A concentration of up to 0.5% DMSO did not affect cell viability or ICAM-1 expression (Figure S1A,B), indicating that the residual amount of DMSO was negligible. A flow cytometric analysis confirmed that cardamonin at 50 µM strongly inhibited cell-surface ICAM-1 expression, which was markedly up-regulated by the TNF-α stimulation (Figure 2B,C). These results confirmed that cardamonin inhibited TNF-α-induced ICAM-1 protein expression.

**Figure 3:**
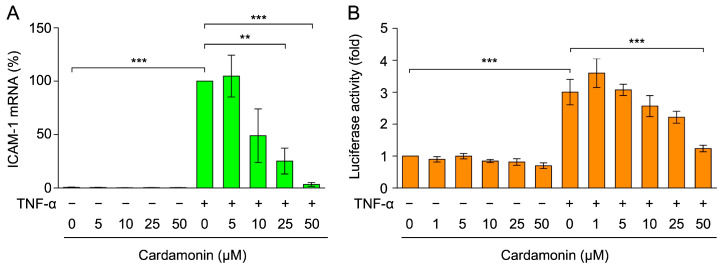
Cardamonin inhibited TNF-α-induced ICAM-1 mRNA expression. (**A**) A549 cells were treated with cardamonin for 1 h, followed by a 2-h stimulation with (+) or without (−) TNF-α (2.5 ng/mL) in the presence or absence of cardamonin (5–50 µM). ICAM-1 mRNA (%) (green bars) is presented as the mean ± standard error (*n* = 3). (**B**) The luciferase gene driven by the ICAM-1 promoter (−1604 to +40) was used for the reporter assay. A549 cells were treated with cardamonin for 1 h, followed by a 2.5-h stimulation with (+) or without (−) TNF-α (2.5 ng/mL) in the presence or absence of cardamonin (1–50 µM). Luciferase activity (fold) (orange bars) is presented as the mean ± standard error (*n* = 3). ** *p* < 0.01 and *** *p* < 0.001.

TNF-α has been shown to predominantly up-regulate the expression of ICAM-1 at the mRNA level [47,48]. Therefore, A549 cells were treated with cardamonin for 1 h, stimulated with TNF-α for 2 h, and total RNA was then isolated. The effects of cardamonin on ICAM-1 mRNA expression were investigated by a quantitative polymerase chain reaction (PCR). TNF-α markedly increased ICAM-1 mRNA expression, while cardamonin reversed this effect at concentrations of 10–50 µM (Figure 3A).

To further examine the effects of cardamonin on ICAM-1 mRNA expression, A549 cells were transiently transfected with a luciferase gene driven by the ICAM-1 promoter and then subjected to a reporter assay. A stimulation with TNF-α for 2.5 h increased luciferase activity by approximately 3-fold, and cardamonin reversed this effect in a dose-dependent manner (Figure 3B). These results show that cardamonin inhibited TNF-α-induced ICAM-1 mRNA expression.

### 2.2. Cardamonin Did Not Affect TNF-α-Induced IκBα Degradation in A549 Cells

**Figure 4:**
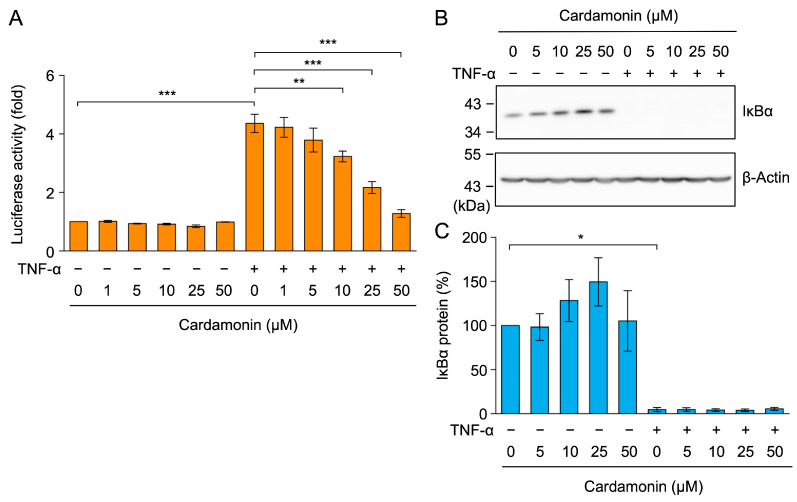
Cardamonin did not affect TNF-α-induced IκBα degradation. (**A**) The NF-κB-responsive luciferase gene was used for the reporter assay. A549 cells were treated with cardamonin for 1 h, followed by a 2.5-h stimulation with (+) or without (−) TNF-α (2.5 ng/mL) in the presence or absence of cardamonin (1–50 µM). Luciferase activity (fold) (orange bars) is presented as the mean ± standard error (*n* = 3). (**B**,**C**) A549 cells were treated with cardamonin for 1 h, followed by a 15-min stimulation with (+) or without (−) TNF-α (2.5 ng/mL) in the presence or absence of cardamonin (5–50 µM). Representative blots from three independent experiments are displayed (**B**). The IκBα protein (%) (cyan bars) is presented as the mean ± standard error (*n* = 3) (**C**). * *p* < 0.05, ** *p* < 0.01, and *** *p* < 0.001. Original blots are presented in Figures S2–S5.

TNF-α-induced ICAM-1 mRNA expression has been shown to primarily depend on NF-κB [47,48]. To examine the effects of cardamonin on NF-κB-dependent transcription, A549 cells were transfected with a luciferase reporter gene fused to two NF-κB response elements. The TNF-α stimulation increased luciferase activity by approximately 4-fold (Figure 4A). Cardamonin reduced TNF-α-induced NF-κB-dependent increases in luciferase activity at concentrations of 10–50 µM (Figure 4A). These results suggest that cardamonin affected the NF-κB signaling pathway.

TNF-α induces the activation of IκB kinases, which phosphorylate IκBα and allow for its ubiquitination and rapid degradation by proteasomes [23,24]. A549 cells were treated with cardamonin for 1 h and then stimulated with TNF-α for 15 min. In TNF-α-stimulated A549 cells, IκBα decreased to basal levels (Figure 4B,C). Cardamonin did not affect the TNF-α-induced degradation of IκBα at concentrations up to 50 µM (Figure 4B,C).

### 2.3. Cardamonin Inhibited the TNF-α-Induced Nuclear Translocation of RelA in A549 Cells

**Figure 5:**
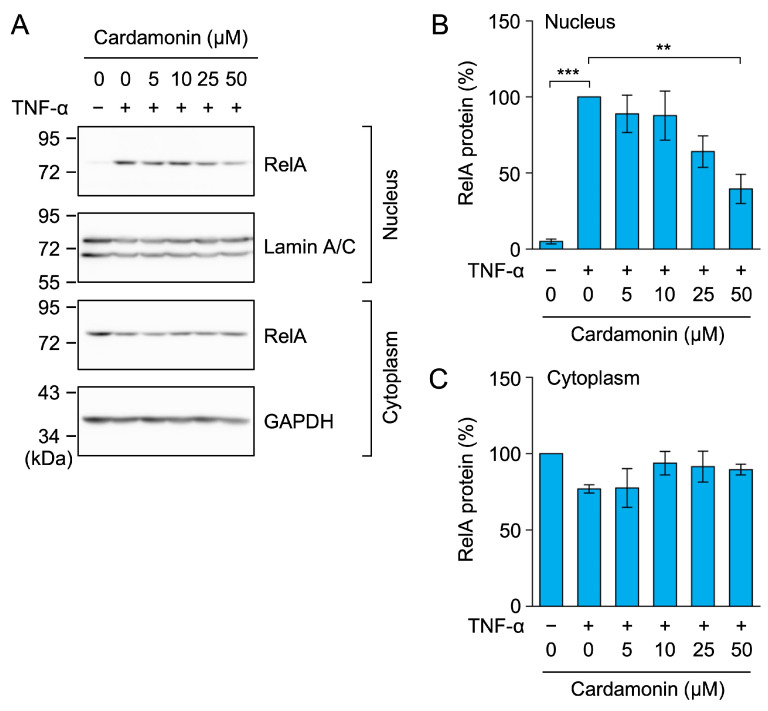
Cardamonin inhibited the TNF-α-induced nuclear translocation of RelA. (**A**–**C**) A549 cells were treated with cardamonin for 1 h, followed by a 30-min stimulation with (+) or without (−) TNF-α (2.5 ng/mL) in the presence or absence of cardamonin (5–50 µM). Representative blots from four independent experiments are displayed (**A**). RelA protein (%) in the nucleus (**B**) and cytoplasm (**C**) (cyan bars) are presented as the mean ± standard error (*n* = 4). ** *p* < 0.01 and *** *p* < 0.001. Original blots are presented in Figures S6–S15.

Based on the results described above, we investigated the impact of cardamonin on the NF-κB signaling pathway following the degradation of IκBα. Upon IκBα degradation, NF-κB subunits are released and translocate from the cytoplasm to the nucleus [23,24]. In TNF-α-stimulated A549 cells, the amount of RelA markedly increased in the nuclear fraction (Figure 5A,B), but slightly decreased in the cytoplasmic fraction (Figure 5A,C). The TNF-α-induced increase in RelA in the nuclear fraction was reduced by cardamonin in a dose-dependent manner (Figure 5A,B). These results show that cardamonin inhibited the TNF-α-induced nuclear translocation of RelA.

### 2.4. Cardamonin Did Not Affect TNF-α-Induced IκBα Degradation, but Inhibited the Nuclear Translocation of RelA in MCF-7 and HT-1080 Cells

**Figure 6:**
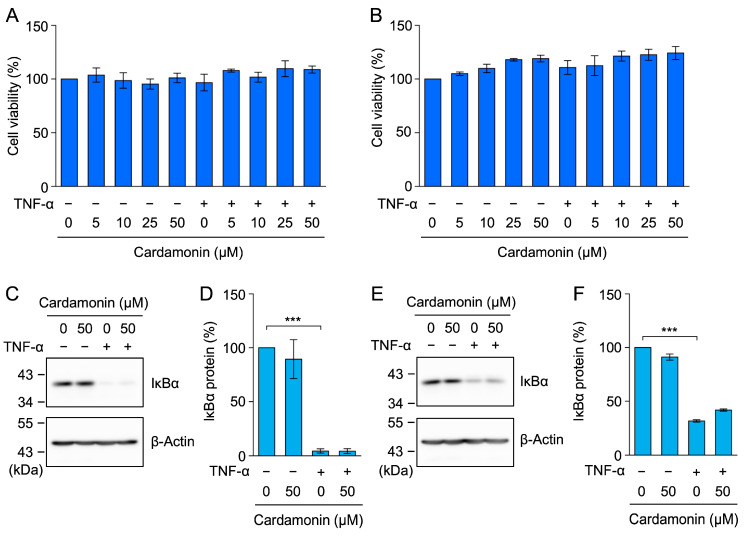
Cardamonin did not block TNF-α-induced IκBα degradation in MCF-7 or HT-1080 cells. (**A**,**B**) MCF-7 cells (**A**) and HT-1080 cells (**B**) were treated with cardamonin for 1 h, followed by a 6-h stimulation with (+) or without (−) TNF-α (2.5 ng/mL) in the presence or absence of cardamonin (5–50 µM). Cell viability (%) (blue bars) is presented as the mean ± standard error (*n* = 3). No significant differences were observed. (**C**–**F**) MCF-7 cells (**C**,**D**) and HT-1080 cells (**E**,**F**) were treated with cardamonin for 1 h, followed by a 15-min stimulation with (+) or without (−) TNF-α (2.5 ng/mL) in the presence or absence of cardamonin (50 µM). Representative blots from three independent experiments are displayed (**C**,**E**). The IκBα protein (%) (cyan bars) is presented as the mean ± standard error (*n* = 3) (**D**,**F**). *** *p* < 0.001. Original blots are presented in Figures S16–S23.

To confirm the results obtained from A549 cells, we used the following cell lines: human breast cancer MCF-7 cells and human fibrosarcoma HT-1080 cells, both of which respond to TNF-α by inducing the activation of NF-κB. Cardamonin at concentrations up to 50 µM did not affect the viability of MCF-7 or HT-1080 cells (Figure 6A,B). TNF-α induced IκBα degradation in MCF-7 and HT-1080 cells, and cardamonin did not block this process (Figure 6C–F).

**Figure 7:**
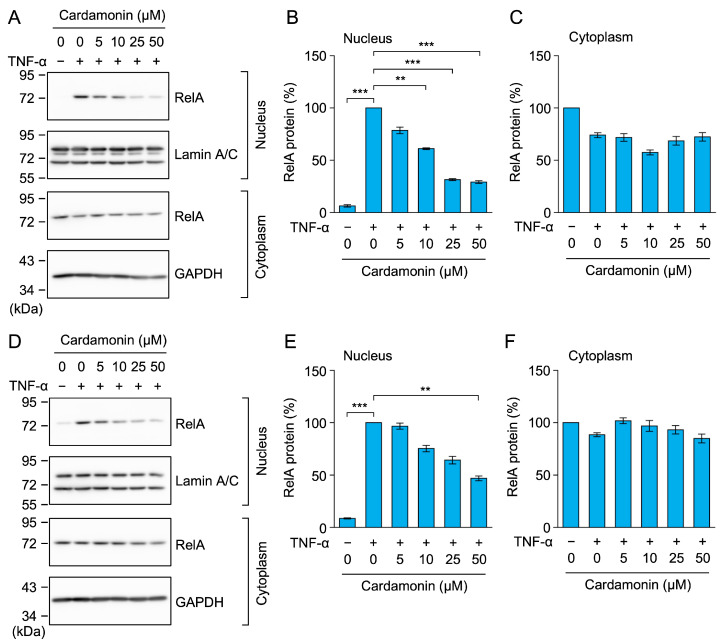
Cardamonin inhibited the TNF-α-induced nuclear translocation of RelA in MCF-7 and HT-1080 cells. (**A**–**F**) MCF-7 cells (**A**–**C**) and HT-1080 cells (**D**–**F**) were treated with cardamonin for 1 h, followed by a 30-min stimulation with (+) or without (−) TNF-α (2.5 ng/mL) in the presence or absence of cardamonin (5–50 µM). Representative blots from three independent experiments are displayed (**A**,**D**). RelA protein (%) in the nucleus (**B**,**E**) and cytoplasm (**C**,**F**) (cyan bars) are presented as the mean ± standard error (*n* = 3). ** *p* < 0.01 and *** *p* < 0.001. Original blots are presented in Figures S24–S39.

MCF-7 and HT-1080 cells were pretreated with cardamonin for 1 h and then stimulated with TNF-α for 30 min. RelA was translocated from the cytoplasm to the nucleus in both cell lines (Figure 7A–F). Cardamonin inhibited the TNF-α-induced accumulation of RelA in the nucleus of MCF-7 cells (Figure 7A,B) and HT-1080 cells (Figure 7D,E). These results confirm that cardamonin did not affect TNF-α-induced IκBα degradation, but inhibited nuclear RelA translocation.

### 2.5. Cardamonin Inhibited TNF-α-Induced RelA Binding to the ICAM-1 Promoter in A549 Cells

**Figure 8:**
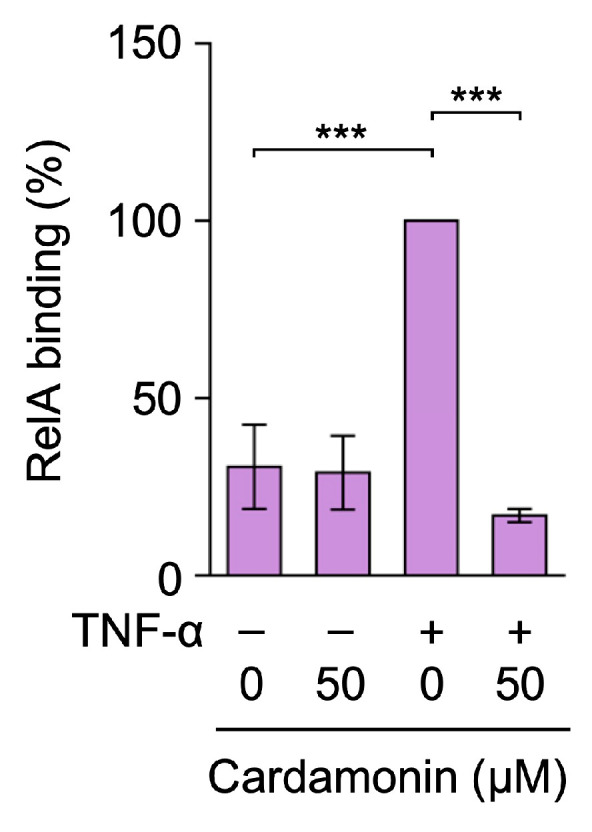
Cardamonin inhibited TNF-α-induced RelA binding to the ICAM-1 promoter. A549 cells were treated with cardamonin for 1 h, followed by a 30-min stimulation with (+) or without (−) TNF-α (2.5 ng/mL) or cardamonin (50 µM). RelA binding (%) (purple bars) is presented as the mean ± standard error (*n* = 3). *** *p* < 0.001.

The ICAM-1 promoter contains multiple NF-κB response elements [47,48]. We previously reported that TNF-α promoted RelA binding to the ICAM-1 promoter in the proximal region (−286 to −90) in A549 cells [49,50]. Therefore, we investigated the effects of cardamonin on RelA binding to the ICAM-1 promoter using a chromatin immunoprecipitation (ChIP) assay. The TNF-α stimulation augmented RelA binding to the ICAM-1 promoter (−286 to −90) (Figure 8). Cardamonin at 50 µM strongly inhibited RelA binding to the ICAM-1 promoter (Figure 8). Therefore, cardamonin suppressed RelA binding to the ICAM-1 promoter.

### 2.6. 4′-Hydroxychalcone, Isoliquiritigenin, and Xanthohumol Inhibited TNF-α-Induced ICAM-1 Expression in A549 Cells

**Figure 9:**
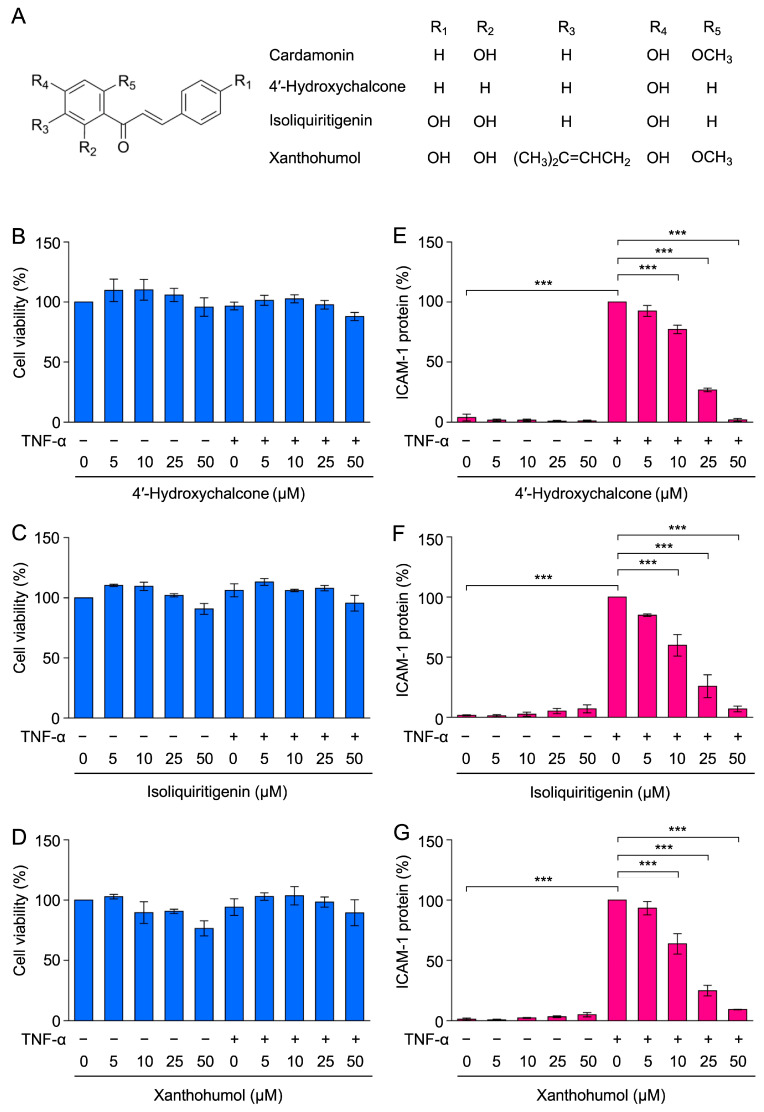
Chalcone derivatives inhibited TNF-α-induced ICAM-1 expression. (**A**) Structures of 4′-hydroxychalcone, isoliquiritigenin, and xanthohumol. (**B**–**G**) A549 cells were treated with 4′-hydroxychalcone (**B**,**E**), isoliquiritigenin (**C**,**F**), and xanthohumol (**D**,**G**) for 1 h, followed by a 6-h stimulation with (+) or without (−) TNF-α (2.5 ng/mL) in the presence or absence of 4′-hydroxychalcone, isoliquiritigenin, and xanthohumol (5–50 µM). Cell viability (%) (blue bars) is presented as the mean ± standard error (*n* = 3) (**B**–**D**). No significant differences were observed (**B**–**D**). ICAM-1 expression (%) (magenta bars) is presented as the mean ± standard error (*n* = 3) (**E**–**G**). *** *p* < 0.001.

Chalcones possess a common backbone of 1,3-diaryl-2-propen-1-ones. In addition to cardamonin, chalcone derivatives have been reported to inhibit the activation of NF-κB [28,29]. Among commercially available chalcone derivatives, 4′-hydroxychalcone, isoliquiritigenin, and xanthohumol have two side chain replacements relative to cardamonin (Figure 9A). The biological effects of three chalcone derivatives on TNF-α-induced ICAM-1 expression and the NF-κB signaling pathway were compared. A549 cells were treated with these chalcone derivatives for 1 h and were then stimulated with TNF-α for 6 h. Crystal violet staining showed that the viability of A549 cells was not markedly affected by any of the chalcone derivatives during the 7-h incubation (Figure 9B–D).

In cell ELISA experiments, ICAM-1 expression was inhibited by 4′-hydroxychalcone, isoliquiritigenin, and xanthohumol at concentrations of 10–50 µM (Figure 9E–G). The inhibitory activities of 4′-hydroxychalcone, isoliquiritigenin, and xanthohumol were similar to that of cardamonin in our previous study [43].

### 2.7. 4′-Hydroxychalcone, Isoliquiritigenin, and Xanthohumol Did Not Markedly Inhibit TNF-α-Induced IκBα Degradation in A549 Cells

**Figure 10:**
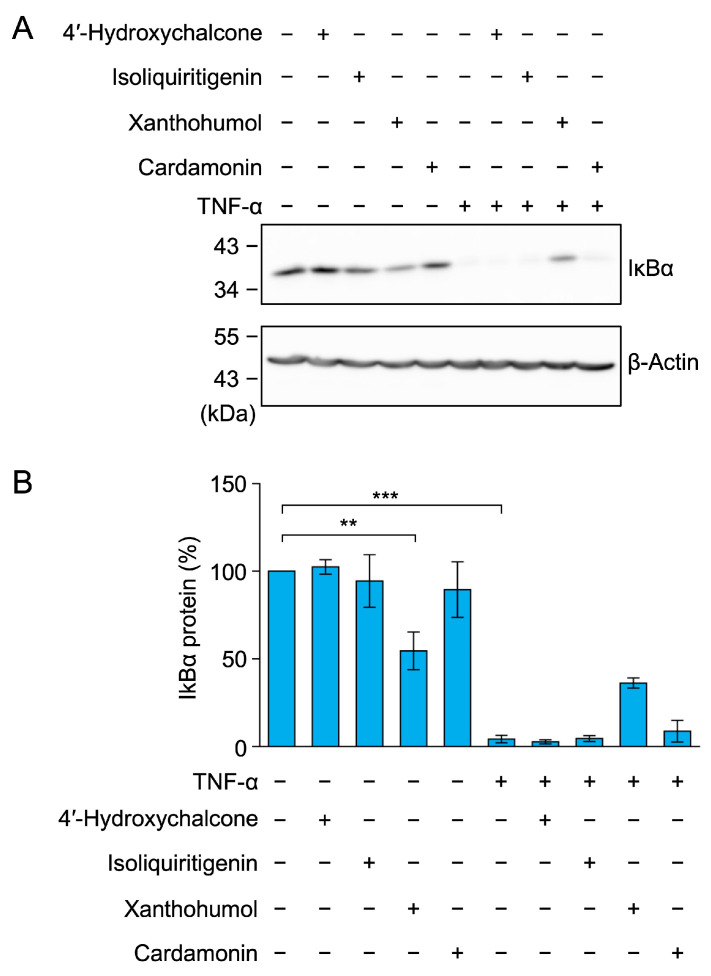
4′-Hydroxychalcone and isoliquiritigenin did not affect TNF-α-induced IκBα degradation. (**A**,**B**) A549 cells were treated with 4′-hydroxychalcone, isoliquiritigenin, and xanthohumol for 1 h, followed by a 15-min stimulation with (+) or without (−) TNF-α (2.5 ng/mL) in the presence or absence of the compounds (each at 50 µM). Representative blots from three independent experiments are displayed (**A**). IκBα protein (%) (cyan bars) is presented as the mean ± standard error (*n* = 3) (**B**). ** *p* < 0.01 and *** *p* < 0.001. Original blots are presented in Figures S40–S43.

We then investigated the effects of chalcone derivatives on TNF-α-induced IκBα degradation. The TNF-α stimulation decreased the amount of IκBα to basal levels (Figure 10A,B). 4′-Hydroxychalcone and isoliquiritigenin did not inhibit TNF-α-induced IκBα degradation (Figure 10A,B). In contrast, xanthohumol alone significantly decreased the amount of IκBα, and slightly inhibited TNF-α-induced IκBα degradation (Figure 10A,B). These results show that 4′-hydroxychalcone, isoliquiritigenin, and xanthohumol did not markedly inhibit TNF-α-induced IκBα degradation.

### 2.8. 4′-Hydroxychalcone, Isoliquiritigenin, and Xanthohumol Inhibited TNF-α-Induced RelA Nuclear Translocation in A549 Cells

**Figure 11:**
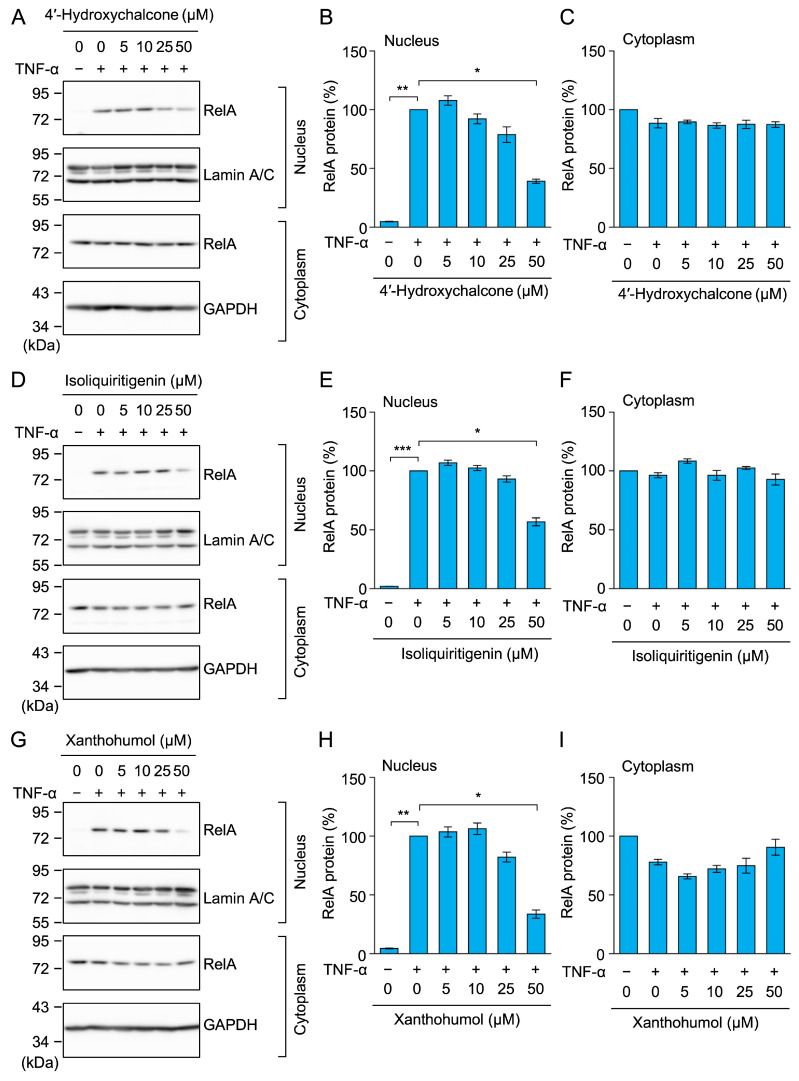
4′-Hydroxychalcone, isoliquiritigenin, and xanthohumol inhibited TNF-α-induced RelA nuclear translocation. (**A**–**I**) A549 cells were treated with 4′-hydroxychalcone (**A**–**C**), isoliquiritigenin (**D**–**F**), and xanthohumol (**G**–**I**) for 1 h, followed by a 30-min stimulation with (+) or without (−) TNF-α (2.5 ng/mL) in the presence or absence of the compounds (5–50 µM). Representative blots from three independent experiments are displayed (**A**,**D**,**G**). RelA protein (%) in the nucleus (**B**,**E**,**H**) and cytoplasm (**C**,**F**,**I**) (cyan bars) are presented as the mean ± standard error (*n* = 3). * *p* < 0.05, ** *p* < 0.01, and *** *p* < 0.001. Original blots are presented in Figures S44–S67.

We also examined the effects of chalcone derivatives on the nuclear translocation of RelA. Following the TNF-α stimulation, the amount of RelA markedly increased in the nuclear fractions, but remained unchanged in the cytoplasmic fractions (Figure 11A–I). At a concentration of 50 µM, 4′-hydroxychalcone, isoliquiritigenin, and xanthohumol inhibited the TNF-α-induced nuclear translocation of RelA (Figure 11A,B,D,E,G,H).

### 2.9. Cardamonin Inhibited the Nuclear Translocation of the RelA C38S Mutant

RelA possesses a cysteine residue at position 38 (C38), which is covalently modified by many compounds via the Michael addition reaction [51,52,53]. The alkylation of RelA C38 inhibits its nuclear translocation and transcriptional activity, while the replacement of C38 with serine prevents its alkylation and thereby confers resistance to these compounds [51,52,53]. We previously showed that santonin-related compound 2 (SRC2), a sesquiterpene lactone, inhibited the nuclear translocation of wild-type (WT) RelA, but not the RelA C38S mutant in TNF-α-stimulated A549 cells [54].

**Figure 12:**
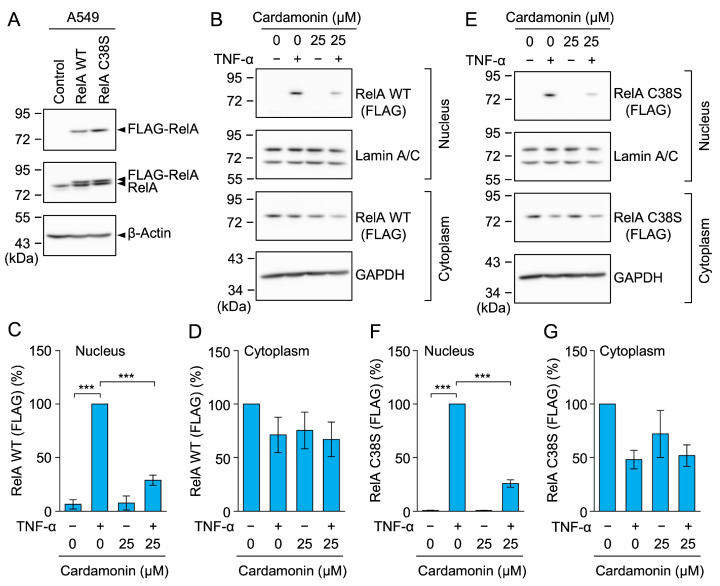
Cardamonin inhibited the TNF-α-induced nuclear translocation of the RelA C38S mutant. (**A**) FLAG-RelA expression in non-transfected A549 cells (Control) and A549 cells stably expressing FLAG-RelA WT and FLAG-RelA C38S. Representative blots from two independent experiments are displayed. (**B**–**G**) A549 cells stably expressing FLAG-RelA WT (**B**–**D**) and FLAG-RelA C38S (**E**–**G**) were treated with or without cardamonin for 1 h, followed by a 30-min stimulation with (+) or without (−) TNF-α (2.5 ng/mL) or cardamonin (25 µM). Representative blots from three independent experiments are displayed (**B**,**E**). RelA protein (%) in the nucleus (**C**,**F**) and cytoplasm (**D**,**G**) (cyan bars) are presented as the mean ± standard error (*n* = 3). *** *p* < 0.001. Original blots are presented in Figures S68–S84.

Cardamonin contains an *α,β*-unsaturated carbonyl moiety that undergoes a Michael addition reaction with cysteine residues [55]. Therefore, we investigated whether cardamonin directly targeted the C38 of RelA to prevent its nuclear translocation. Lentivirus transfection was used to establish A549 cells stably expressing the FLAG-tagged RelA WT and RelA C38S mutant. In Western blotting, similar FLAG-RelA WT and FLAG-RelA C38S levels were observed in stable A549 transfectants (Figure 12A). Cardamonin at 25 µM inhibited the TNF-α-induced nuclear translocation of FLAG-RelA WT (Figure 12B–D). Unexpectedly, cardamonin at 25 µM also inhibited the nuclear translocation of FLAG-RelA C38S (Figure 12E–G). These results suggest that cardamonin did not primarily target C38 in RelA.

### 2.10. In Silico Molecular Docking Showed a Potential Interaction Between Cardamonin and RelA

**Figure 13:**
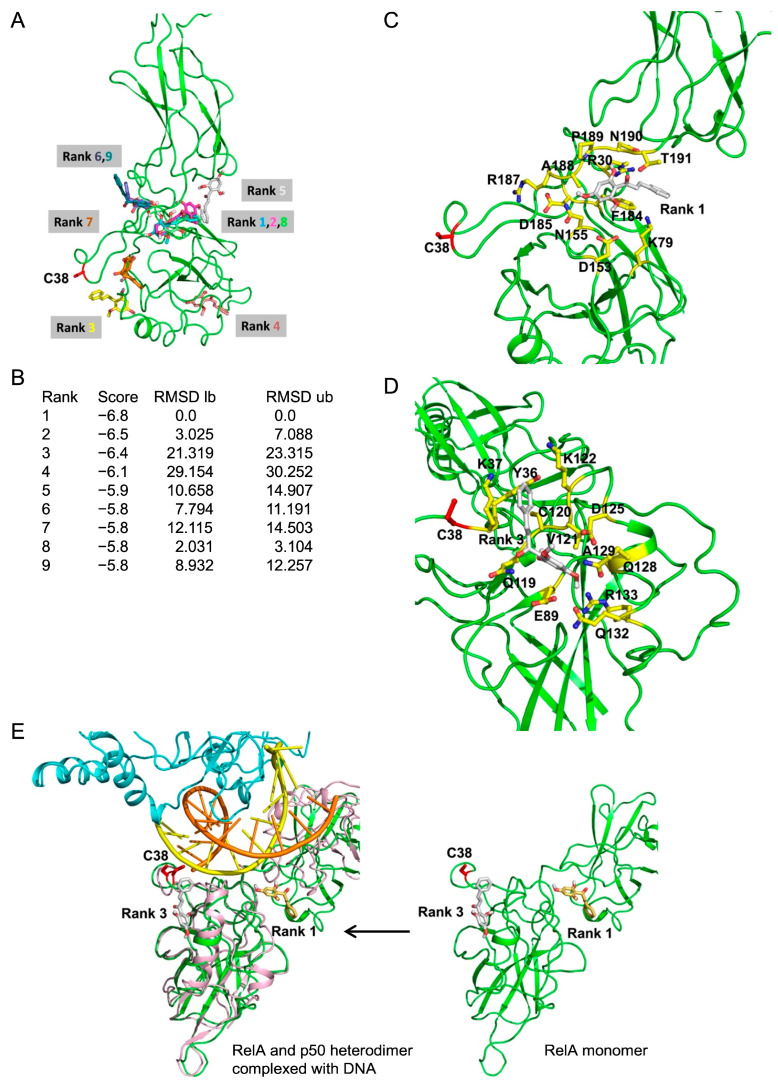
Cardamonin potentially interacted with RelA. (**A**) Rank 1 to Rank 9 models are shown in different colors. The position of C38 is indicated. (**B**) The rank, score (binding free energy; kcal/mol), and root mean square deviation (RMSD) lower bound (lb) and upper bound (ub) are presented for the Rank 1 to Rank 9 models. (**C**,**D**) The positions of cardamonin and amino acid residues located within 4 Å of cardamonin are shown in the Rank 1 model (**C**) and Rank 3 model (**D**). (**E**) The positions of cardamonin in the Rank 1 and Rank 3 models are presented in the RelA monomer (green) and the RelA and p50 heterodimer (RelA: pink; p50: cyan) complexed with DNA (DNA strands: yellow and orange).

Chalcones, such as cardamonin, contain an *α,β*-unsaturated carbonyl moiety that may undergo a Michael addition reaction with cysteine residues [55]. Previous studies demonstrated that a number of Michael acceptor compounds directly inhibited RelA by alkylating C38 [51,52,53]. However, our cell-based results showed that cardamonin did not primarily target C38 in RelA. Therefore, we performed an *in silico* molecular docking analysis to identify the positions at which cardamonin potentially binds to human RelA. Nine potential docking models between RelA and cardamonin with scores ≥ −5.8 kcal/mol are shown in Figure 13A,B. In the Rank 1 and Rank 2 models, cardamonin interacted with RelA through similar positions (Figure 13A). In the Rank 1 model, cardamonin was located in the pocket with a score of −6.8 kcal/mol by interacting with 11 amino acid residues (R30, K79, D153, N155, F184, D185, A188, R187, P189, N190, and T191) (Figure 13C). In the Rank 3 model, cardamonin was positioned near RelA C38 with a score of −6.4 kcal/mol by interacting with twelve amino acid residues (Y36, K37, E89, Q119, C120, V121, K122, D125, Q128, A129, Q132, and R133) (Figure 13D). However, cardamonin did not appear to be close enough to RelA C38 to mediate covalent binding via its *α, β*-unsaturated carbonyl moiety (Figure 13D).

We also examined the binding modes of cardamonin by comparing them with the crystal structure of the murine RelA and p50 heterodimer complexed with DNA [56]. In the Rank 1 and Rank 3 models, cardamonin was found near the contact sites of RelA with a DNA strand (Figure 13E). RelA C38 was positioned very close to a DNA strand (Figure 13E), suggesting that the alkylation of C38 directly hindered the interaction with DNA. The structure of RelA markedly changed when it formed complexes with p50 and DNA (Figure 13E). Notably, the DNA contact region, which included the cardamonin-binding site in the Rank 1 model, underwent a prominent structural change (Figure 13E). In contrast, the cardamonin-binding site in the Rank 3 model only slightly changed (Figure 13E). These results suggest that cardamonin prevented the structural changes necessary for RelA to bind to DNA when it was bound to RelA in the Rank 1 model.

### 2.11. RelA R30A/N155A/T191A and Y36A/K37A/K122A Mutants Did Not Undergo Nuclear Translocation in Response to the TNF-α Stimulation

**Figure 14:**
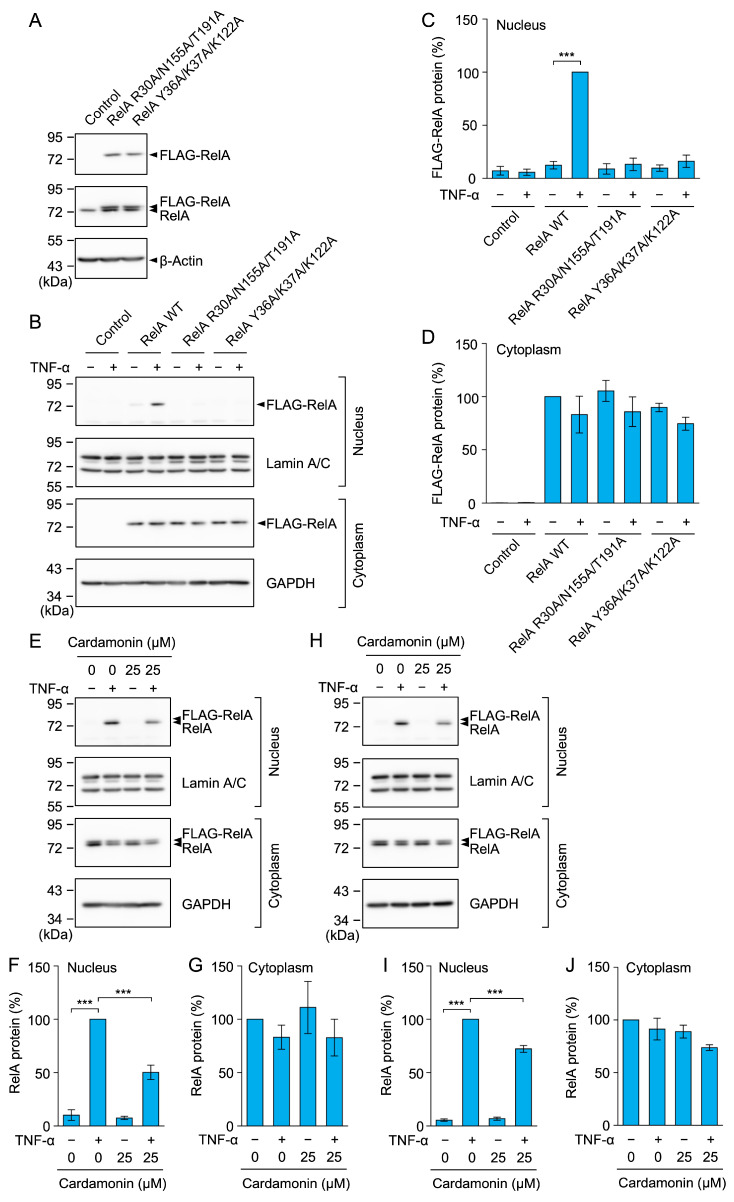
RelA R30A/N155A/T191A and Y36A/K37A/K122A mutants did not undergo nuclear translocation. (**A**) FLAG-RelA expression in non-transfected A549 cells (Control) and A549 cells stably expressing FLAG-RelA R30A/N155A/T191A and Y36A/K37A/K122A. Representative blots from two independent experiments are displayed. (**B**–**J**) A549 cells stably expressing FLAG-RelA WT, FLAG-RelA R30A/N155A/T191A, and Y36A/K37A/K122A were treated with (+) or without (−) TNF-α (2.5 ng/mL) for 30 min (**B**–**D**). A549 cells stably expressing FLAG-RelA R30A/N155A/T191A (**E**–**G**) and Y36A/K37A/K122A (**H**–**J**) were treated with or without cardamonin for 1 h, followed by a 30-min stimulation with (+) or without (−) TNF-α (2.5 ng/mL) or cardamonin (25 µM). Representative blots from three independent experiments are displayed (**B**,**E**,**H**). The FLAG-RelA and RelA bands were quantitated together (**F**,**G**,**I**,**J**). RelA protein (%) in the nucleus (**C**,**F**,**I**) and cytoplasm (**D**,**G**,**J**) (cyan bars) are presented as the mean ± standard error (*n* = 3). *** *p* < 0.001. Original blots are presented in Figures S85–S109.

Based on the interaction between cardamonin and RelA, we designed and constructed RelA mutants in which amino acid residues were replaced with alanine. In the Rank 1 model, the R30A/N155A/T191A mutations did not form hydrogen bonds with cardamonin. In the Rank 3 model, the Y36A/K37A/K122A mutations reduced the hydrophobic interaction with cardamonin. A549 cells were stably transfected with these FLAG-RelA mutants by retrovirus transfection. In the Western blotting analysis, FLAG-RelA mutants were found to be expressed at similar levels (Figure 14A). Unlike FLAG-RelA WT, FLAG-RelA R30A/N155A/T191A and Y36A/K37A/K122A barely underwent nuclear translocation in response to the TNF-α stimulation (Figure 14B–D). In A549 cells stably expressing the RelA mutants, endogenous RelA translocated to the nucleus, and this was inhibited by cardamonin (Figure 14E–J). While these results do not directly confirm the RelA-binding sites of cardamonin, the predicted cardamonin-binding sites in the Rank 1 and Rank 3 models appear to be essential for nuclear translocation.

## 3. Discussion

**Figure 15:**
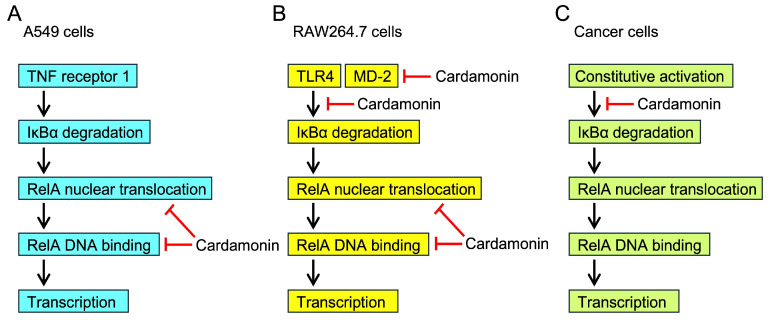
Inhibitory mechanisms of cardamonin on the NF-κB signaling pathway. (**A**–**C**) The RelA and p50 heterodimer associates with IκBα. Upon the stimulation, IκBα is phosphorylated and rapidly degraded by the ubiquitin-proteasome system. The RelA and p50 heterodimer is then released and translocates to the nucleus where it binds to the promoter regions of target genes and promotes their transcription. The inhibitory mechanisms of cardamonin reported in previous studies and herein involve the TNF receptor 1-dependent NF-κB signaling pathway in A549 cells (**A**), the TLR4-dependent NF-κB signaling pathway in RAW264.7 cells (**B**), and the constitutive NF-κB signaling pathway in cancer cells (**C**). The red lines indicate the inhibitory steps by cardamonin.

The NF-κB pathway is activated by distinct sets of adaptor proteins that are recruited to cell-surface receptors in response to proinflammatory cytokines or Toll-like receptor (TLR) ligands. Previous studies showed that cardamonin prevented the LPS-induced NF-κB signaling pathway in macrophages [34,35,36,37,38,39]. Cardamonin has also been reported to inhibit TNF-α-induced NF-κB activation in cancer cells [40,41,42]. We previously used TNF-α-stimulated A549 cells to investigate the molecular mechanisms of different compounds (e.g., isopanduratin A, alantolactone derivatives, porphyrin derivatives, quinacrine, *α*-conidendrin, and santonin-related compound 2), which were shown to inhibit either processes upstream of IκB degradation or the levels of Rel A nuclear translocation or RelA DNA binding [43,44,45,46,49,50,54]. The present results demonstrated that cardamonin did not affect IκBα degradation, but inhibited the nuclear translocation of RelA at concentrations of 10 to 50 µM and its binding to the ICAM-1 promoter in TNF-α-stimulated A549 cells (Figure 15A). Consistent with the present results, the effective concentrations of cardamonin were shown to be in the range of 10–50 µM in different cell types [35,36,37,38,39,40,41,42]. Following its oral administration, cardamonin exhibited low bioavailability in the serum of rats and mice [57,58]. As discussed in recent reviews [31,32,33], more studies are needed to increase the bioavailability of cardamonin and assess its serum concentration.

TLR4 is associated with myeloid differentiation factor 2 (MD-2) on the cell surface, and the TLR4/MD-2 complex recognizes LPS [59,60]. Upon binding to LPS, TLR4 recruits and activates a set of adaptor proteins, including MyD88, interleukin-1 receptor-associated kinase, and TRAF6, which leads to the activation of the IκB kinase complex phosphorylating IκBα [61,62]. Phosphorylated IκBα undergoes rapid degradation by the ubiquitin-proteasome system [23,24]. Previous studies showed that cardamonin interfered with the NF-κB signaling pathway in murine RAW264.7 macrophages in response to LPS or LPS/interferon-γ (IFN-γ) [34,35,36,37,38,39]. Cardamonin has been shown to inhibit the phosphorylation and degradation of IκBα in LPS- or LPS/IFN-γ-stimulated RAW264.7 cells [34,36]. Dimethyl cardamonin exhibited similar biological activity [63]. In addition to these inhibitory effects, the following findings were obtained from LPS-stimulated RAW264.7 cells: cardamonin prevented RelA nuclear translocation [37], did not affect the phosphorylation or degradation of IκBα, but inhibited NF-κB DNA binding [35], and directly interacted with MD-2 as an initial molecular target [39]. These findings showed the multiple mechanisms by which cardamonin primarily inhibits processes involving MD-2, IκB, and RelA in the NF-κB signaling pathway of LPS-stimulated RAW264.7 cells (Figure 15B).

Cardamonin has been shown to suppress the proliferation of and induce apoptosis in various cancers by targeting multiple signaling pathways [31,32,33]. In cancer cells, NF-κB often up-regulates target genes that promote cell proliferation and prevent apoptosis [25,26,27]. NF-κB is constitutively activated by a number of mechanisms in cancer cells [64,65,66]. Cardamonin has been shown to inhibit IκB phosphorylation and its downstream processes in several cancer cell types, including human multiple myeloma cells [67], human breast cancer stem cells [68], and human ovarian cancer cells [69]. Therefore, cardamonin appears to inhibit the NF-κB signaling pathway upstream of IκB degradation in these cancer cells (Figure 15C). However, in contrast to these cancer cells, NF-κB activation may only be at the basal level in A549 cells because we previously showed that the expression of NF-κB-responsive target genes, such as ICAM-1, was negligible in unstimulated A549 cells [50,70]. Cardamonin was found to inhibit proliferation, arrest the cell cycle, and induce apoptosis mainly by modulating the mammalian target of rapamycin (mTOR) pathway in A549 cells [71,72,73]. Therefore, in contrast to the NF-κB signaling pathway, the mTOR signaling pathway plays a major role in the proliferation and survival of A549 cells. Cardamonin has been shown to inhibit multiple cellular signaling pathways, including the phosphatidylinositol 3-kinase pathway, the Janus kinase-signal transducer and activation of transcription pathway, and the β-catenin pathway [31,32,33]. These pathways crosstalk with and promote the activation of the NF-κB pathway [27]. We speculate that cardamonin prevents the activation of these upstream signaling pathways, which are required for the constitutive activation of NF-κB.

Upon TNF-α binding, TNF receptor 1 recruits the adaptor proteins TRADD, RIPK1, and TRAF2 to its cytoplasmic domain, thereby inducing the activation of the IκB kinase complex [21,22]. The degradation of IκBα allows the release and nuclear translocation of the NF-κB heterodimer containing RelA [23,24]. Cardamonin was previously shown to inhibit TNF-α-induced NF-κB reporter activity in human embryonic kidney 293 cells [41]. It also suppressed TNF-α-induced nuclear RelA translocation in human hepatocellular carcinoma HepG2 cells [40] and human ovarian cancer SKOV3 cells [42]. We found that cardamonin did not affect IκBα degradation, but inhibited the nuclear translocation of RelA and its binding to the ICAM-1 promoter in A549 cells. Three other chalcones had inhibitory profiles that were similar to that of cardamonin. Based on these results, we herein demonstrated that cardamonin selectively interfered with the NF-κB signaling pathway downstream of IκBα degradation, and inhibited the nuclear translocation and DNA binding of RelA (Figure 15A). These results are consistent with previous findings showing that cardamonin inhibited NF-κB DNA binding in LPS-stimulated RAW264.7 cells and their nuclear extracts [35].

In the NF-κB pathway, IκB kinases and NF-κB subunits are often alkylated by *α,β*-unsaturated carbonyl compounds. The alkylation of C179 in IκB kinase β has been shown to inhibit its activation by preventing the phosphorylation of nearby serine residues located in the activation domain [74]. Conversely, the alkylation of C38 in RelA suppressed its nuclear translocation and DNA-binding activity [51,52,53]. The replacement of these cysteines does not affect the biological activities of IκB kinase β or RelA; however, it confers resistance to alkylation. We previously reported that the sesquiterpene lactone SRC2 inhibited the TNF-α-induced nuclear translocation of RelA WT, but not the RelA C38S mutant in A549 cells, indicating that C38 is a direct target of SRC2 [54]. Cardamonin contains an *α,β*-unsaturated carbonyl moiety that undergoes a Michael addition reaction with cysteine residues [55]. In the present study, cardamonin did not affect the TNF-α-induced degradation of IκBα, but inhibited RelA activity in A549 cells, suggesting that it did not directly affect IκB kinase β, but rather RelA. Further experiments showed that cardamonin inhibited the TNF-α-induced nuclear translocation of both RelA WT and the RelA C38S mutant in A549 cells, suggesting that RelA C38 is not the primary target of cardamonin.

*In silico* molecular docking was performed to investigate the potential interaction between cardamonin and RelA. In the Rank 3 model, cardamonin bound to RelA with −6.4 kcal/mol at a site near C38 by interacting with Y36, K37, E89, Q119, C120, V121, K122, D125, Q128, A129, Q132, and R133, but was not close enough for covalent binding to C38 via the Michael addition reaction. One group demonstrated that cardamonin bound to RelA with a binding energy of −6.5 kcal/mol by interacting with C38, K37, K122, D125, Q127, A129, Q132, and R133 [75]. This position of cardamonin is similar to the Rank 3 model because seven amino acids are common to binding, while it is susceptible to C38 alkylation, in contrast to the Rank 3 model. However, our cell-based experiments showed that cardamonin inhibited the nuclear translocation of the RelA C38S mutant, suggesting that C38 alkylation alone is not a primary mechanism for cardamonin to inhibit RelA.

In the Rank 1 model, cardamonin interacted with R30, K79, D153, N155, F184, D185, A188, R187, P189, N190, and T191, positioning it in a pocket near the binding site between RelA and a DNA strand. Cardamonin has been reported to bind to RelA with a free binding energy of −7.28 kcal/mol by interacting with T191, L194, K195, I196, E282, and M284 [76]. This position of cardamonin is near that in the Rank 1 model because T191 is the common amino acid mediating the interaction between cardamonin and RelA. A previous study based on the crystal structures of the RelA and p50 heterodimer complexed with DNA reported that R187 formed hydrogen bonds with a specific base in the κB DNA sequence [56]. In comparison with the structures of the RelA monomer and the RelA and p50 heterodimer complexed with DNA, a RelA region that includes a cardamonin-binding site appears to undergo a more pronounced structural change when it forms the complex with DNA. Therefore, our docking model hypothesizes that cardamonin directly inhibited the RelA structural change required for DNA binding, thereby inhibiting DNA-binding activity and possibly affecting its nuclear translocation activity. To confirm our Rank 1 and Rank 3 docking models, two RelA mutants affecting the interaction with cardamonin were tested for their activities. The result showing that RelA mutants barely underwent nuclear translocation in response to the TNF-α stimulation did not directly confirm the RelA-binding sites of cardamonin. However, the predicted cardamonin-binding sites in the Rank 1 and Rank 3 models appeared to be essential for nuclear translocation activity. This result also suggests that cardamonin interfered with this process when it was bound to these sites.

## 4. Materials and Methods

### 4.1. Cells

A549 cells (JCRB0076; human lung adenocarcinoma), MCF-7 cells (JCRB0134; human breast cancer), and HT-1080 cells (JCRB9113; human fibrosarcoma) were obtained from the National Institutes of Biomedical Innovation, Health and Nutrition JCRB Cell Bank (Osaka, Japan). 293T cells (RCB2202; human embryonic kidney) were obtained from the RIKEN BioResource Research Center Cell Bank (Tsukuba, Japan).

### 4.2. Reagents

Cardamonin (Cayman Chemical, Ann Arbor, MI, USA), 4′-hydroxychalcone (Fujifilm Wako Pure Chemical Corporation, Osaka, Japan), isoliquiritigenin (Tokyo Chemical Industry Co., Ltd., Tokyo, Japan), and xanthohumol (Angene International Limited, London, UK) were obtained commercially. Recombinant human TNF-α (Dainippon Pharmaceutical Co., Ltd., Osaka, Japan) was kindly provided.

### 4.3. Antibodies

The target proteins and sources of the primary antibodies used are listed below: ICAM-1 (15.2; Leinco Technologies, Inc., St. Louis, MO, USA), IκBα (25/IkBa/MAD-3; BD Biosciences, Danvers, MA, USA), RelA (F-6; Santa Cruz Biotechnology, Dallas, TX, USA), DYKDDDDK (FLAG) (1E6; Fujifilm Wako Pure Chemical Corporation, Osaka, Japan), β-actin (AC-15; Sigma-Aldrich, St. Louis, MO, USA), glyceraldehyde-3-phosphate dehydrogenase (6C5; Santa Cruz Biotechnology, Dallas, TX, USA), and lamin A/C (E-1; Santa Cruz Biotechnology, Dallas, TX, USA). A peroxidase-conjugated goat anti-mouse IgG (H+L) antibody (Jackson ImmunoResearch Laboratories, West Grove, PA, USA) was used as the secondary antibody.

### 4.4. Plasmids

A pGL4.22 [luc2CP/Puro] reporter vector carrying the ICAM-1 promoter (−1604 to +40) [77], a luciferase reporter vector encoding two copies of the κB sequence from the Igκ enhancer (a kind gift from Ralph C. Budd) [78], and the pCR3 expression vector encoding cytomegalovirus promoter-driven *Renilla* luciferase [45,79] were previously described. The lentivirus vectors CSII-CMV-MCS-IRES2-Bsd (RDB04385), pCMV-VSV-G-RSV-Rev (RDB04393), and pCAG-HIVgp (RDB04394) were kindly provided by Hiroyuki Miyoshi (RIKEN BioResource Center, Tsukuba, Japan). N-terminal FLAG-tagged human RelA WT cDNA and human RelA C38S cDNA [54] were inserted into CSII-CMV-MCS-IRES2-Bsd lentivirus vectors. Human RelA possessing the R30A/N155A/T191A and Y36A/K37A/K122A mutations were constructed and inserted into pCR3 expression vectors and CSII-CMV-MCS-IRES2-Bsd lentivirus vectors. Lentivirus particles were prepared by 293T cells transfected with pCMV-VSV-G-RSV-Rev, pCAG-HIVgp, and CSII-CMV-MCS-IRES2-Bsd encoding human RelA mutants.

### 4.5. Cell Culture

A549, MCF-7, and HT-1080 cells were subcultured with RPMI 1640 medium (Thermo Fisher Scientific, Grand Island, NY, USA) supplemented with heat-inactivated fetal calf serum (Sigma-Aldrich, St. Louis, MO, USA) and a penicillin-streptomycin mixed solution (Nacalai Tesque, Kyoto, Japan) in a CO_2_ incubator at 37 °C with 5% CO_2_. For MCF-7 cells, human recombinant insulin (10 µg/mL; Fujifilm Wako Pure Chemical Corporation, Osaka, Japan) was included in the culture medium. The day before experiments, cells were seeded on plates or dishes. The cells were preincubated with compounds for 1 h and then incubated with or without TNF-α (2.5 ng/mL) in the presence or absence of the compounds for the indicated times. A549 cells were infected with lentivirus particles and cultured in the presence of blasticidin to establish stable transfectants.

### 4.6. Cell Viability Assay

Cells were stained with 0.2% crystal violet in methanol for 15 min and washed extensively with water. After adding methanol, an iMark^TM^ microplate reader (Bio-Rad Laboratories, Hercules, CA, USA) was used to measure absorbance at 570 nm. Each experiment consisted of three biological replicates.

### 4.7. Flow Cytometry

An isotype control antibody (MOPC-1; BioLegend, San Diego, CA, USA) or mouse anti-ICAM-1 antibody (15.2) was used to stain cells, followed by a phycoerythrin-labeled anti-mouse IgG antibody (Jackson ImmunoResearch Laboratories, West Grove, PA, USA) as previously described [50]. FACSCalibur (BD Biosciences, Danvers, MA, USA) was used to measure fluorescent intensity. Further analyses were conducted using FlowJo software version 8.5.1 (Tomy Digital Biology, Tokyo, Japan). Each experiment consisted of three technical replicates.

### 4.8. Quantitative PCR

Total RNA was prepared using Sepasol^®^-RNA I Super G (Nacalai Tesque, Kyoto, Japan) and was then used for cDNA synthesis with ReverTra Ace^®^ (Toyobo, Osaka, Japan) and oligo (dT)20 (Thermo Fisher Scientific, Waltham, MA, USA), as previously described [46]. Real-time PCR was conducted using Thermal Cycler Dice^®^ Real Time System Lite (Takara Bio, Kusatsu, Japan) according to our previous study [46], using the primers for ICAM-1 [80] and β-actin mRNA [81]. The amount of ICAM-1 mRNA was normalized with that of β-actin mRNA.

### 4.9. Luciferase Reporter Assay

Cells were transfected with luciferase reporter vectors. HilyMax Transfection Reagent was obtained from Dojindo Laboratories (Kumamoto, Japan). Cell lysates were prepared and measured for relative light units using a Lumitester C-110 (Kikkoman Biochemifa, Tokyo, Japan), as previously described [45]. Each experiment consisted of three biological replicates.

### 4.10. Western Blotting

The methods of cell lysate preparation and Western blotting were previously described [45,46]. In brief, to prepare the nuclear and cytoplasmic fractions, cells were rinsed with phosphate-buffered saline, lysed with Triton X-100 lysis buffer, and then centrifuged to separate supernatants, which were collected as the cytoplasmic fraction, from the precipitates. The precipitates were rinsed with the Triton X-100 lysis buffer and then treated with sonication and centrifugation to prepare the nuclear fraction. Protein bands were visualized by primary antibodies specific for target proteins and secondary antibodies conjugated with peroxidases, followed by chemiluminescence reactions, which were detected by Amersham Imager 680 (GE Healthcare, Tokyo, Japan). Band intensities were measured using version 7.0 of the ImageQuant TL software toolbox (GE Healthcare Japan, Tokyo, Japan). The blots were treated with Stripping Solution (Fujifilm Wako Pure Chemical Corporation, Osaka, Japan) and then reprobed with antibodies for loading controls.

### 4.11. ChIP Assay

Cell fixation and the ChIP assay were performed as previously described [79]. Immunoprecipitation was conducted using an anti-RelA antibody (F-6). The amounts of immunoprecipitated and input DNA were measured by real-time PCR using primers that amplified the ICAM-1 promoter (−286 to −90) [82].

### 4.12. Cell ELISA

Cells were fixed with paraformaldehyde, blocked with bovine serum albumin, and treated with an anti-ICAM-1 antibody and peroxidase-conjugated secondary antibody, as previously described [45,46]. Hydrogen peroxide and *o*-phenylenediamine hydrochloride were used as substrates of the colorimetric reaction, followed by an evaluation of absorbance at 450 nm by an iMark^TM^ microplate reader. Each experiment consisted of three biological replicates.

### 4.13. Statistical Analysis

To evaluate the significance of differences, each experiment was repeated independently at least three times. A one-way analysis of variance (ANOVA) followed by Tukey’s post hoc test was performed using KaleidaGraph software version 4.5.1 (Hulinks, Tokyo, Japan).

### 4.14. In Silico Molecular Docking Analysis

AutoDock vina version 1.1.2 was used to evaluate the semiflexible docking of complexes composed of a flexible ligand and rigid protein [83,84]. The complex of the human RelA protein (PDB ID: 1NFI, chain A) and cardamonin as a ligand was assessed. The whole RelA protein was covered, the center coordinate (x, y, z) was (−3, 49, 11), and the box size was 80 Å. The top nine models of RelA and cardamonin complexes were identified with scores ≥ −5.8 kcal/mol. The crystal structure of the murine RelA and p50 heterodimer complexed with DNA (PDB ID: 1VKX) was also used.

## 5. Conclusions

Cardamonin has been reported to inhibit various signaling pathways, including the NF-κB signaling pathway [30,31,32,33]. Based on its broader inhibitory effects, cardamonin is assumed to directly interfere with multiple intracellular targets. Cardamonin suppressed the nuclear translocation of RelA and its DNA binding in the TNF-α-induced NF-κB signaling pathway in A549 cells. Cardamonin also inhibited the nuclear translocation of the RelA C38S mutant, indicating that the alkylation of C38 is not a primary target site of RelA, in contrast to many compounds undergoing the Michael addition reaction. However, *in silico* molecular docking results showed that cardamonin interacted with RelA at a distinct region that underwent a marked structural change when it formed a complex with DNA. Mutations of the predicted cardamonin-binding sites of RelA abrogated its nuclear translocation activity. As a limitation of this study, we mainly conducted *in vitro* cell cultures, and thus the results obtained need to be confirmed *in vivo* in follow-up studies. In conclusion, we herein demonstrated that cardamonin interfered with RelA nuclear translocation and DNA binding. We also hypothesized that cardamonin directly bound to RelA at a specific site near the DNA-binding region, thereby inhibiting structural changes. The present study provides novel insights into the molecular mechanisms by which cardamonin binds to cellular target proteins and inhibits their biological activities. Herbal and medicinal plants may provide useful ingredients for the development of anti-inflammatory and anticancer agents with fewer side effects. In the future, a more detailed understanding of the interactome of cardamonin and its derivatives with their cellular target proteins will contribute to the development of novel strategies that manipulate the NF-κB signaling pathway for preventive and therapeutic agents.

## Data Availability

Data will be made available upon reasonable request.

## Supplementary Materials

The following supporting information may be downloaded at: https://www.mdpi.com/article/10.3390/molecules30224324/s1, Figure S1: Effects of DMSO on cell viability and ICAM-1 expression in A549 cells; Figure S2: Original blots in Figure 4B; Figure S3: Original blots (1) in Figure 4C; Figure S4: Original blots (2) in Figure 4C; Figure S5: Original blots (3) in Figure 4C; Figure S6: Original blots in Figure 5A (nucleus); Figure S7: Original blots in Figure 5A (cytoplasm); Figure S8: Original blots (1) in Figure 5B; Figure S9: Original blots (2) in Figure 5B; Figure S10: Original blots (3) in Figure 5B; Figure S11: Original blots (4) in Figure 5B; Figure S12: Original blots (1) in Figure 5C; Figure S13: Original blots (2) in Figure 5C; Figure S14: Original blots (3) in Figure 5C; Figure S15: Original blots (4) in Figure 5C; Figure S16: Original blots in Figure 6C; Figure S17: Original blots (1) in Figure 6D; Figure S18: Original blots (2) in Figure 6D; Figure S19: Original blots (3) in Figure 6D; Figure S20: Original blots in Figure 6E; Figure S21: Original blots (1) in Figure 6F; Figure S22: Original blots (2) in Figure 6F; Figure S23: Original blots (3) in Figure 6F; Figure S24: Original blots in Figure 7A (nucleus); Figure S25: Original blots in Figure 7A (cytoplasm); Figure S26: Original blots (1) in Figure 7B; Figure S27: Original blots (2) in Figure 7B; Figure S28: Original blots (3) in Figure 7B; Figure S29: Original blots (1) in Figure 7C; Figure S30: Original blots (2) in Figure 7C; Figure S31: Original blots (3) in Figure 7C; Figure S32: Original blots in Figure 7D (nucleus); Figure S33: Original blots in Figure 7D (cytoplasm); Figure S34: Original blots (1) in Figure 7E; Figure S35: Original blots (2) in Figure 7E; Figure S36: Original blots (3) in Figure 7E; Figure S37: Original blots (1) in Figure 7F; Figure S38: Original blots (2) in Figure 7F; Figure S39: Original blots (3) in Figure 7F; Figure S40: Original blots in Figure 10A; Figure S41: Original blots (1) in Figure 10B; Figure S42: Original blots (2) in Figure 10B; Figure S43: Original blots (3) in Figure 10B; Figure S44: Original blots in Figure 11A (nucleus); Figure S45: Original blots in Figure 11A (cytoplasm); Figure S46: Original blots (1) in Figure 11B; Figure S47: Original blots (2) in Figure 11B; Figure S48: Original blots (3) in Figure 11B; Figure S49: Original blots (1) in Figure 11C; Figure S50: Original blots (2) in Figure 11C; Figure S51: Original blots (3) in Figure 11C; Figure S52: Original blots in Figure 11D (nucleus); Figure S53: Original blots in Figure 11D (cytoplasm); Figure S54: Original blots (1) in Figure 11E; Figure S55: Original blots (2) in Figure 11E; Figure S56: Original blots (3) in Figure 11E; Figure S57: Original blots (1) in Figure 11F; Figure S58: Original blots (2) in Figure 11F; Figure S59: Original blots (3) in Figure 11F; Figure S60: Original blots in Figure 11G (nucleus); Figure S61: Original blots in Figure 11G (cytoplasm); Figure S62: Original blots (1) in Figure 11H; Figure S63: Original blots (2) in Figure 11H; Figure S64: Original blots (3) in Figure 11H; Figure S65: Original blots (1) in Figure 11I; Figure S66: Original blots (2) in Figure 11I; Figure S67: Original blots (3) in Figure 11I. Figure S68: Original blots in Figure 12A; Figure S69: Original blots in Figure 12B (nucleus); Figure S70: Original blots in Figure 12B (cytoplasm); Figure S71: Original blots (1) in Figure 12C; Figure S72: Original blots (2) in Figure 12C; Figure S73: Original blots (3) in Figure 12C; Figure S74: Original blots (1) in Figure 12D; Figure S75: Original blots (2) in Figure 12D; Figure S76: Original blots (3) in Figure 12D; Figure S77: Original blots in Figure 12E (nucleus); Figure S78: Original blots in Figure 12E (cytoplasm); Figure S79: Original blots (1) in Figure 12F; Figure S80: Original blots (2) in Figure 12F; Figure S81: Original blots (3) in Figure 12F; Figure S82: Original blots (1) in Figure 12G; Figure S83: Original blots (2) in Figure 12G; Figure S84: Original blots (3) in Figure 12G; Figure S85: Original bots in Figure 14A; Figure S86: Original blots in Figure 14B (nucleus); Figure S87: Original blots in Figure 14B (cytoplasm); Figure S88: Original blots (1) in Figure 14C; Figure S89: Original blots (2) Figure 14C; Figure S90: Original blots (3) in Figure 14C; Figure S91: Original blots (1) in Figure 14D; Figure S92: Original blots (2) Figure 14D; Figure S93: Original blots (3) in Figure 14D; Figure S94: Original blots in Figure 14E (nucleus); Figure S95: Original blots in Figure 14E (cytoplasm); Figure S96: Original blots (1) in Figure 14F; Figure S97: Original blots (2) Figure 14F; Figure S98: Original blots (3) in Figure 14F; Figure S99: Original blots (1) in Figure 14G; Figure S100: Original blots (2) Figure 14G; Figure S101: Original blots (3) in Figure 14G; Figure S102: Original blots in Figure 14H (nucleus); Figure S103: Original blots in Figure 14H (cytoplasm); Figure S104: Original blots (1) in Figure 14I; Figure S105: Original blots (2) Figure 14I; Figure S106: Original blots (3) in Figure 14I; Figure S107: Original blots (1) in Figure 14J; Figure S108: Original blots (2) Figure 14J; Figure S109: Original blots (3) in Figure 14J.

## Abbreviations

The following abbreviations are used in this manuscript:

ICAM-1	Intercellular adhesion molecule 1
TNF	Tumor necrosis factor
NF-κB	Nuclear factor κB
IκB	Inhibitor of nuclear factor κB
RIPK1	Receptor-interacting protein kinase 1
TRADD	TNF receptor-associated death domain protein
TRAF	TNF receptor-associated factor
LPS	Lipopolysaccharide
ELISA	Enzyme-linked immunosorbent assay
PCR	Polymerase chain reaction
ChIP	Chromatin immunoprecipitation
SRC2	Santonin-related compound 2
WT	Wild type
TLR	Toll-like receptor
MD-2	Myeloid differentiation factor 2
IFN-γ	Interferon-γ
mTOR	Mammalian target of rapamycin
