# Supplementary material for: Cardamonin Inhibits the Nuclear Translocation and DNA Binding of RelA in the Tumor Necrosis Factor-α-Induced NF-κB Signaling Pathway in Human Lung Adenocarcinoma A549 Cells
Source: Molecules. 2025 Nov 7;30(22):4324. doi: 10.3390/molecules30224324 (PMC12655324; doi:10.3390/molecules30224324)
Supplement: Supplementary file 1 [file molecules-30-04324-s001.zip › Supplementary Raw Data_20251111(revised).pdf]

Figure S1: Effects of DMSO on cell viability and ICAM-1 expression in A549 cells.

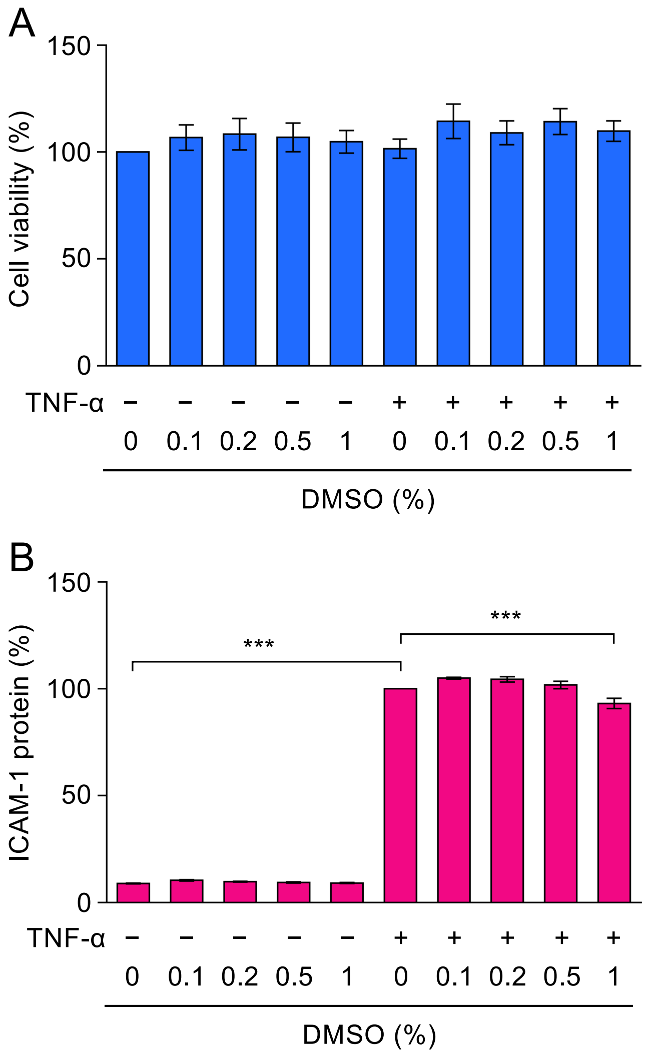

Figure S1. Effects of DMSO on cell viability and ICAM-1 expression in A549 cells. (A and B) A549 cells were treated with different concentrations of DMSO for 1 h, followed by a 6-h stimulation with (+) or without (−) TNF-α (2.5 ng/mL) in the presence or absence of DMSO at the indicated final concentrations. Cell viability (%) is presented as the mean ± standard error (n = 3) (A). No significant differences were observed (A). ICAM-1 expression (%) is presented as the mean ± standard error (n = 3) (B). ***$P < 0.001$.

Figure S2: Original blots in Figure 4B

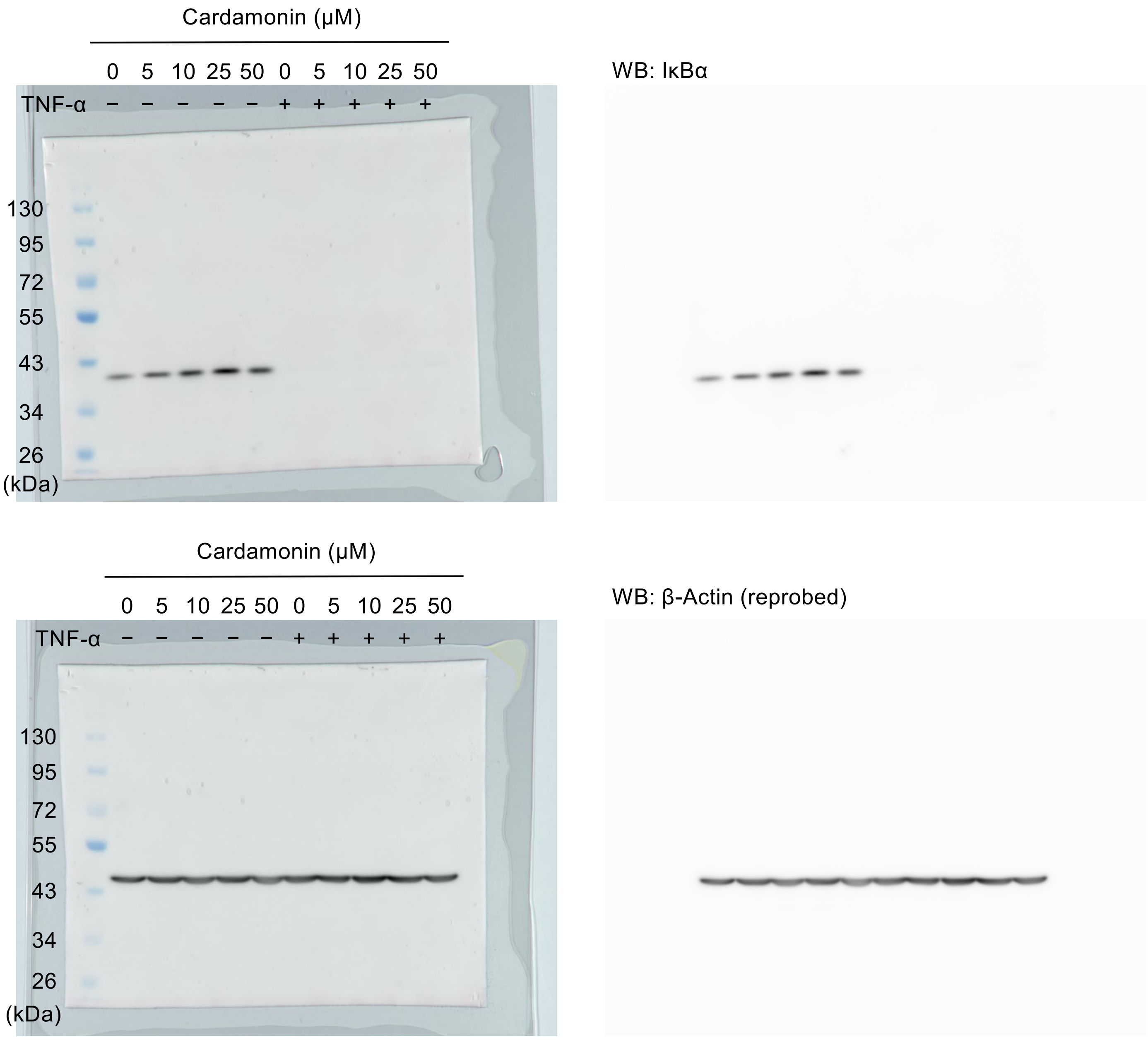

Figure S3: Original blots (1) in Figure 4C

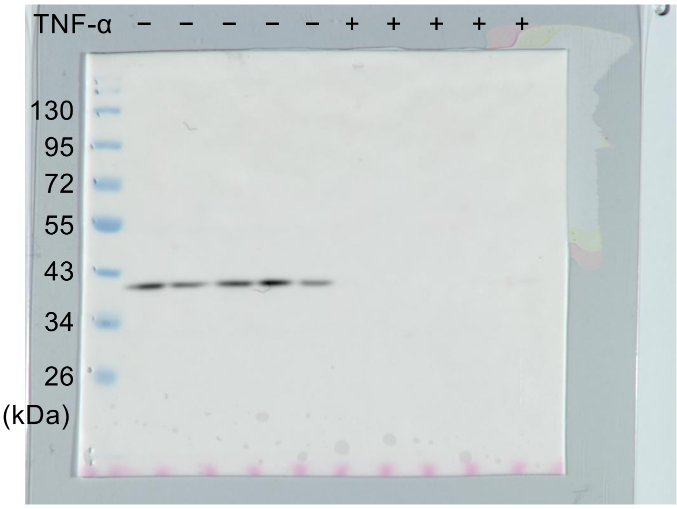

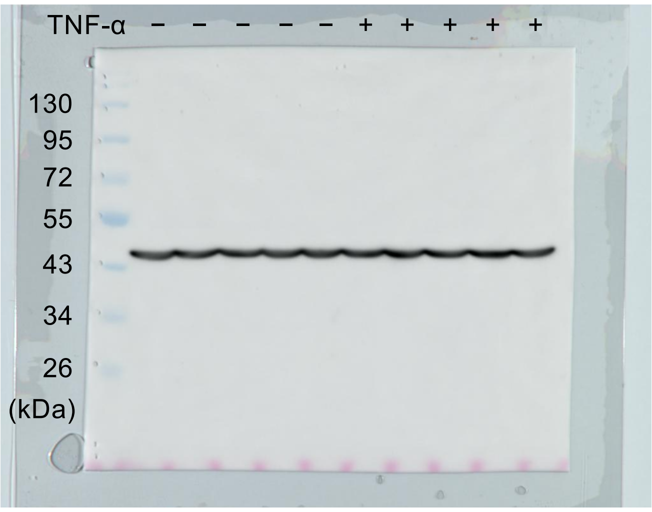

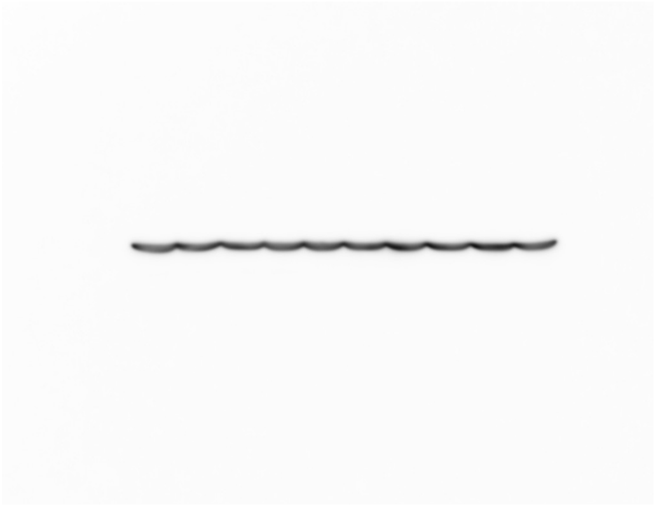

Figure S4: Original blots (2) in Figure 4C

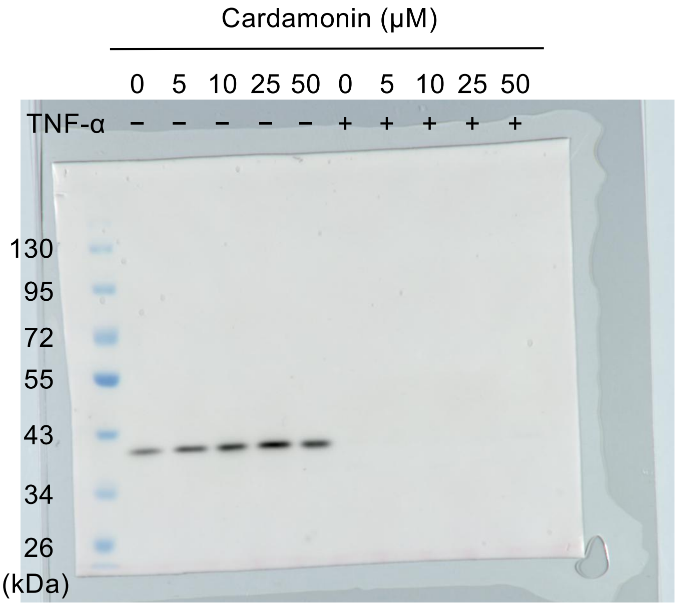

WB: IκBα

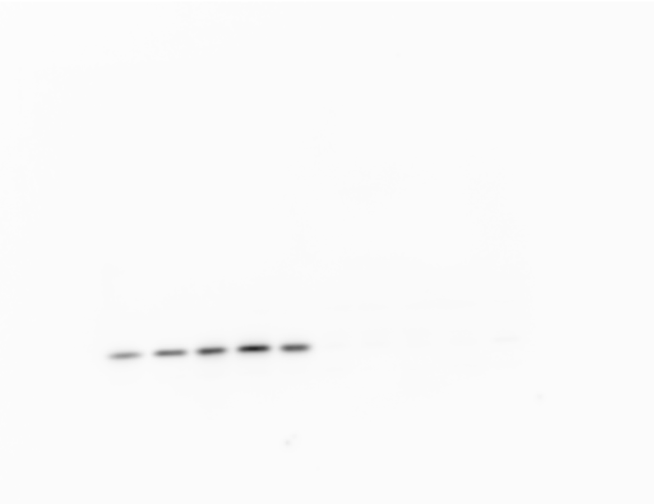

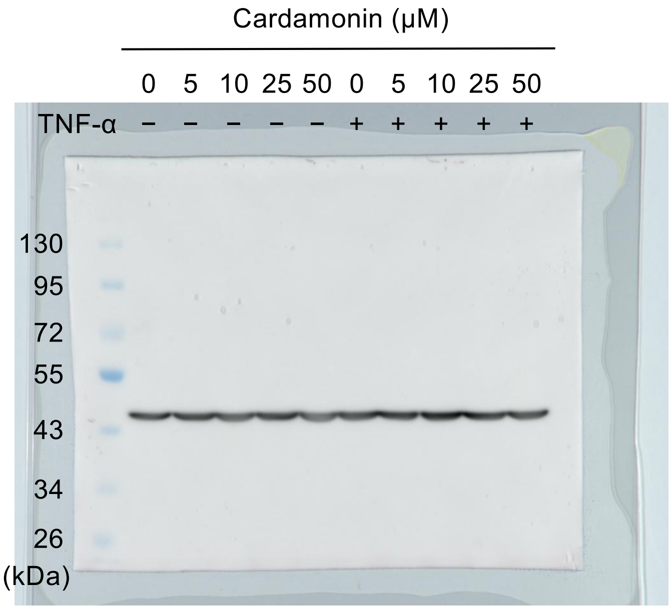

WB: β-Actin (reprobed)

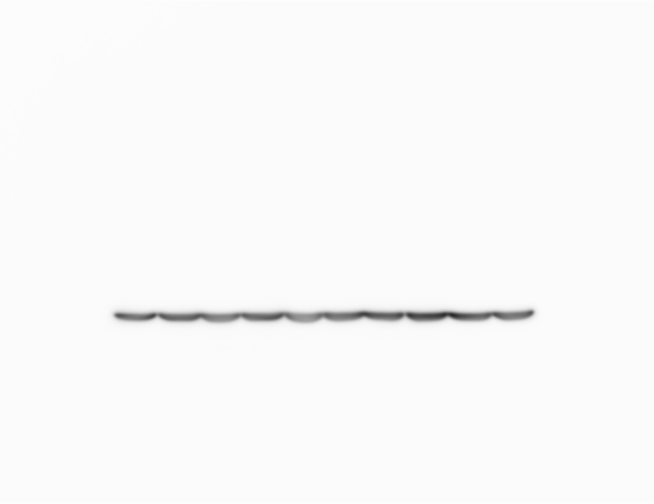

Figure S5: Original blots (3) in Figure 4C

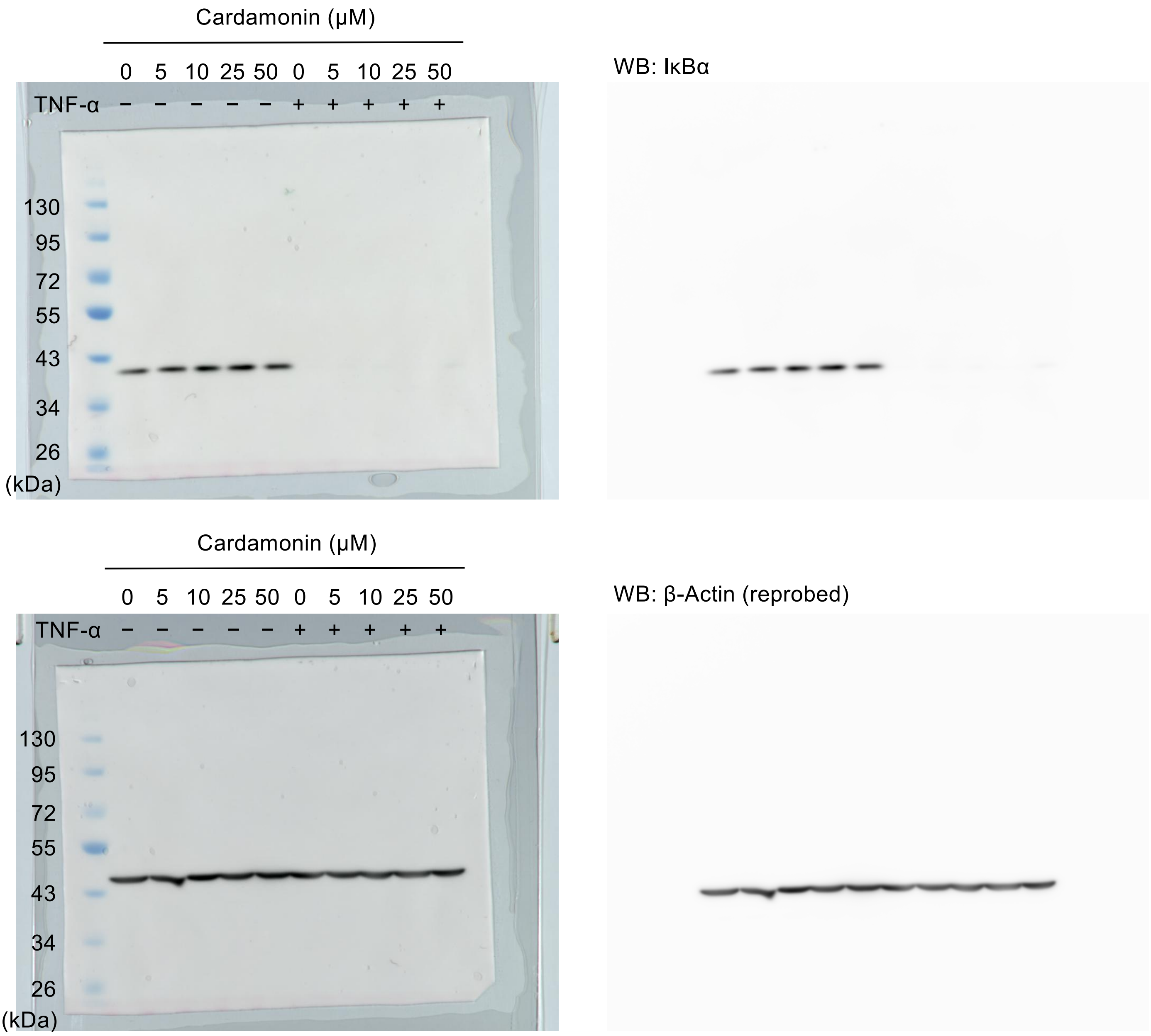

Figure S6: Original blots in Figure 5A (nucleus)

Cardamonin (μM)

0 0 5 10 25 50

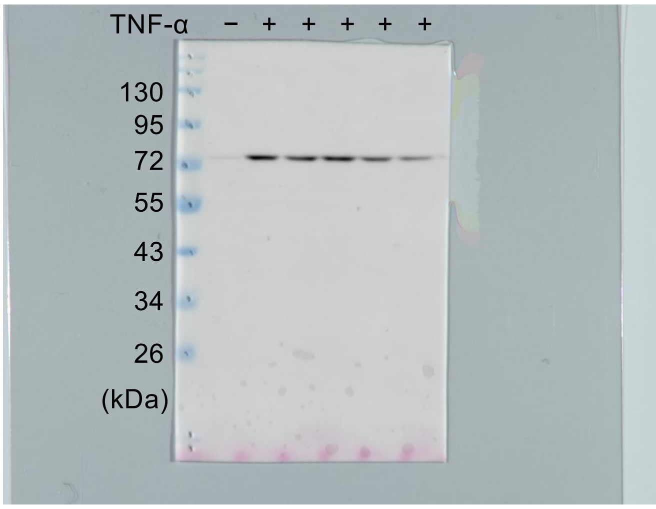

WB: RelA

Cardamonin (μM)

0 0 5 10 25 50

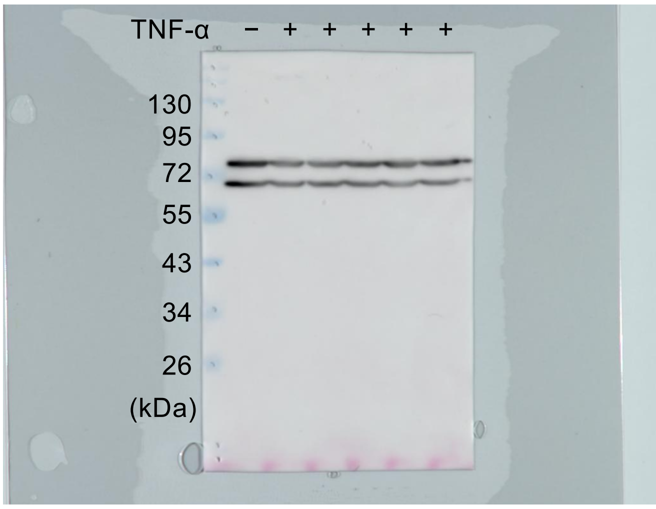

WB: Lamin A/C (reprobed)

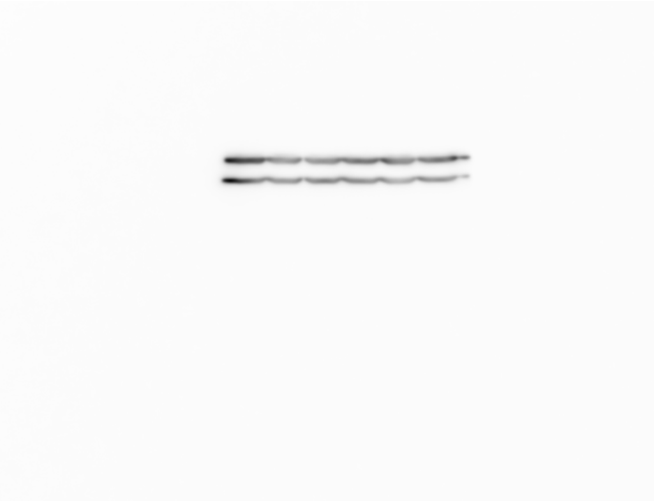

Figure S7: Original blots in Figure 5A (cytoplasm)

Cardamonin (μM)

0 0 5 10 25 50

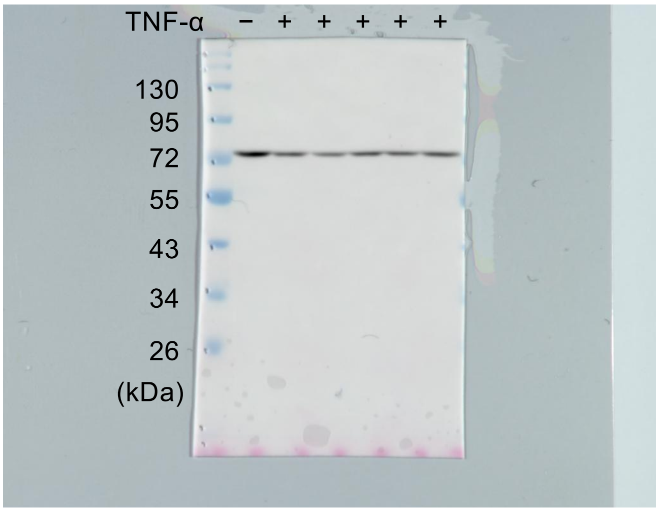

WB: RelA

Cardamonin (μM)

0 0 5 10 25 50

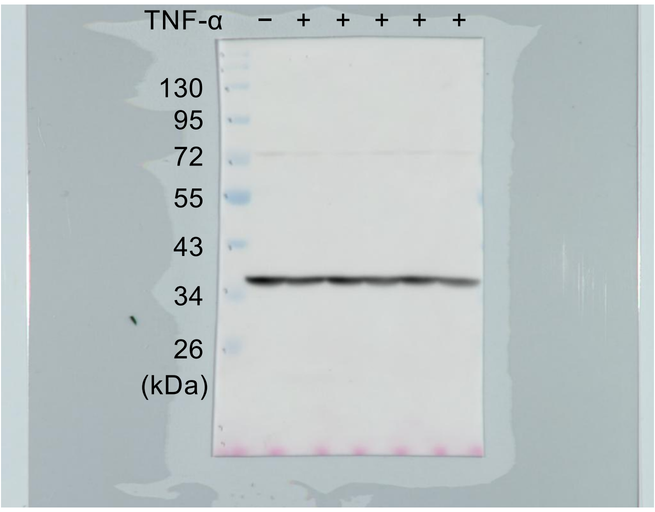

WB: GAPDH (reprobed)

Figure S8: Original blots (1) in Figure 5B

Cardamonin (μM)

0 0 5 10 25 50

TNF-α − + + + + +

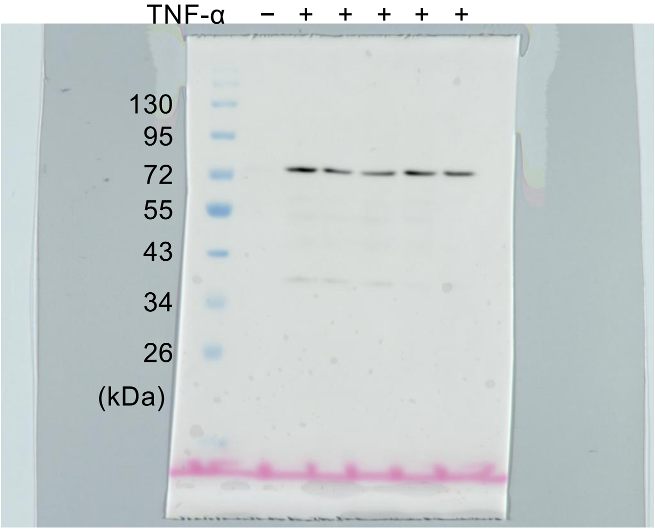

WB: RelA

Cardamonin (μM)

0 0 5 10 25 50

TNF-α − + + + + +

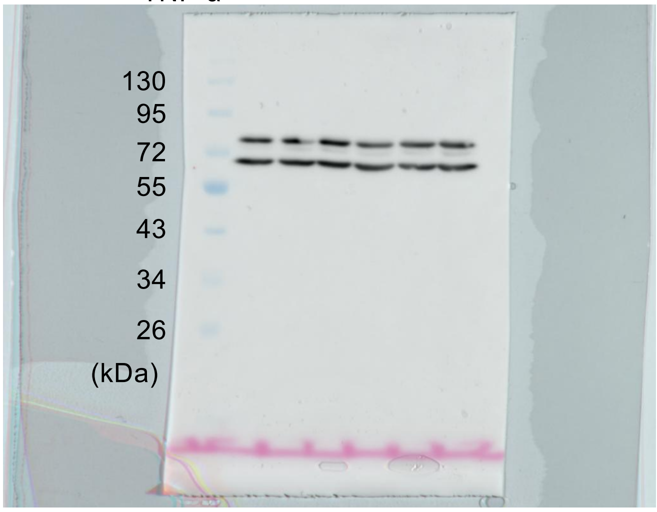

WB: Lamin A/C (reprobed)

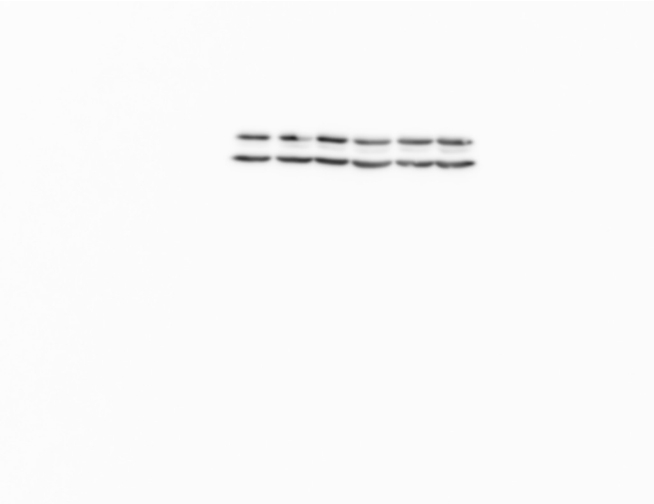

Figure S9: Original blots (2) in Figure 5B

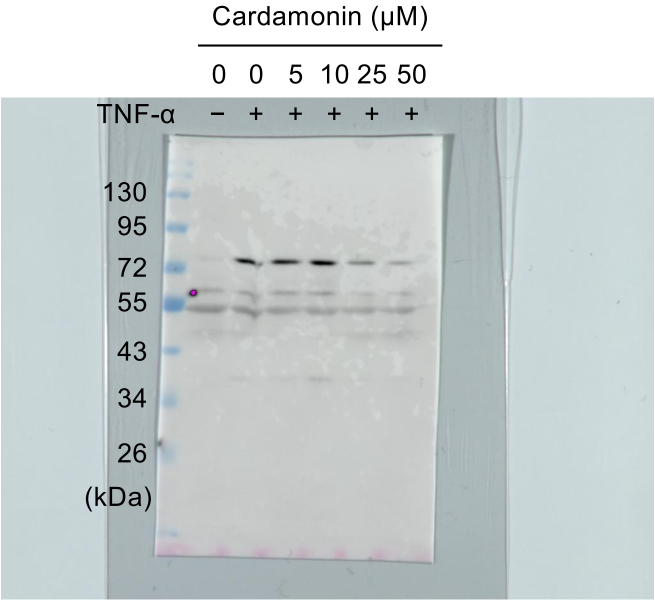

WB: RelA

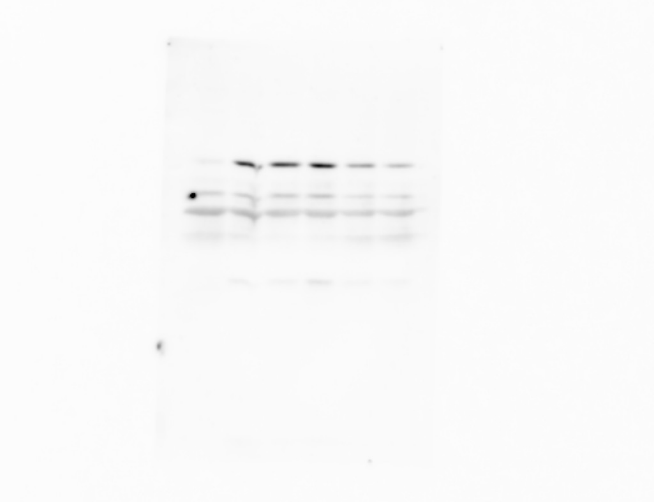

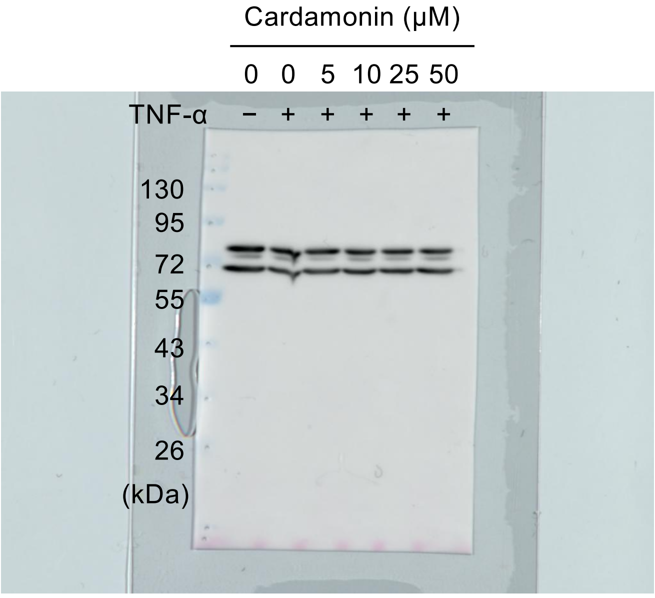

WB: Lamin A/C (reprobed)

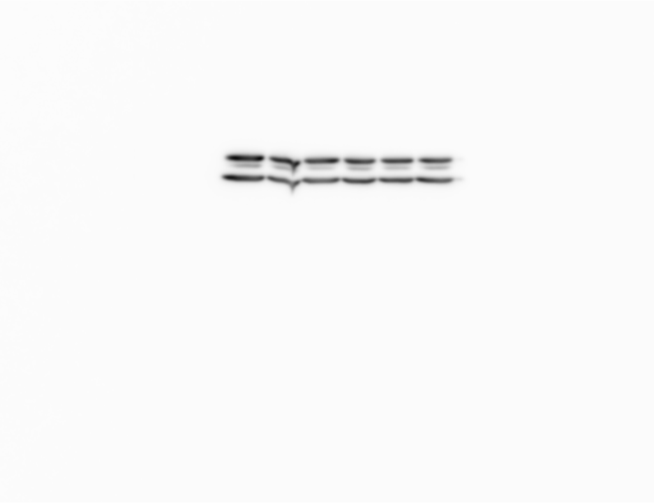

Figure S10: Original blots (3) in Figure 5B

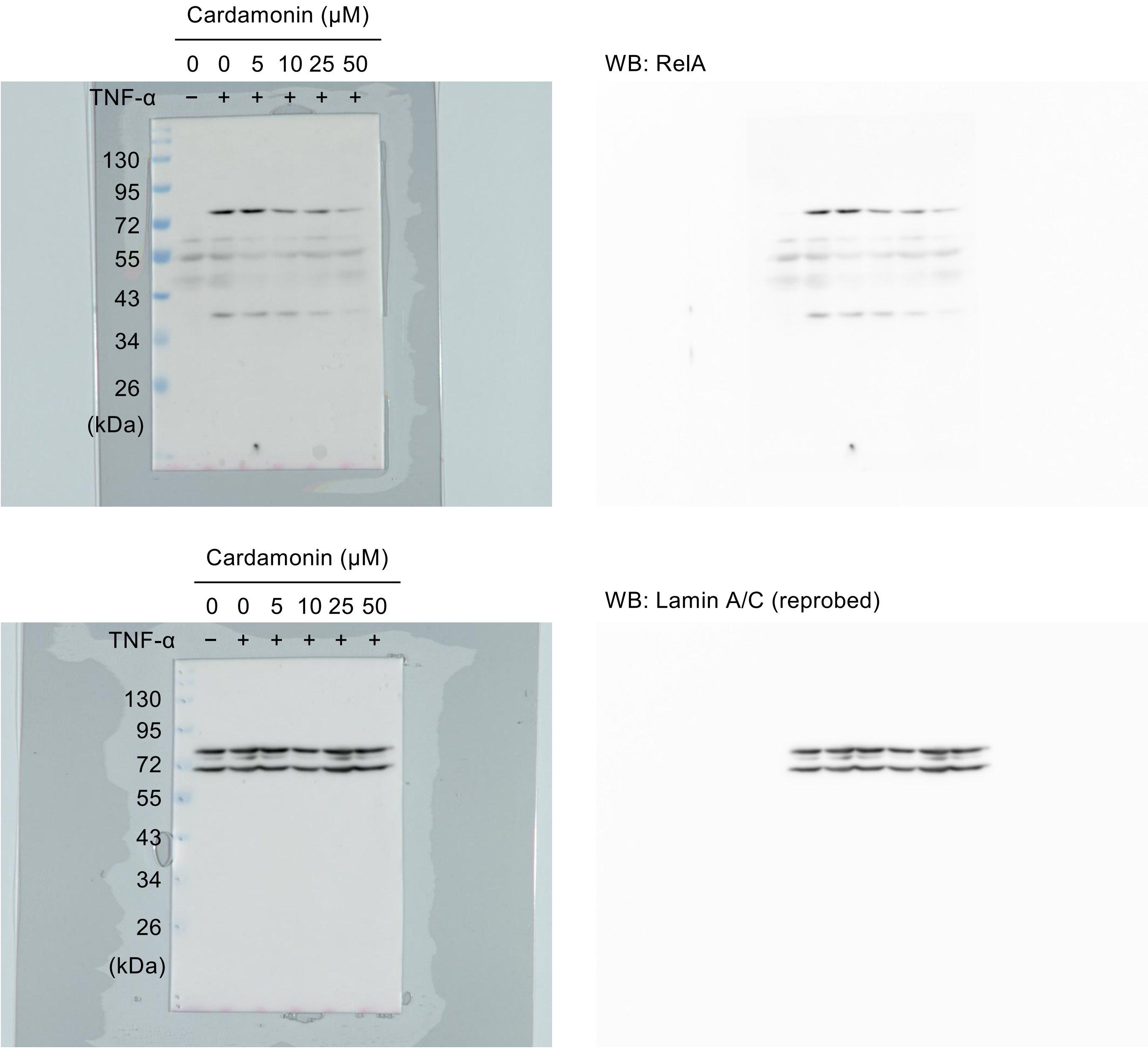

Figure S11: Original blots (4) in Figure 5B

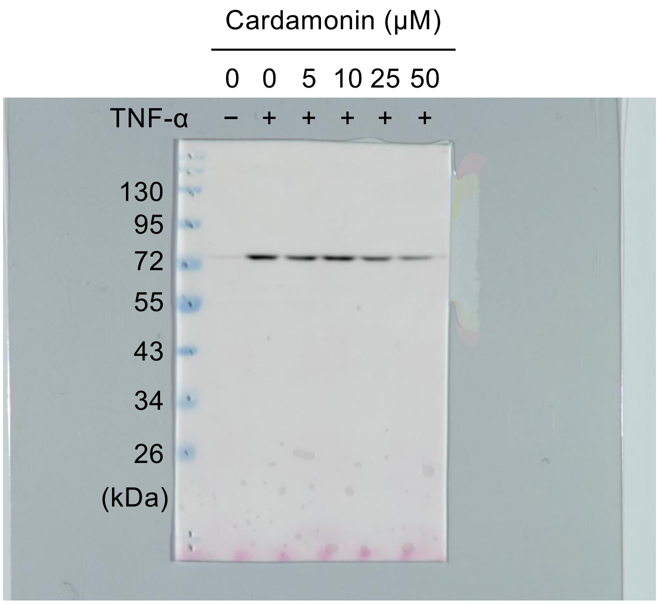

WB: RelA

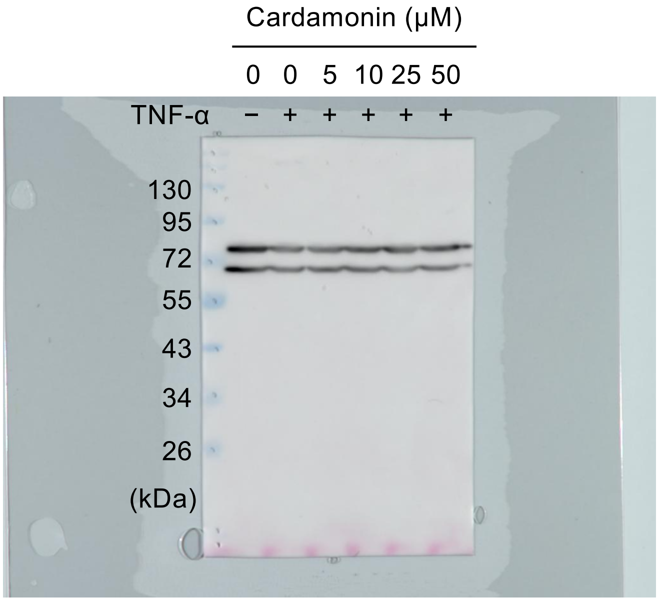

WB: Lamin A/C (reprobed)

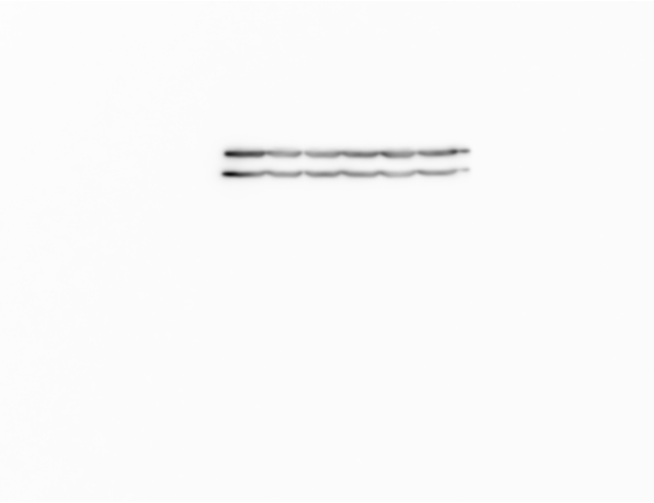

Figure S12: Original blots (1) in Figure 5C

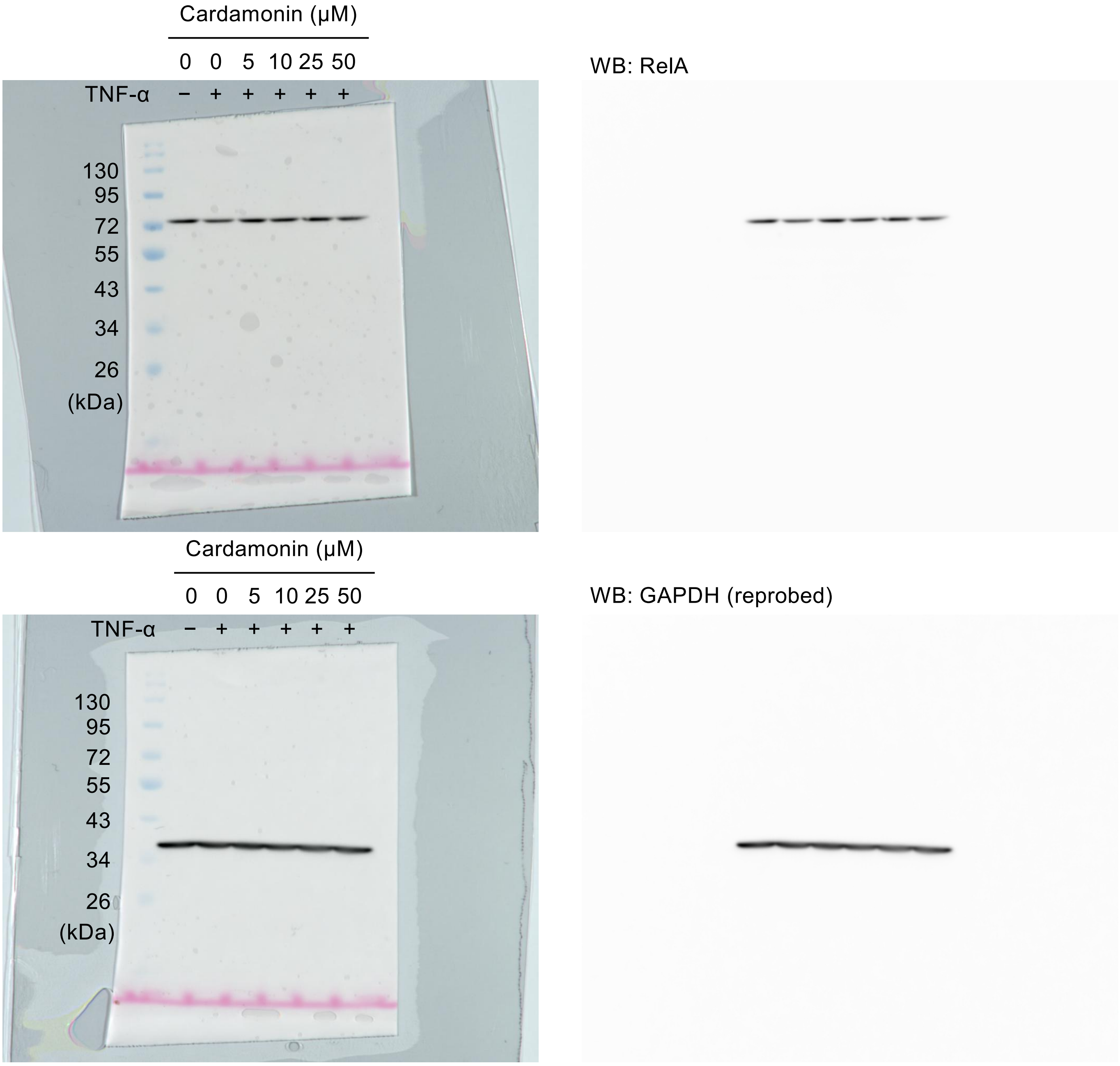

Figure S13: Original blots (2) in Figure 5C

Cardamonin (μM)

0 0 5 10 25 50

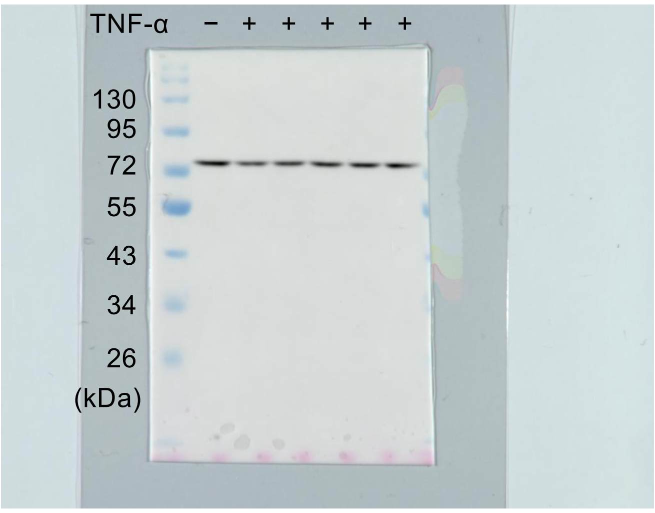

WB: RelA

Cardamonin (μM)

0 0 5 10 25 50

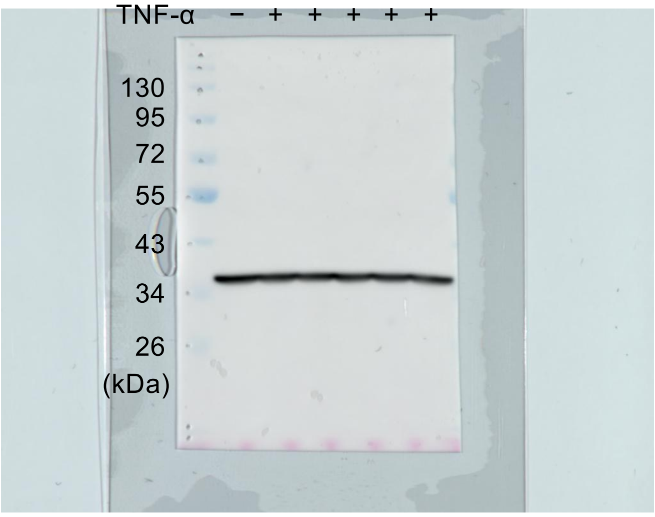

WB: GAPDH (reprobed)

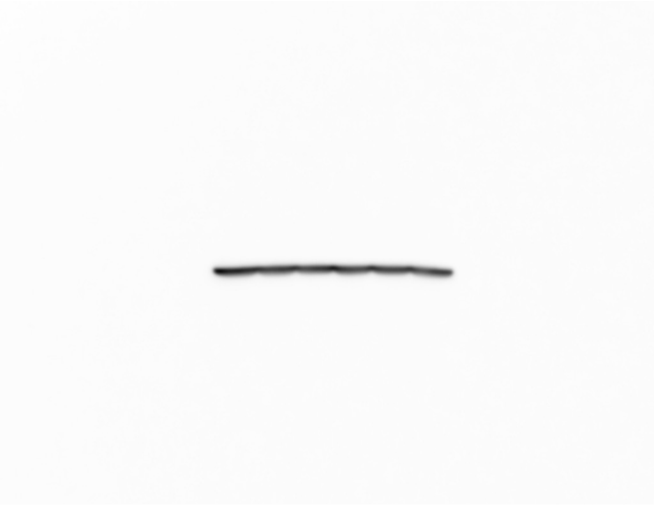

Figure S14: Original blots (3) in Figure 5C

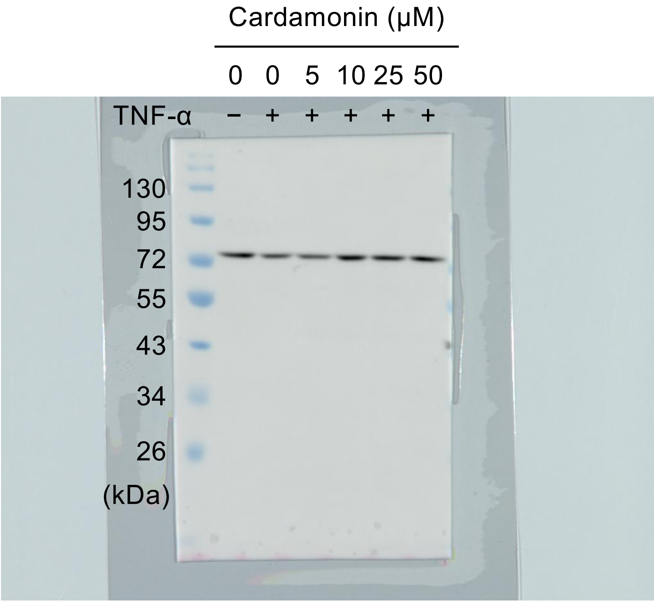

WB: RelA

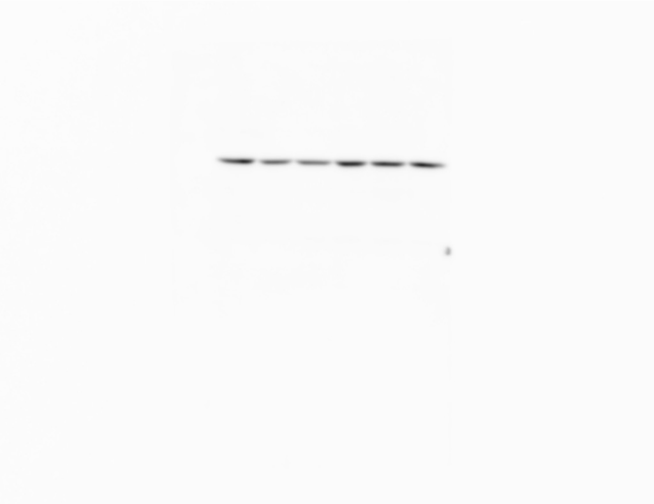

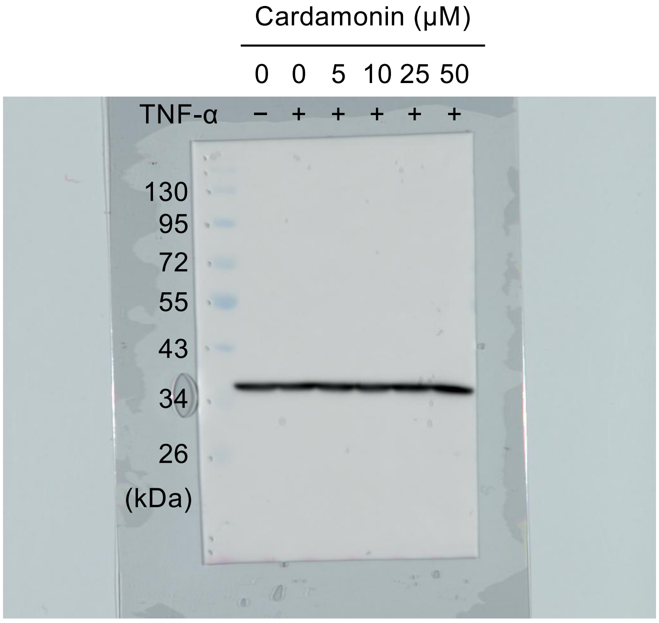

WB: GAPDH (reprobed)

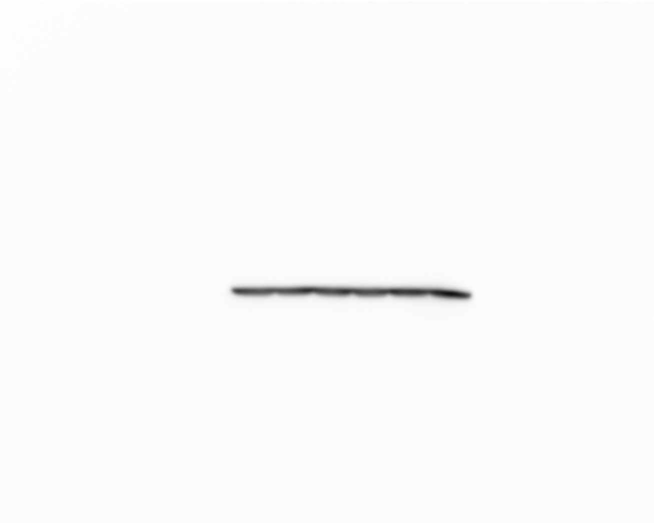

Figure S15: Original blots (4) in Figure 5C

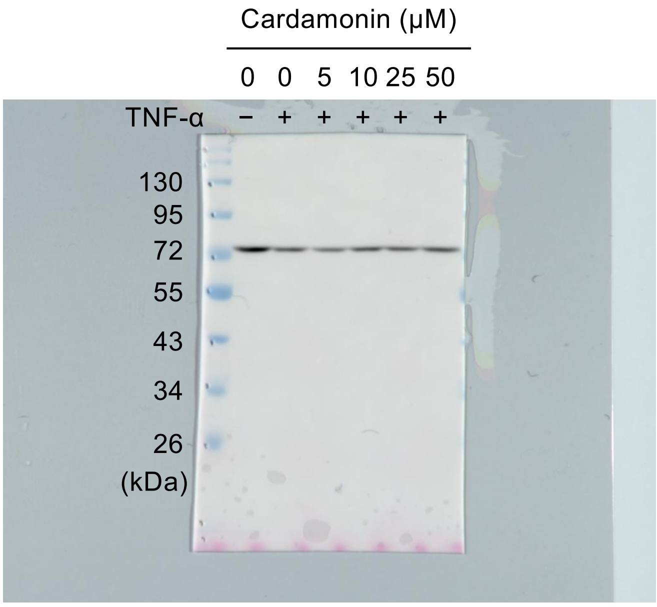

WB: RelA

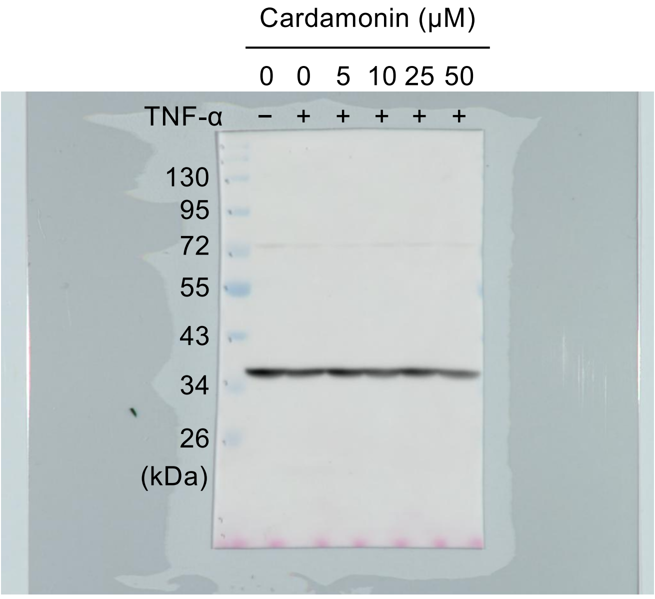

WB: GAPDH (reprobed)

Figure S16: Original blots in Figure 6C

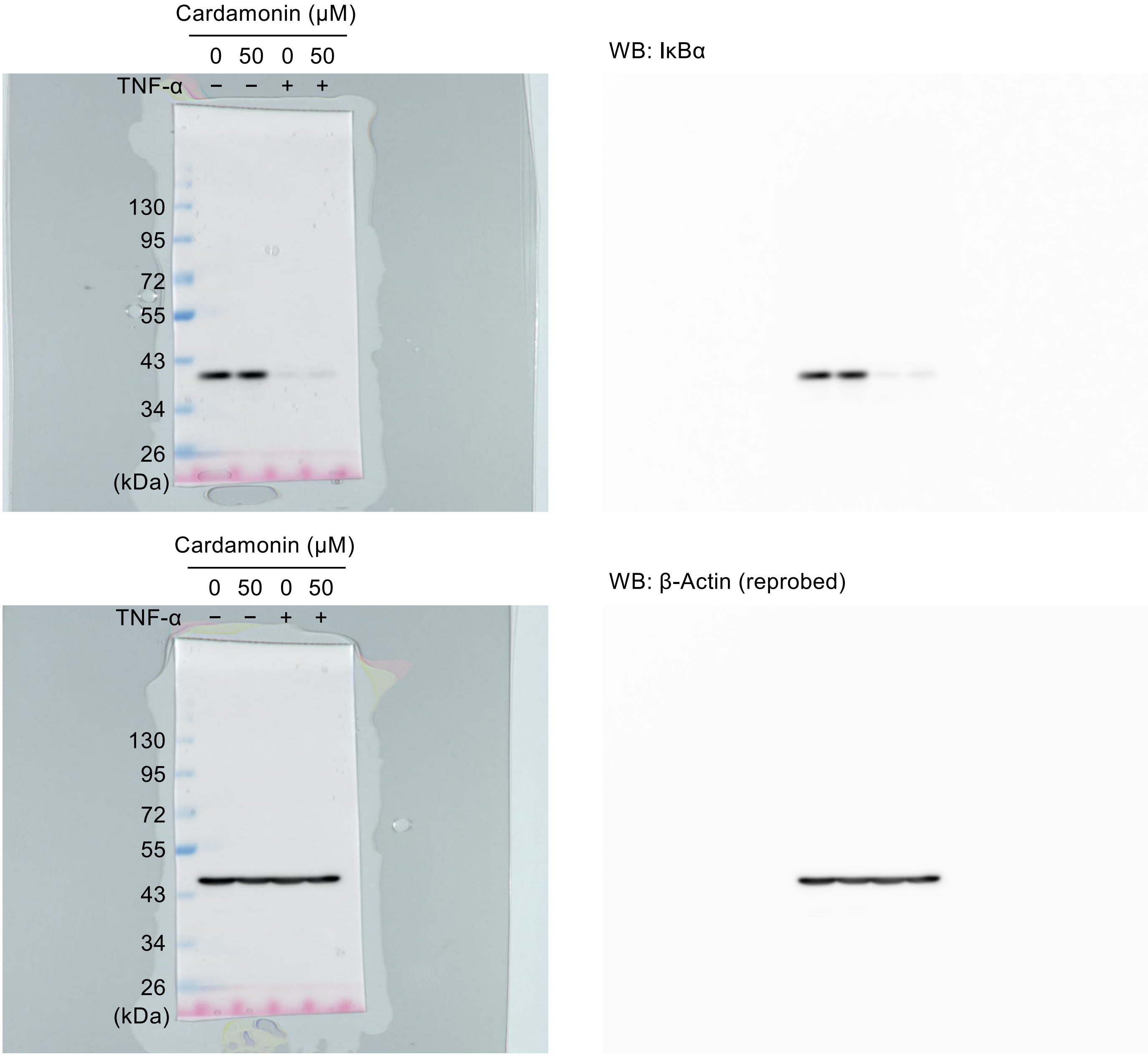

Figure S17: Original blots (1) in Figure 6D

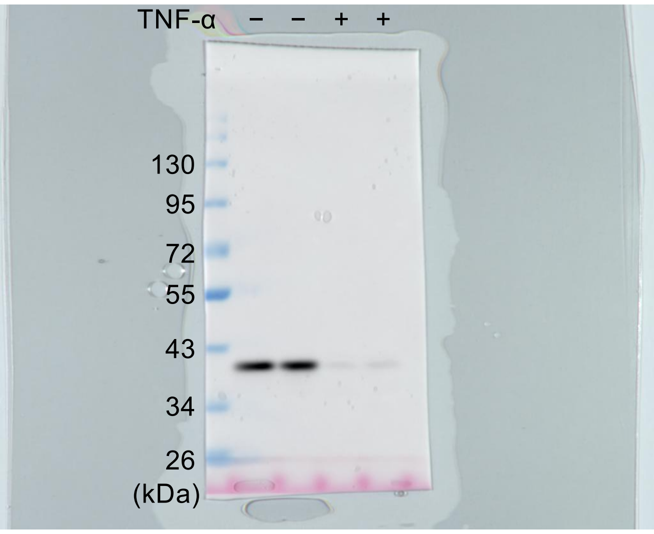

WB: IκBα

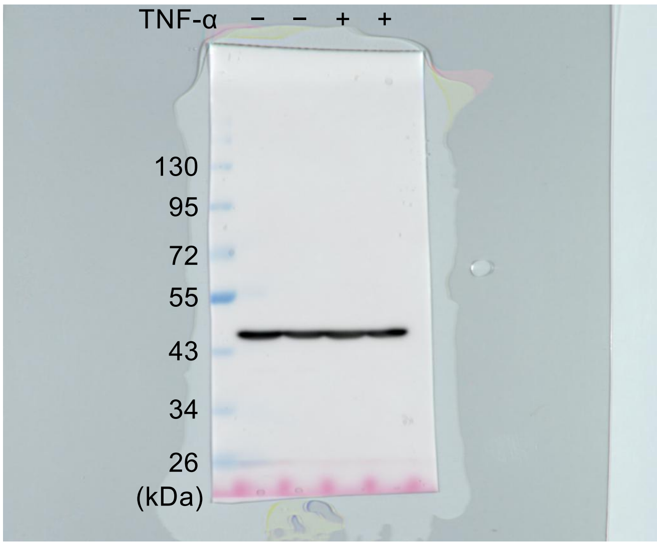

WB: β-Actin (reprobed)

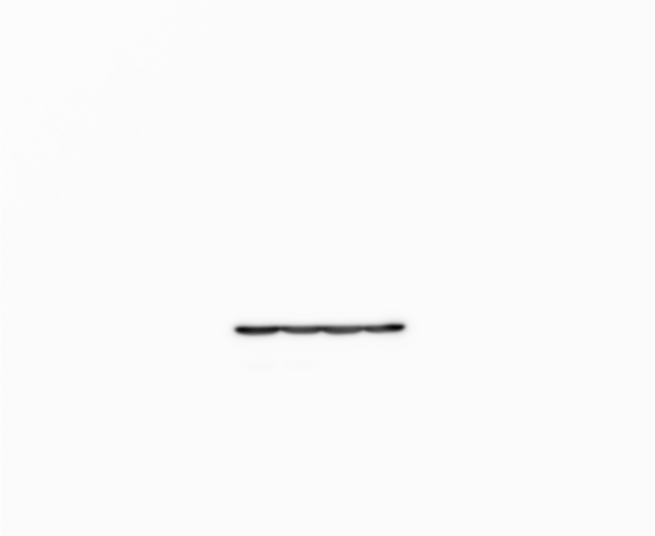

Figure S18: Original blots (2) in Figure 6D

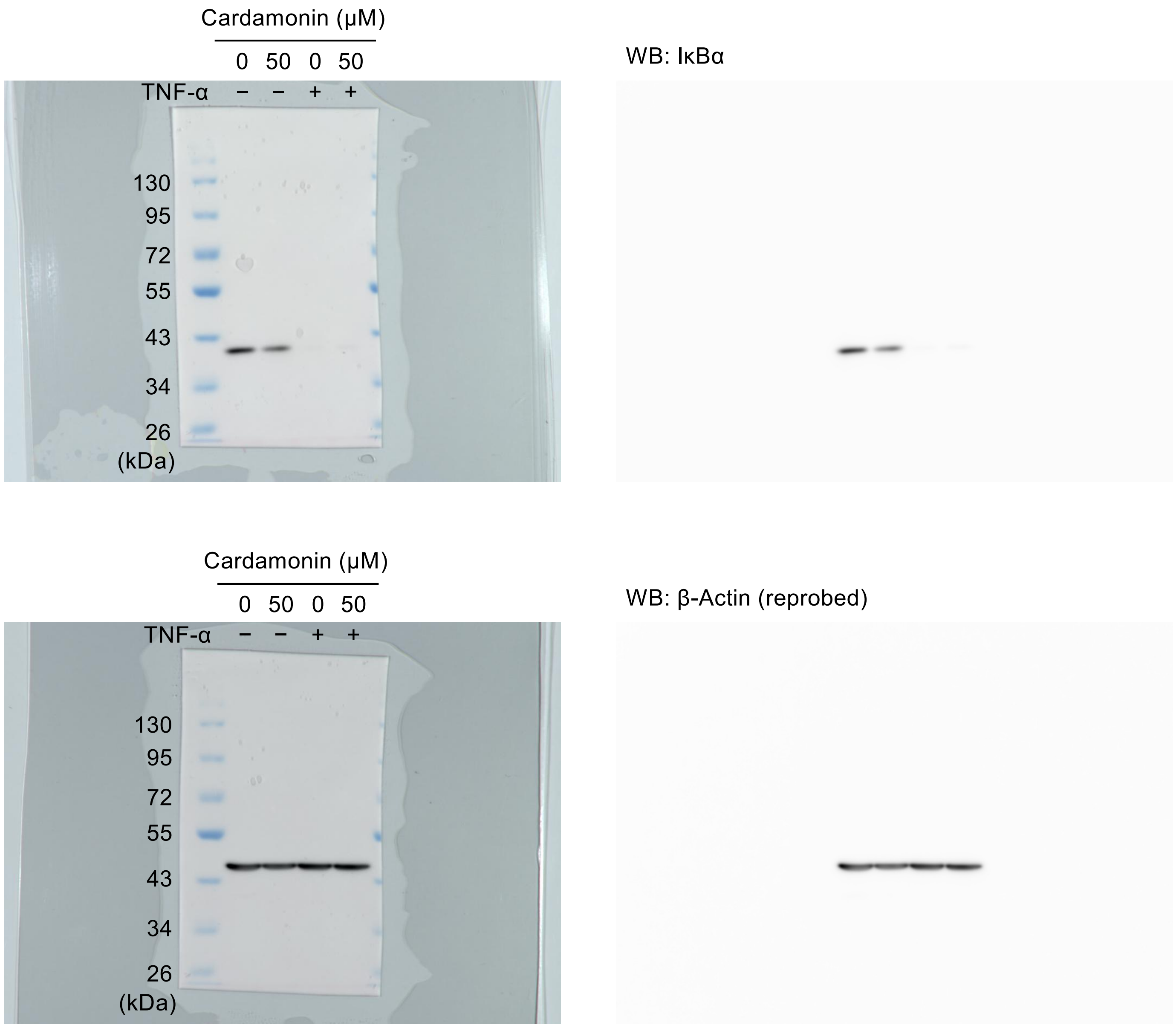

Figure S19: Original blots (3) in Figure 6D

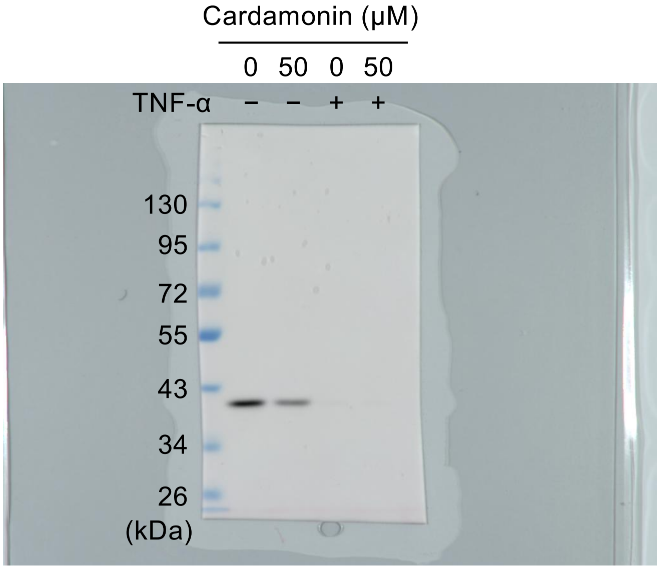

WB: IκBα

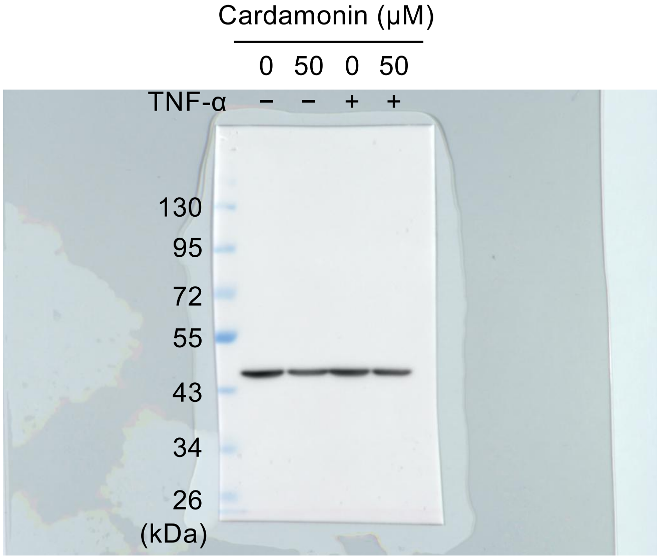

WB: β-Actin (reprobed)

Figure S20: Original blots in Figure 6E

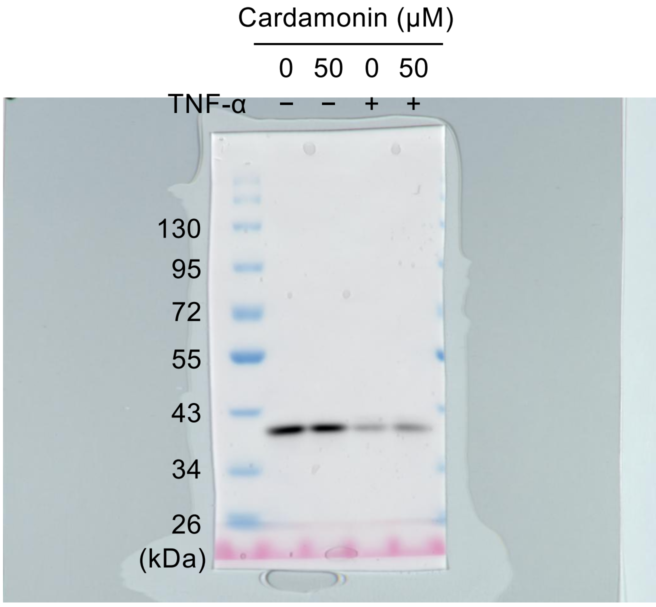

WB: IκBα

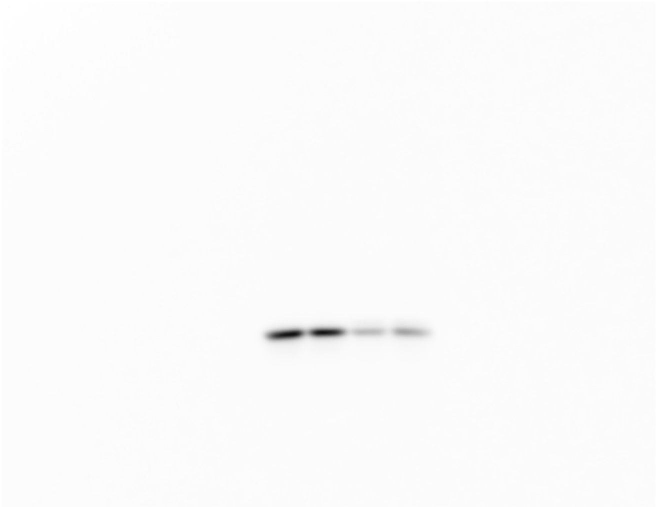

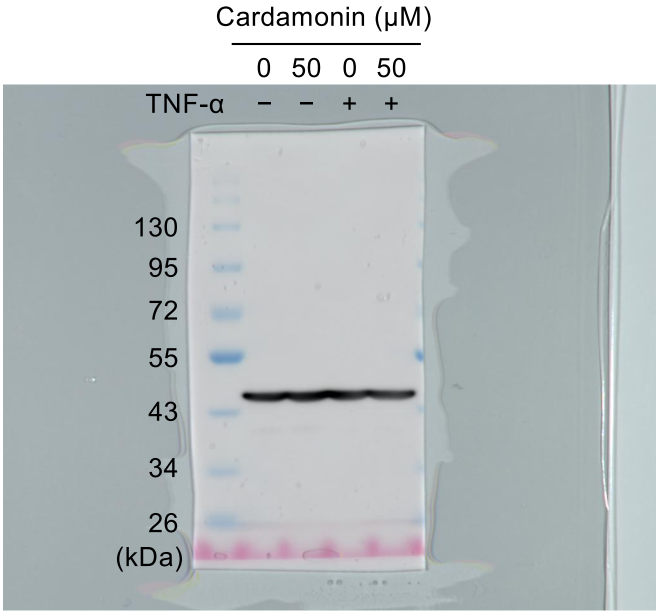

WB: β-Actin (reprobed)

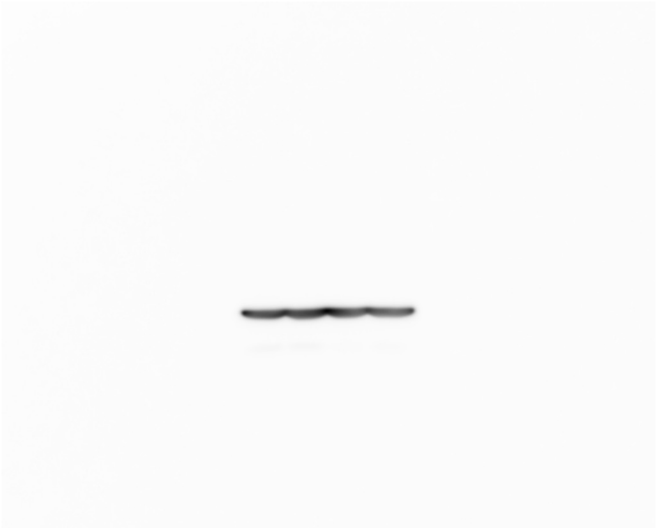

Figure S21: Original blots (1) in Figure 6F

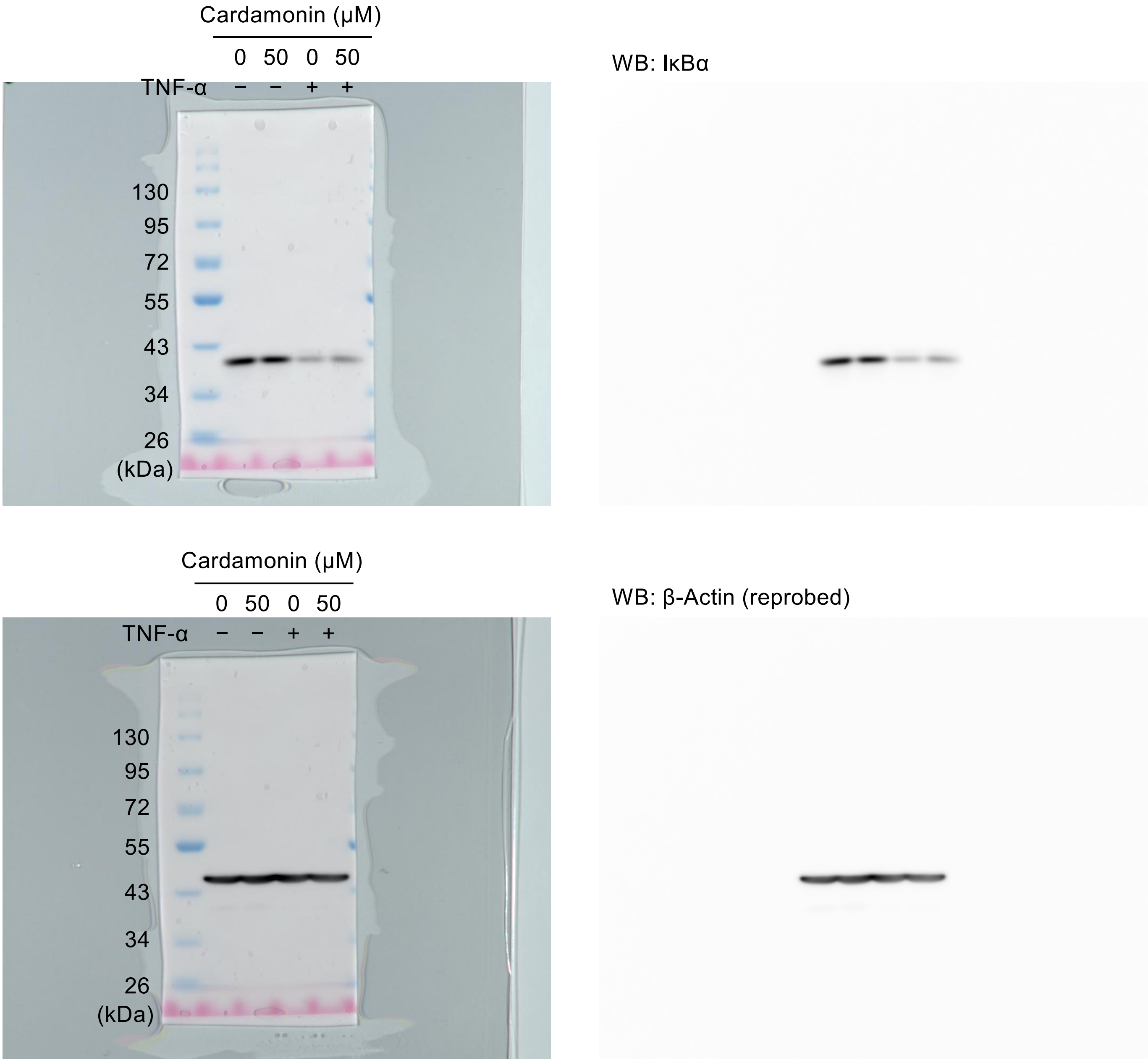

Figure S22: Original blots (2) in Figure 6F

Cardamonin (μM)

0 50 0 50

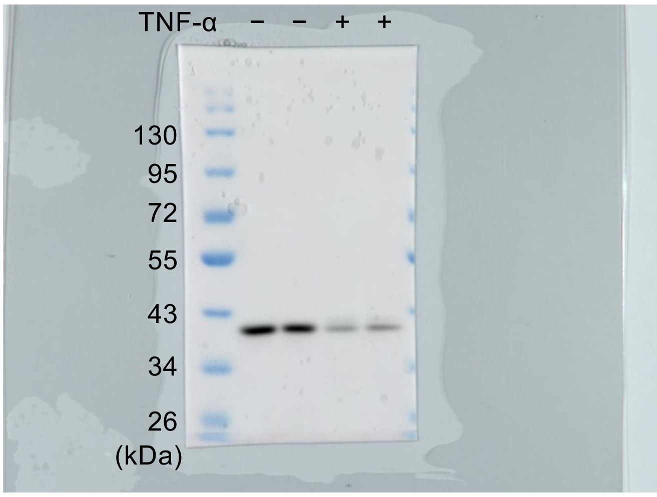

WB: IκBα

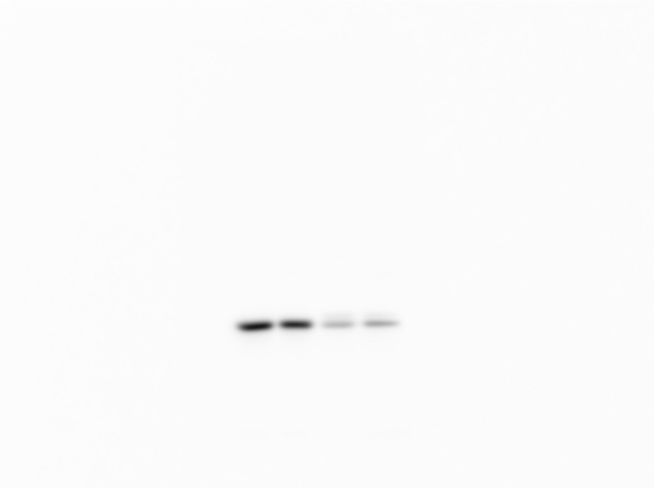

Cardamonin (μM)

0 50 0 50

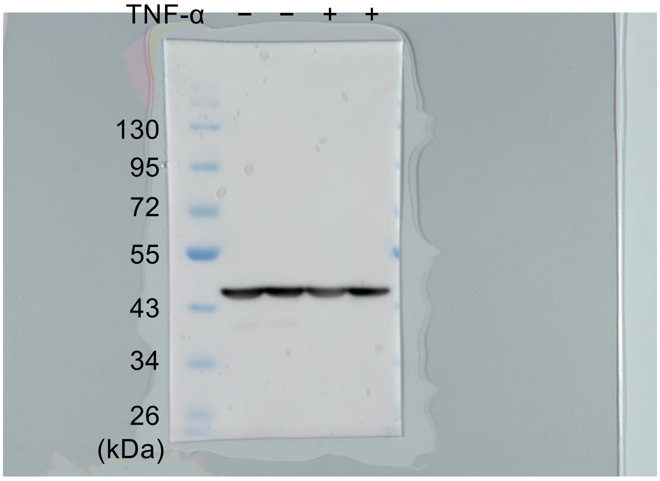

WB: β-Actin (reprobed)

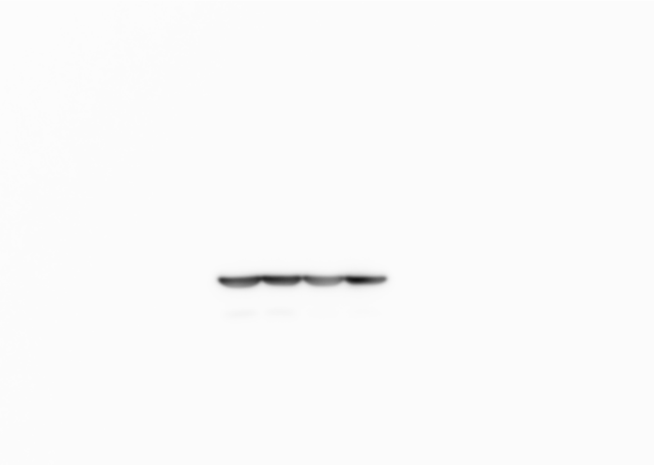

Figure S23: Original blots (3) in Figure 6F

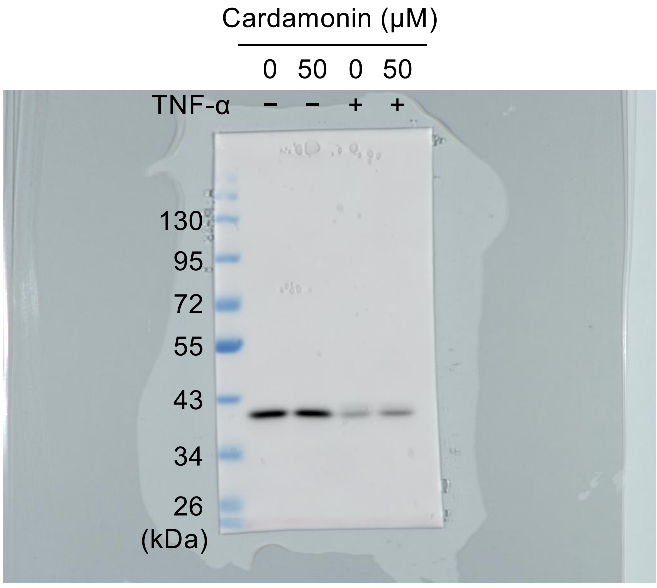

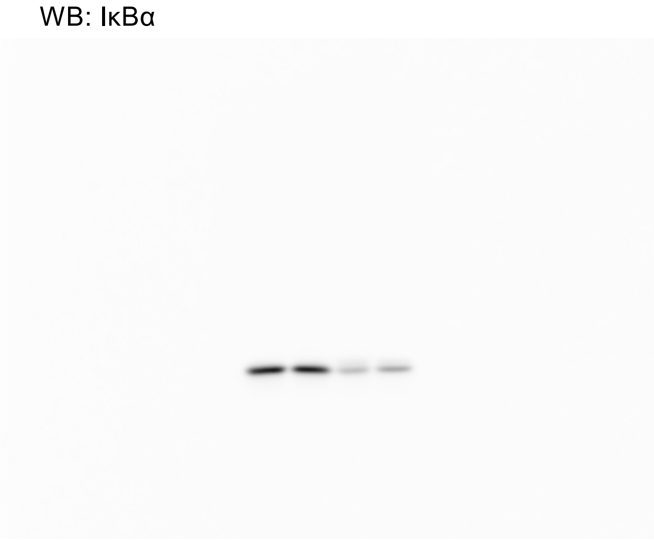

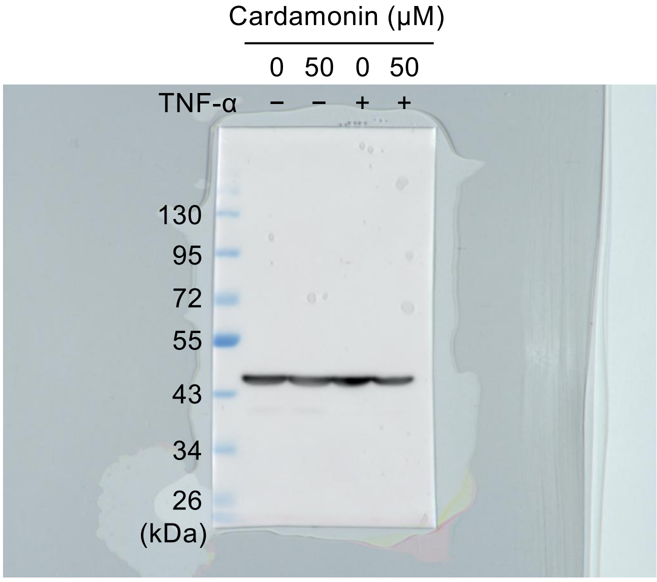

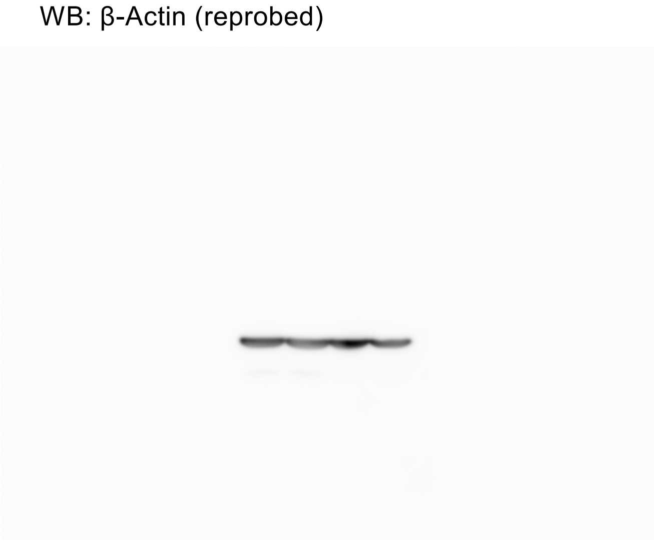

Figure S24: Original blots in Figure 7A (nucleus)

Cardamonin (μM)

0 0 5 10 25 50

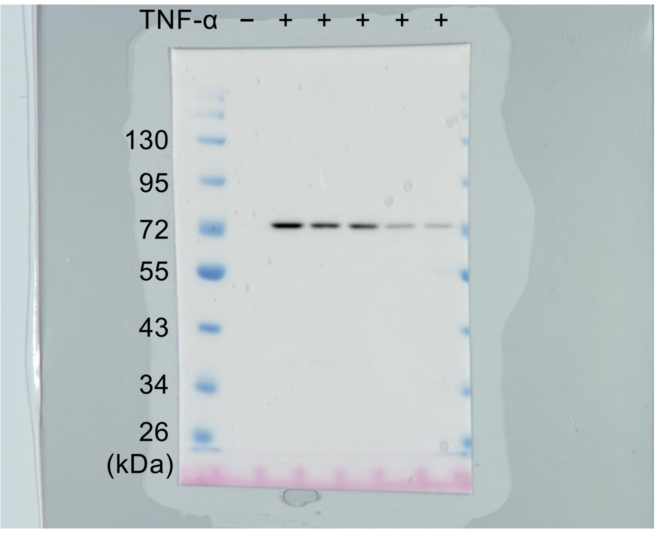

WB: RelA

Cardamonin (μM)

0 0 5 10 25 50

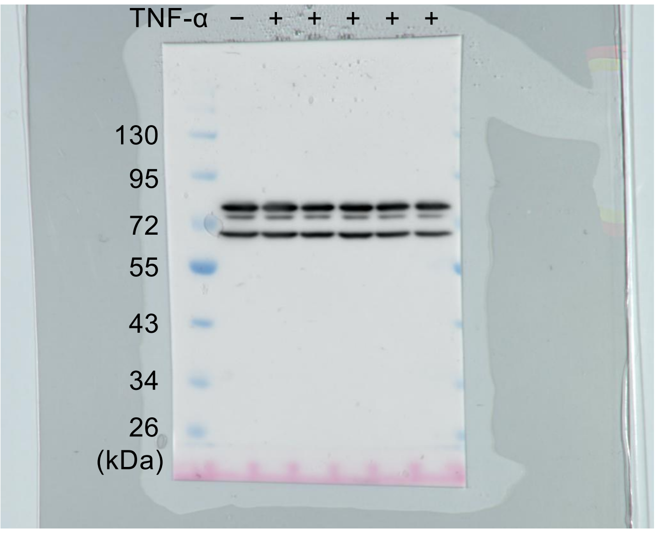

WB: Lamin A/C (reprobed)

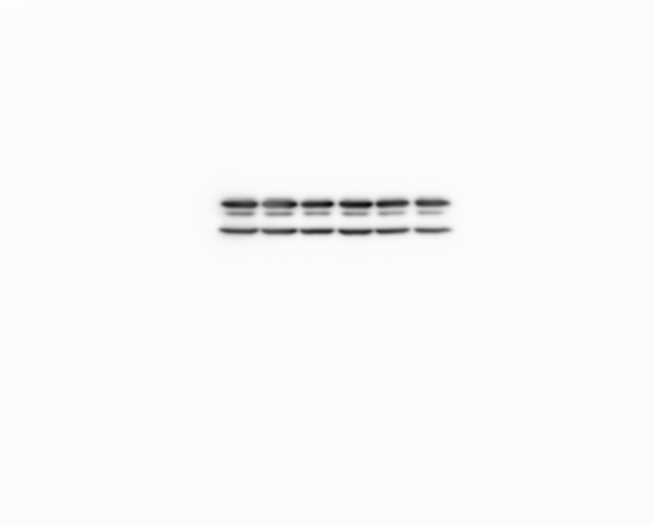

Figure S25: Original blots in Figure 7A (cytoplasm)

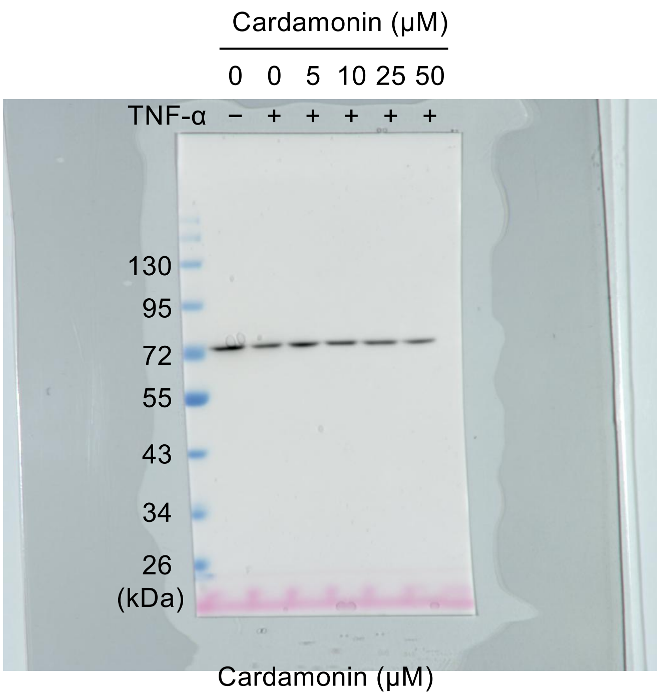

WB: RelA

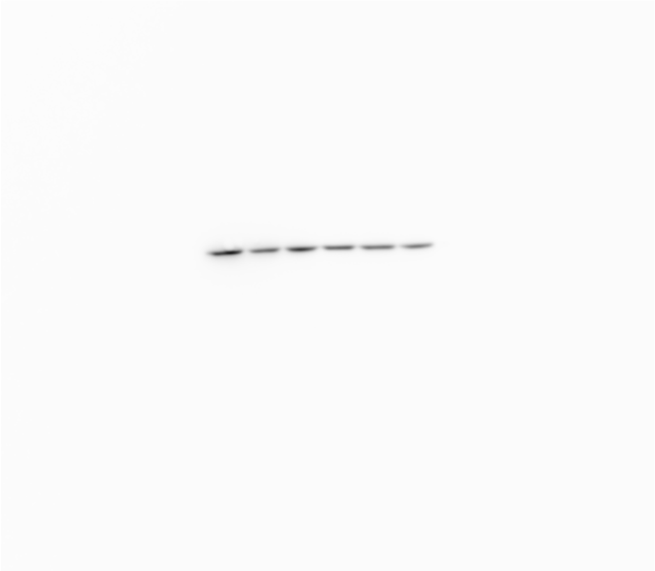

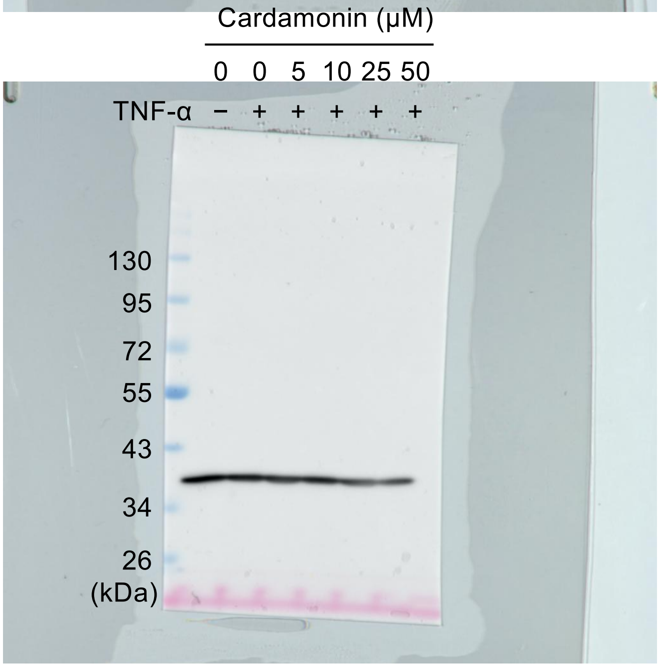

WB: GAPDH (reprobed)

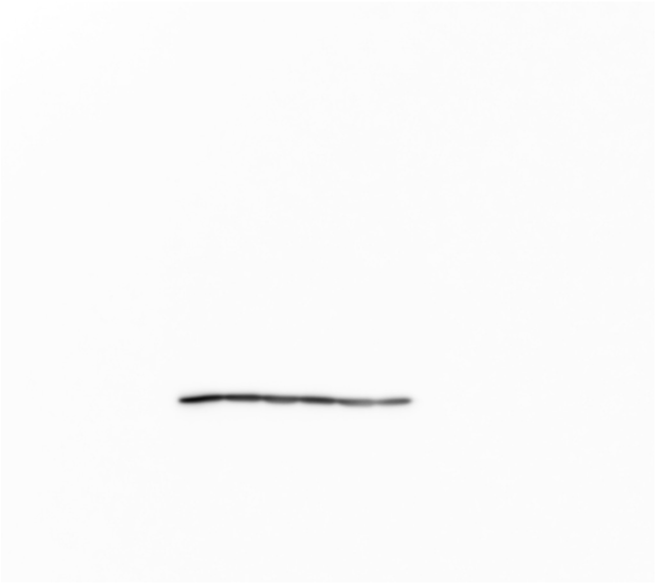

Figure S26: Original blots (1) in Figure 7B

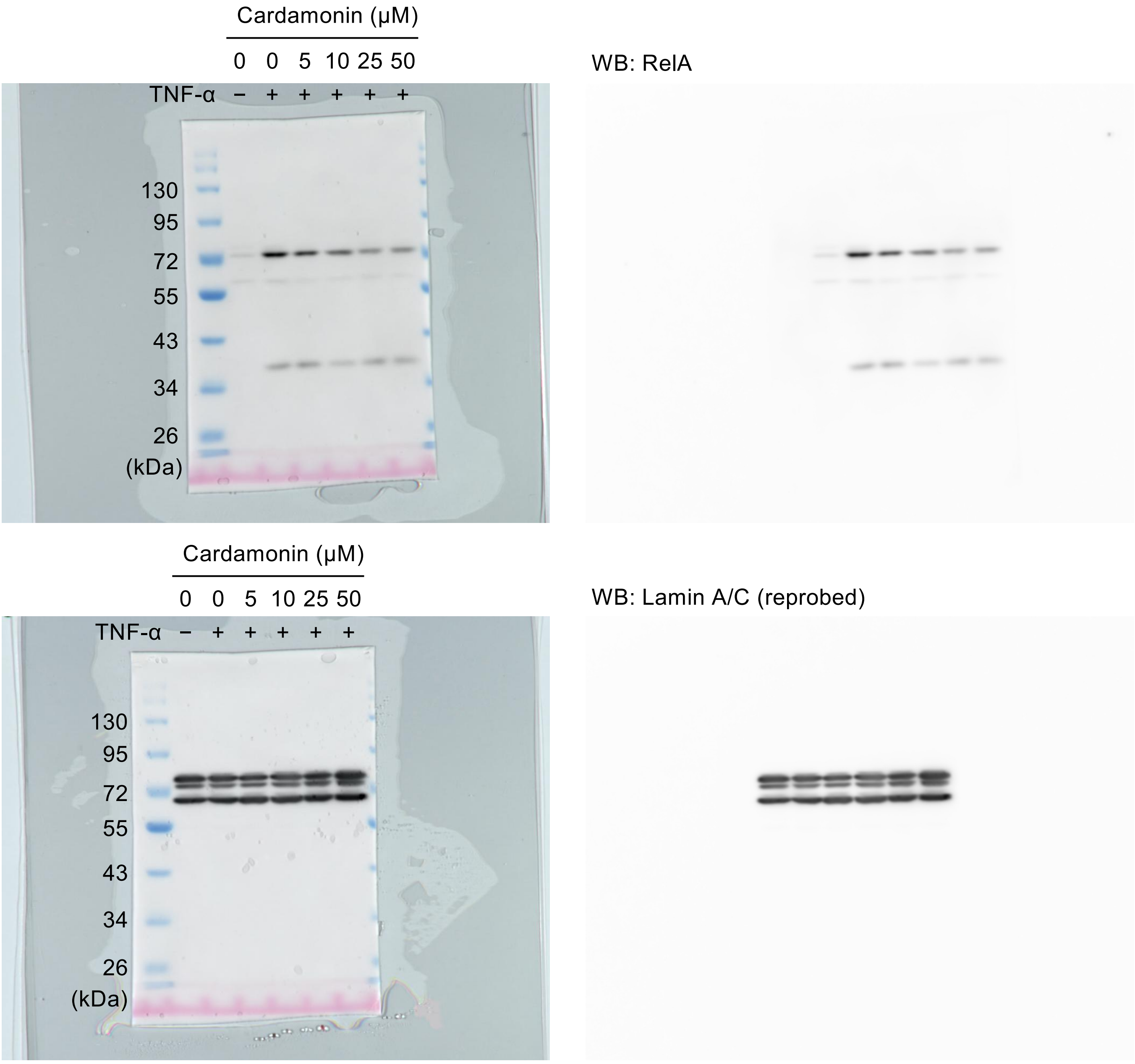

Figure S27: Original blots (2) in Figure 7B

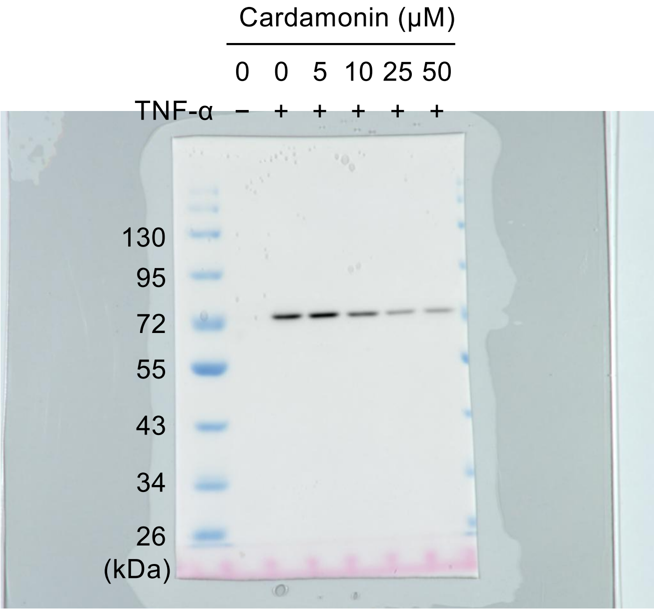

WB: RelA

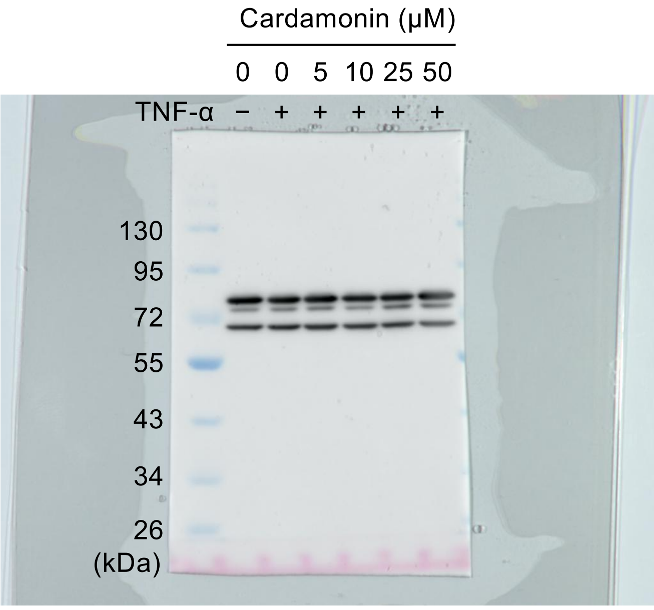

WB: Lamin A/C (reprobed)

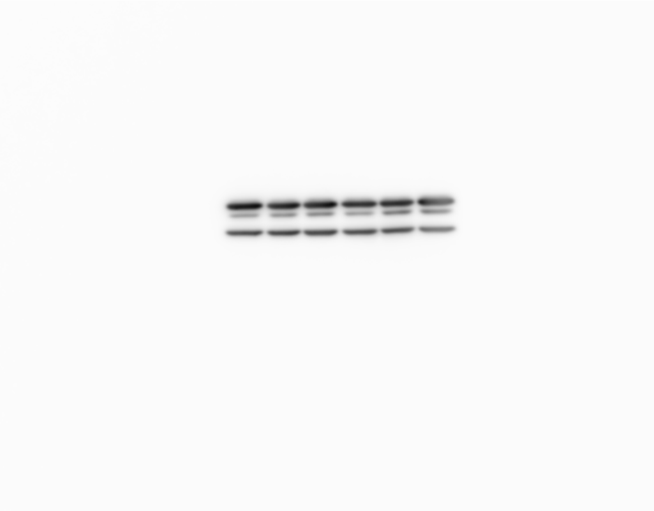

Figure S28: Original blots (3) in Figure 7B

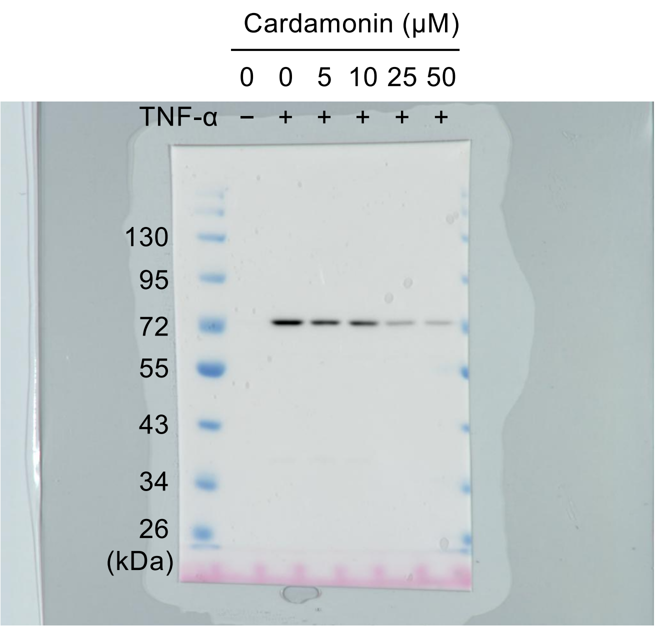

WB: RelA

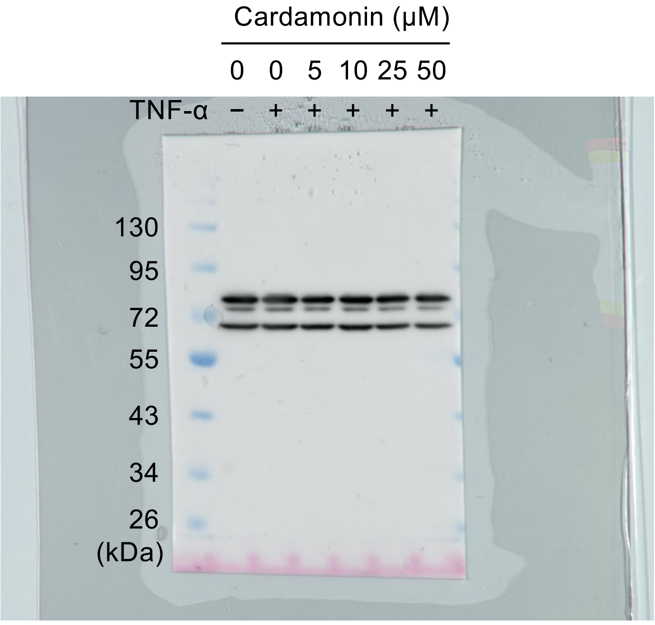

WB: Lamin A/C (reprobed)

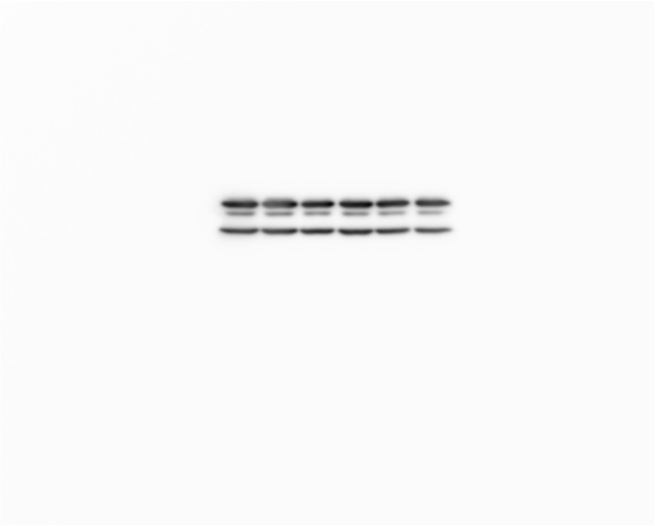

Figure S29: Original blots (1) in Figure 7C

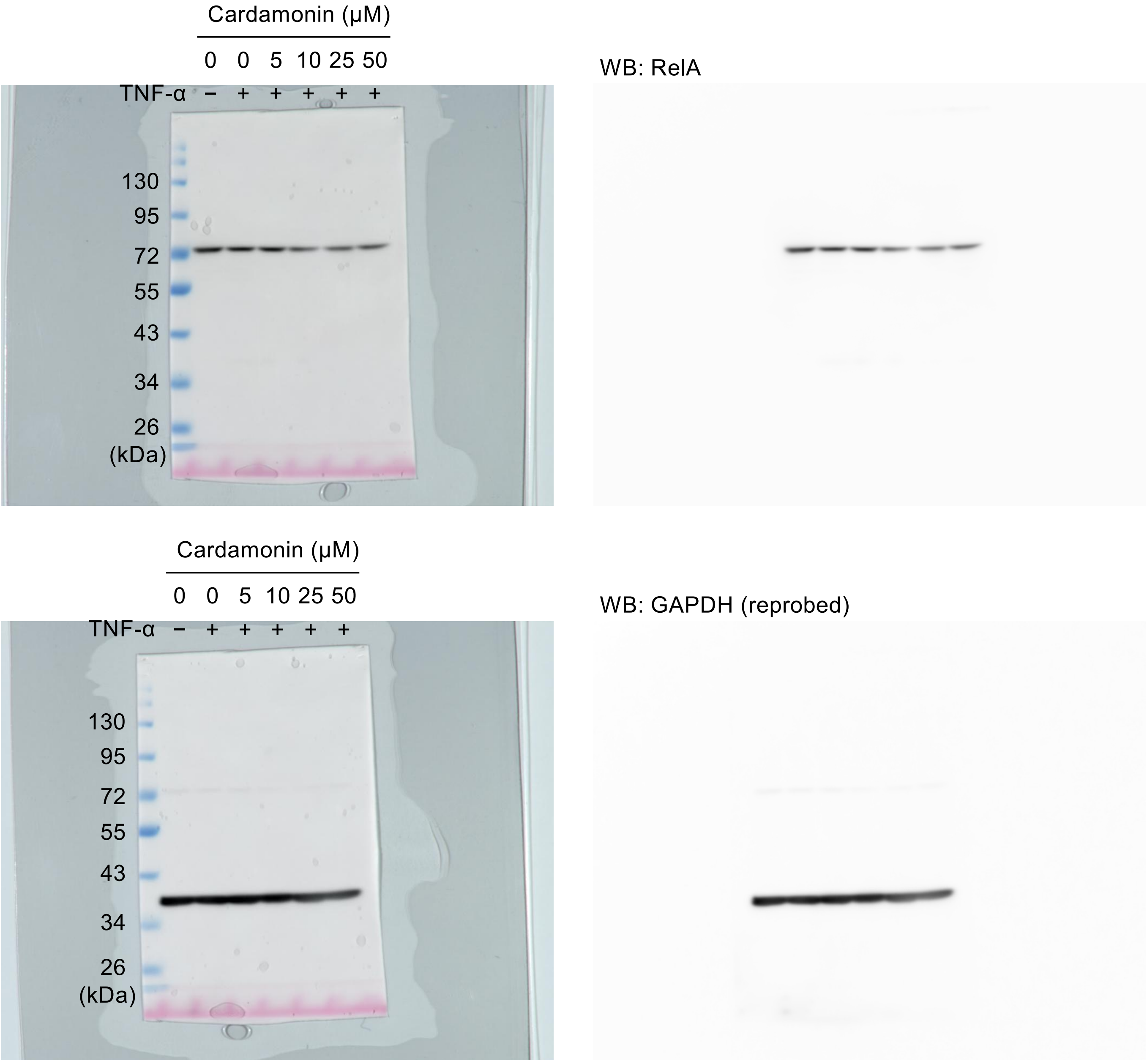

Figure S30: Original blots (2) in Figure 7C

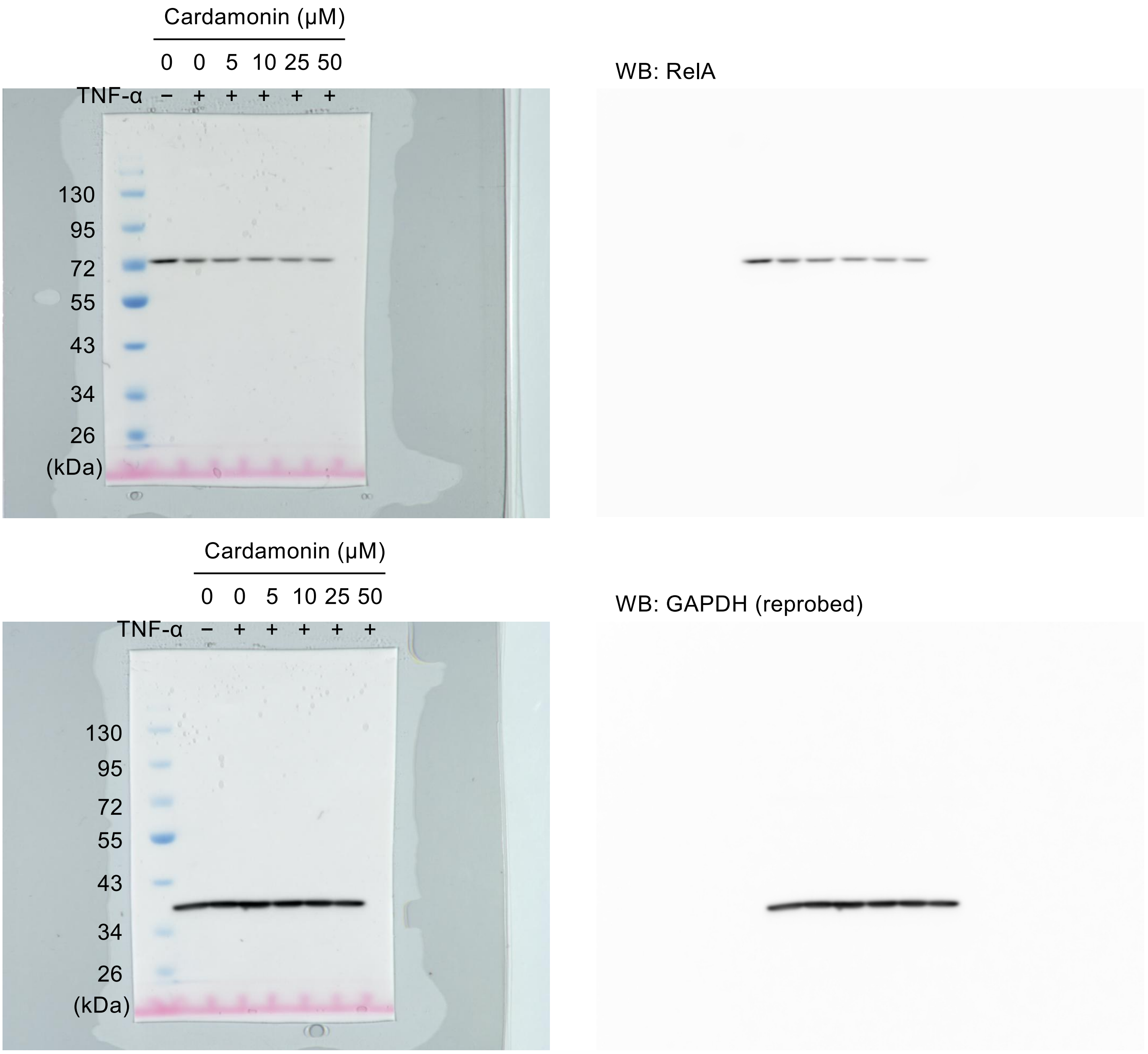

Figure S31: Original blots (3) in Figure 7C

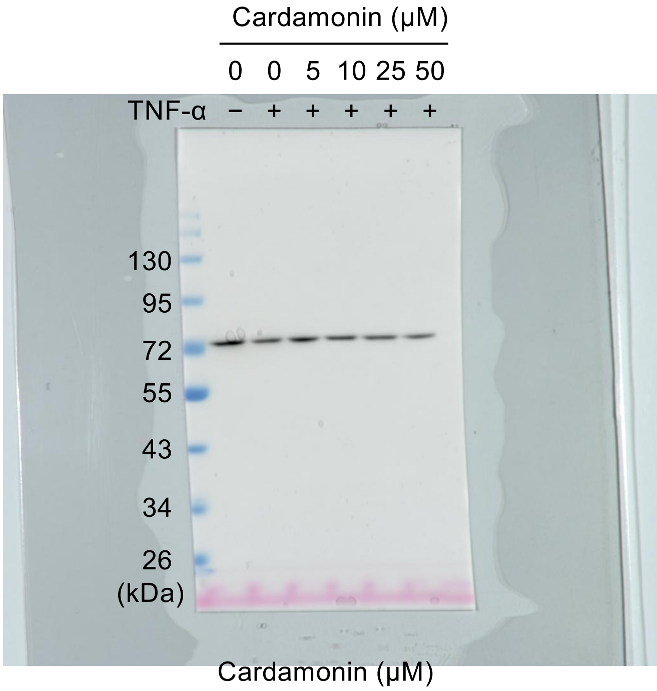

WB: RelA

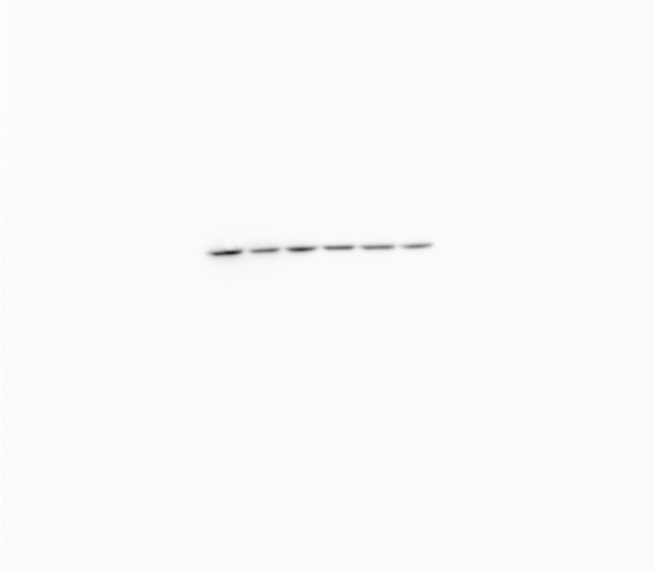

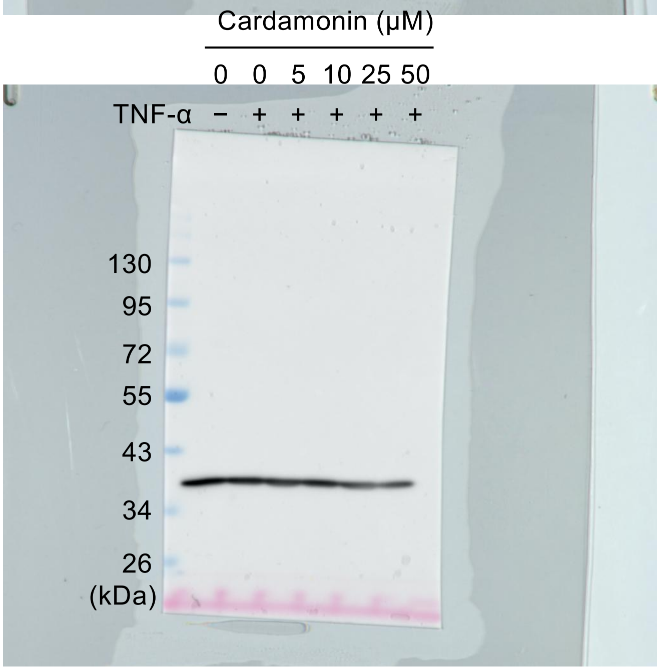

WB: GAPDH (reprobed)

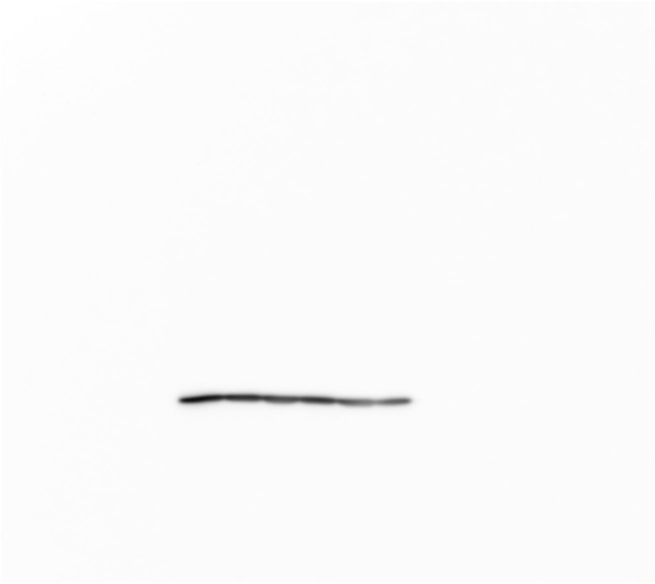

Figure S32: Original blots in Figure 7D (nucleus)

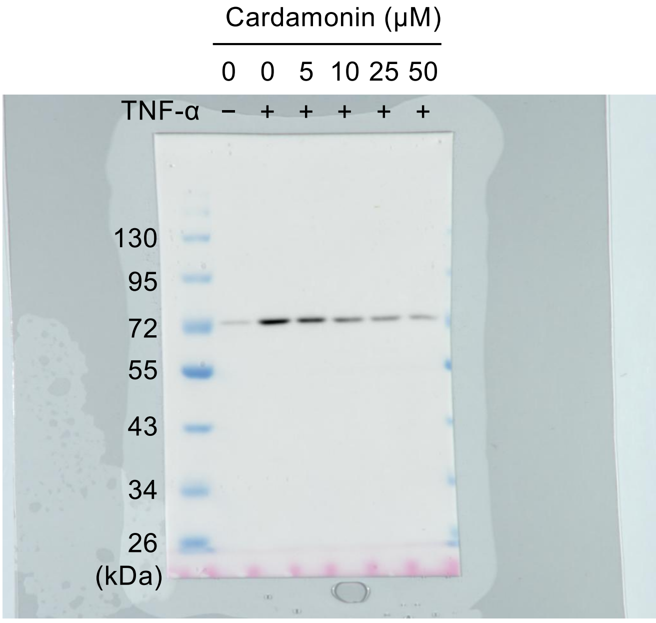

WB: RelA

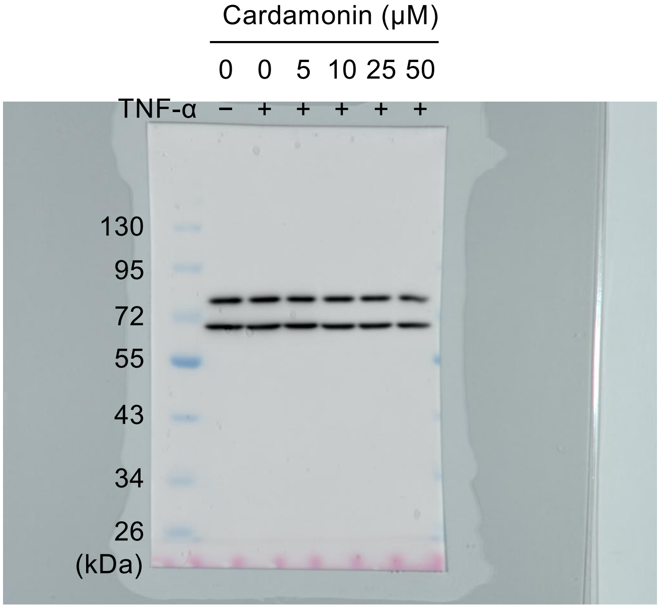

WB: Lamin A/C (reprobed)

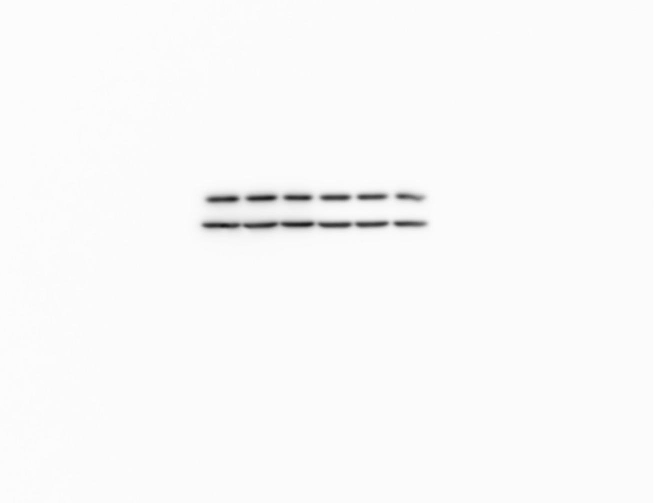

Figure S33: Original blots in Figure 7D (cytoplasm)

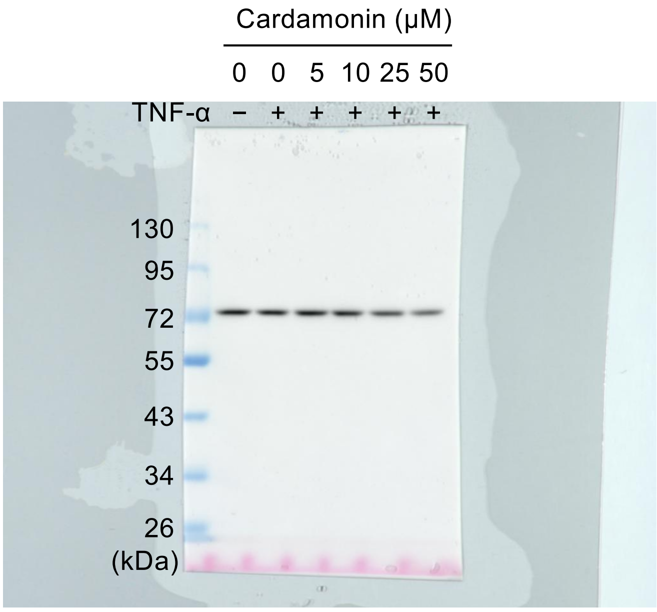

WB: RelA

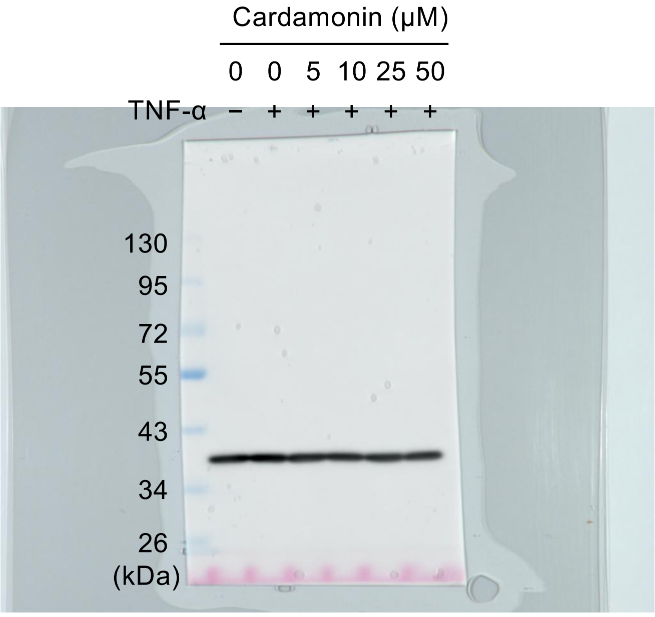

WB: GAPDH (reprobed)

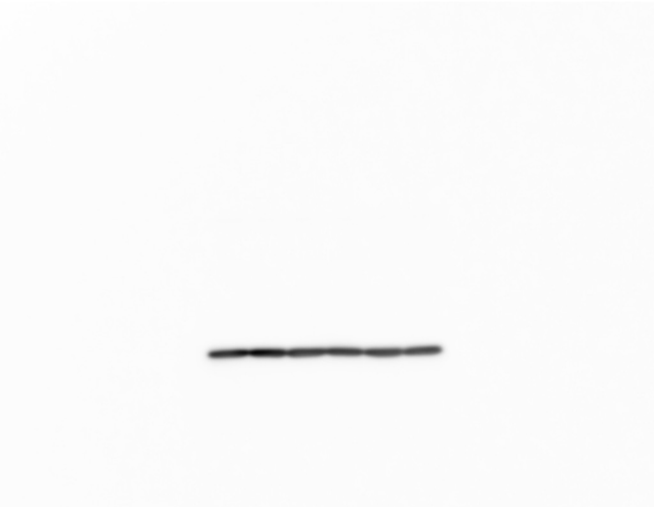

Figure S34: Original blots (1) in Figure 7E

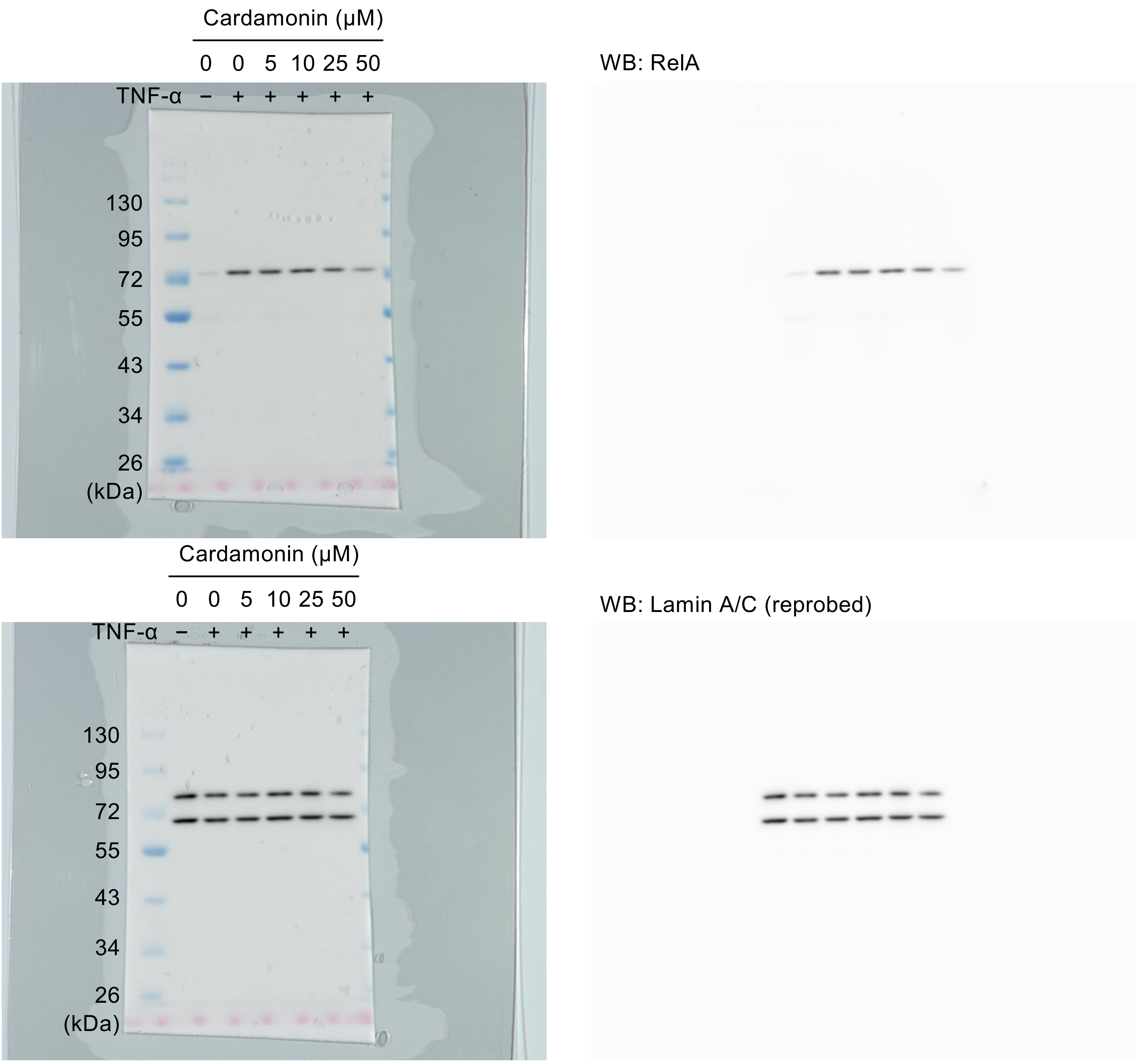

Figure S35: Original blots (2) in Figure 7E

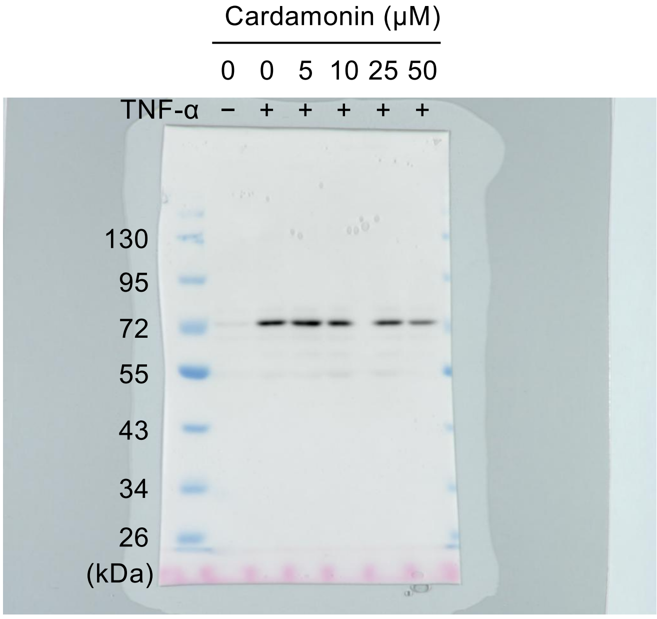

WB: RelA

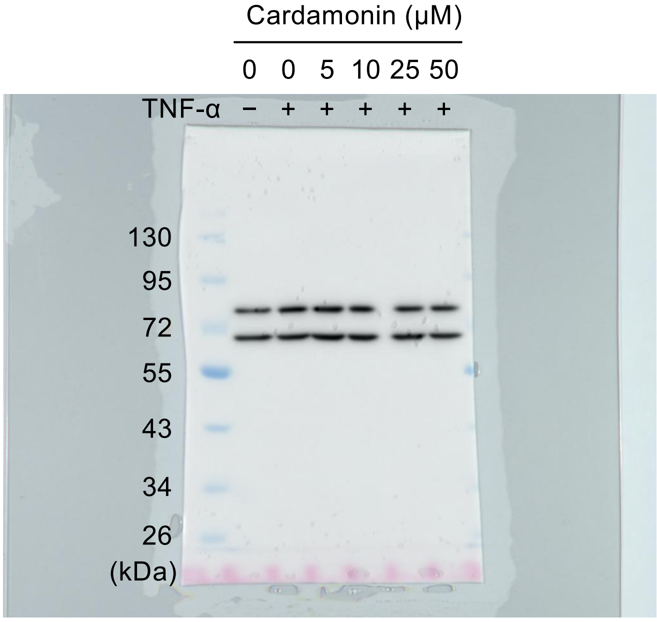

WB: Lamin A/C (reprobed)

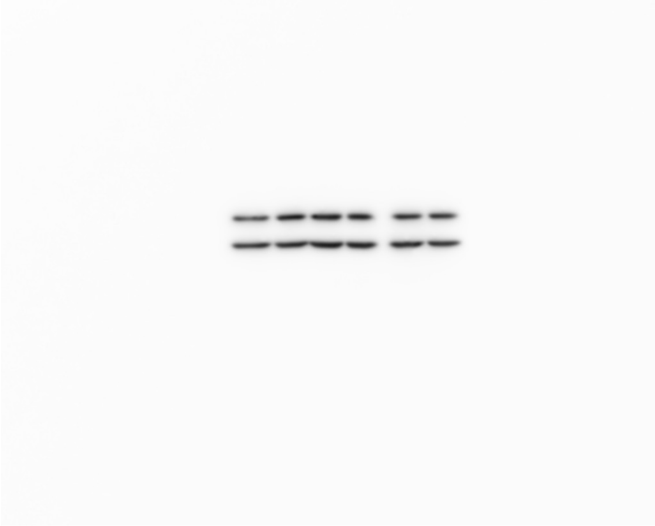

Figure S36: Original blots (3) in Figure 7E

Cardamonin (μM)

0 0 5 10 25 50

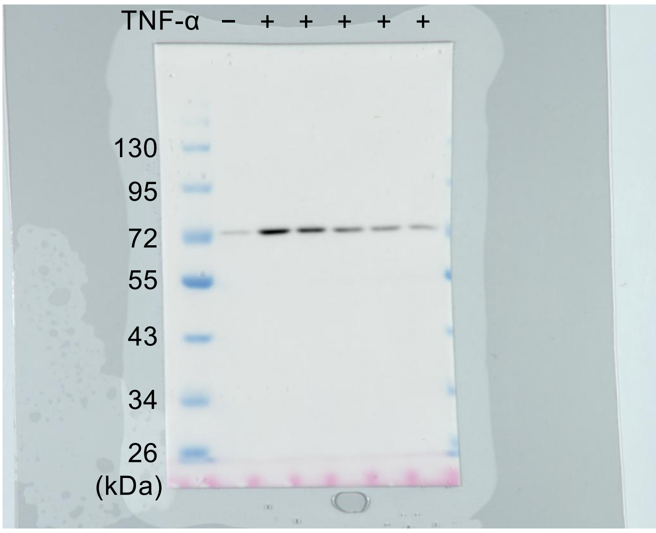

WB: RelA

Cardamonin (μM)

0 0 5 10 25 50

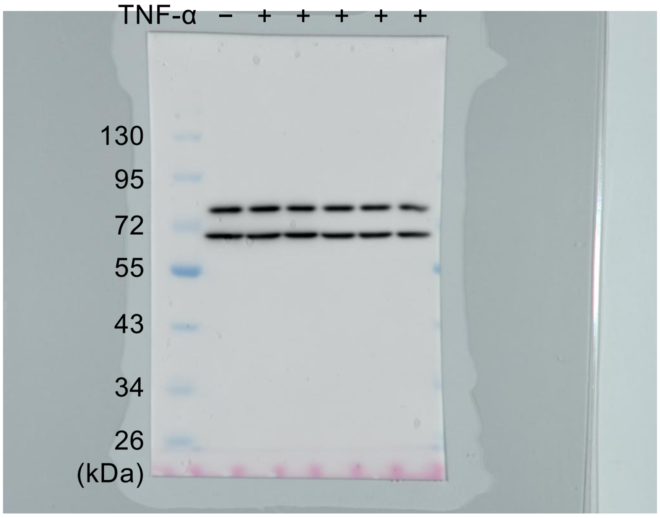

WB: Lamin A/C (reprobed)

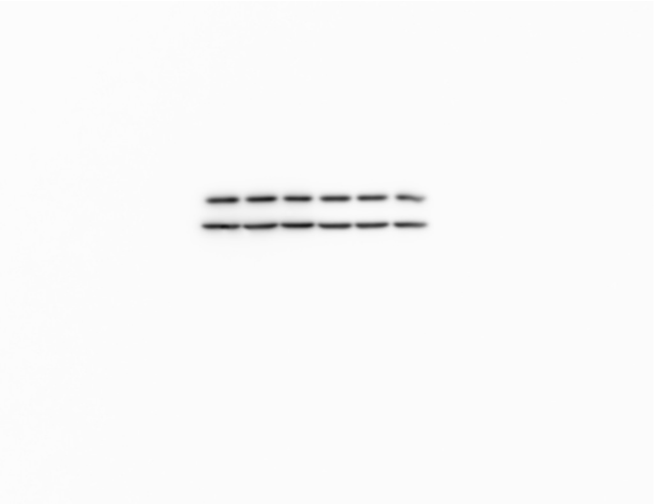

Figure S37: Original blots (1) in Figure 7F

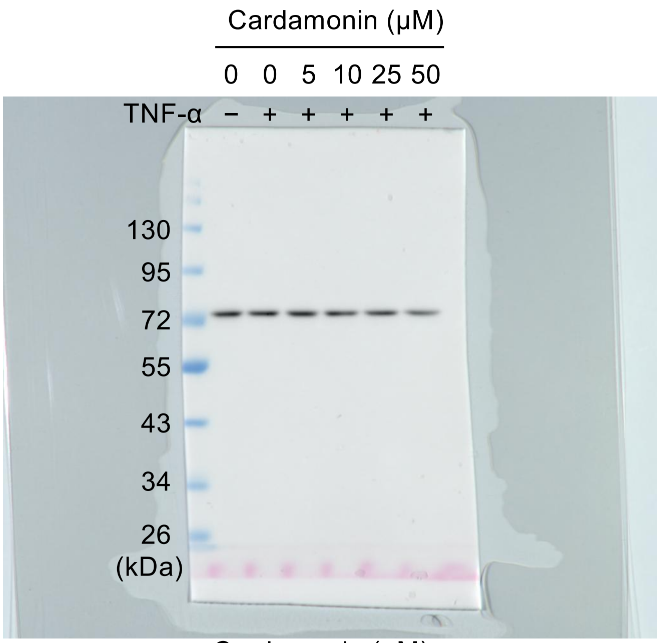

WB: RelA

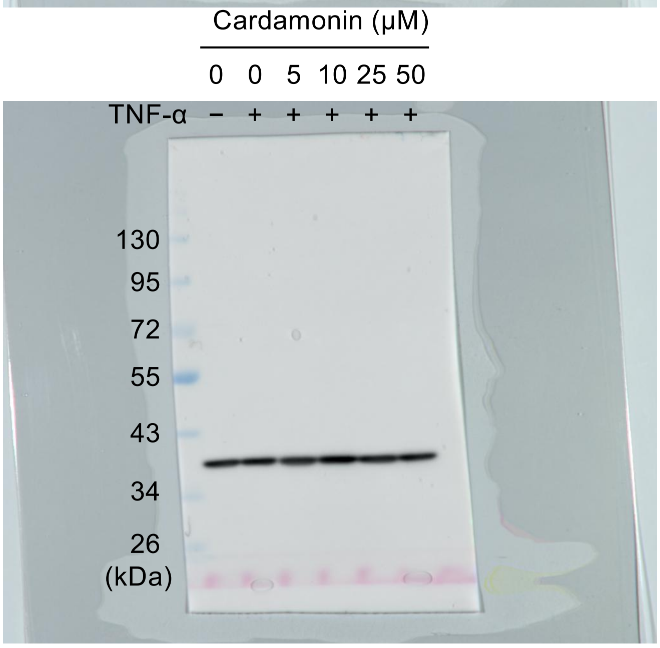

WB: GAPDH (reprobed)

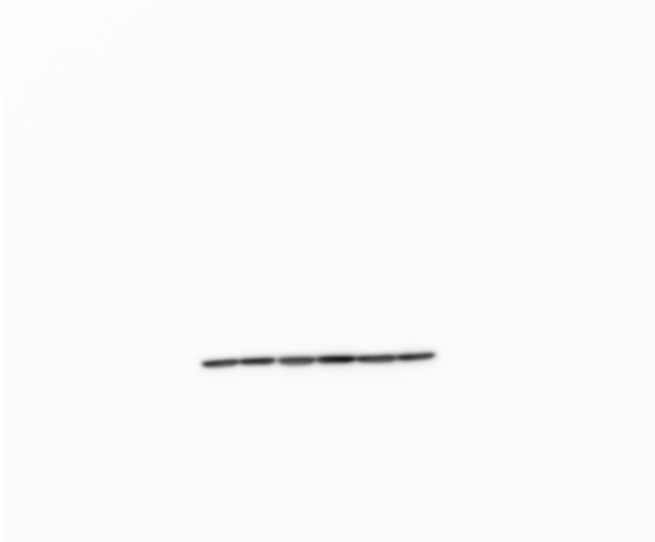

Figure S38: Original blots (2) in Figure 7F

Cardamonin (μM)

0 0 5 10 25 50

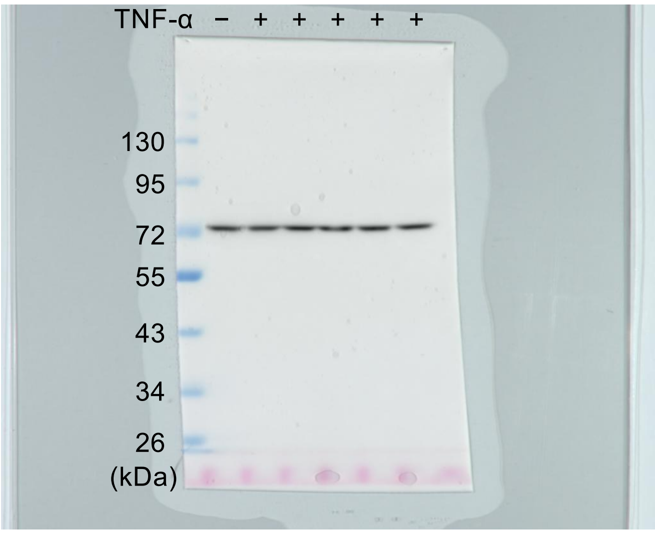

WB: RelA

Cardamonin (μM)

0 0 5 10 25 50

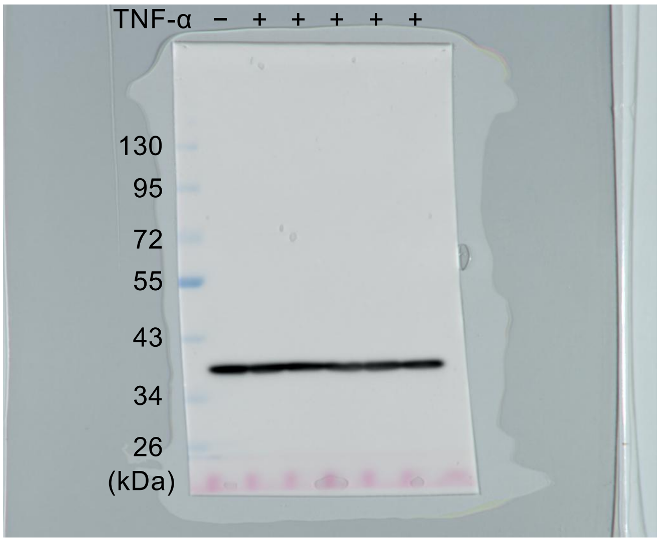

WB: GAPDH (reprobed)

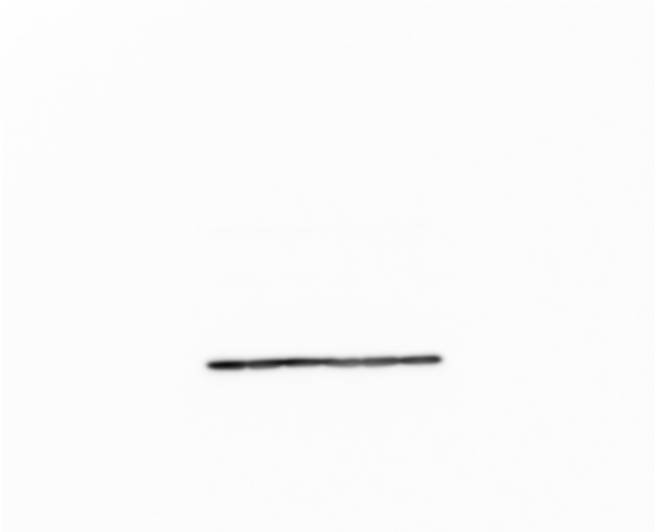

Figure S39: Original blots (3) in Figure 7F

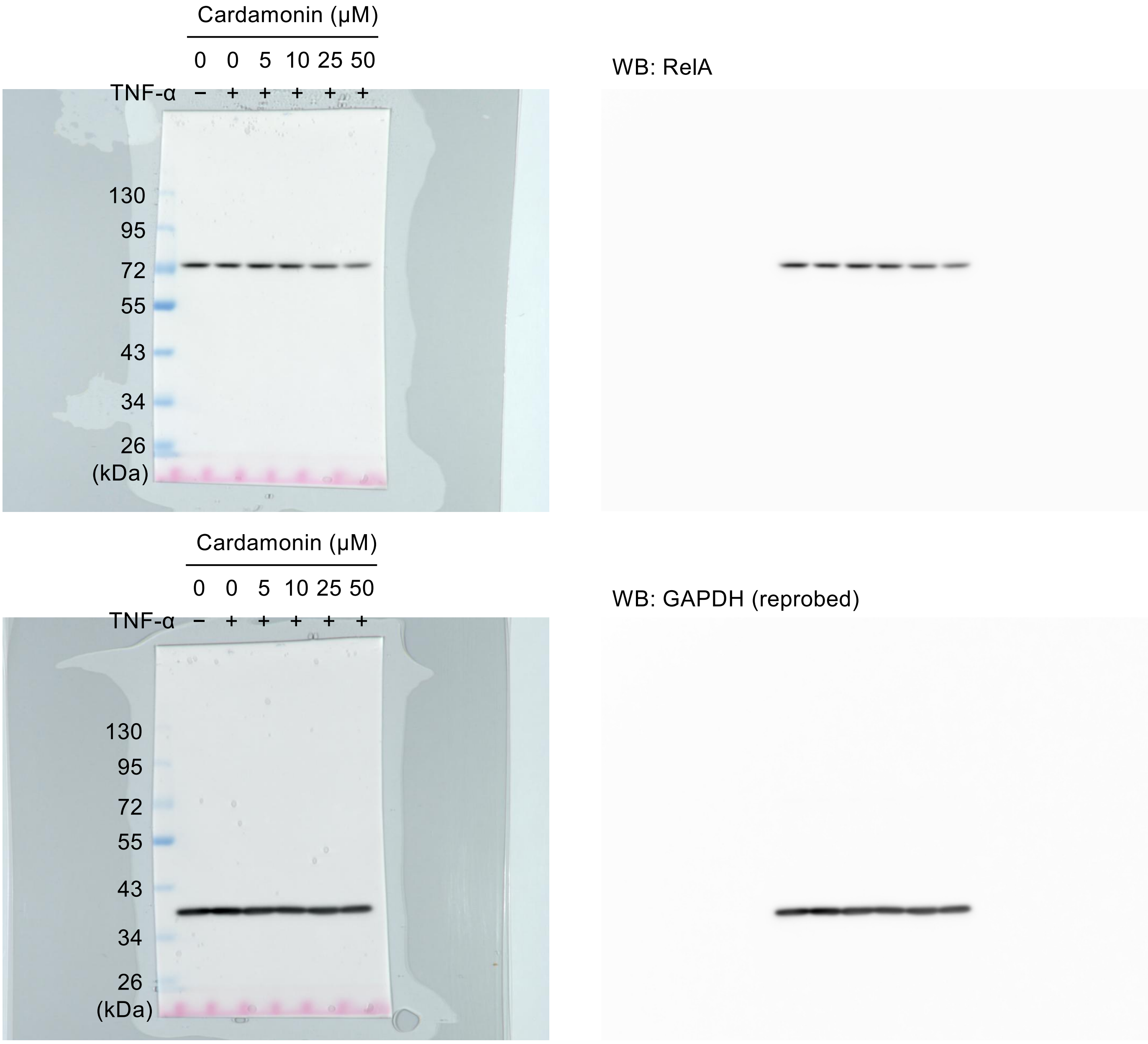

Figure S40: Original blots in Figure 10A

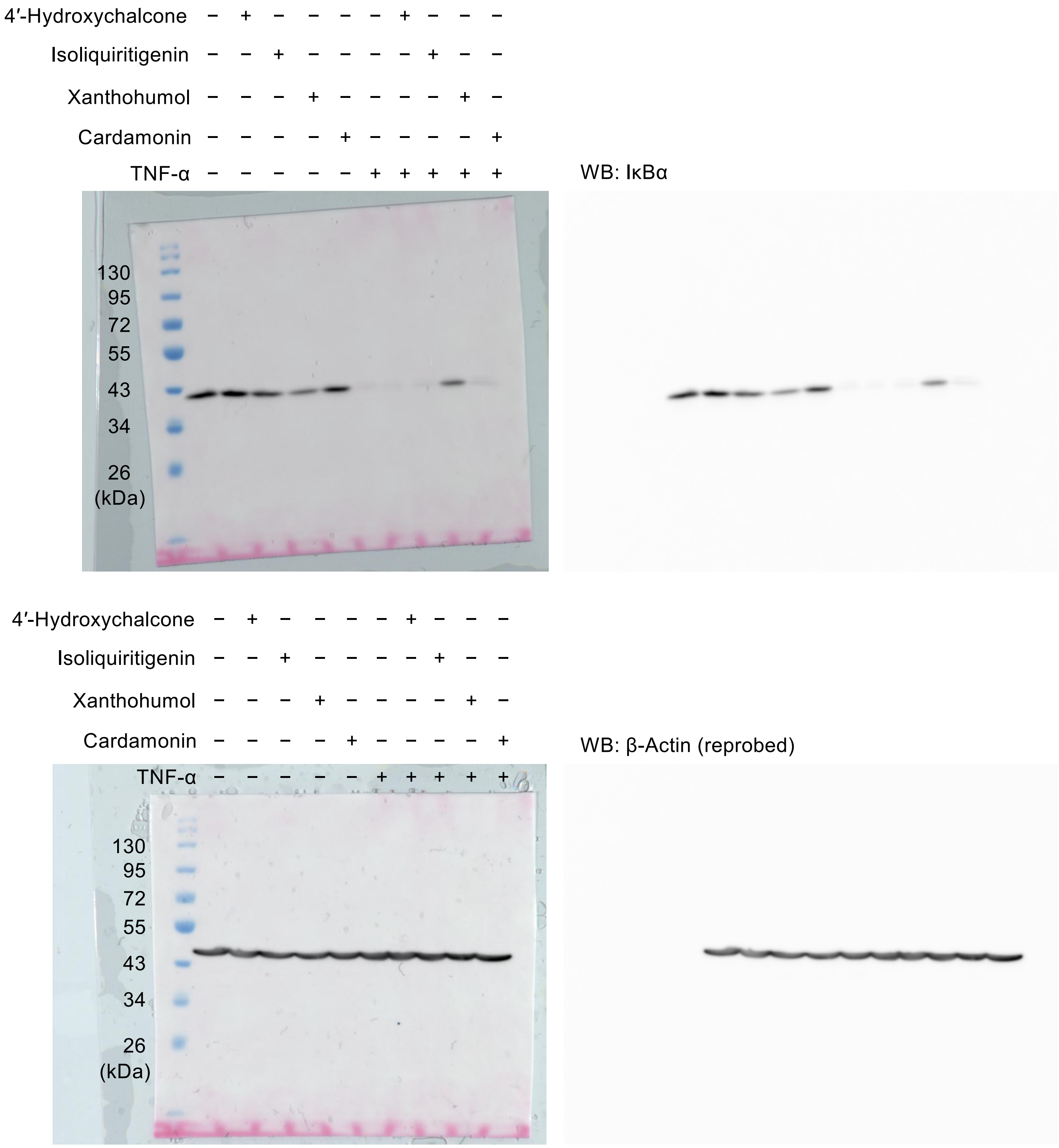

Figure S41: Original blots (1) in Figure 10B

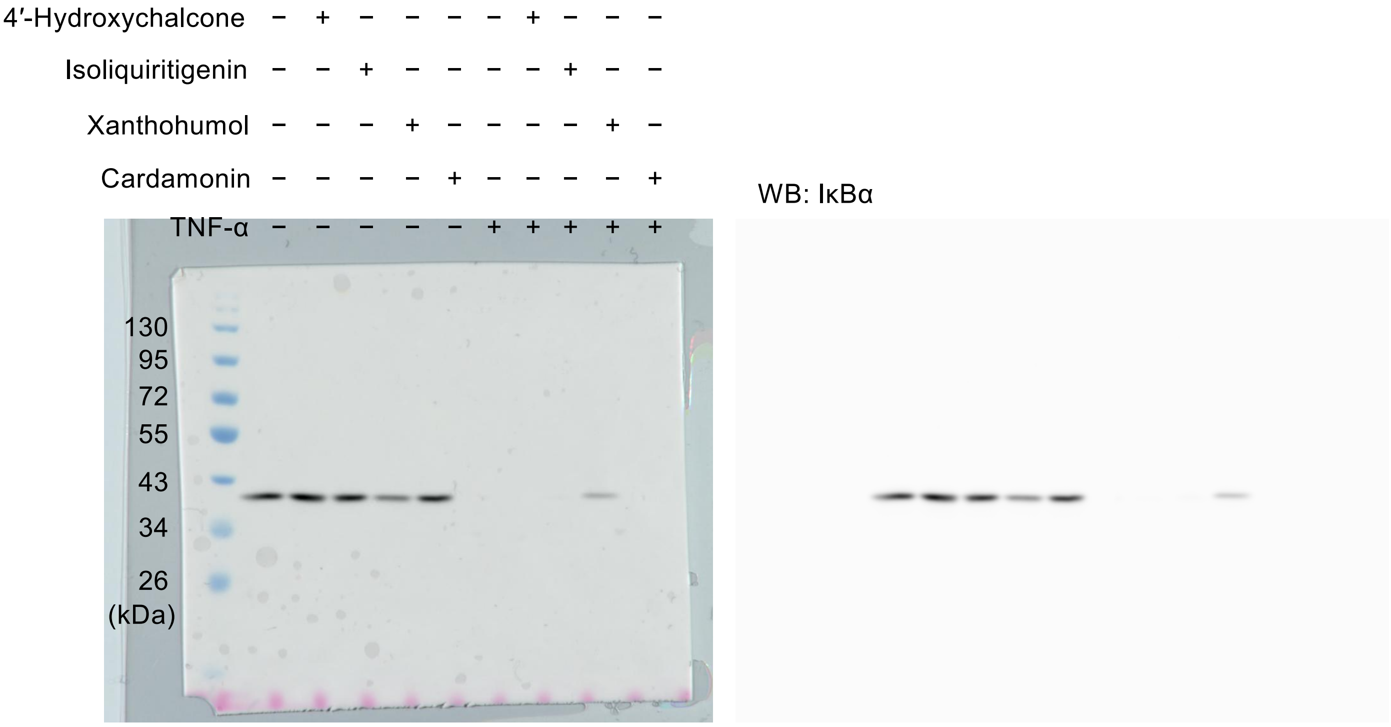

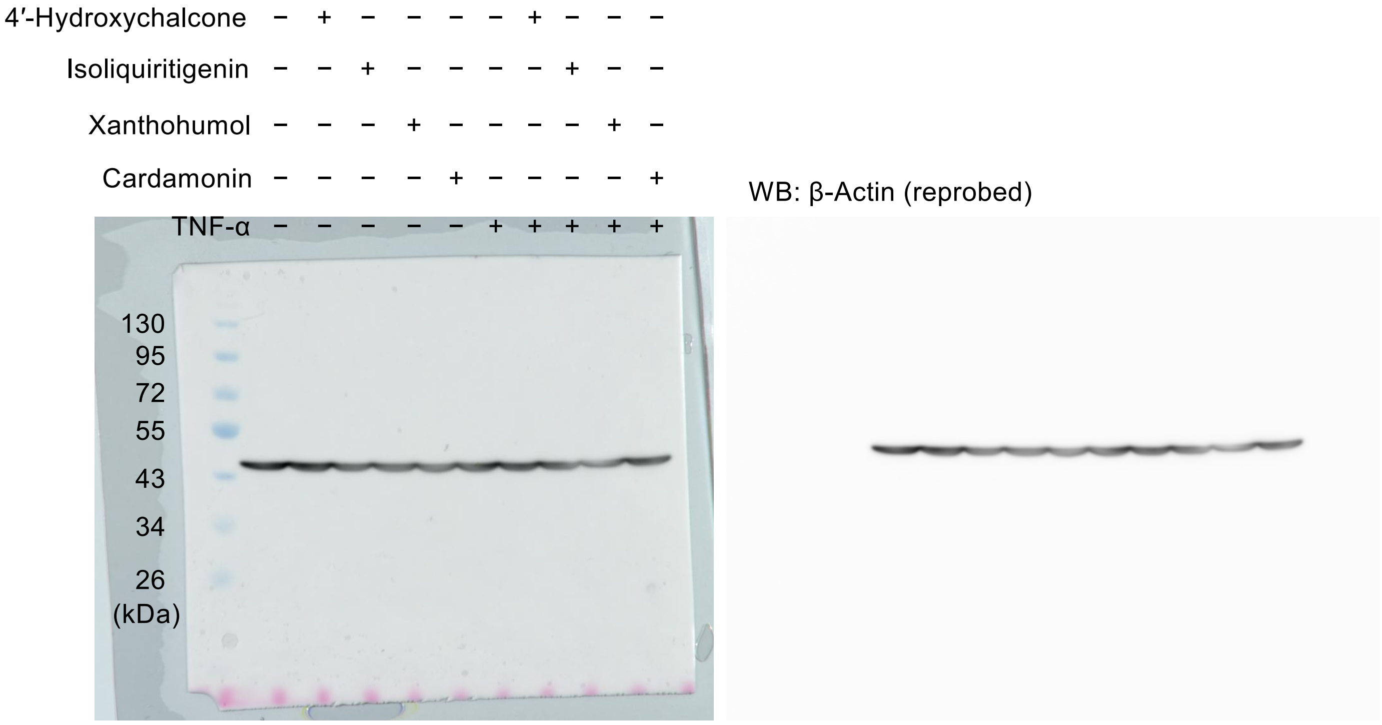

Figure S42: Original blots (2) in Figure 10B

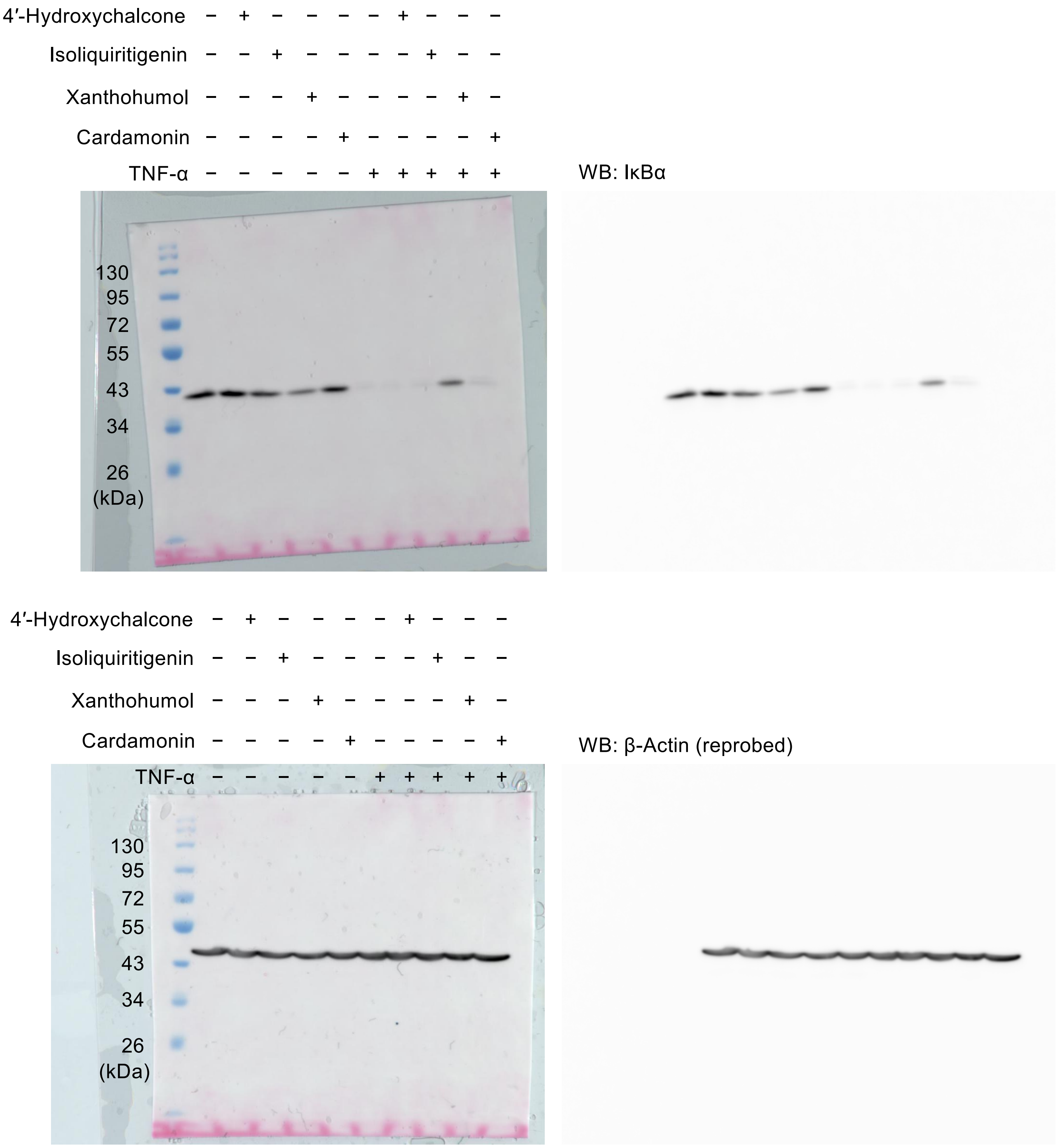

Figure S43: Original blots (3) in Figure 10B

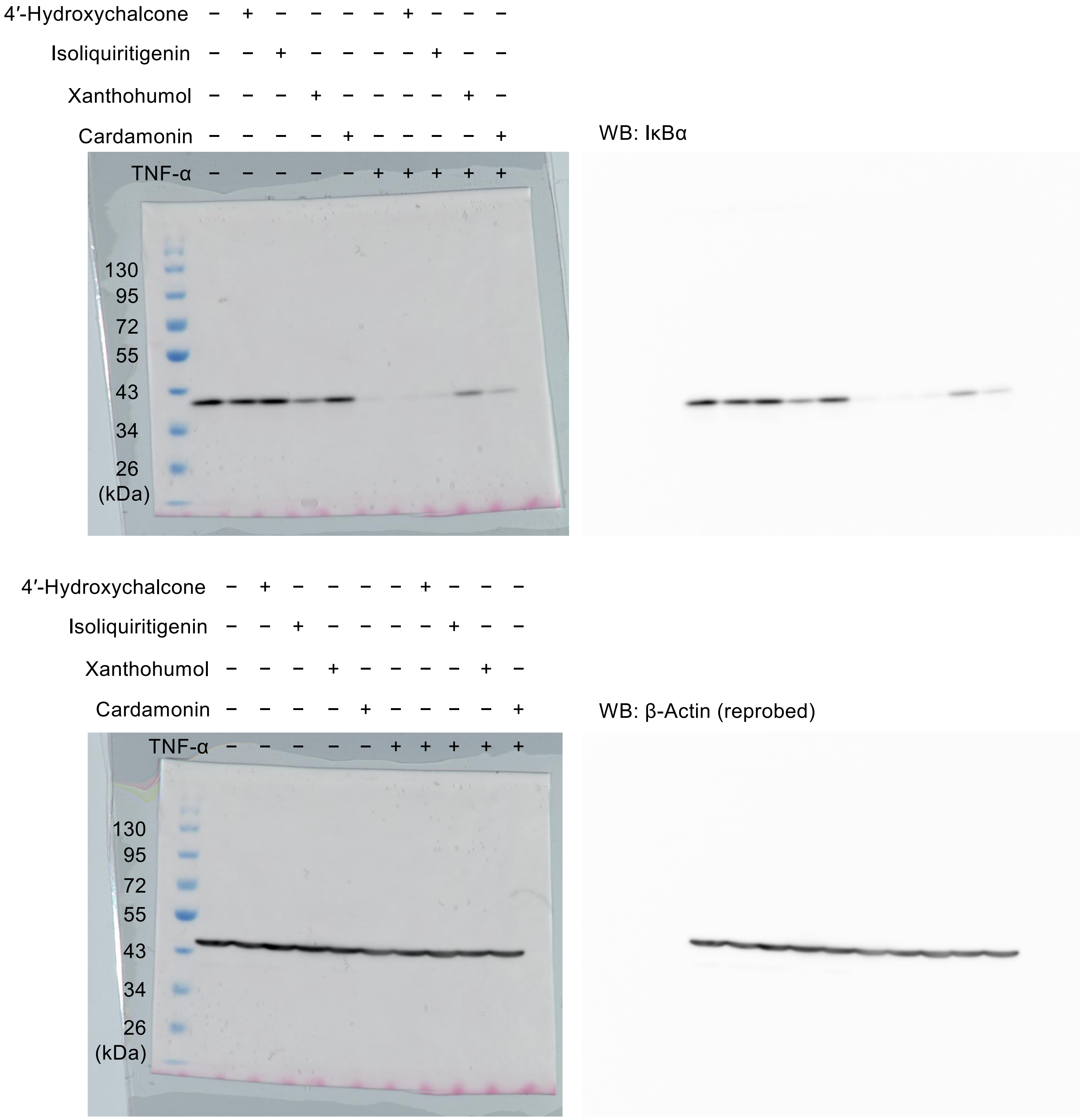

Figure S44: Original blots in Figure 11A (nucleus)

4′-Hydroxychalcone (μM)

0 0 5 10 25 50

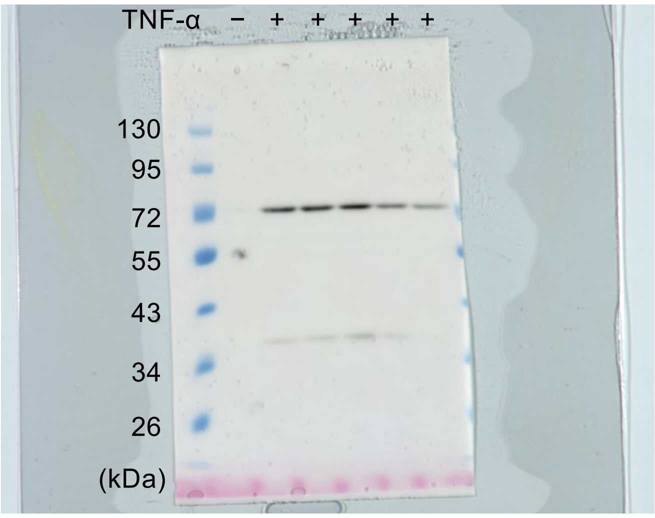

WB: RelA

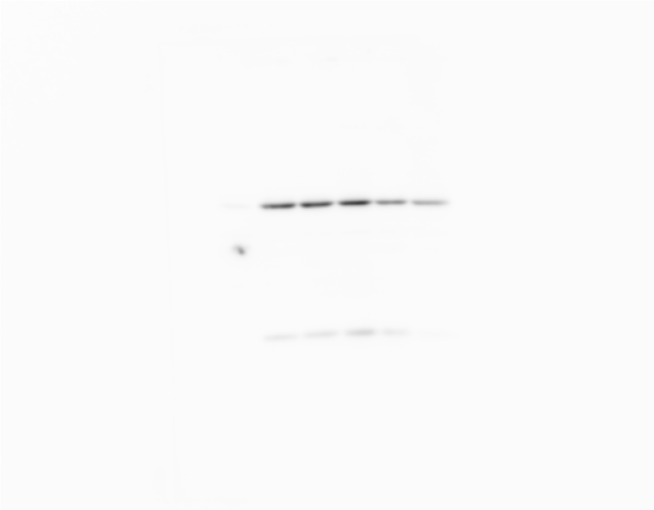

4′-Hydroxychalcone (μM)

0 0 5 10 25 50

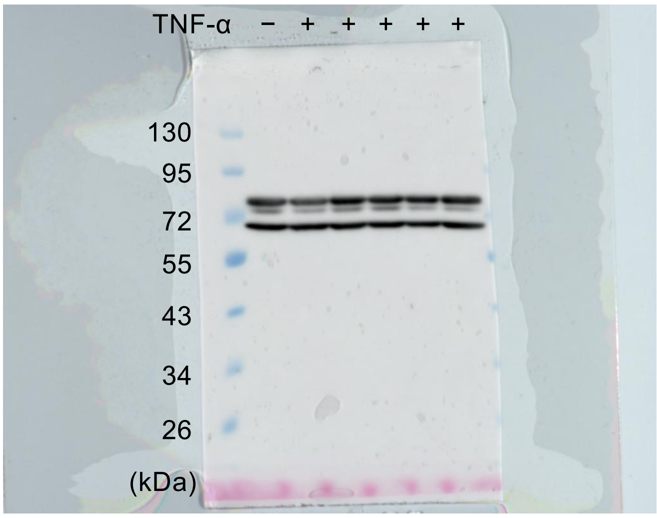

WB: Lamin A/C (reprobed)

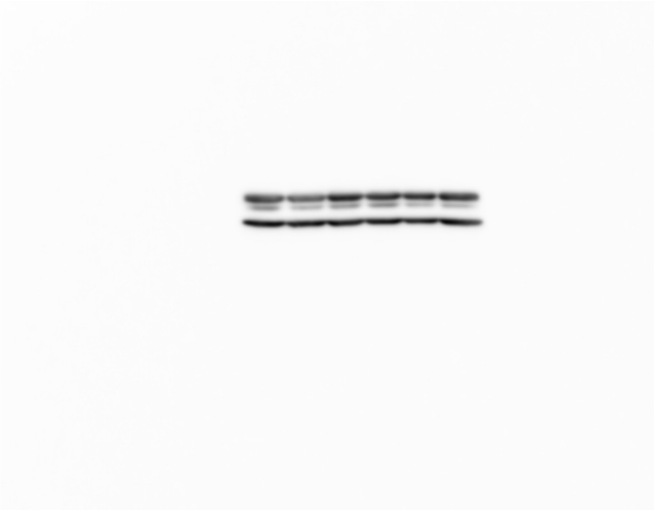

Figure S45: Original blots in Figure 11A (cytoplasm)

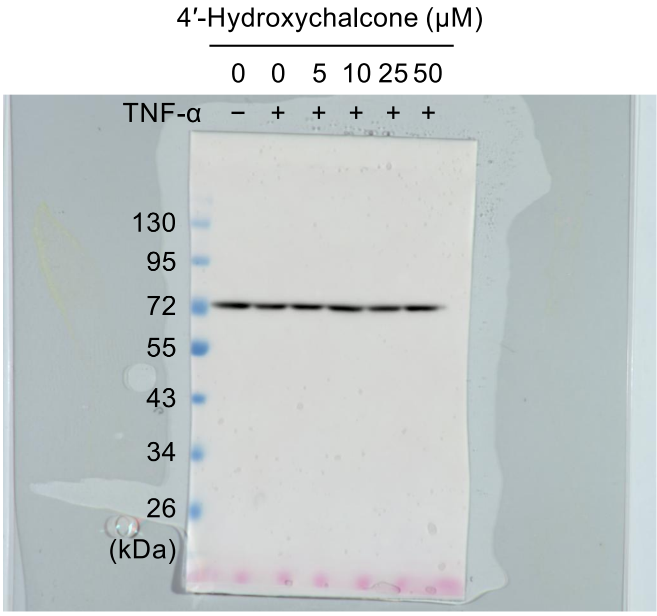

WB: RelA

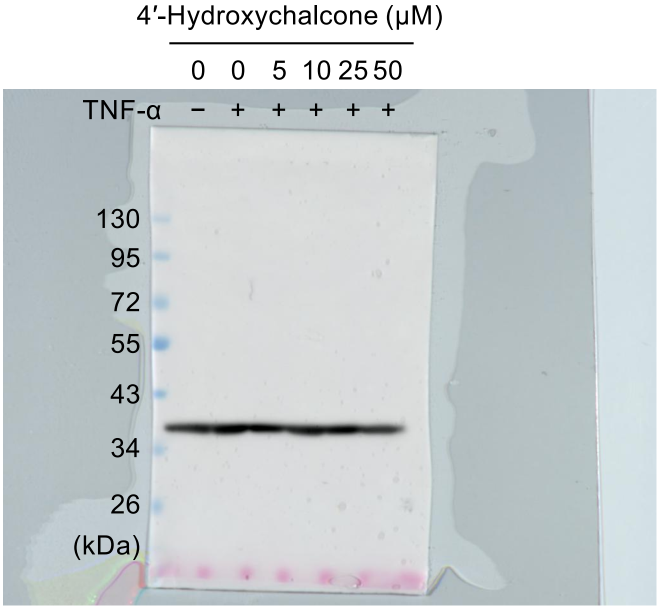

WB: GAPDH (reprobed)

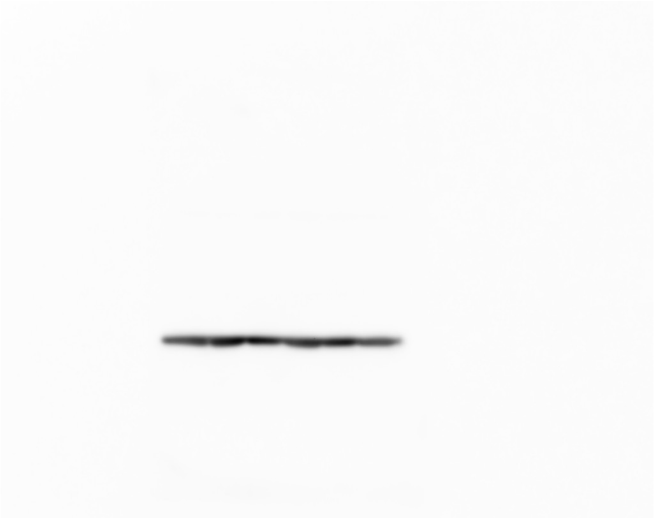

Figure S46: Original blots (1) in Figure 11B

4′-Hydroxychalcone (μM)

0 0 5 10 25 50

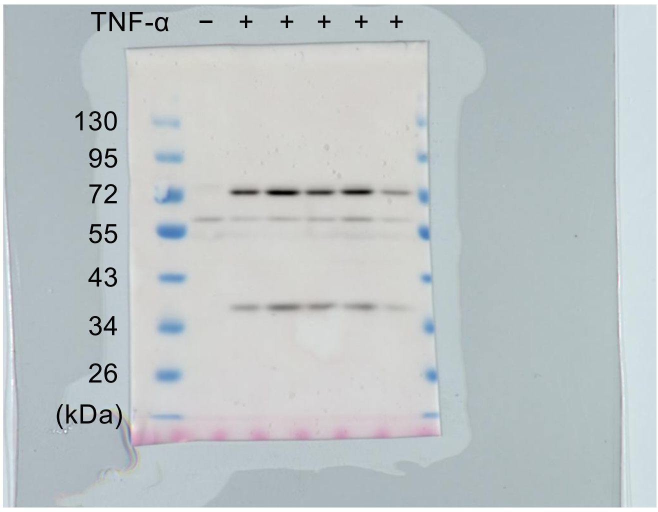

WB: RelA

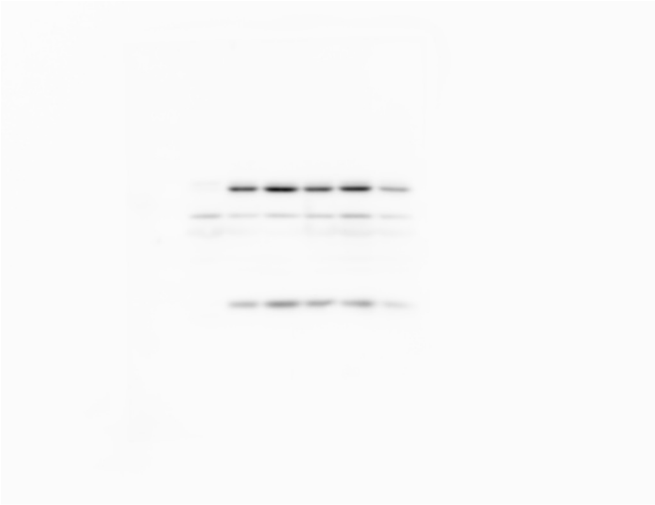

4′-Hydroxychalcone (μM)

0 0 5 10 25 50

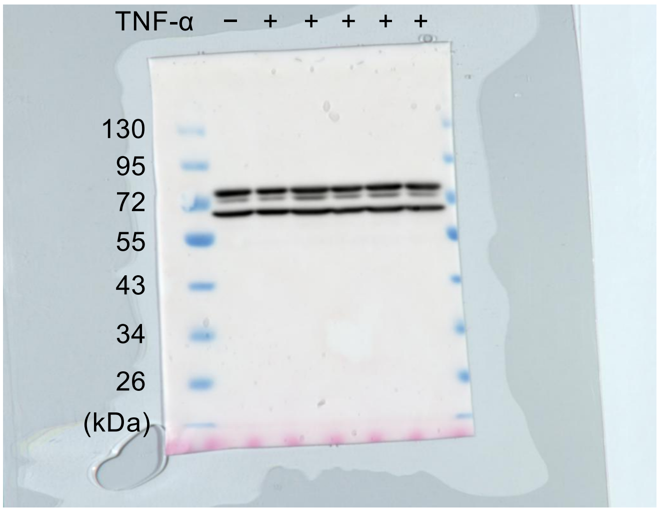

WB: Lamin A/C (reprobed)

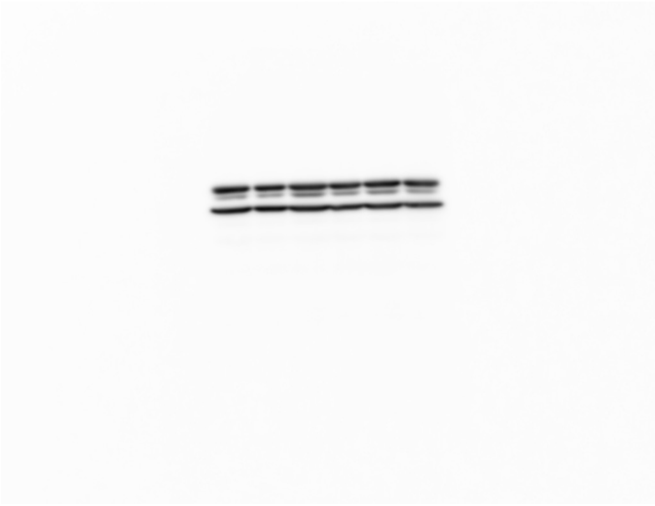

Figure S47: Original blots (2) in Figure 11B

4′-Hydroxychalcone (μM)

0 0 5 10 25 50

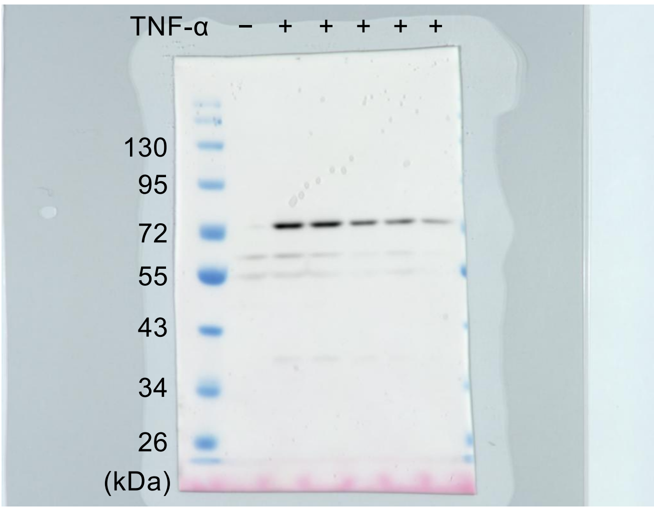

WB: RelA

4′-Hydroxychalcone (μM)

0 0 5 10 25 50

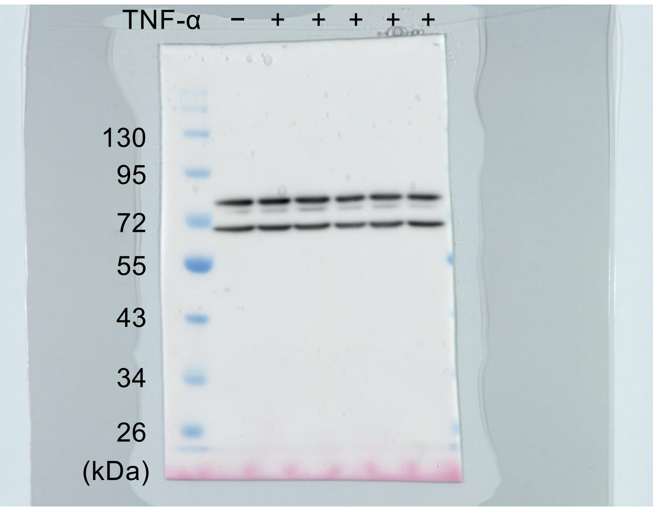

WB: Lamin A/C (reprobed)

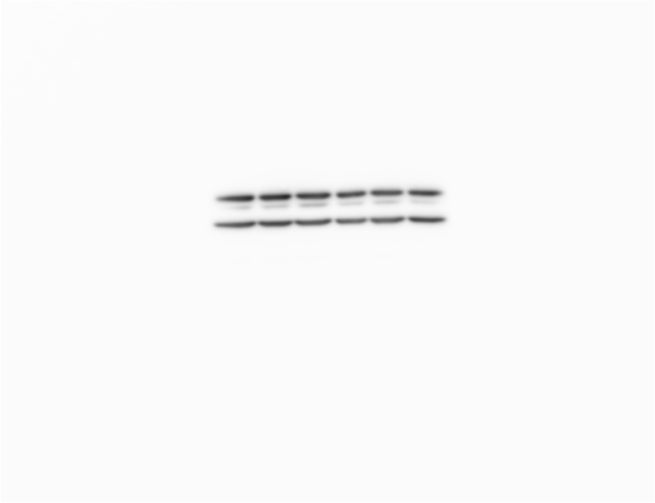

Figure S48: Original blots (3) in Figure 11B

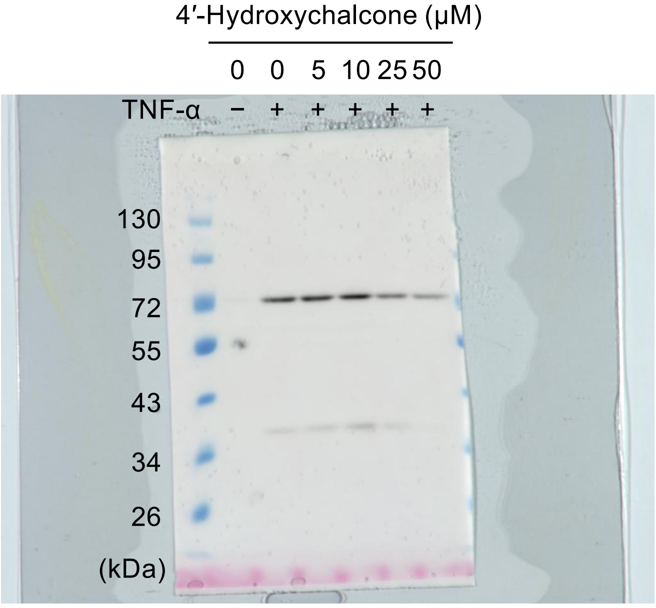

WB: RelA

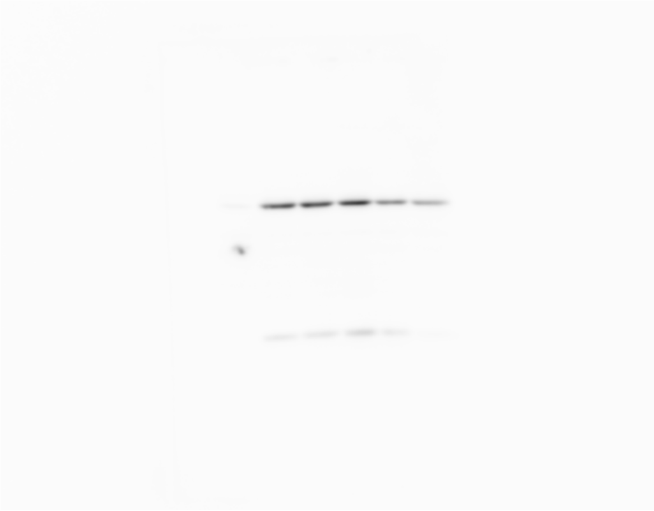

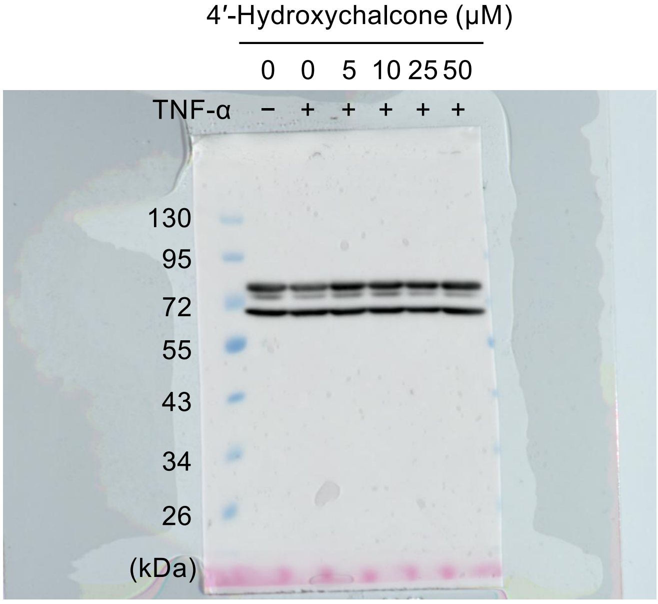

WB: Lamin A/C (reprobed)

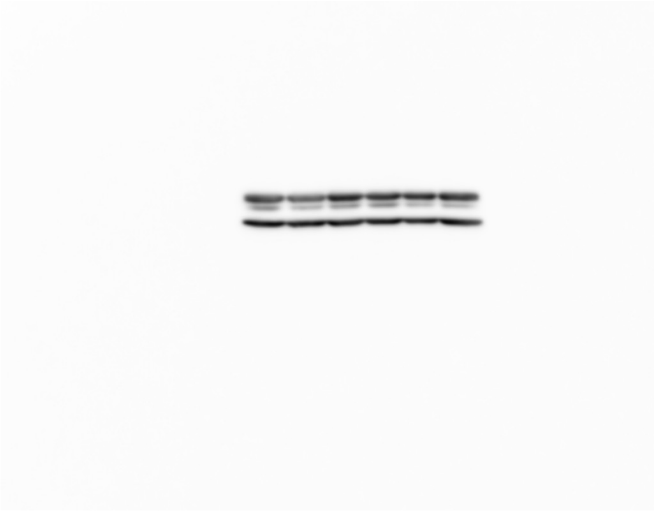

Figure S49: Original blots (1) in Figure 11C

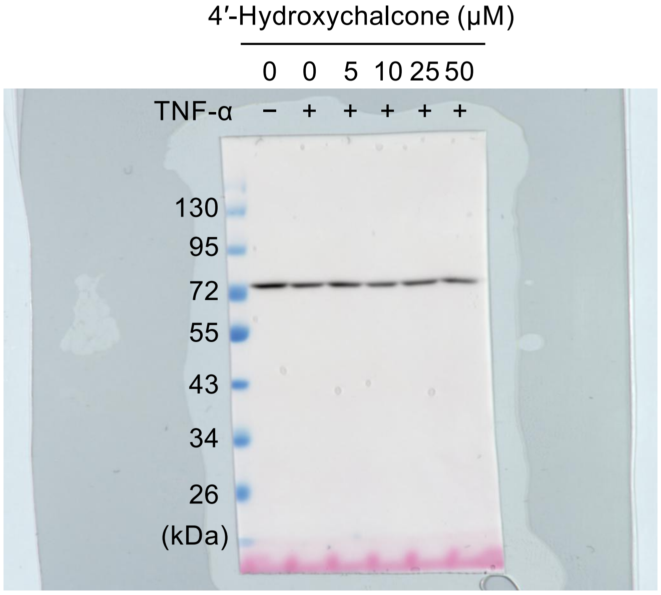

WB: RelA

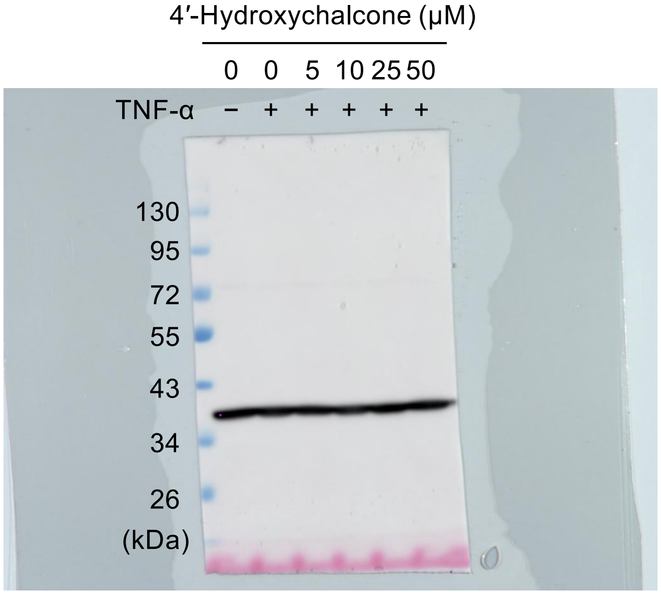

WB: GAPDH (reprobed)

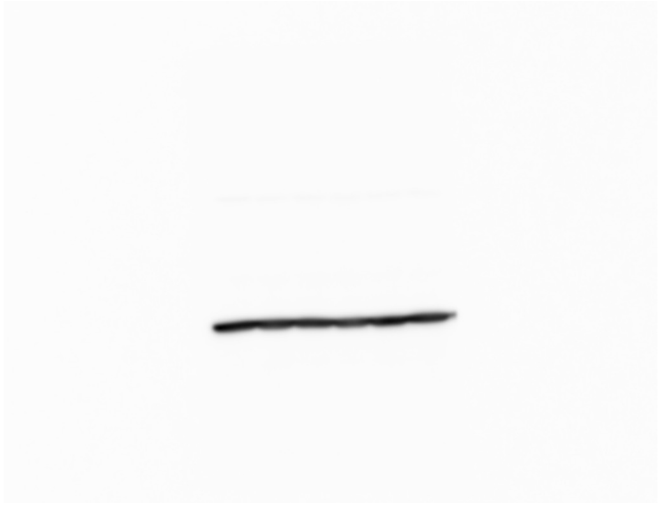

Figure S50: Original blots (2) in Figure 11C

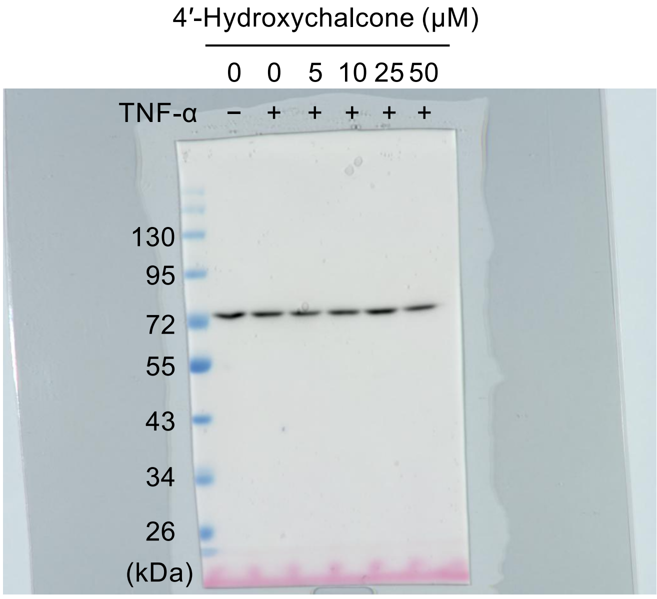

WB: RelA

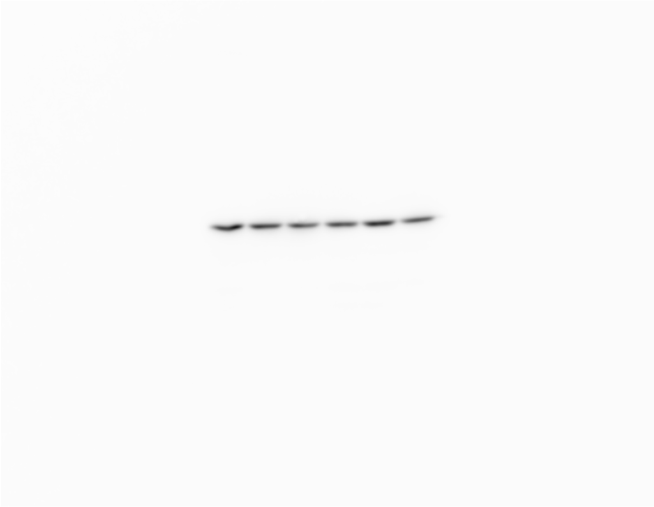

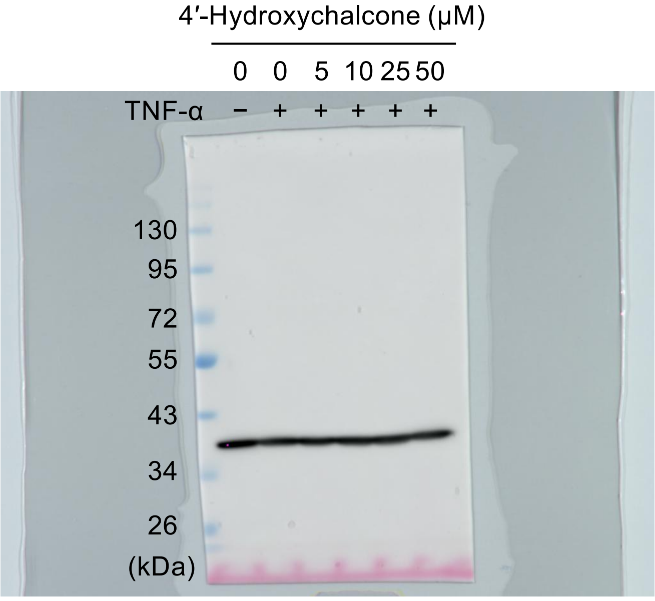

WB: GAPDH (reprobed)

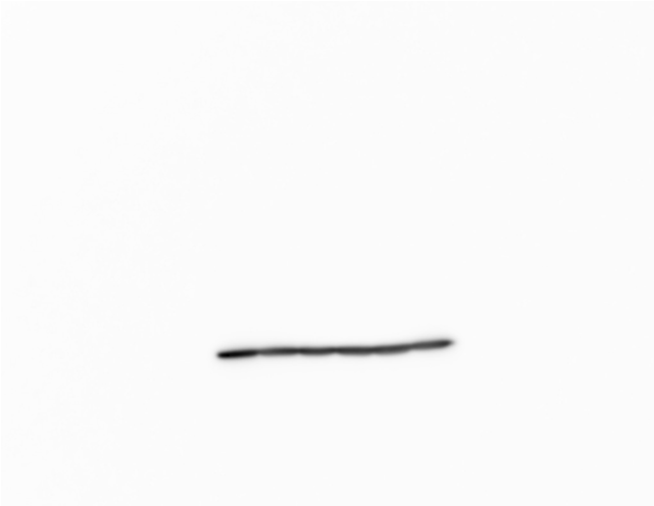

Figure S51: Original blots (3) in Figure 11C

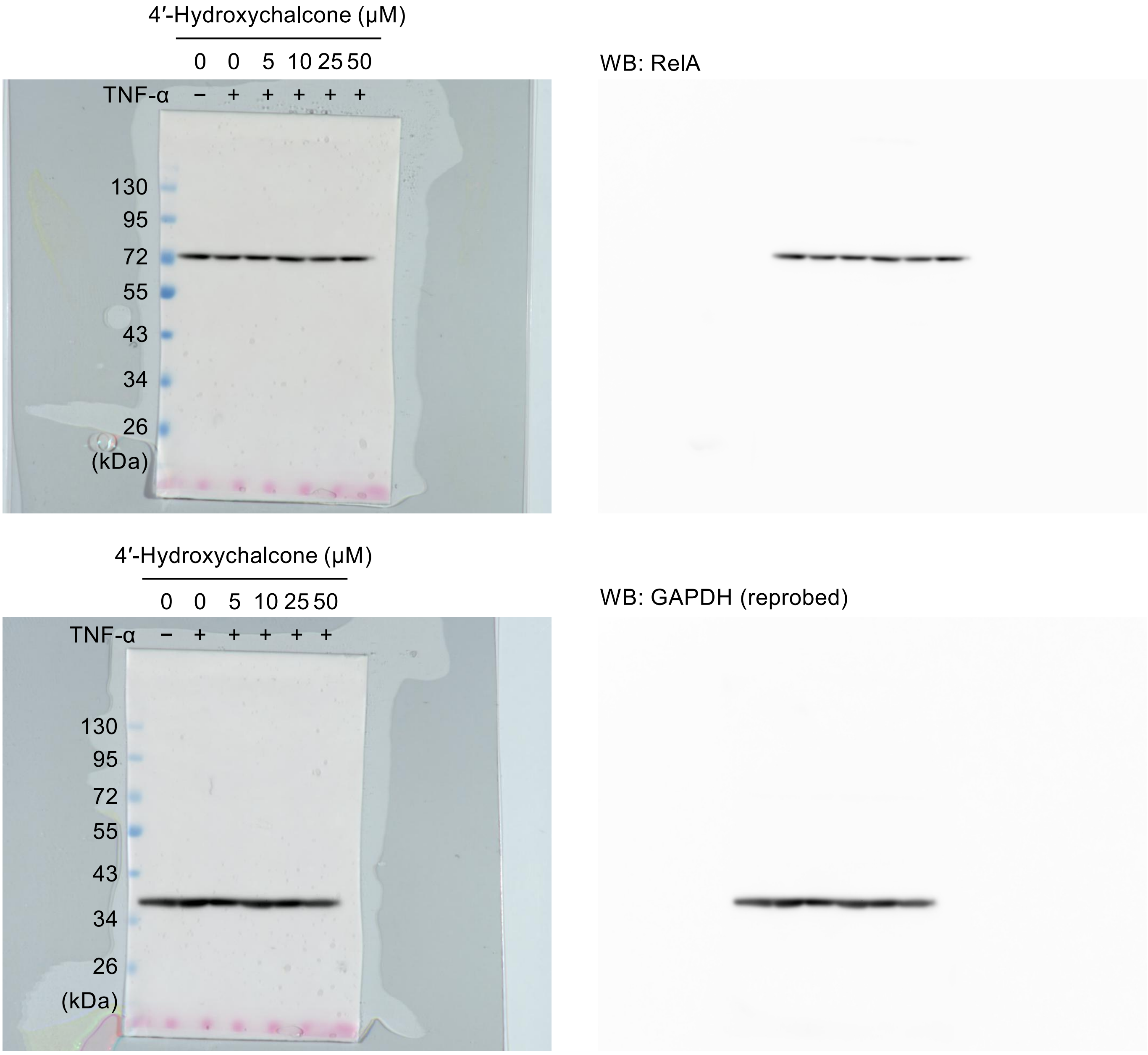

Figure S52: Original blots in Figure 11D (nucleus)

Isoliquiritigenin (µM)

0 0 5 10 25 50

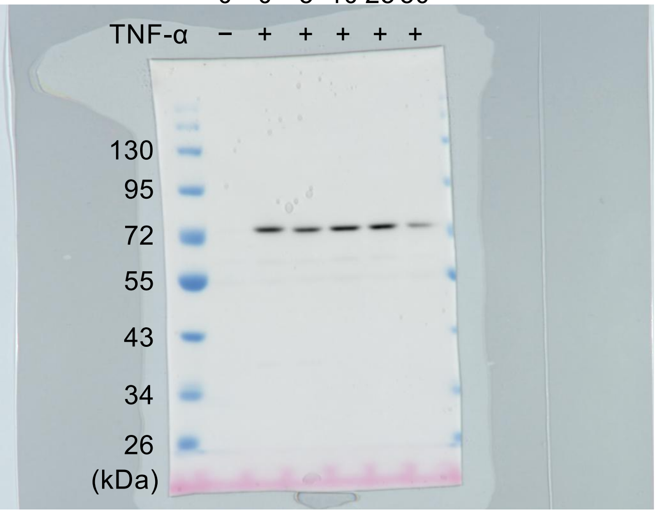

WB: RelA

Isoliquiritigenin (µM)

0 0 5 10 25 50

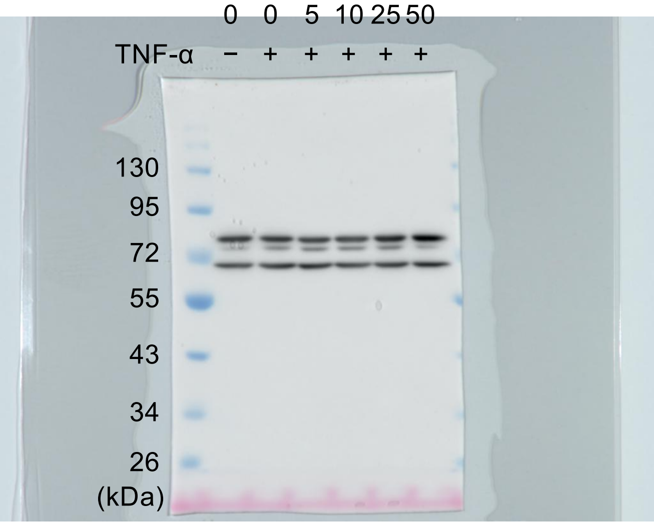

WB: Lamin A/C (reprobed)

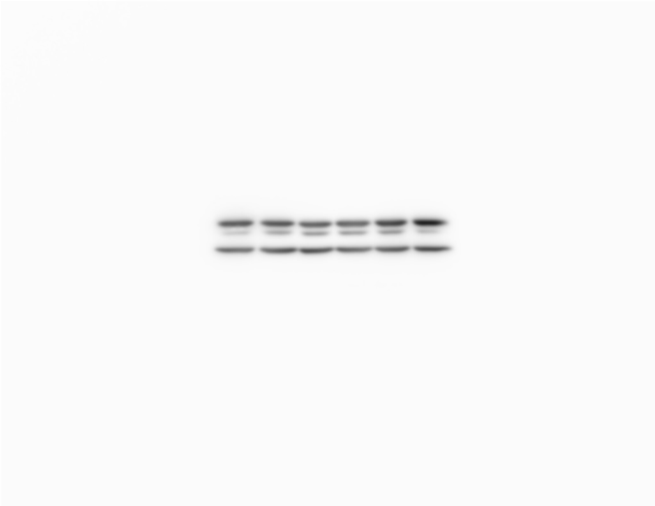

Figure S53: Original blots in Figure 11D (cytoplasm)

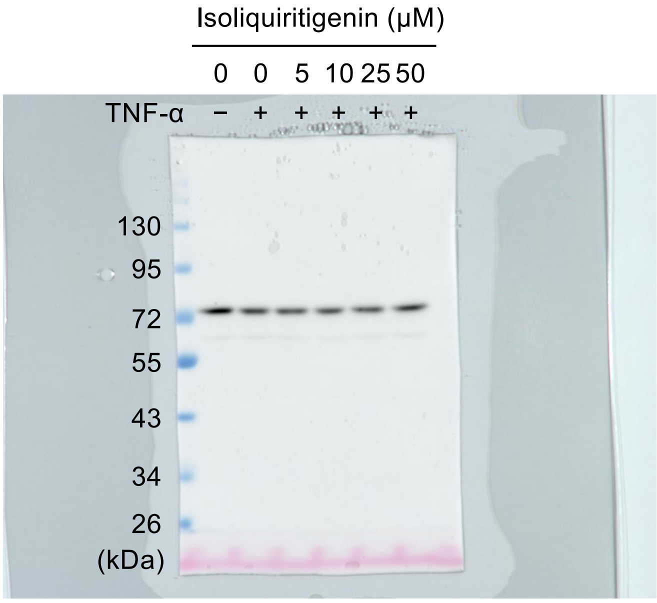

WB: RelA

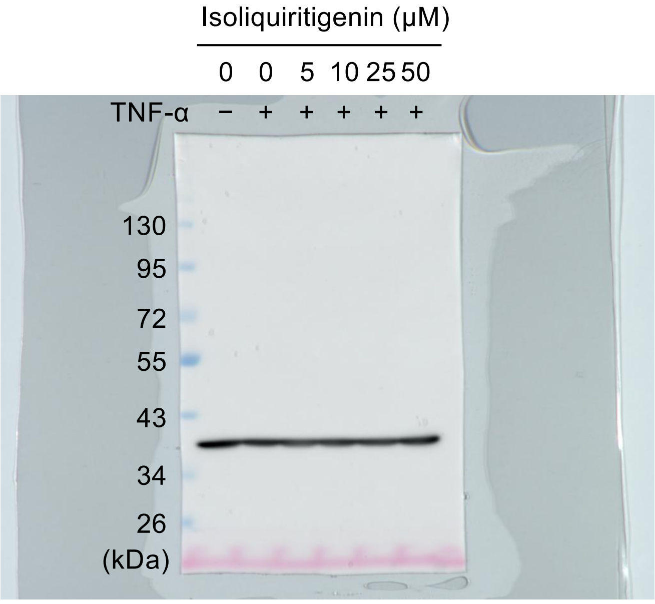

WB: GAPDH (reprobed)

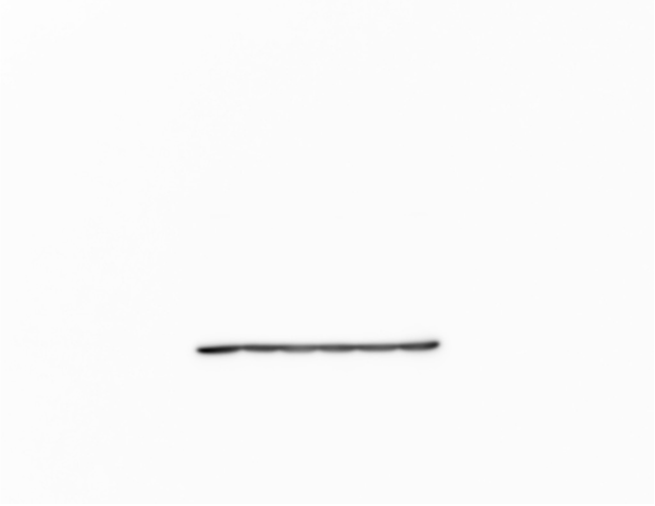

Figure S54: Original blots (1) in Figure 11E

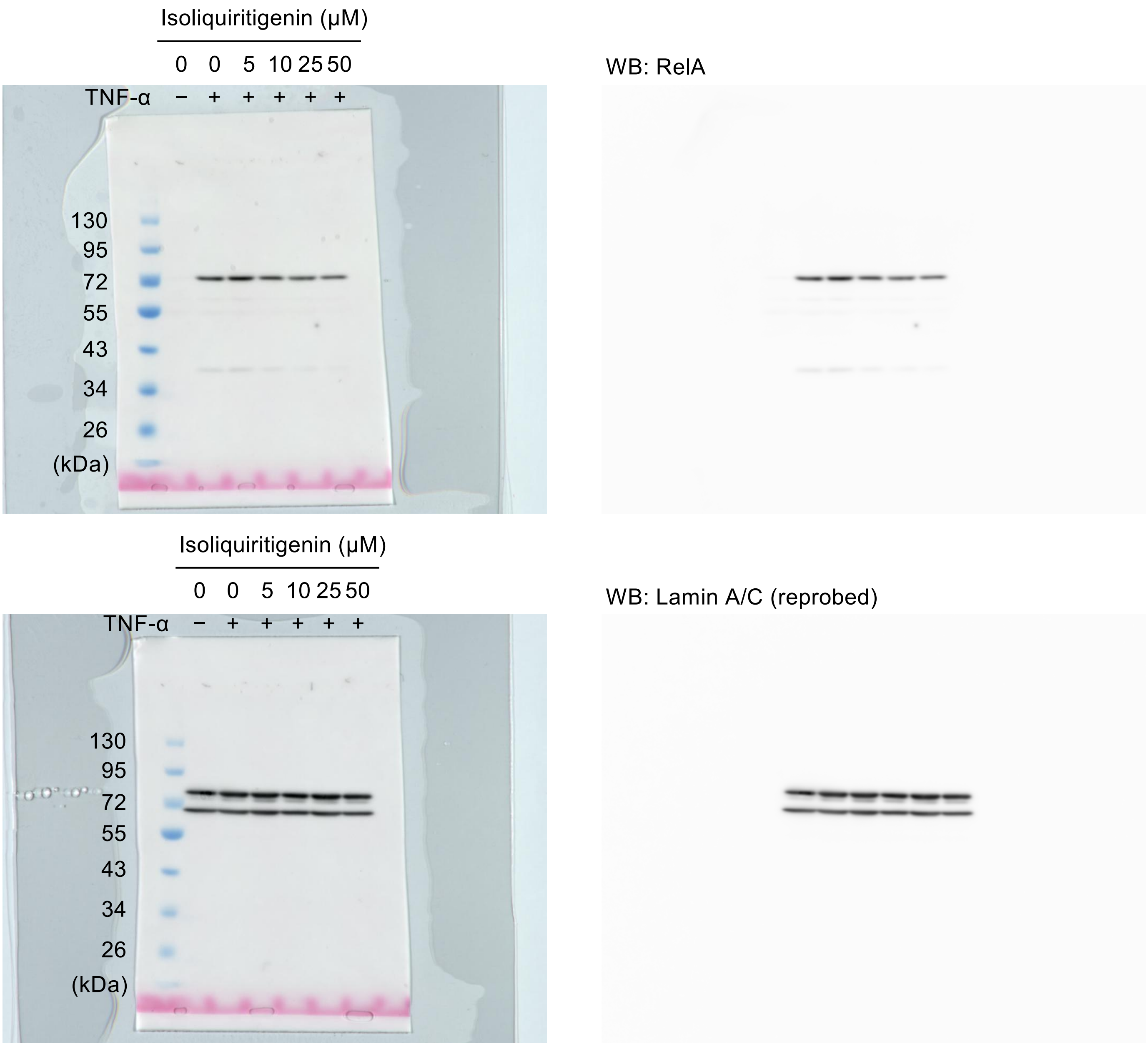

Figure S55: Original blots (2) in Figure 11E

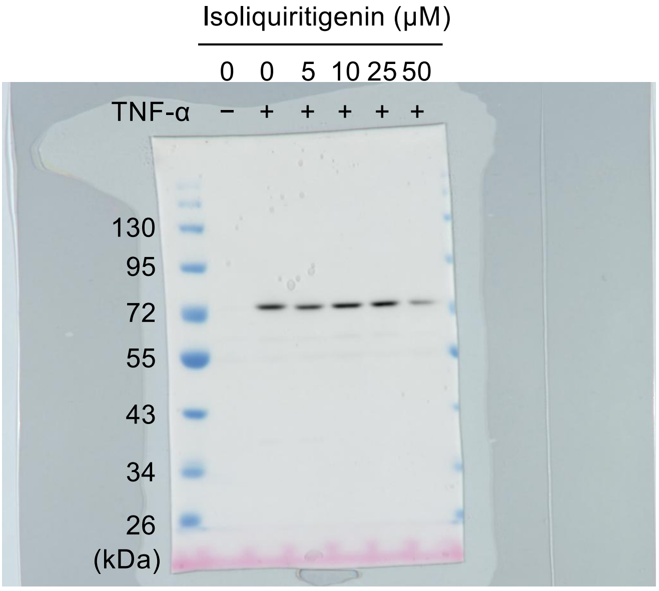

WB: RelA

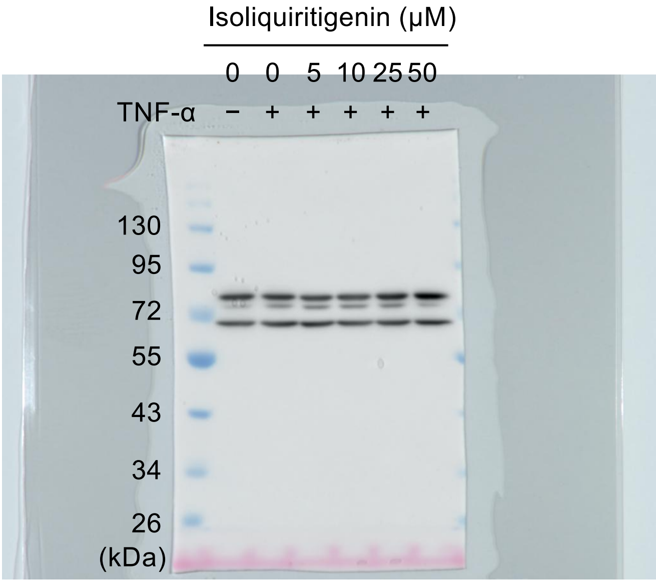

WB: Lamin A/C (reprobed)

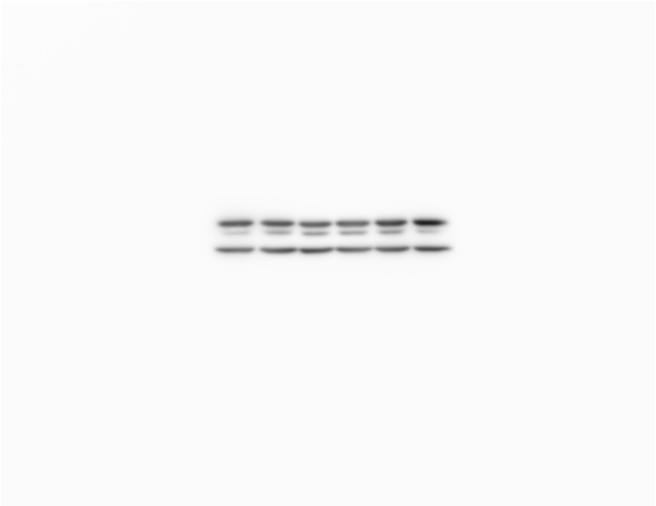

Figure S56: Original blots (3) in Figure 11E

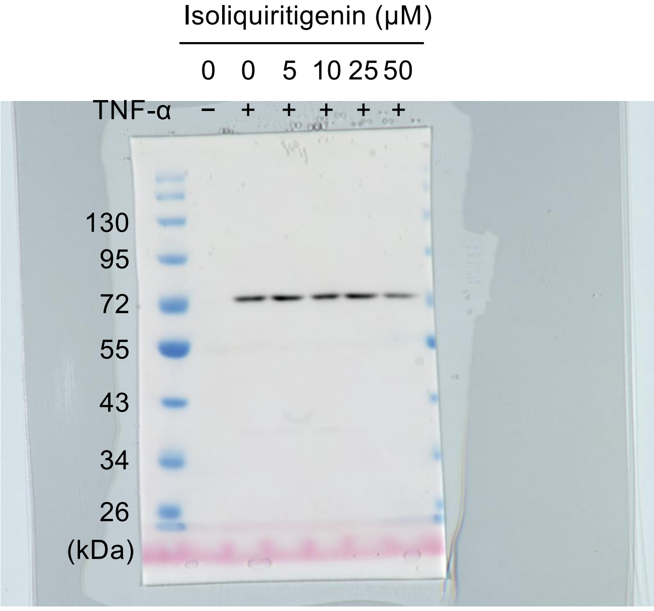

WB: RelA

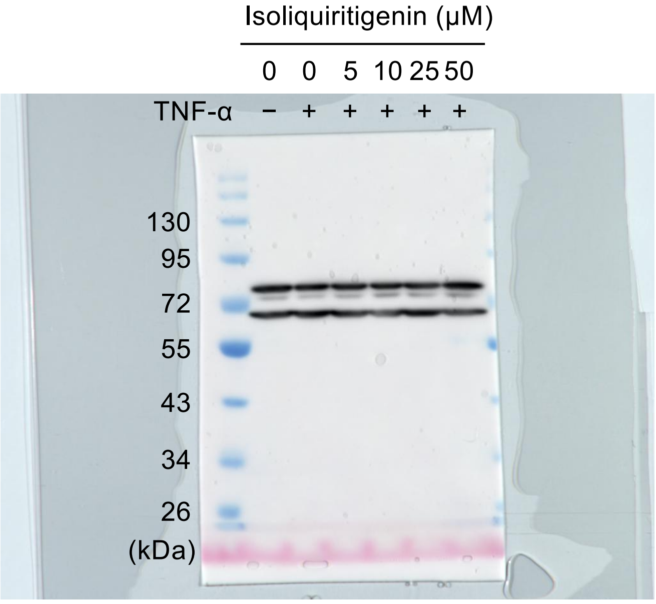

WB: Lamin A/C (reprobed)

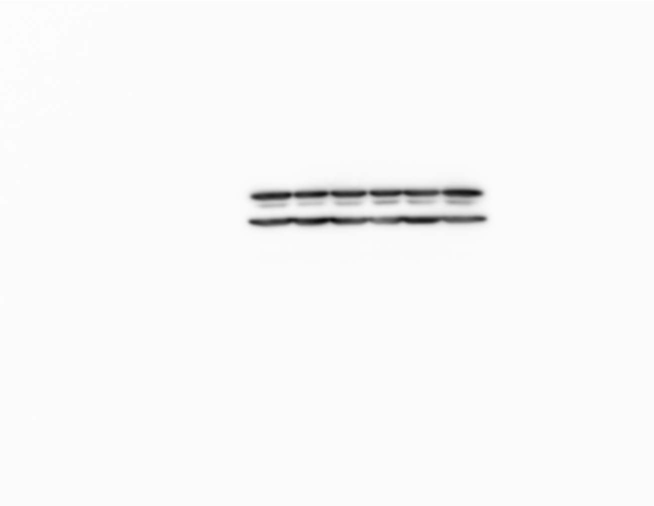

Figure S57: Original blots (1) in Figure 11F

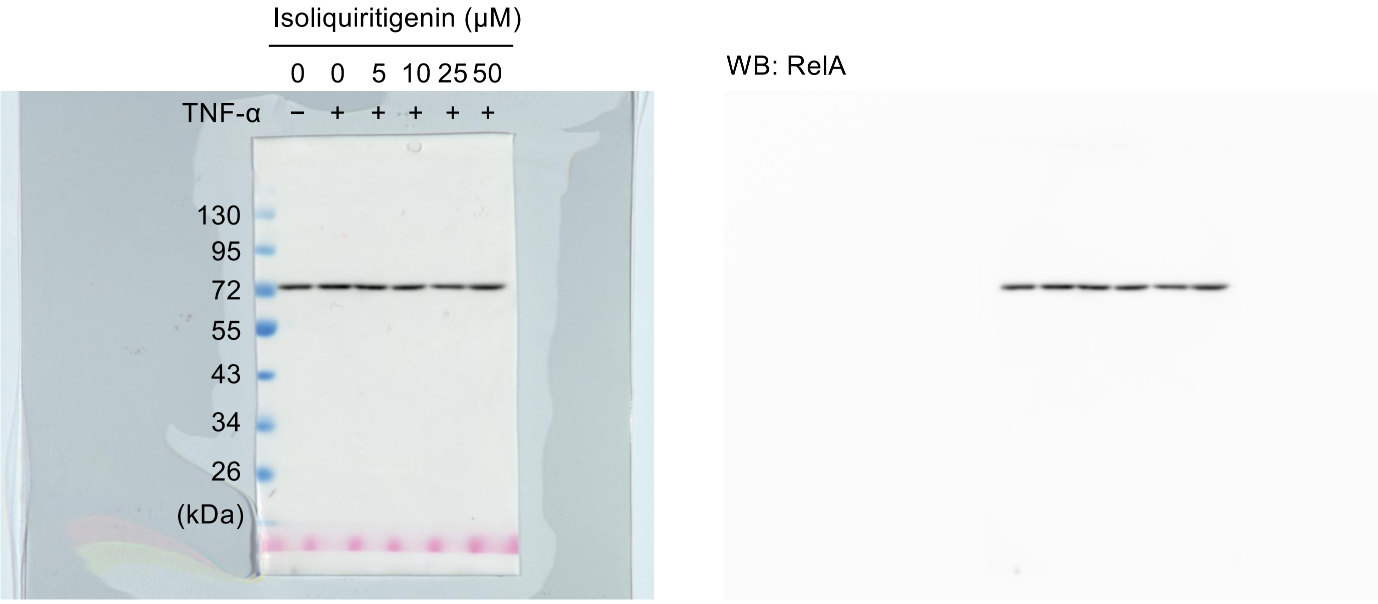

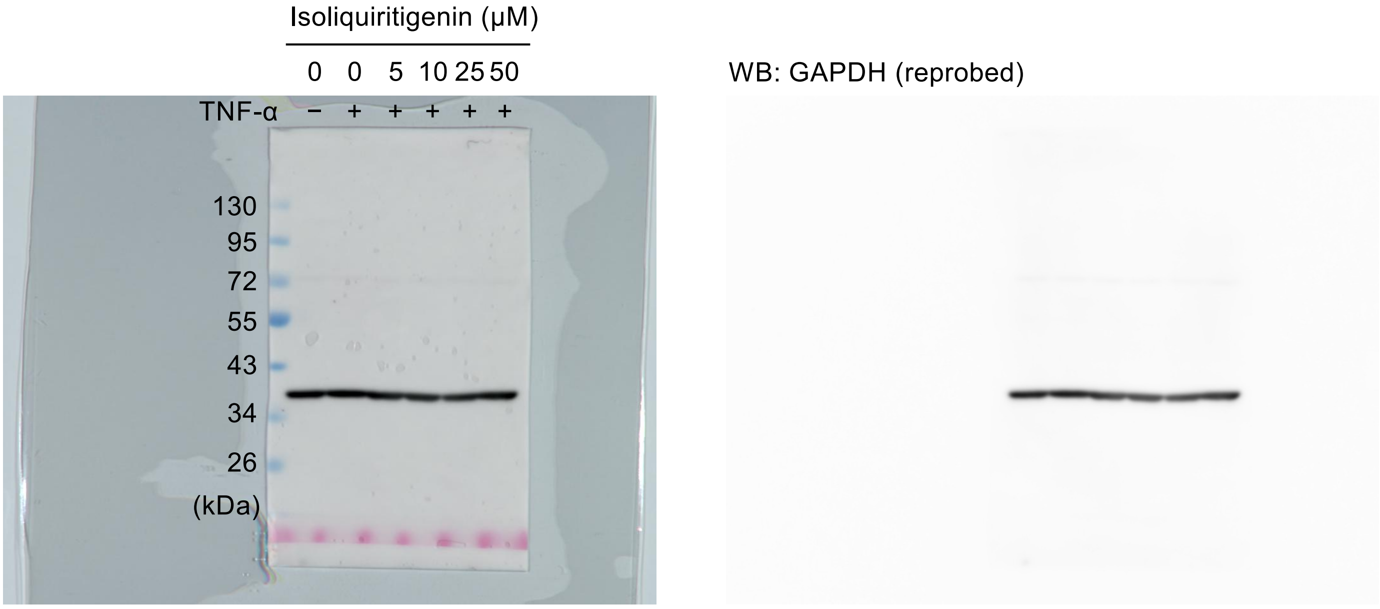

Figure S58: Original blots (2) in Figure 11F

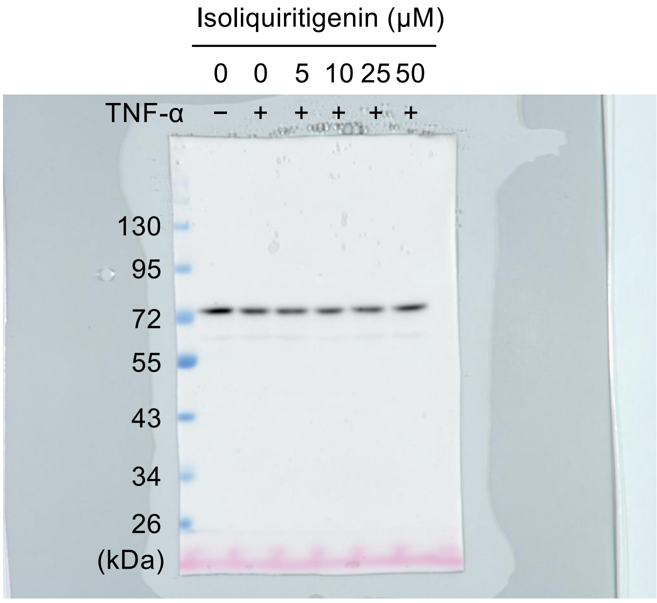

WB: RelA

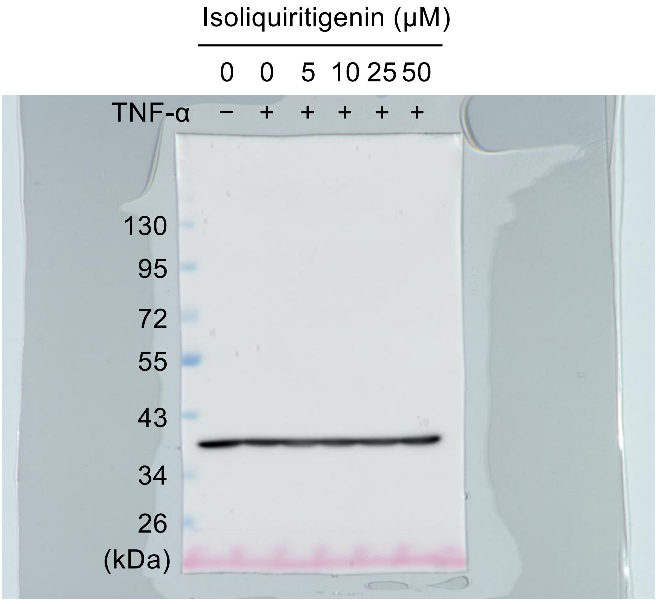

WB: GAPDH (reprobed)

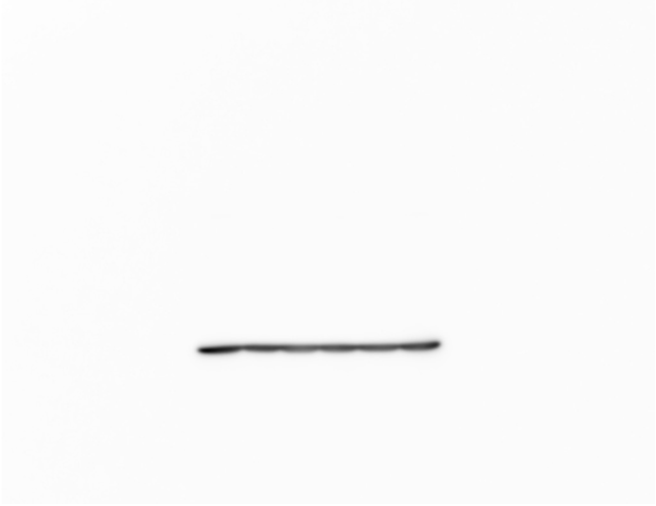

Figure S59: Original blots (3) in Figure 11F

Isoliquiritigenin (μM)

0 0 5 10 25 50

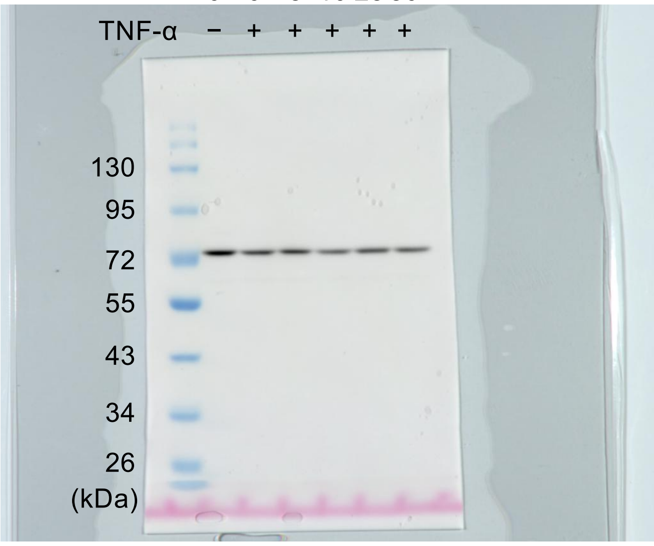

WB: RelA

Isoliquiritigenin (μM)

0 0 5 10 25 50

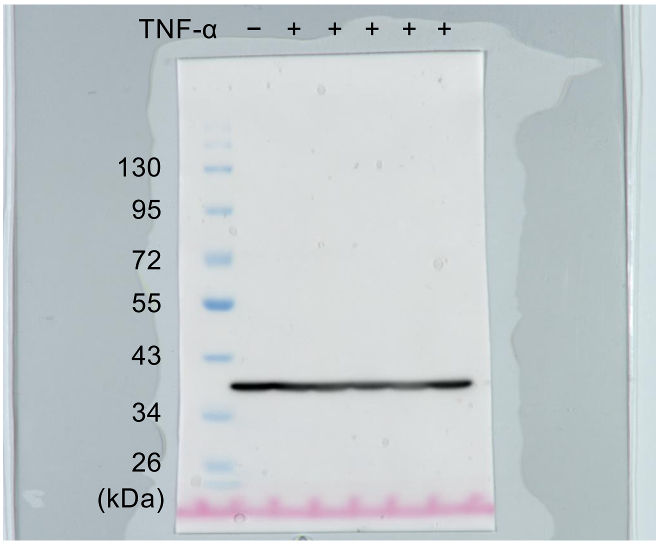

WB: GAPDH (reprobed)

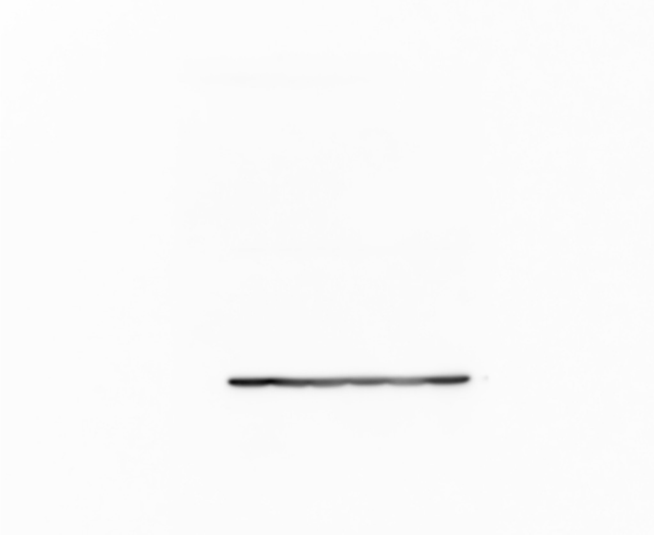

Figure S60: Original blots in Figure 11G (nucleus)

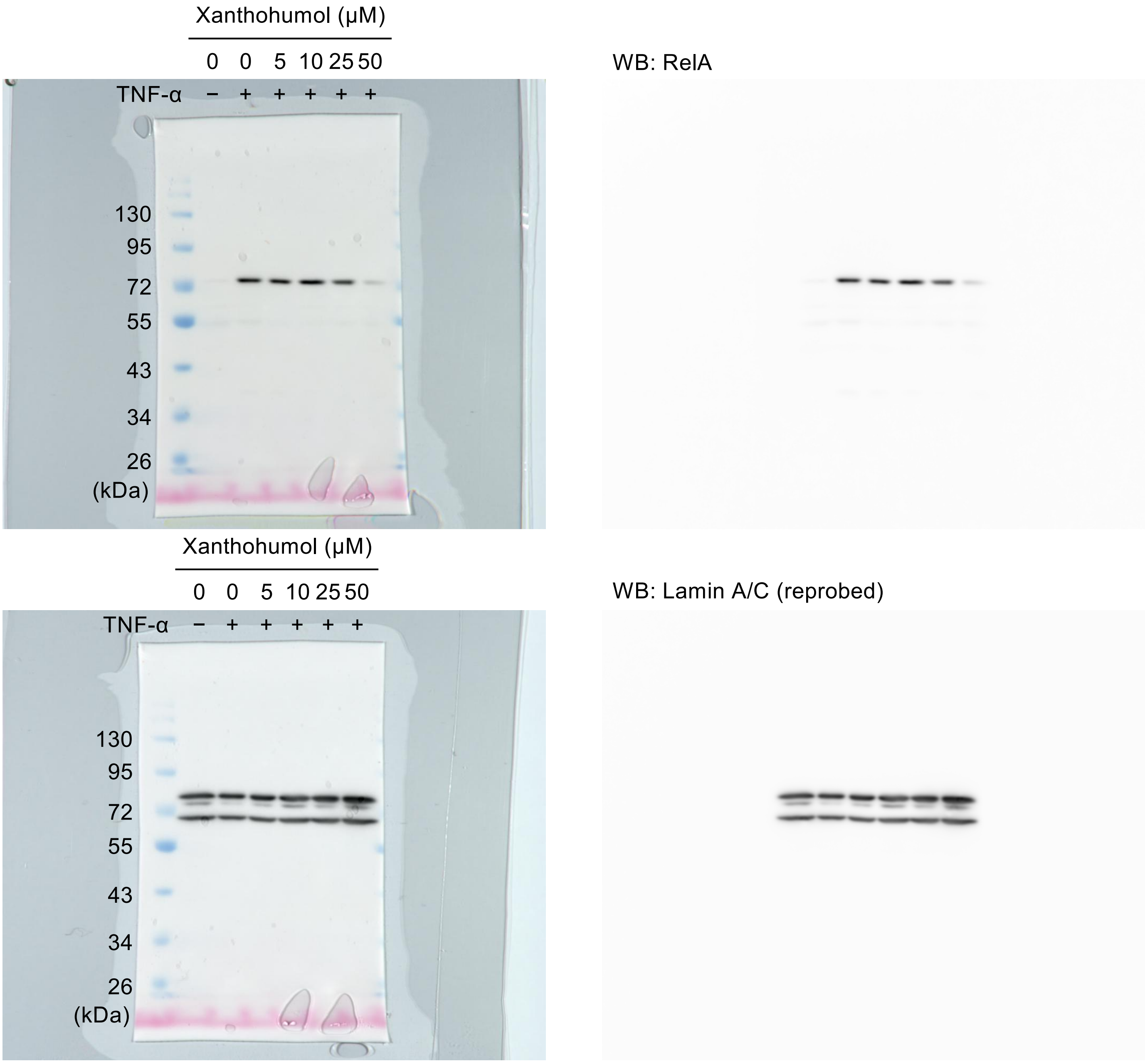

Figure S61: Original blots in Figure 11G (cytoplasm)

Xanthohumol (μM)

0 0 5 10 25 50

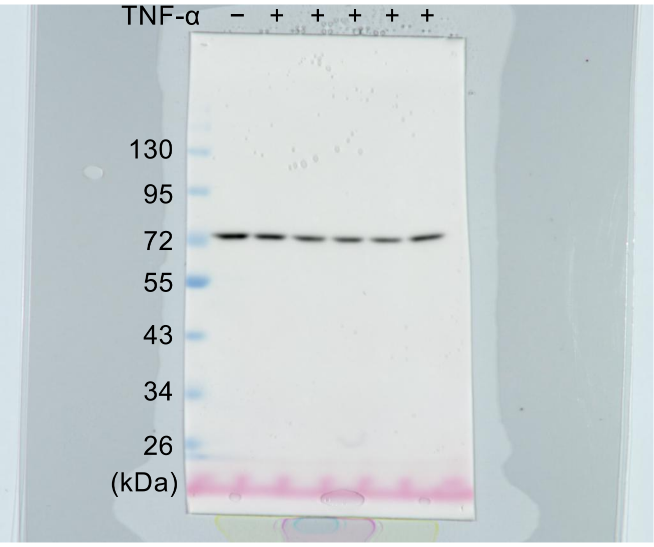

WB: RelA

Xanthohumol (μM)

0 0 5 10 25 50

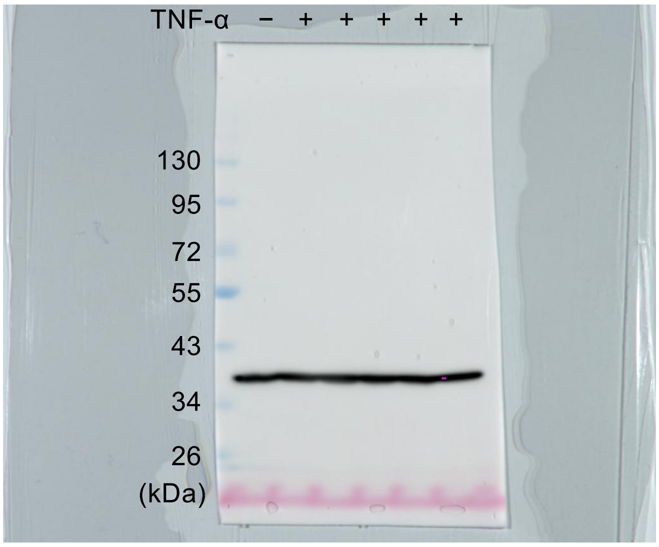

WB: GAPDH (reprobed)

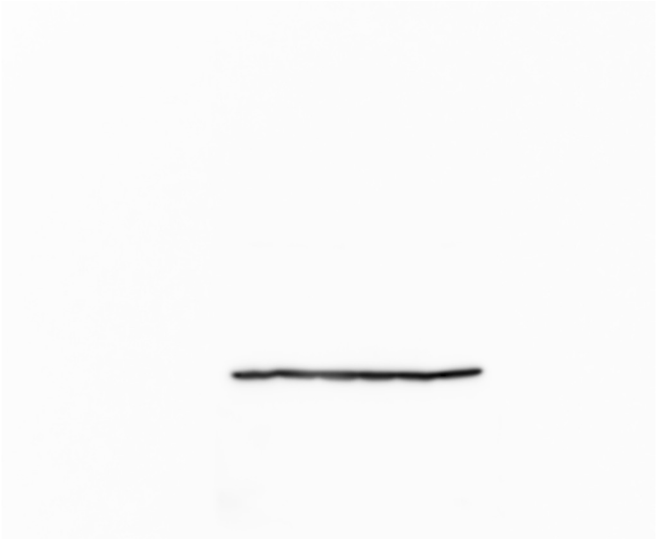

Figure S62: Original blots (1) in Figure 11H

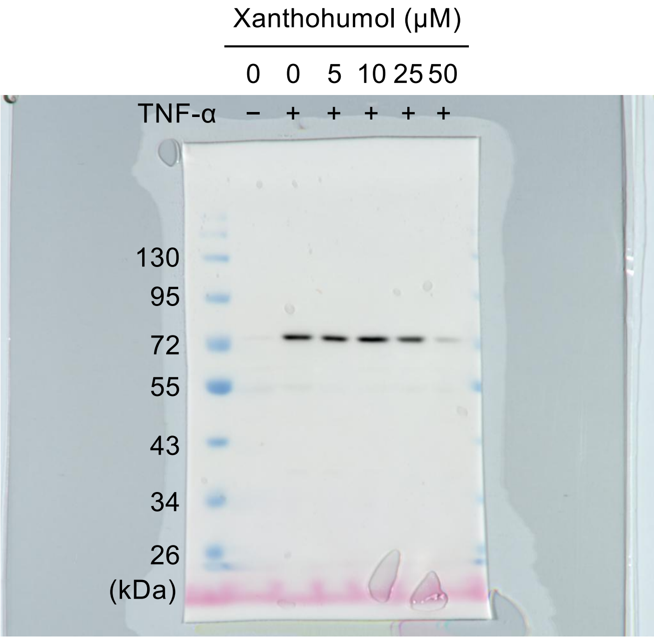

WB: RelA

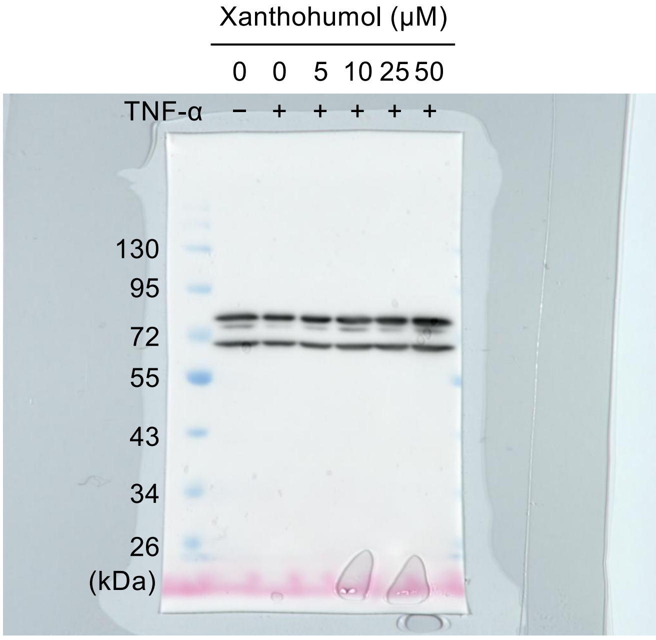

WB: Lamin A/C (reprobed)

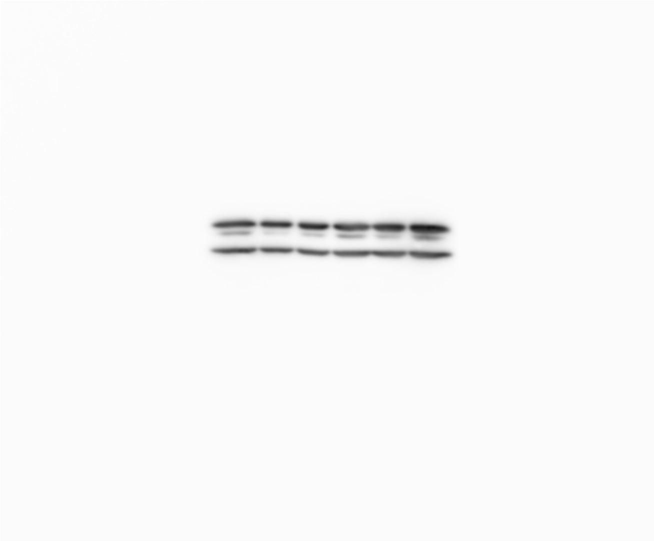

Figure S63: Original blots (2) in Figure 11H

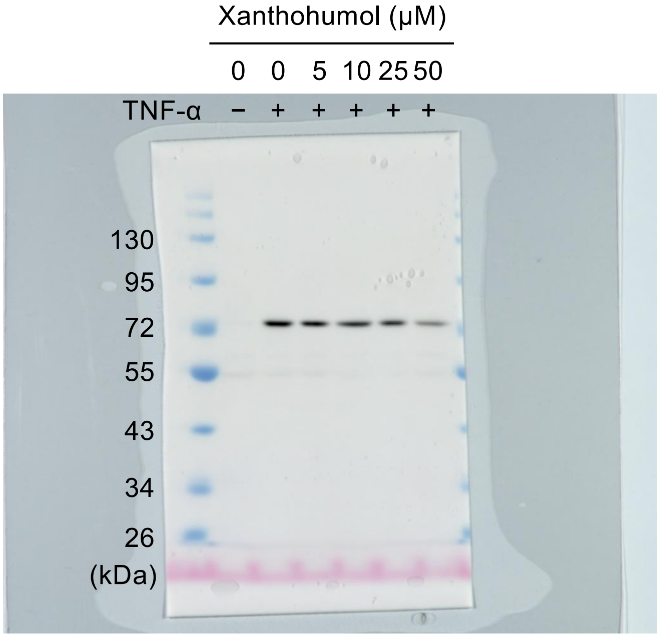

WB: RelA

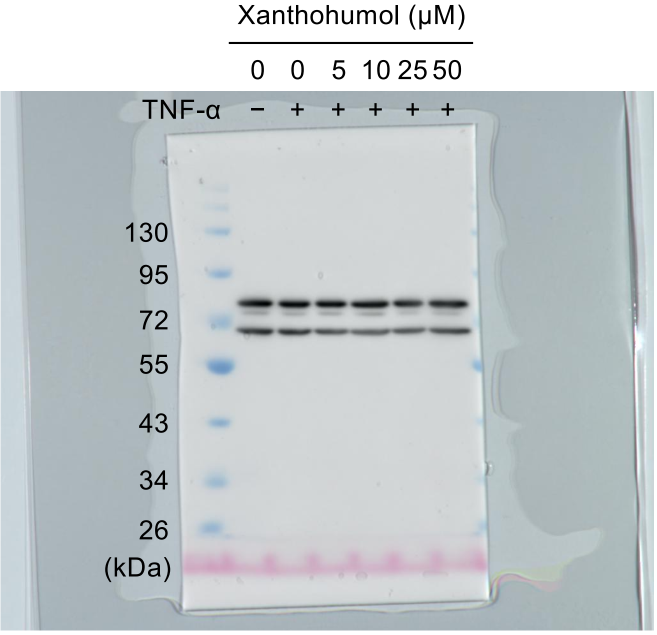

WB: Lamin A/C (reprobed)

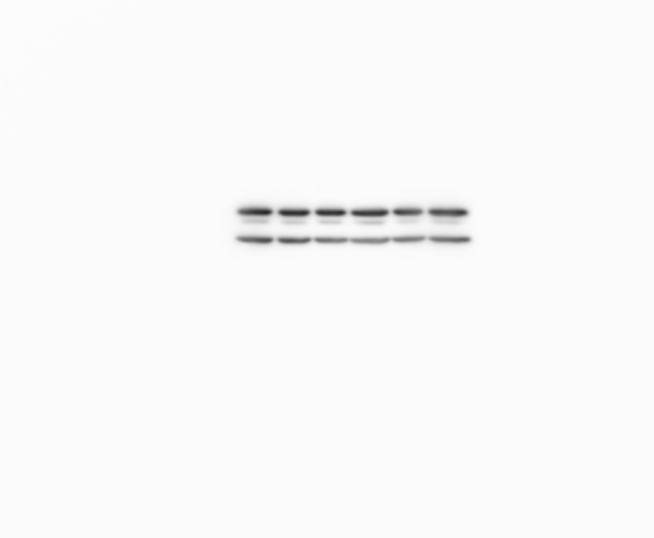

Figure S64: Original blots (3) in Figure 11H

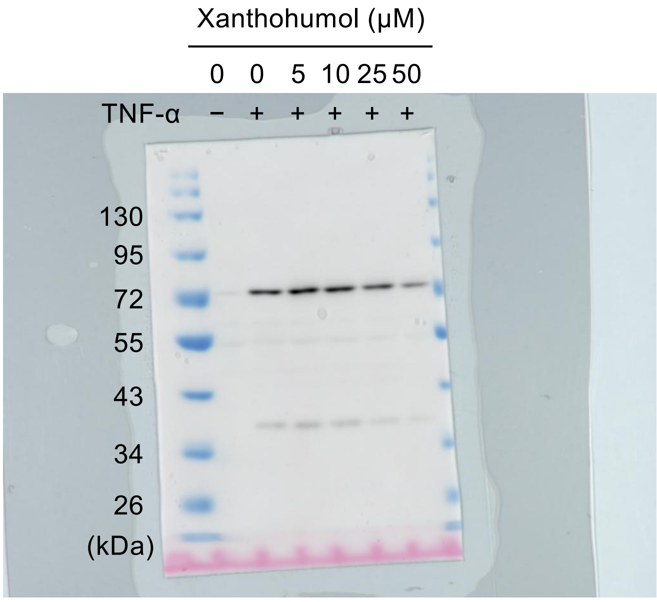

WB: RelA

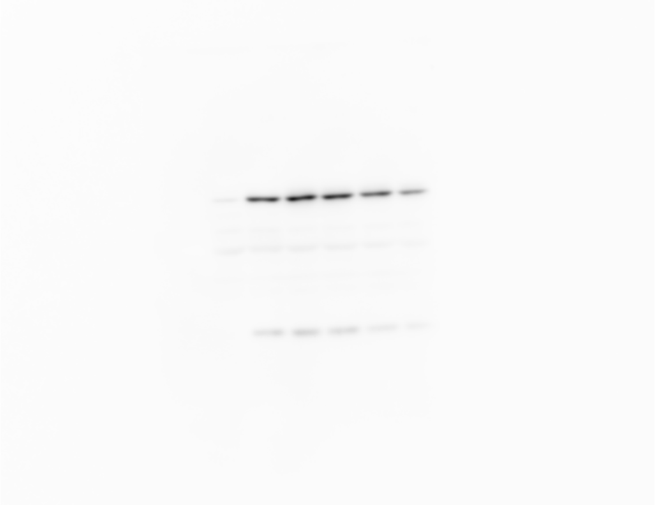

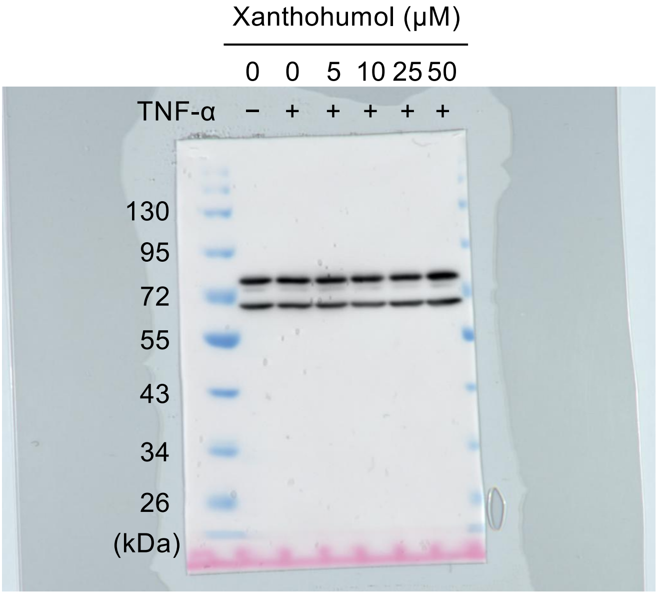

WB: Lamin A/C (reprobed)

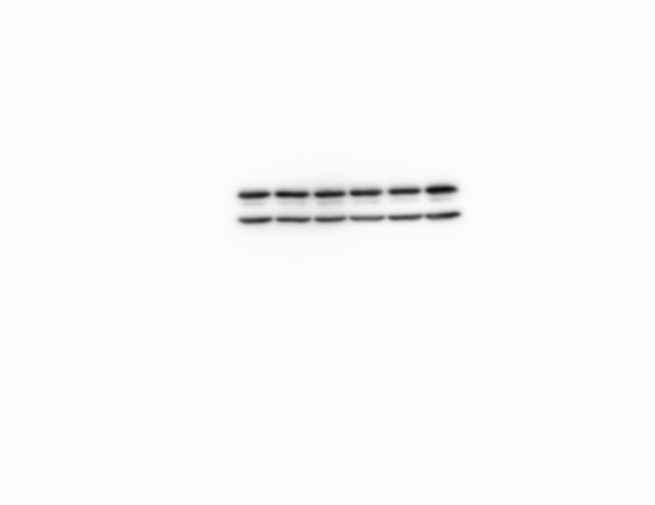

Figure S65: Original blots (1) in Figure 11I

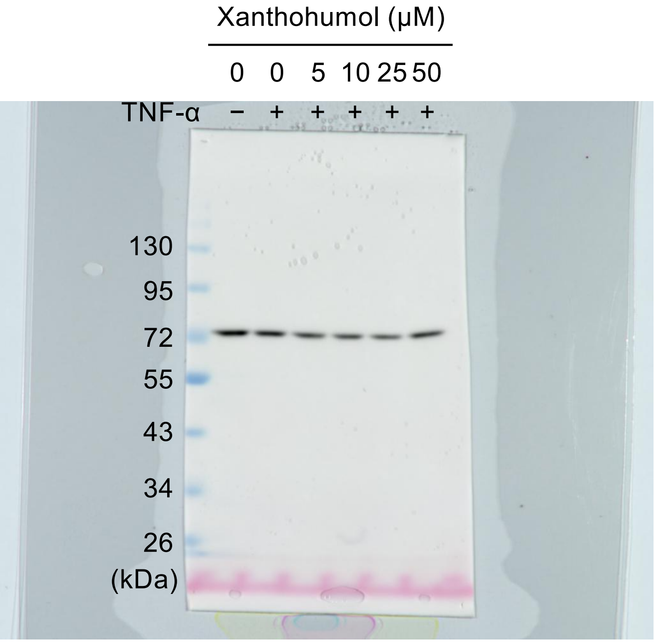

WB: RelA

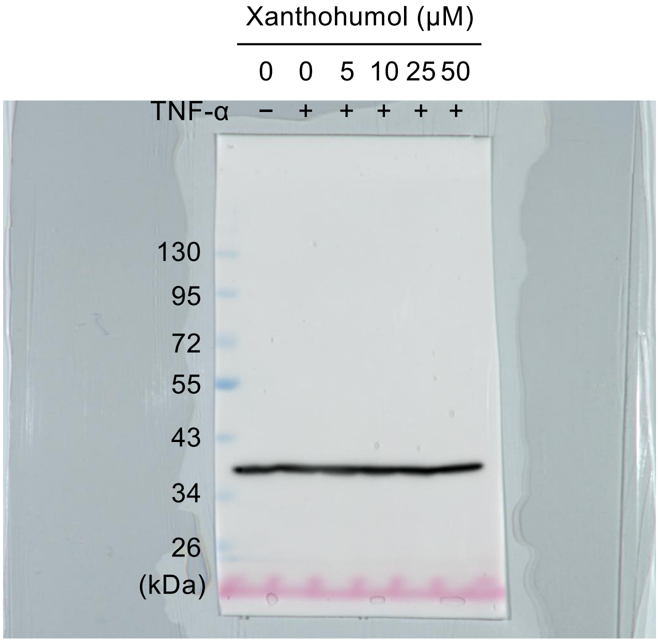

WB: GAPDH (reprobed)

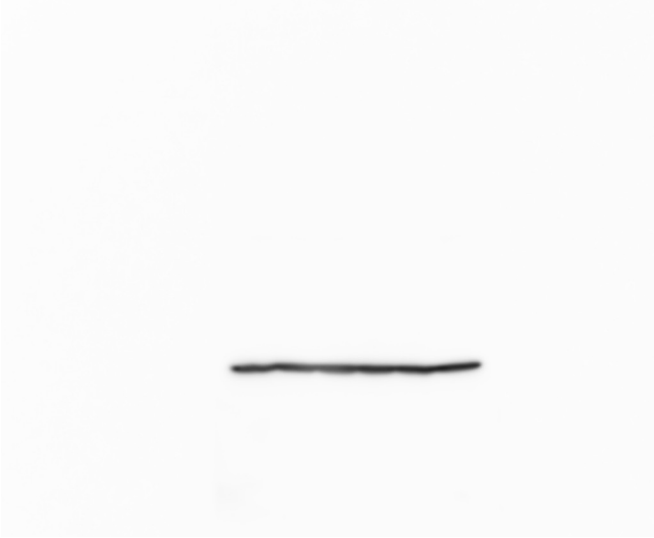

Figure S66: Original blots (2) in Figure 11I

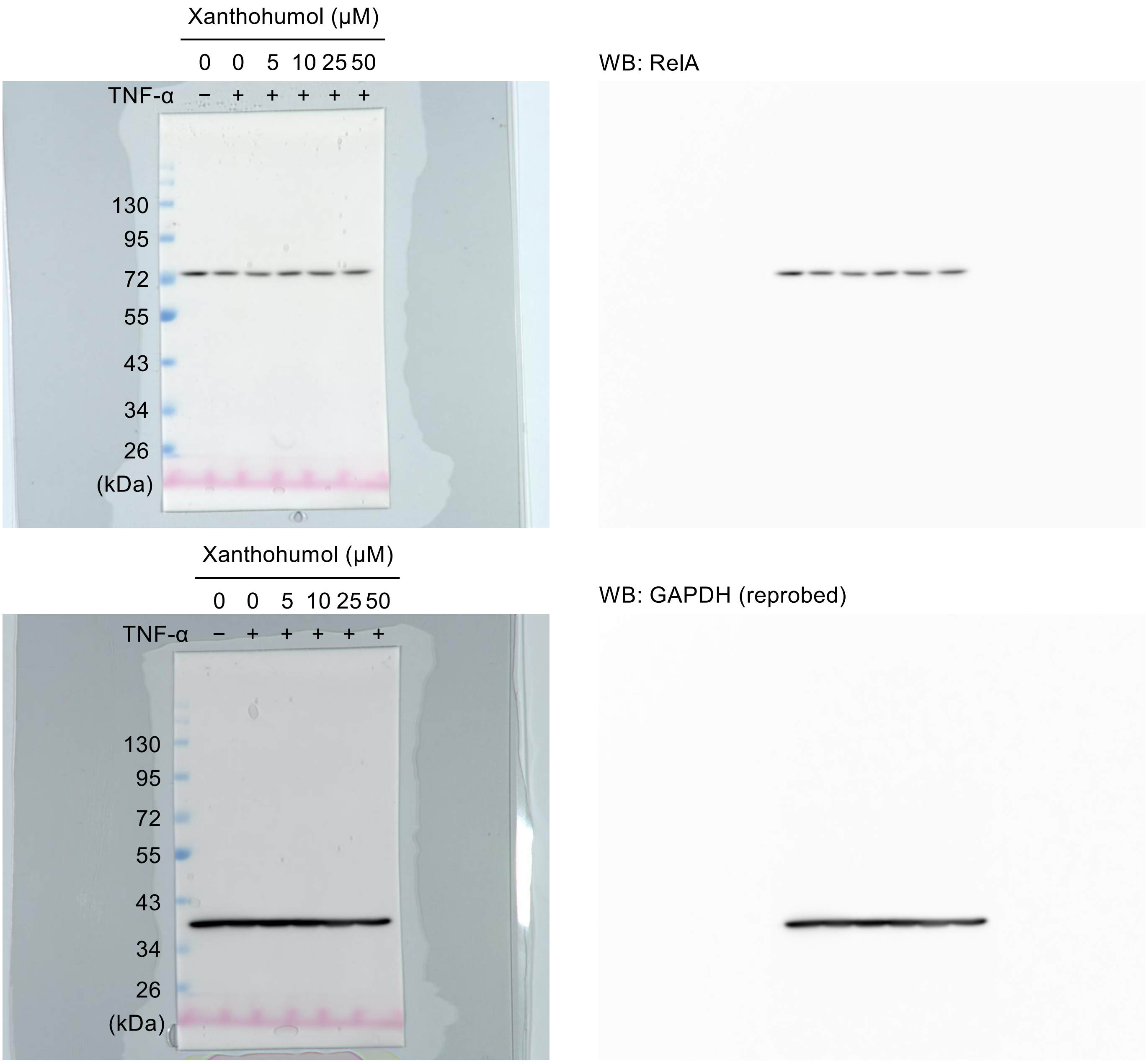

Figure S67: Original blots (3) in Figure 11I

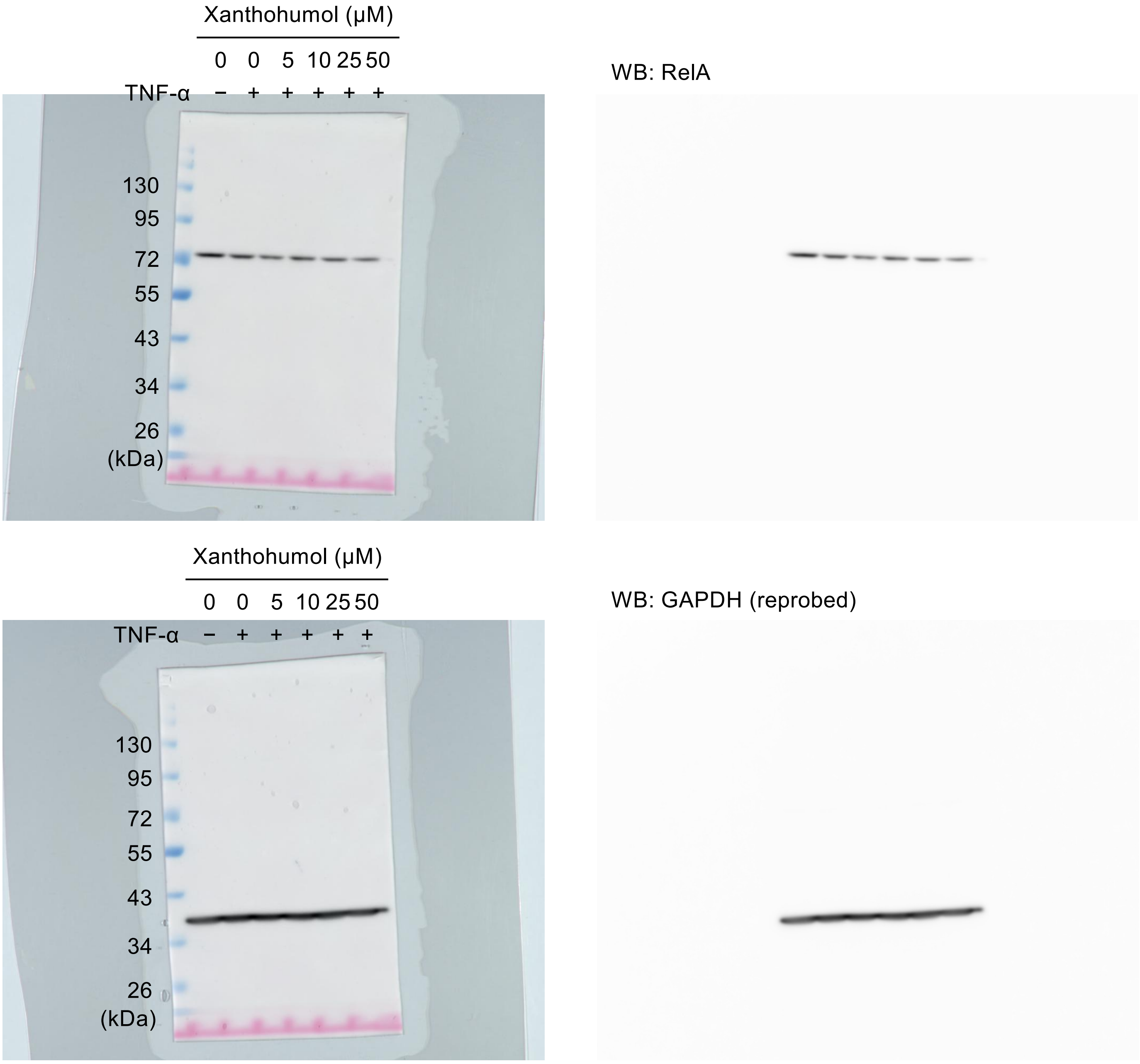

Figure S68: Original blots in Figure 12A

A549

Control
RelA WT
RelA C38S

WB: FLAG

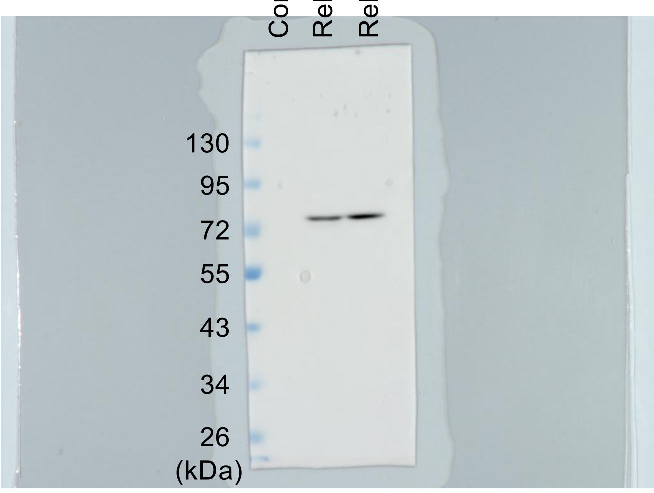

A549

Control
RelA WT
RelA C38S

WB: RelA (reprobed)

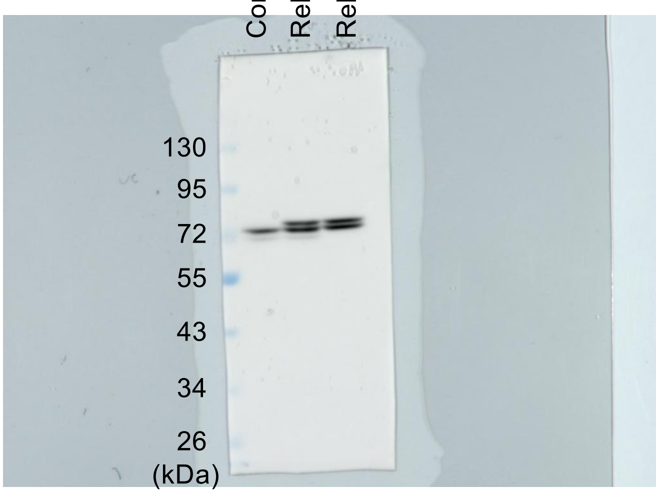

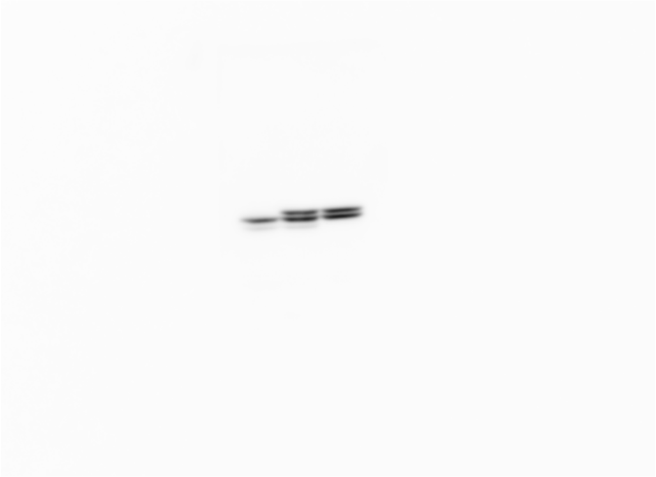

A549

Control
RelA WT
RelA C38S

WB: β-Actin (reprobed)

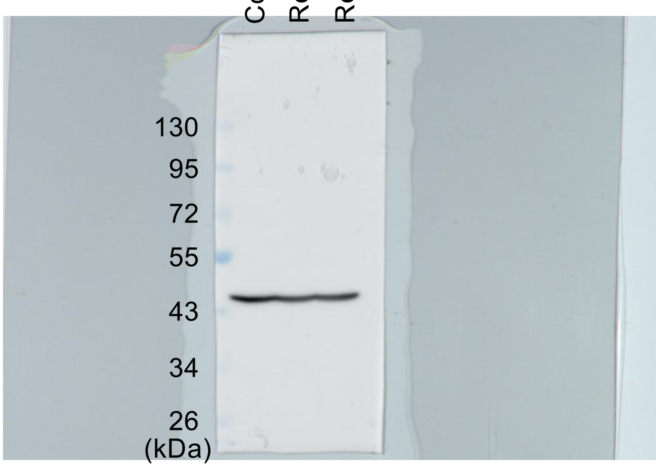

Figure S69: Original blots in Figure 12B (nucleus)

Cardamonin (μM)

0 0 25 25

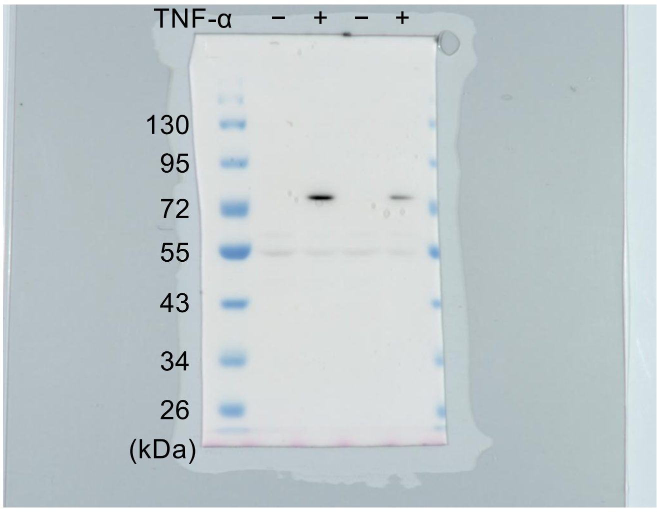

WB: RelA

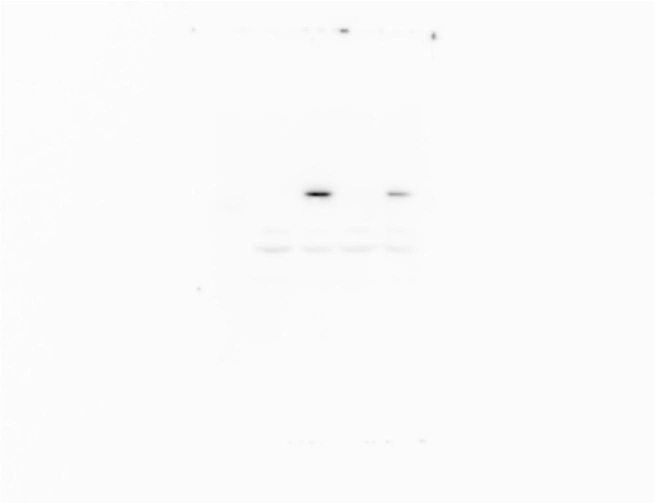

Cardamonin (μM)

0 0 25 25

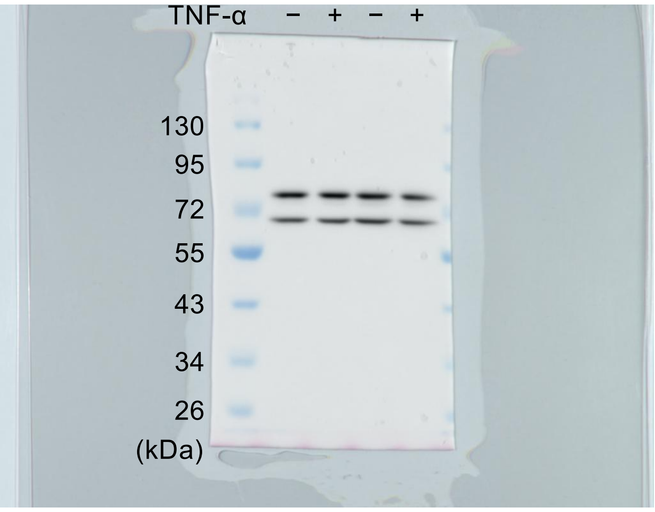

WB: Lamin A/C (reprobed)

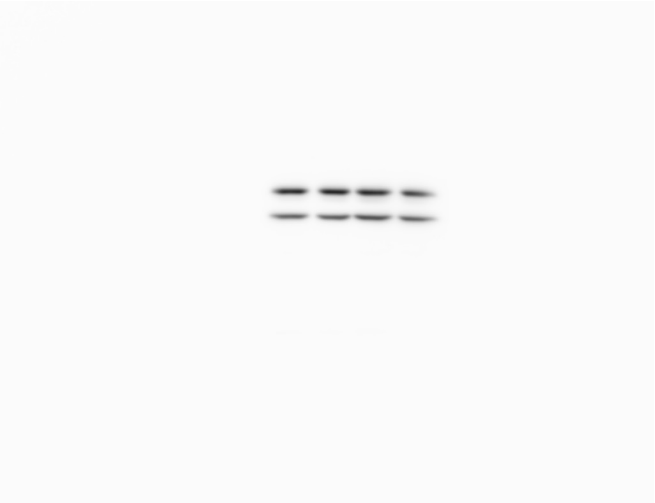

Figure S70: Original blots in Figure 12B (cytoplasm)

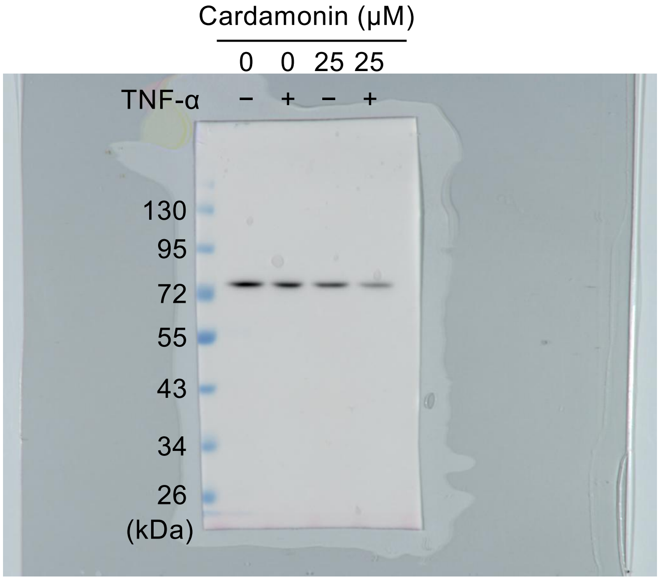

WB: RelA

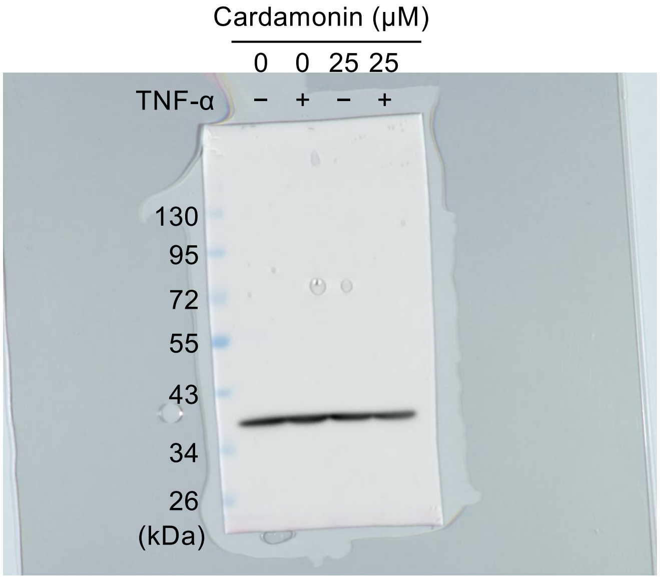

WB: GAPDH (reprobed)

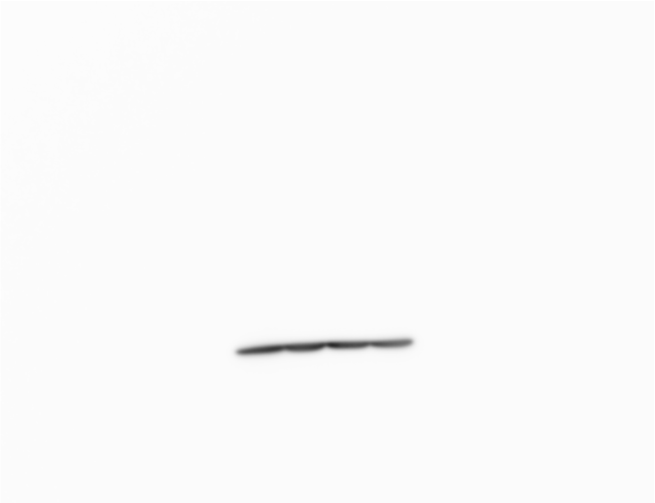

Figure S71: Original blots (1) in Figure 12C

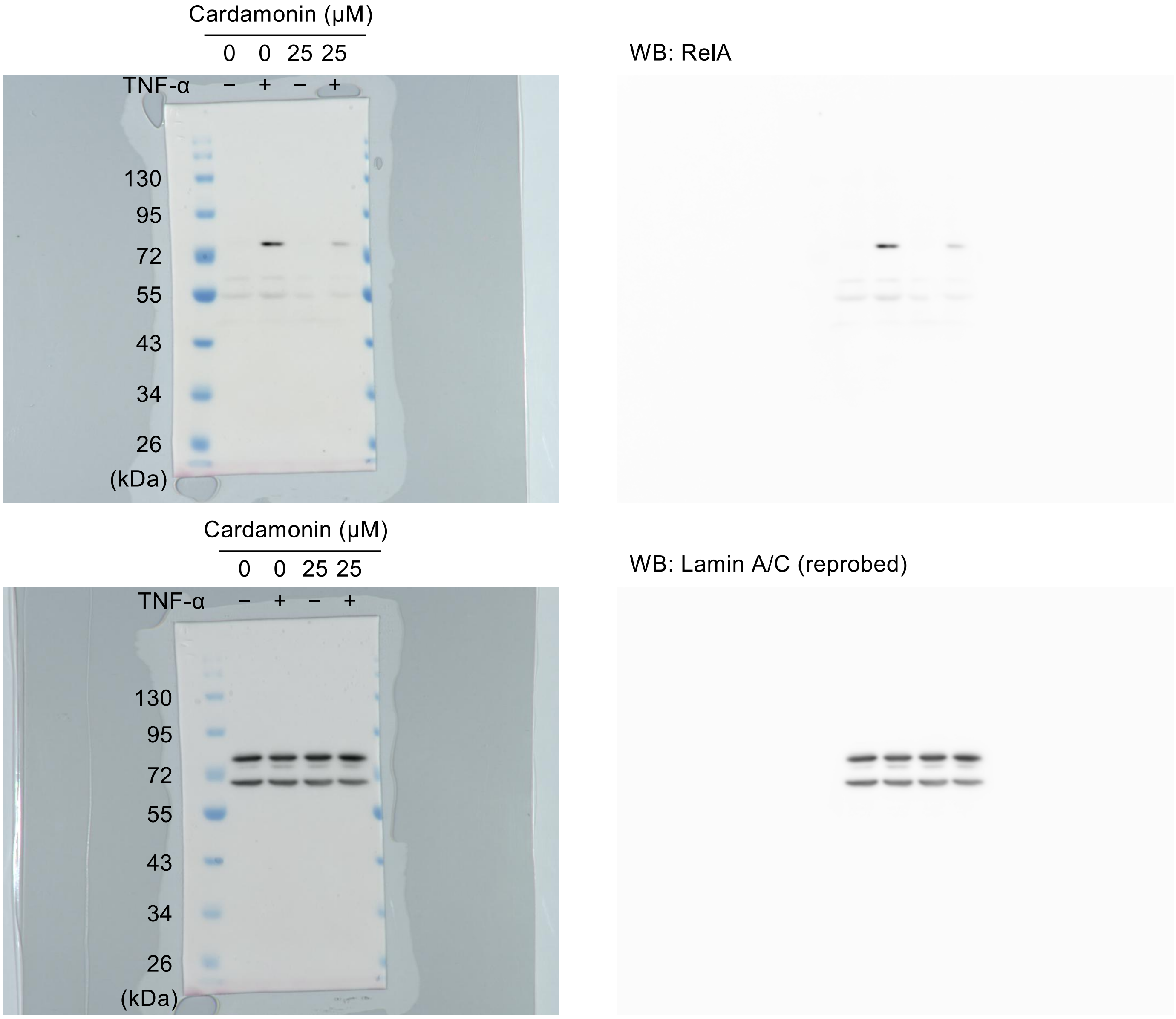

Figure S72: Original blots (2) in Figure 12C

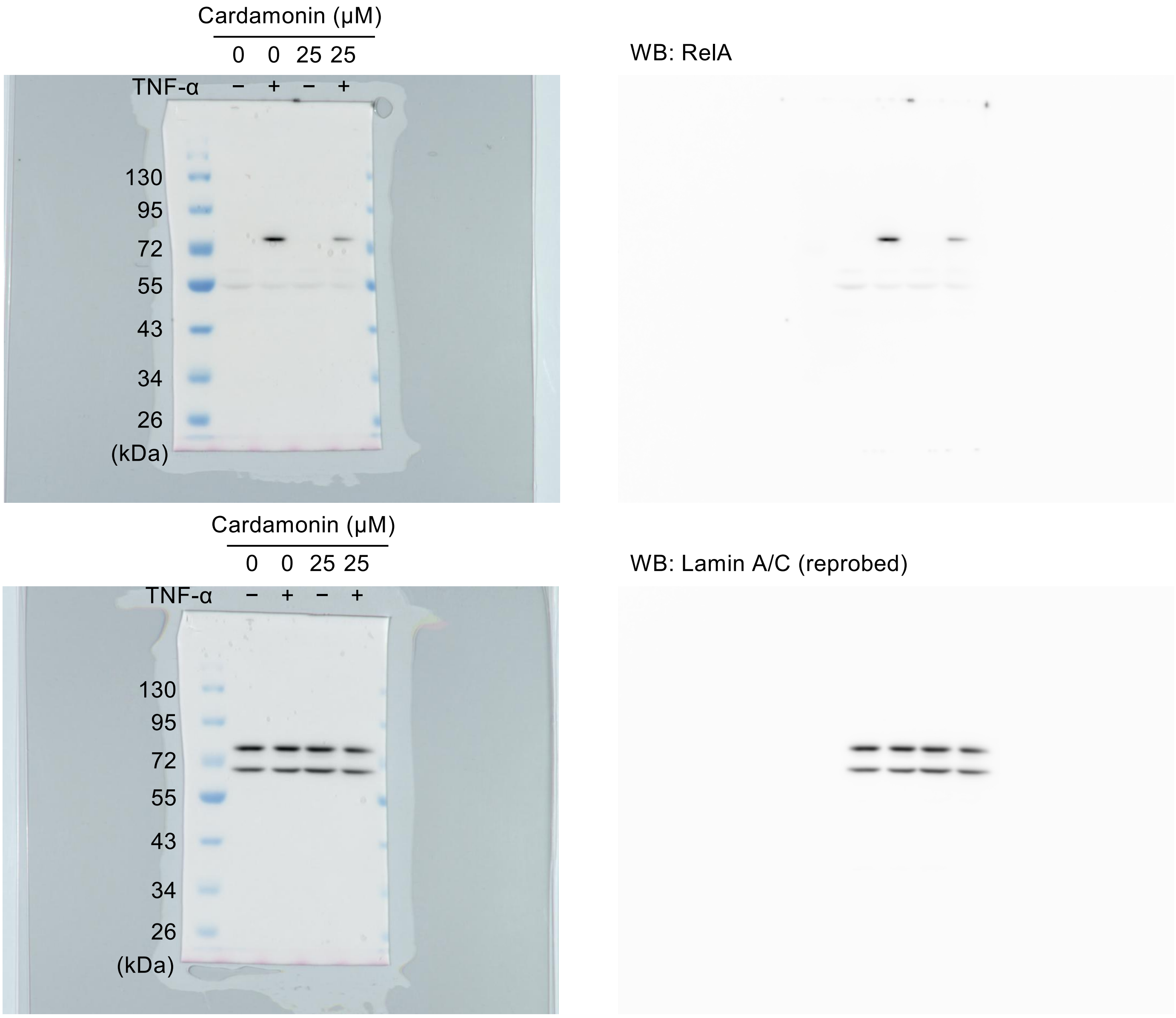

Figure S73: Original blots (3) in Figure 12C

Cardamonin (μM)

0 0 25 25

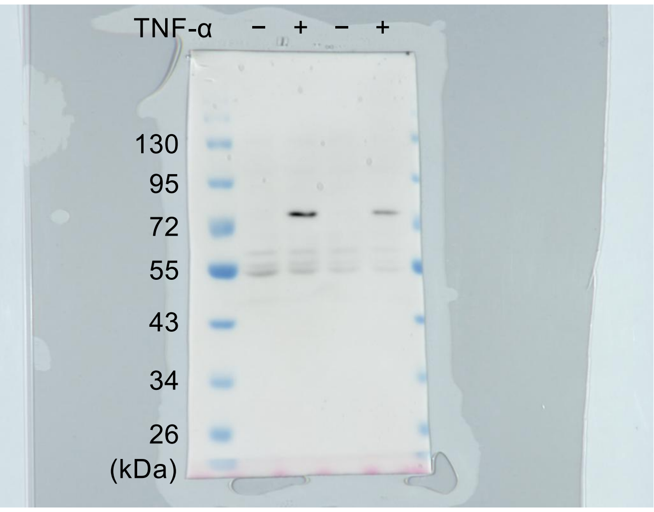

WB: RelA

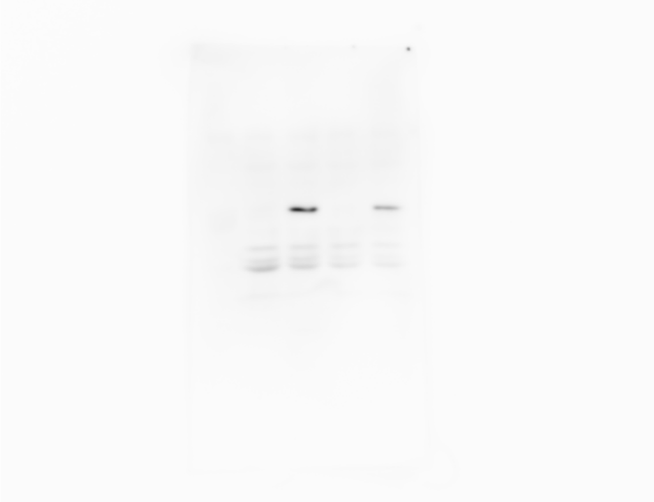

Cardamonin (μM)

0 0 25 25

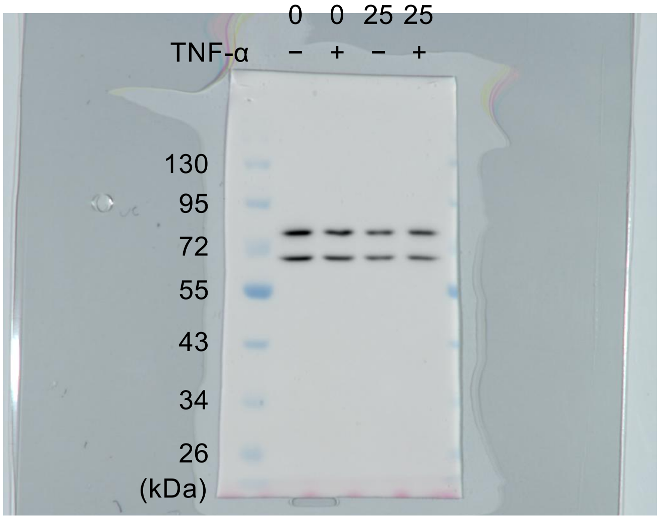

WB: Lamin A/C (reprobed)

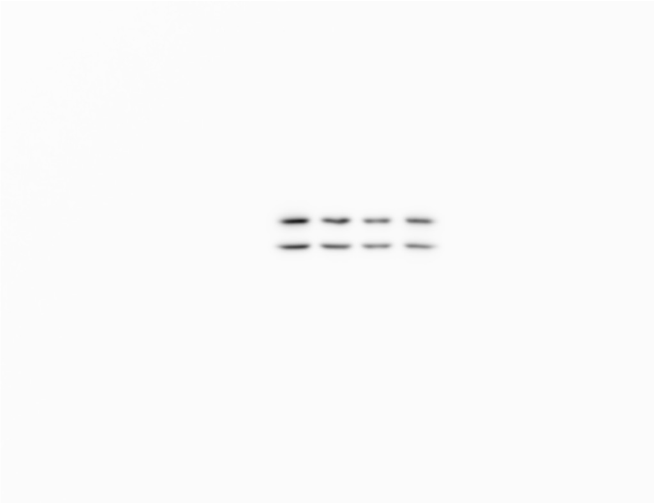

Figure S74: Original blots (1) in Figure 12D

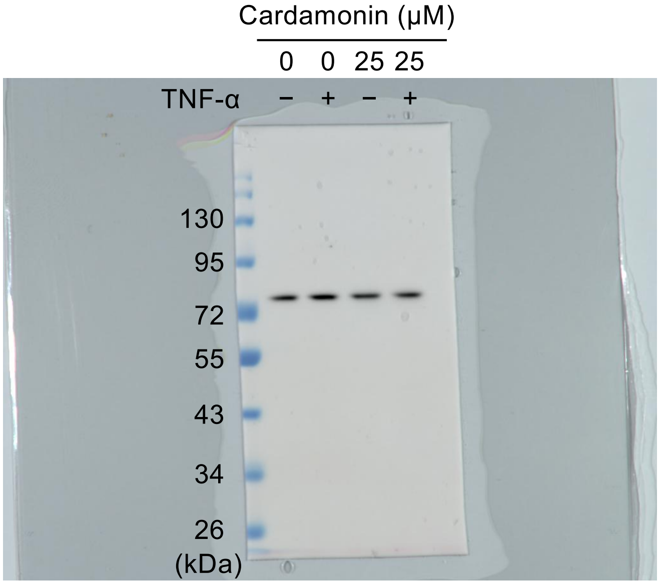

WB: RelA

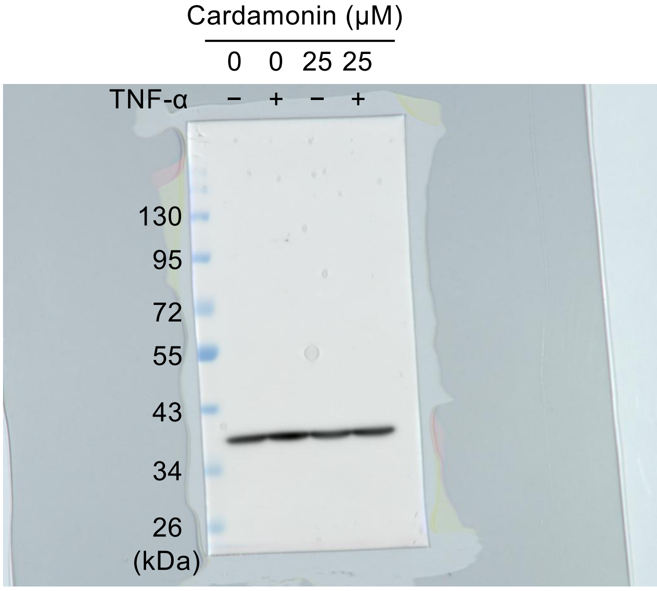

WB: GAPDH (reprobed)

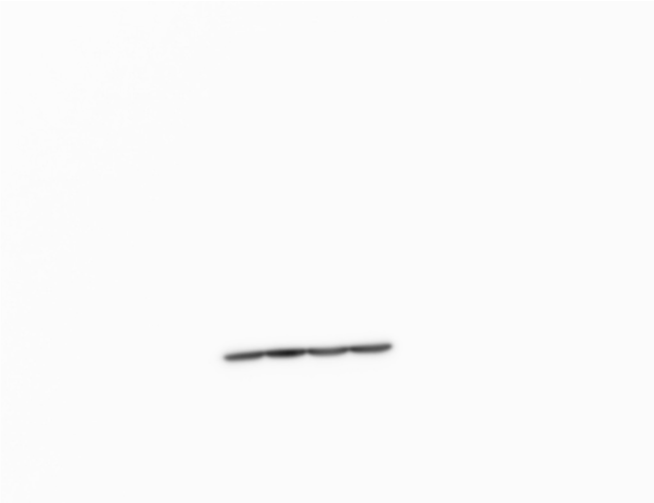

Figure S75: Original blots (2) in Figure 12D

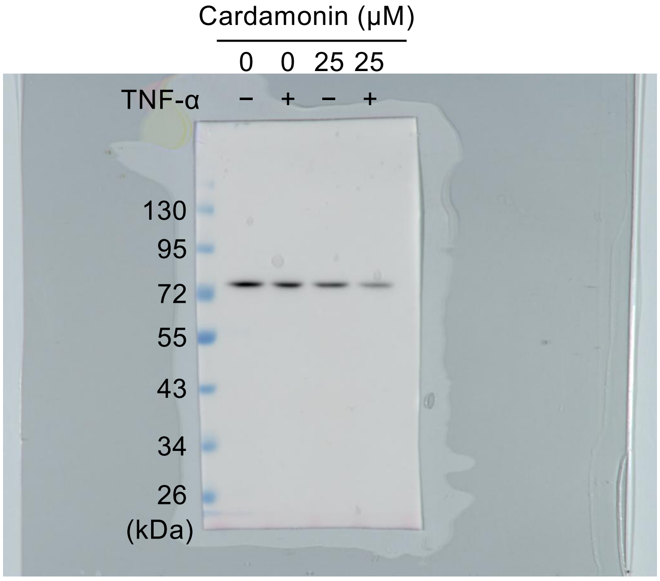

WB: RelA

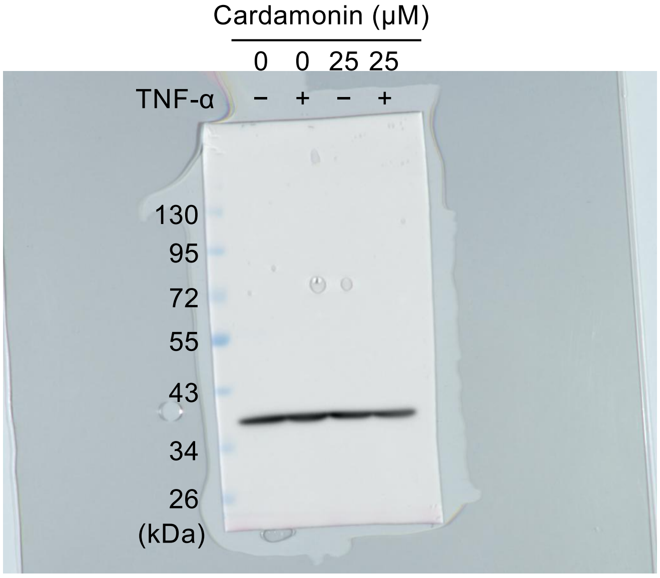

WB: GAPDH (reprobed)

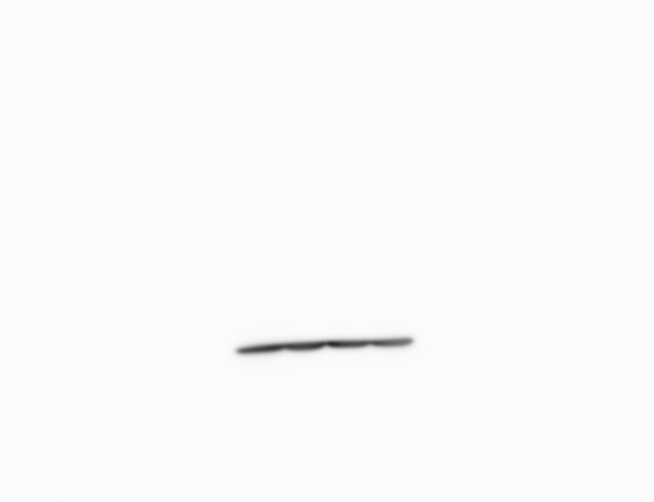

Figure S76: Original blots (3) in Figure 12D

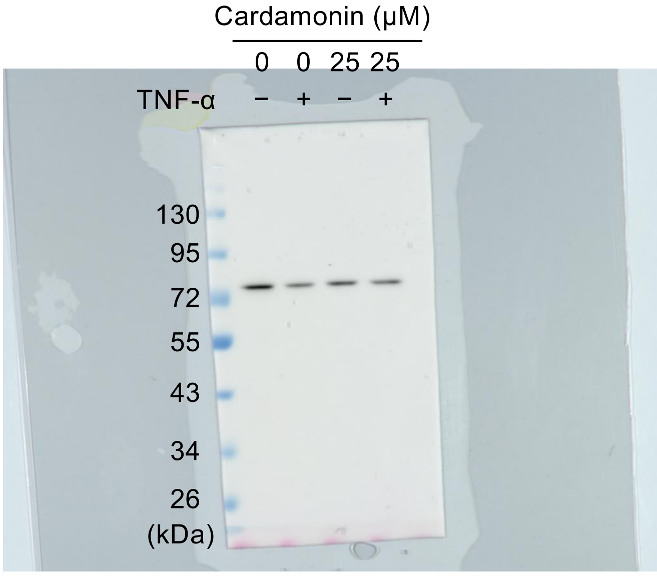

WB: RelA

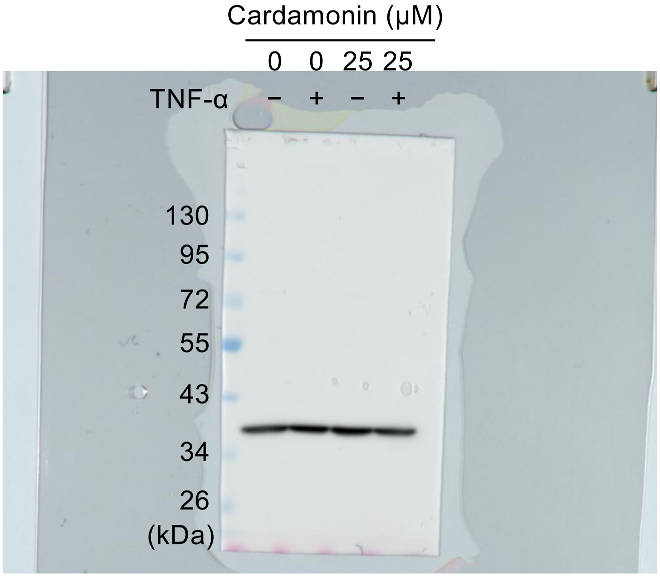

WB: GAPDH (reprobed)

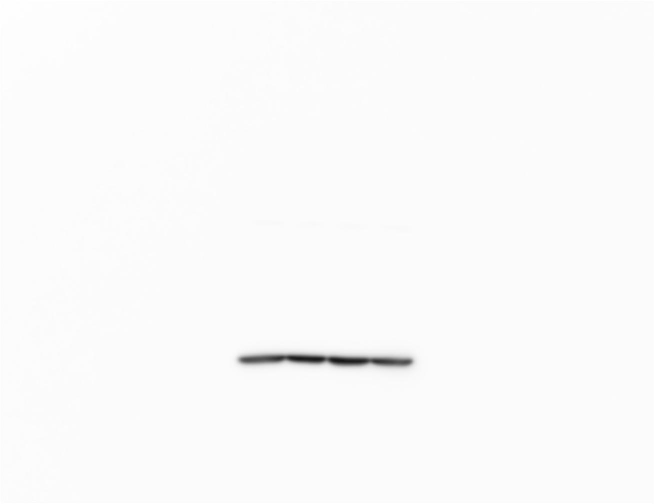

Figure S77: Original blots in Figure 12E (nucleus)

Cardamonin (μM)

0 0 25 25

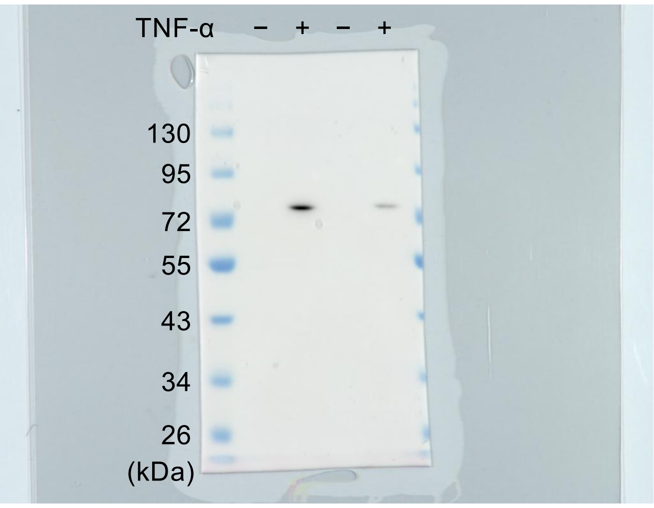

WB: RelA

Cardamonin (μM)

0 0 25 25

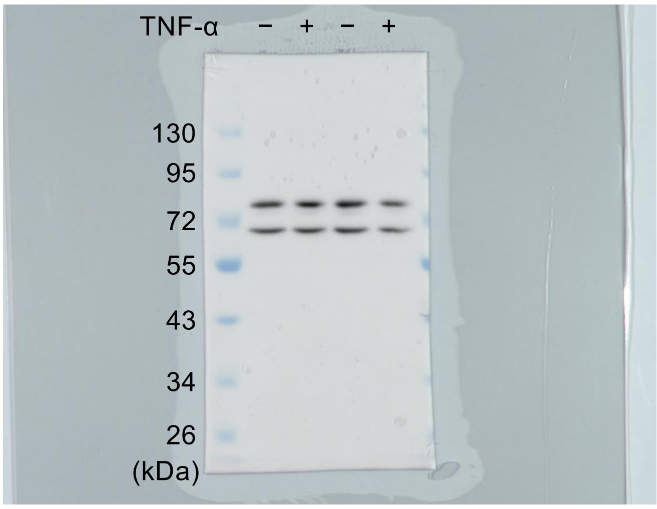

WB: Lamin A/C (reprobed)

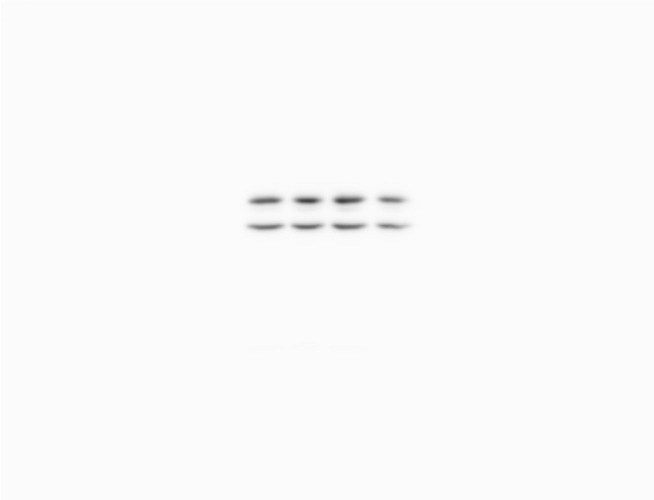

Figure S78: Original blots in Figure 12E (cytoplasm)

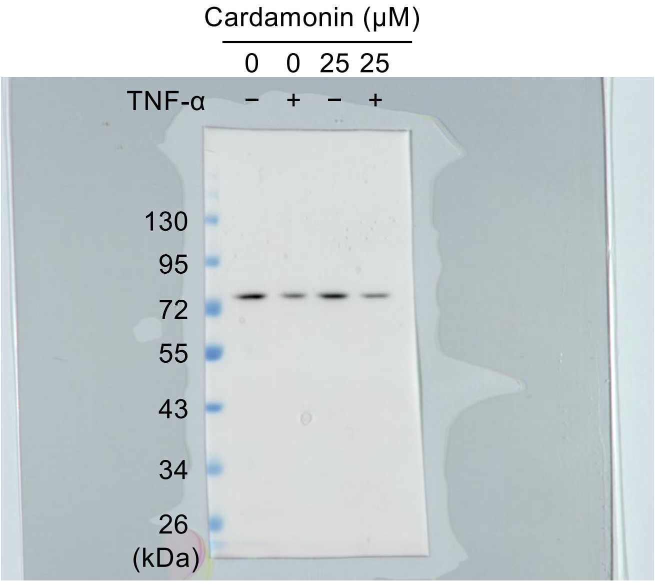

WB: RelA

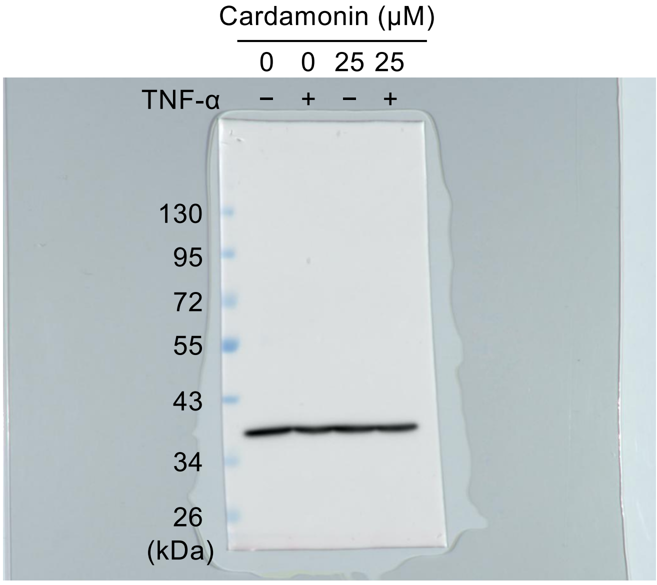

WB: GAPDH (reprobed)

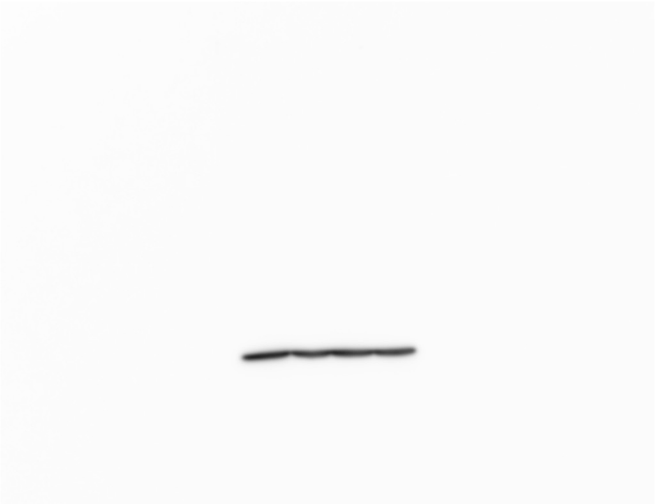

Figure S79: Original blots (1) in Figure 12F

Cardamonin (μM)

0 0 25 25

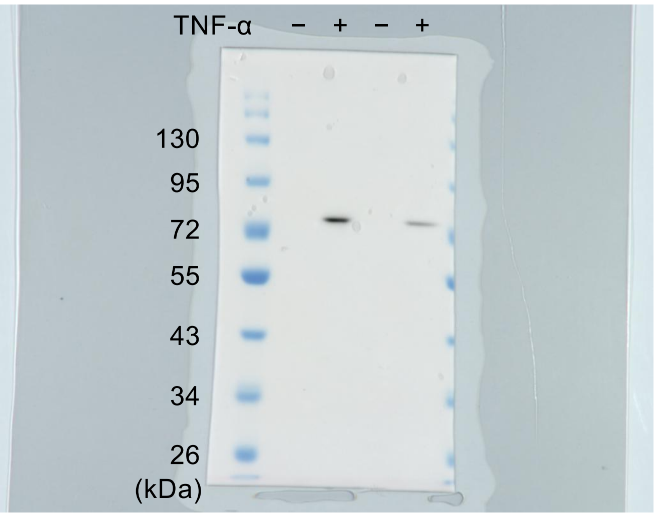

WB: RelA

Cardamonin (μM)

0 0 25 25

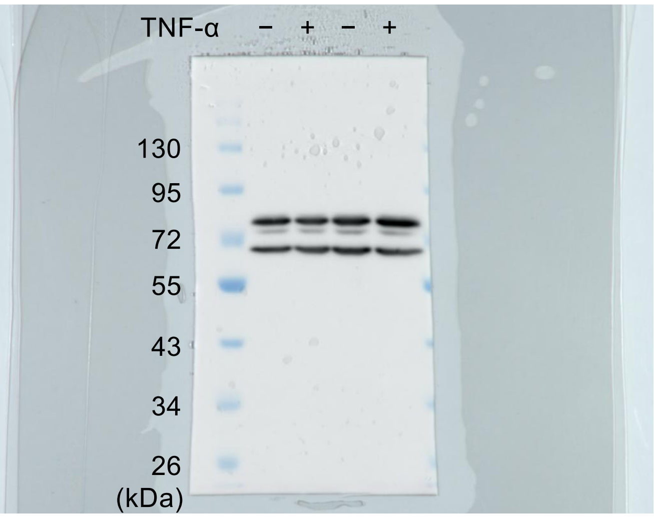

WB: Lamin A/C (reprobed)

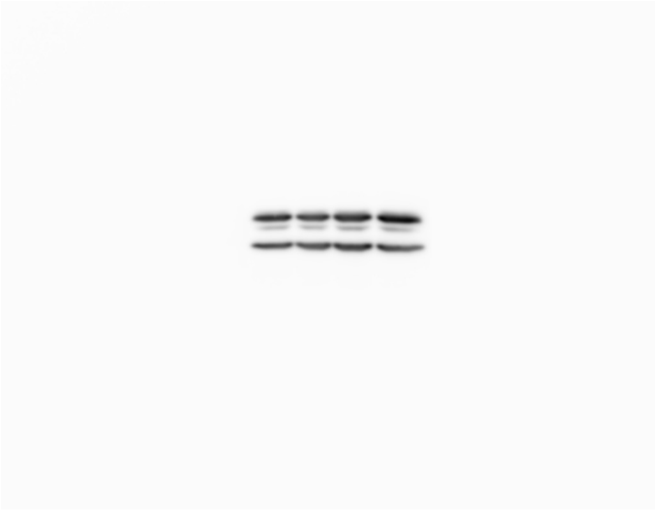

Figure S80: Original blots (2) in Figure 12F

Cardamonin (μM)

0 0 25 25

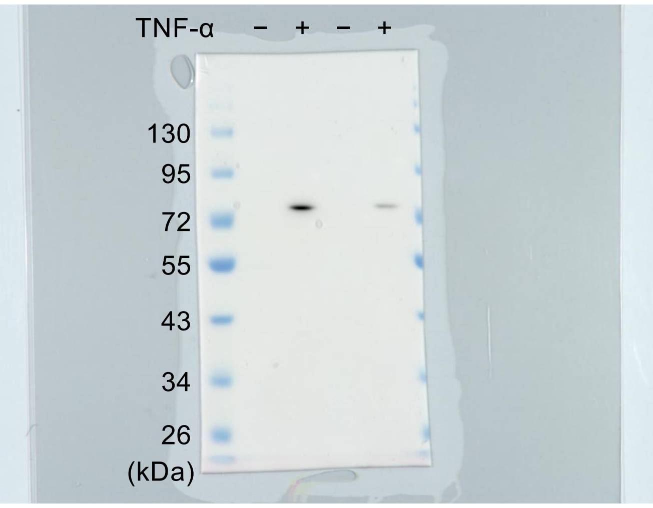

WB: RelA

Cardamonin (μM)

0 0 25 25

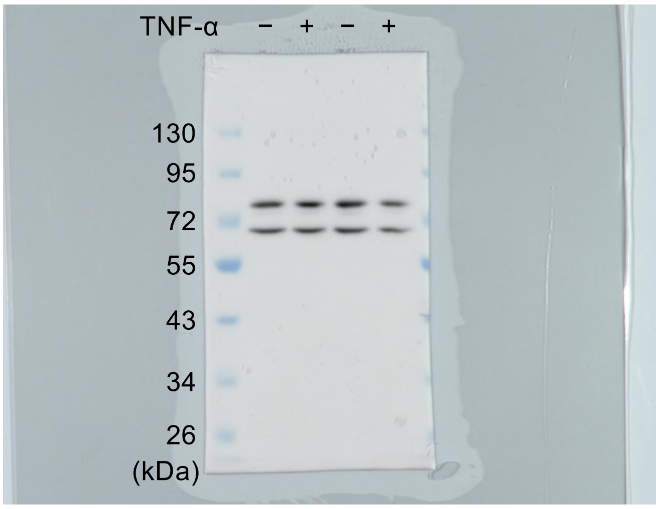

WB: Lamin A/C (reprobed)

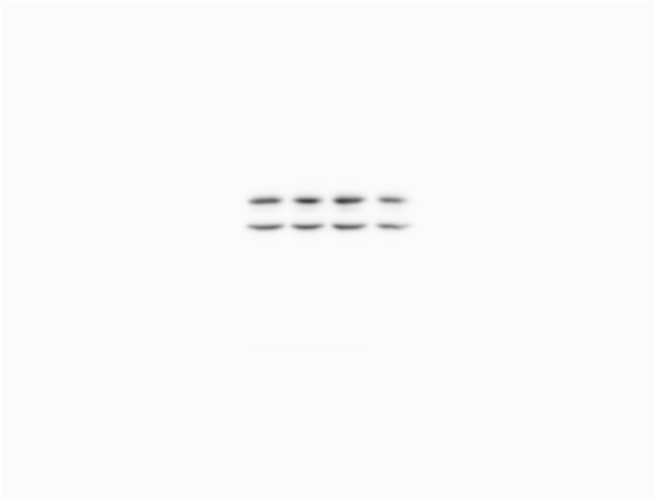

Figure S81: Original blots (3) in Figure 12F

Cardamonin (μM)

0 0 25 25

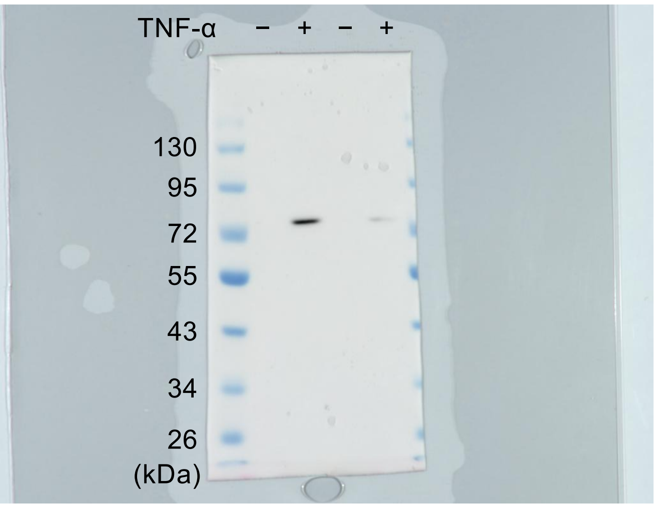

WB: RelA

Cardamonin (μM)

0 0 25 25

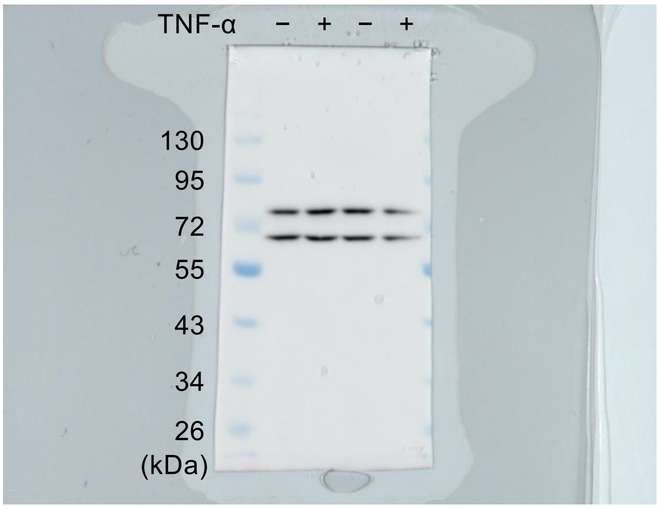

WB: Lamin A/C (reprobed)

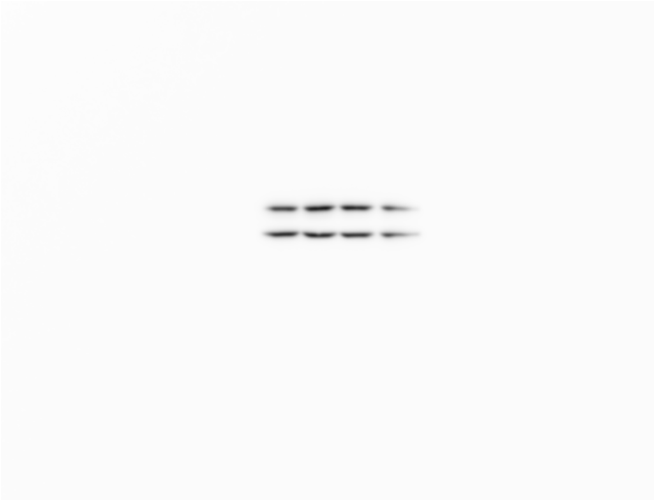

Figure S82: Original blots (1) in Figure 12G

Cardamonin (μM)

0 0 25 25

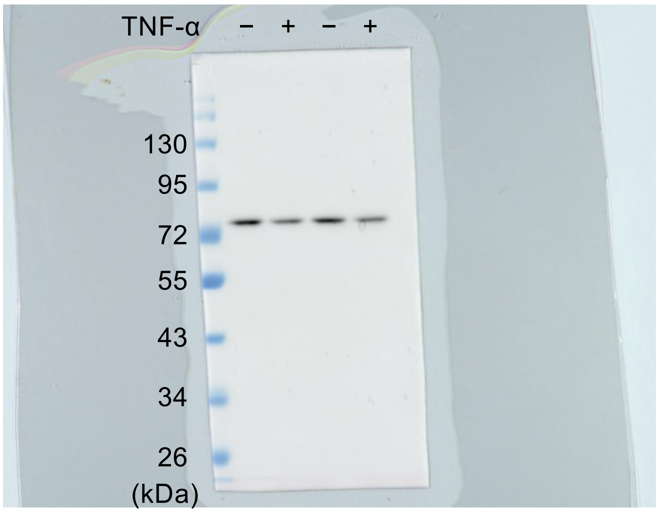

WB: RelA

Cardamonin (μM)

0 0 25 25

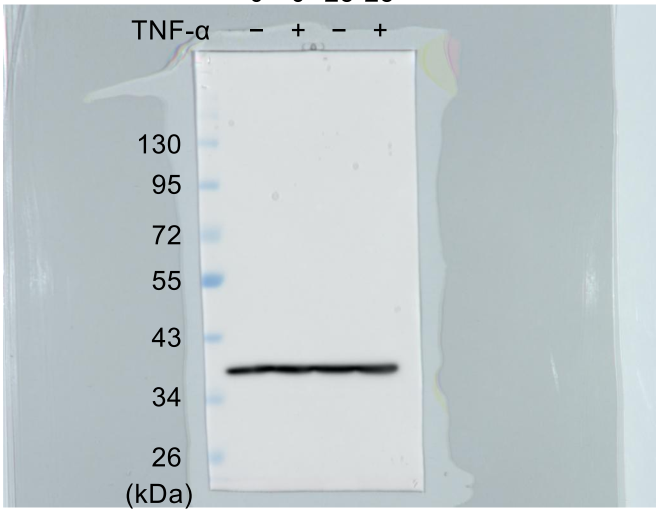

WB: GAPDH (reprobed)

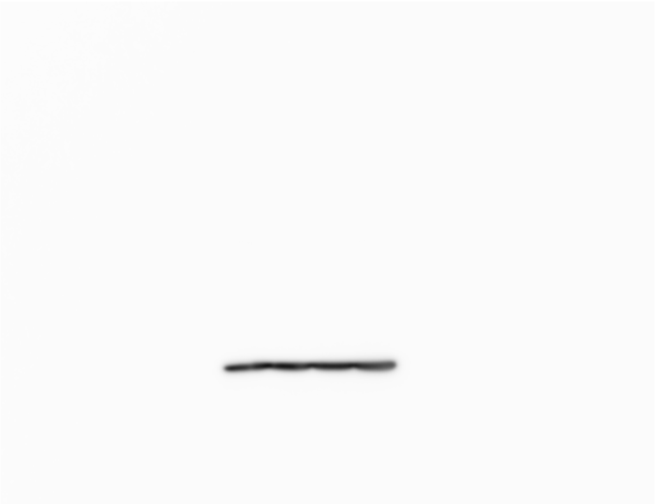

Figure S83: Original blots (2) in Figure 12G

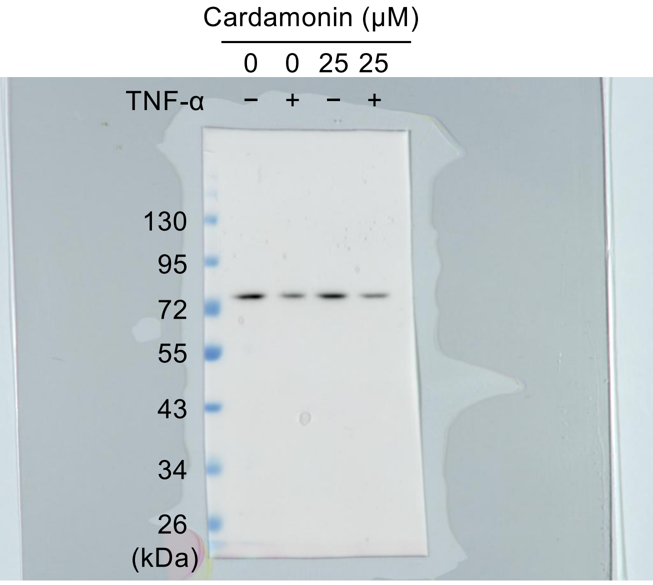

WB: RelA

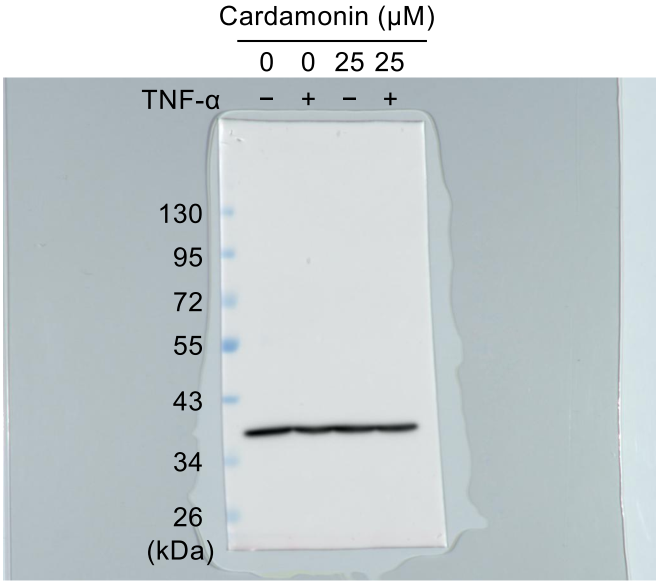

WB: GAPDH (reprobed)

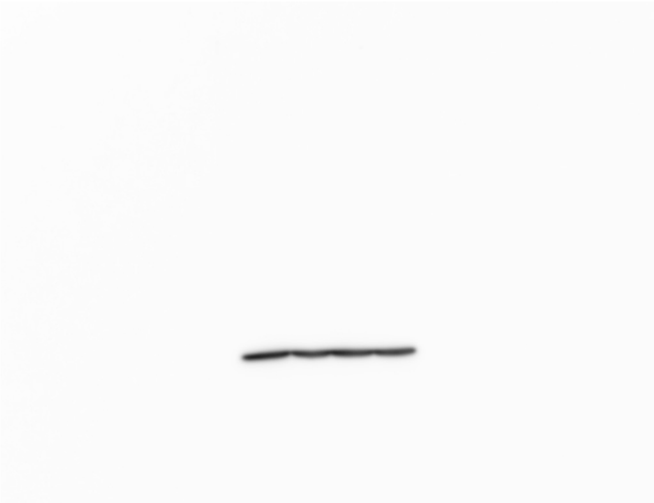

Figure S84: Original blots (3) in Figure 12G

Cardamonin (μM)

0 0 25 25

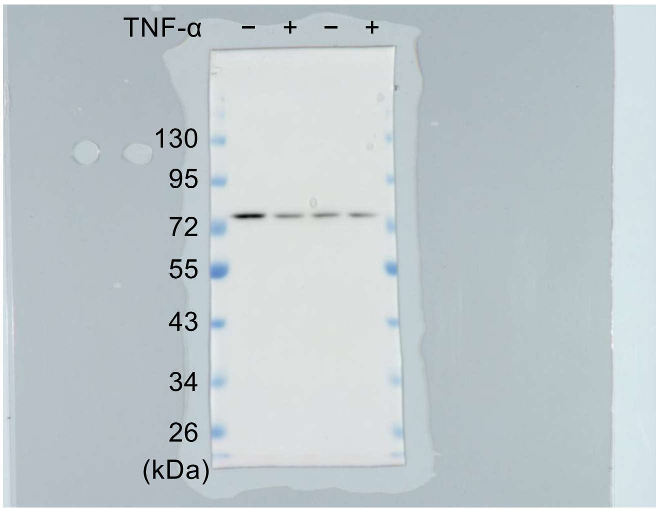

WB: RelA

Cardamonin (μM)

0 0 25 25

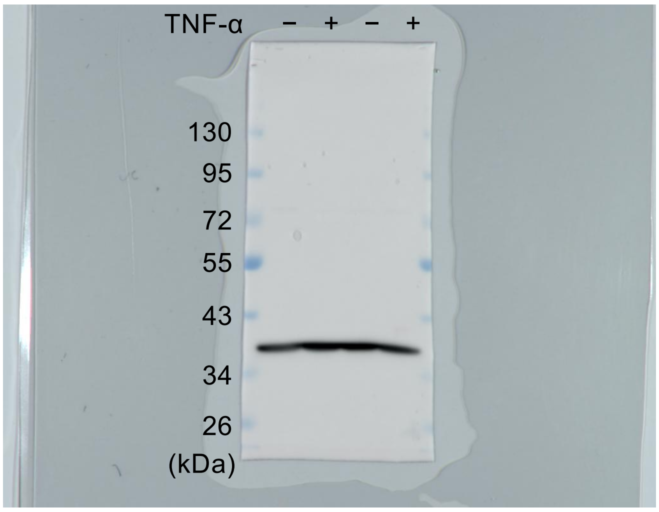

WB: GAPDH (reprobed)

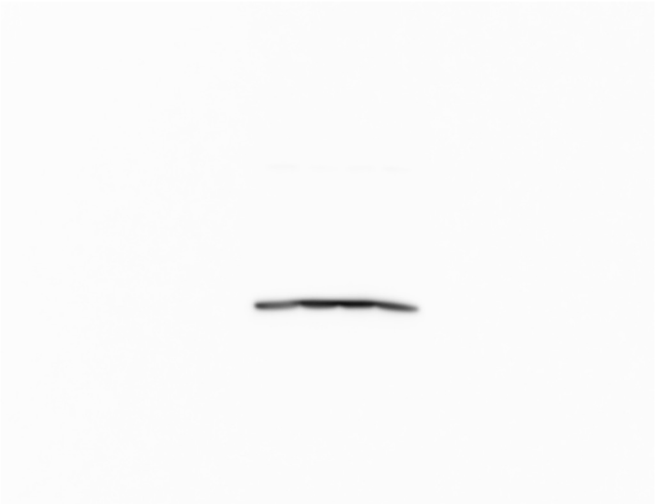

Figure S85: Original blots in Figure 14A

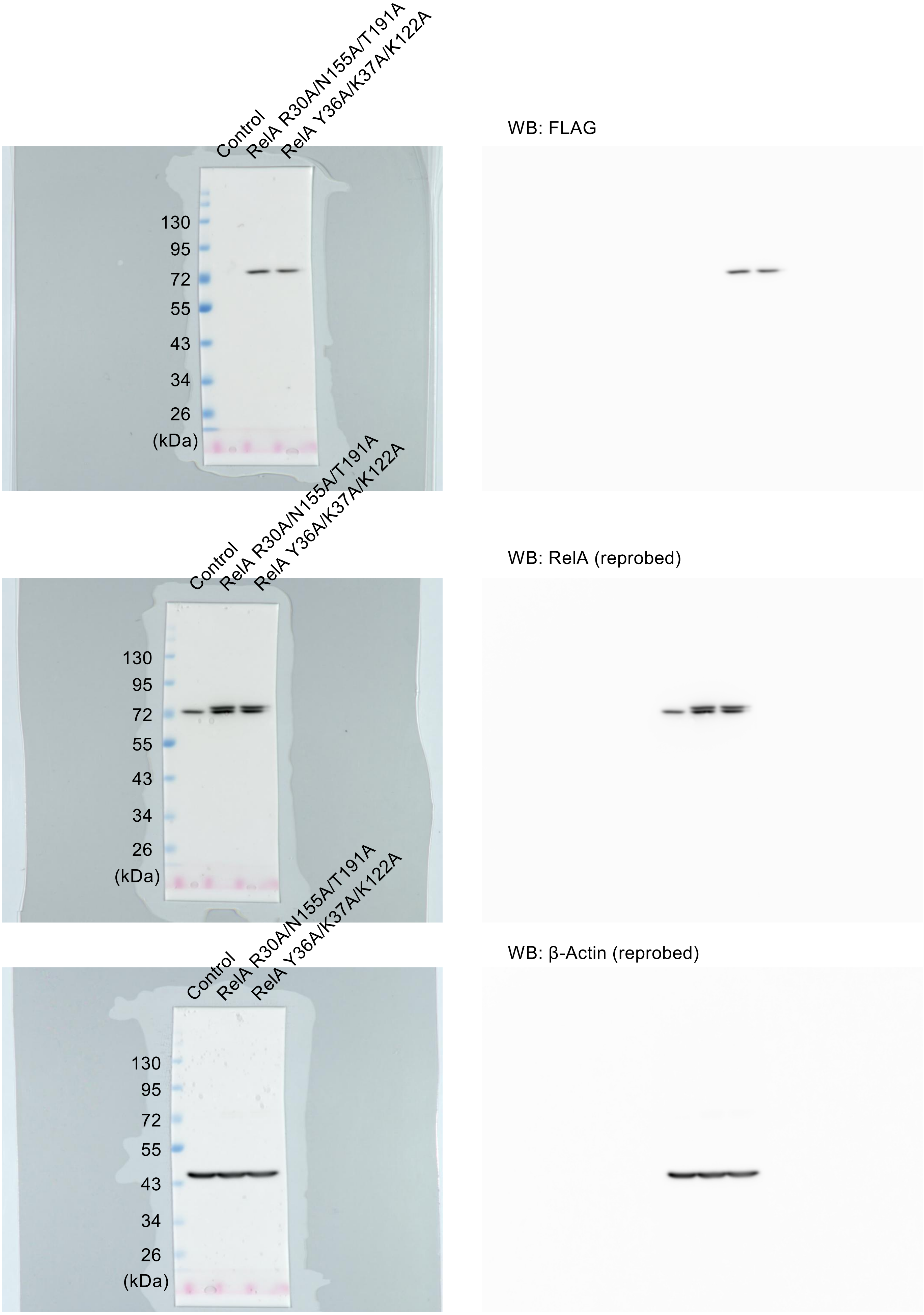

Figure S86: Original blots in Figure 14B (nucleus)

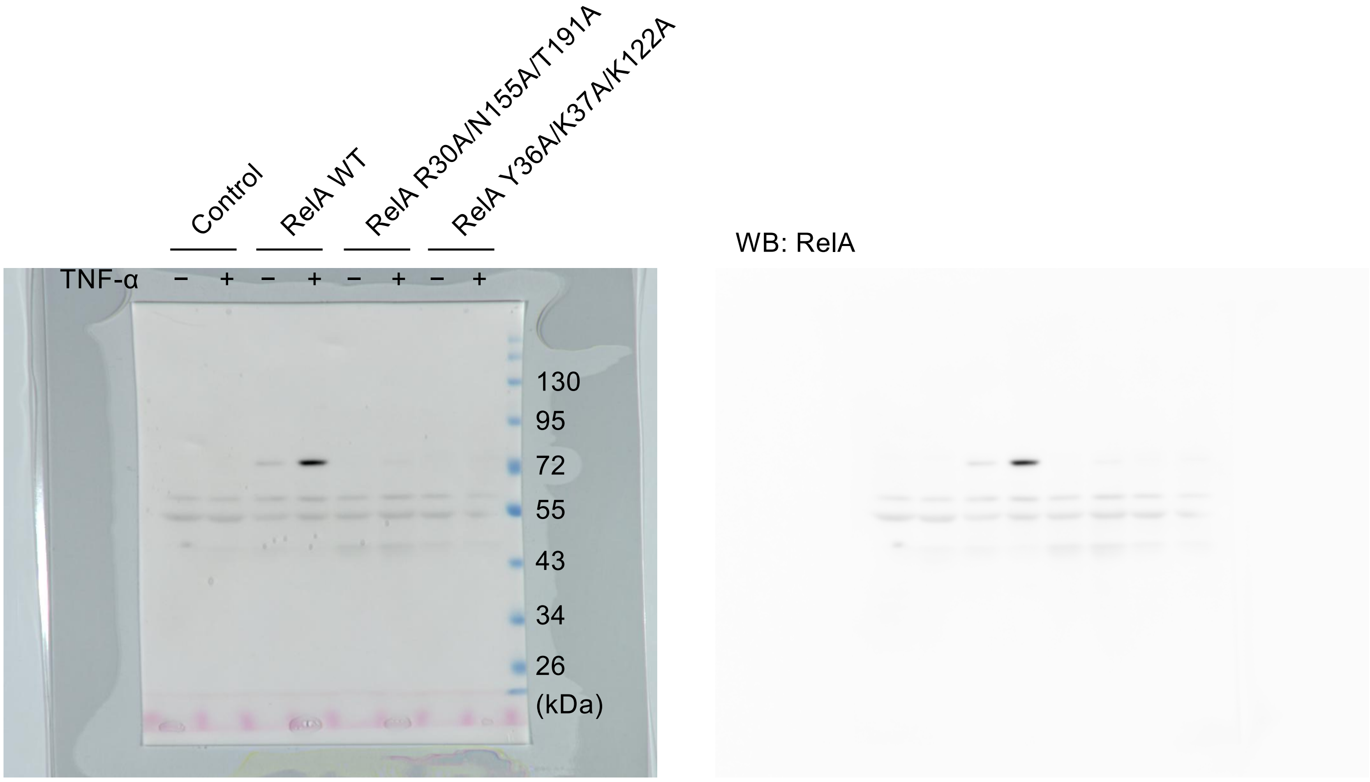

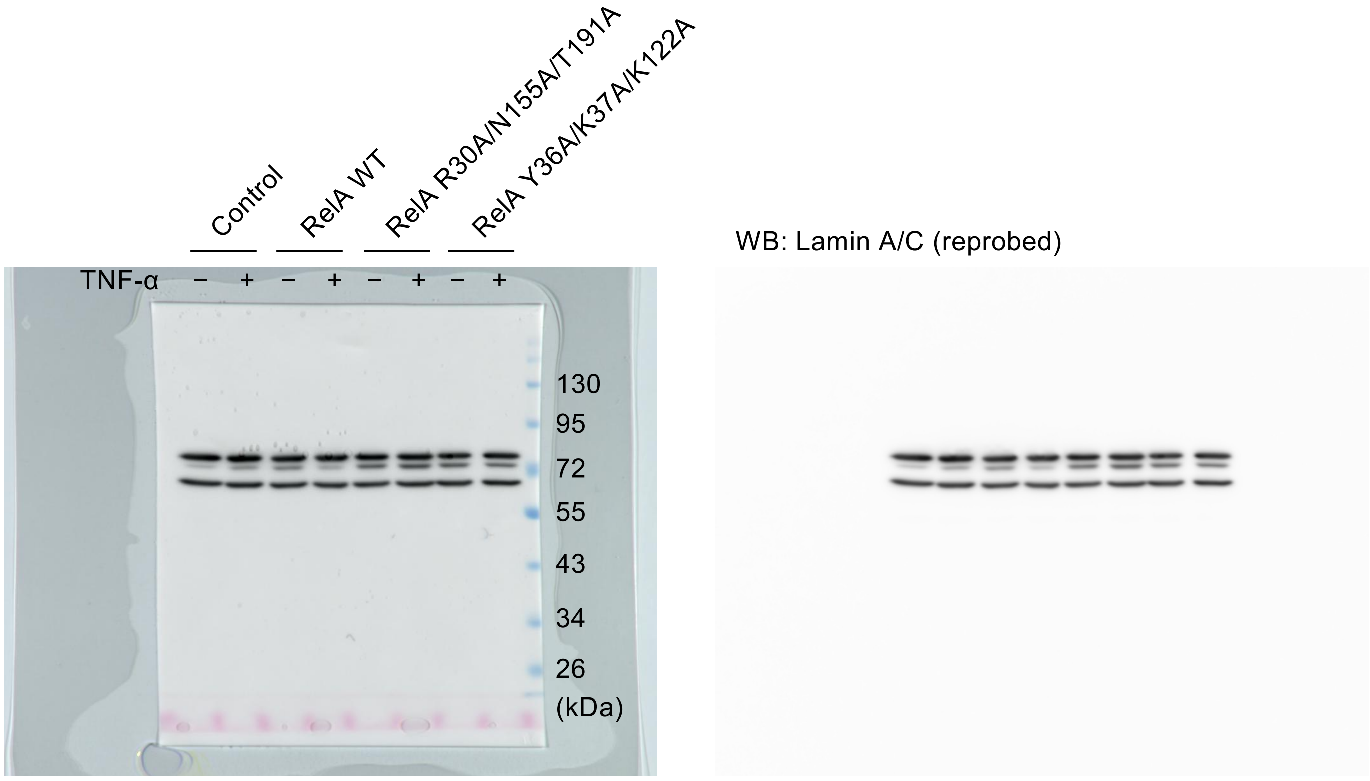

Figure S87: Original blots in Figure 14B (cytoplasm)

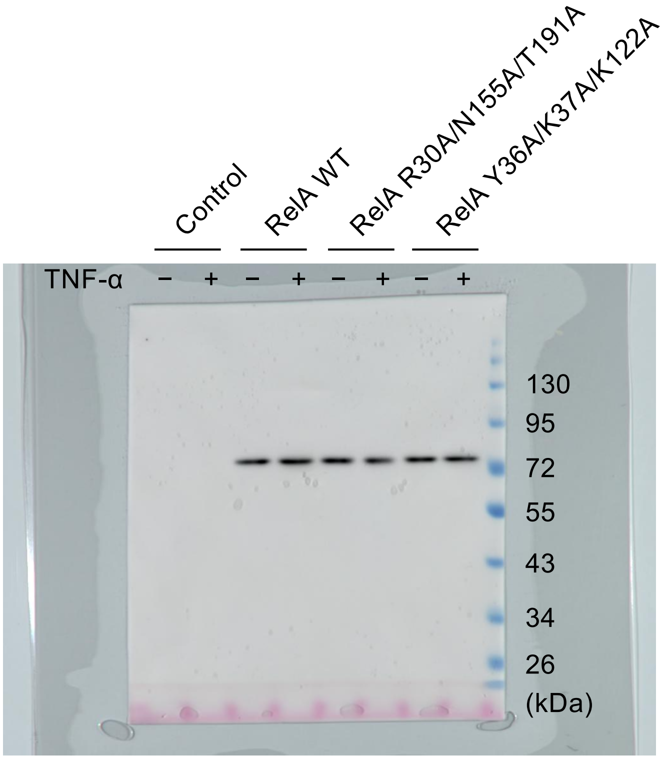

WB: RelA

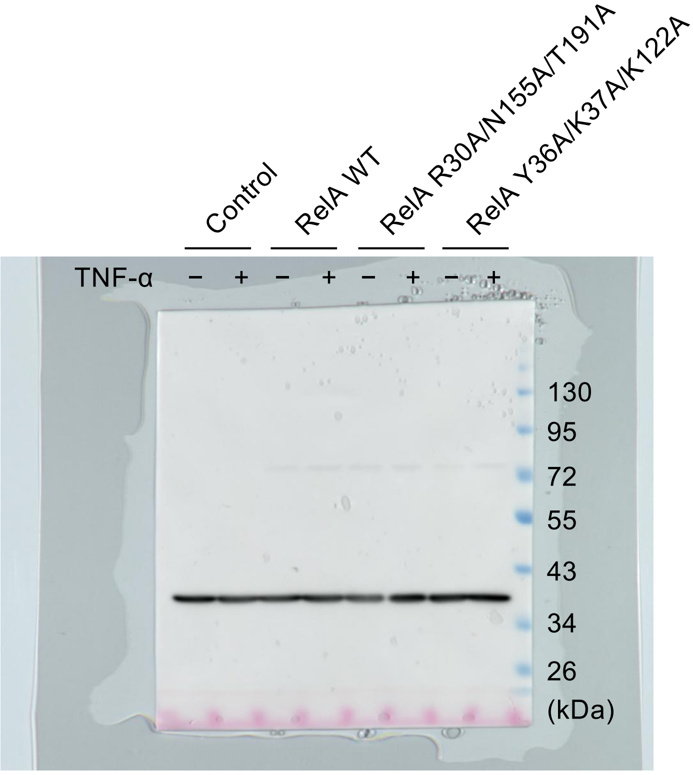

WB: GAPDH (reprobed)

Figure S88: Original blots (1) in Figure 14C

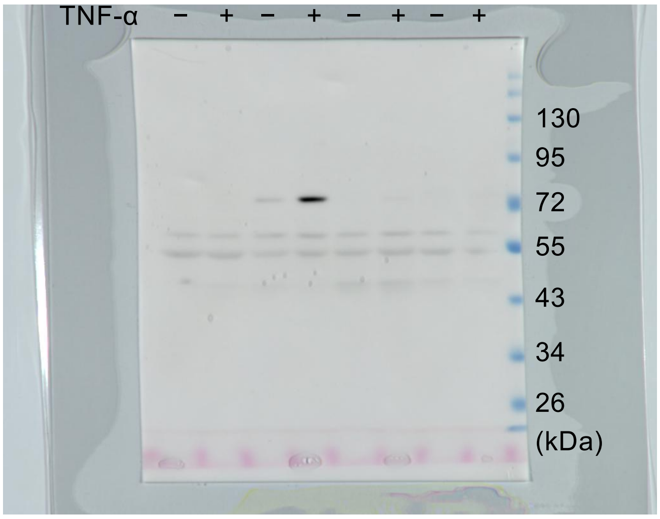

WB: RelA

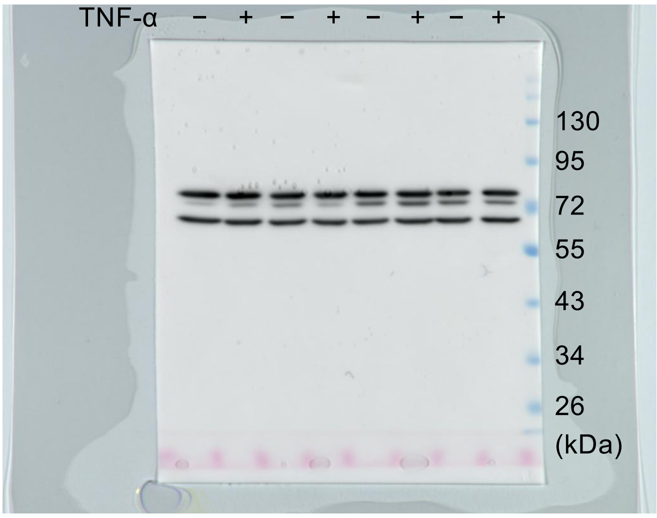

WB: LaminA/C (reprobed)

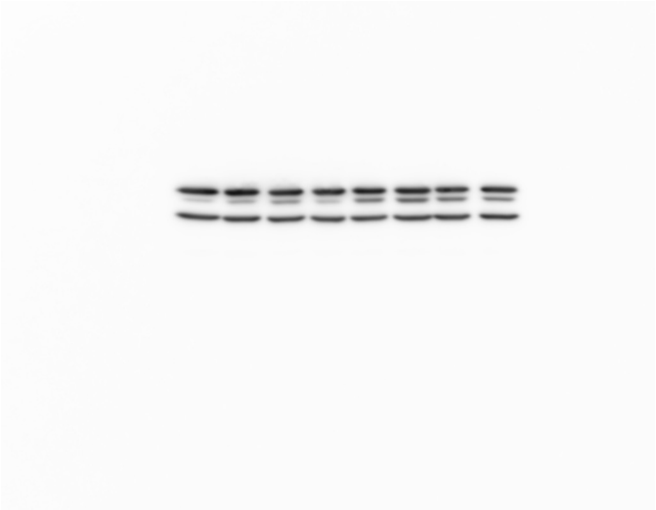

Figure S89: Original blots (2) in Figure 14C

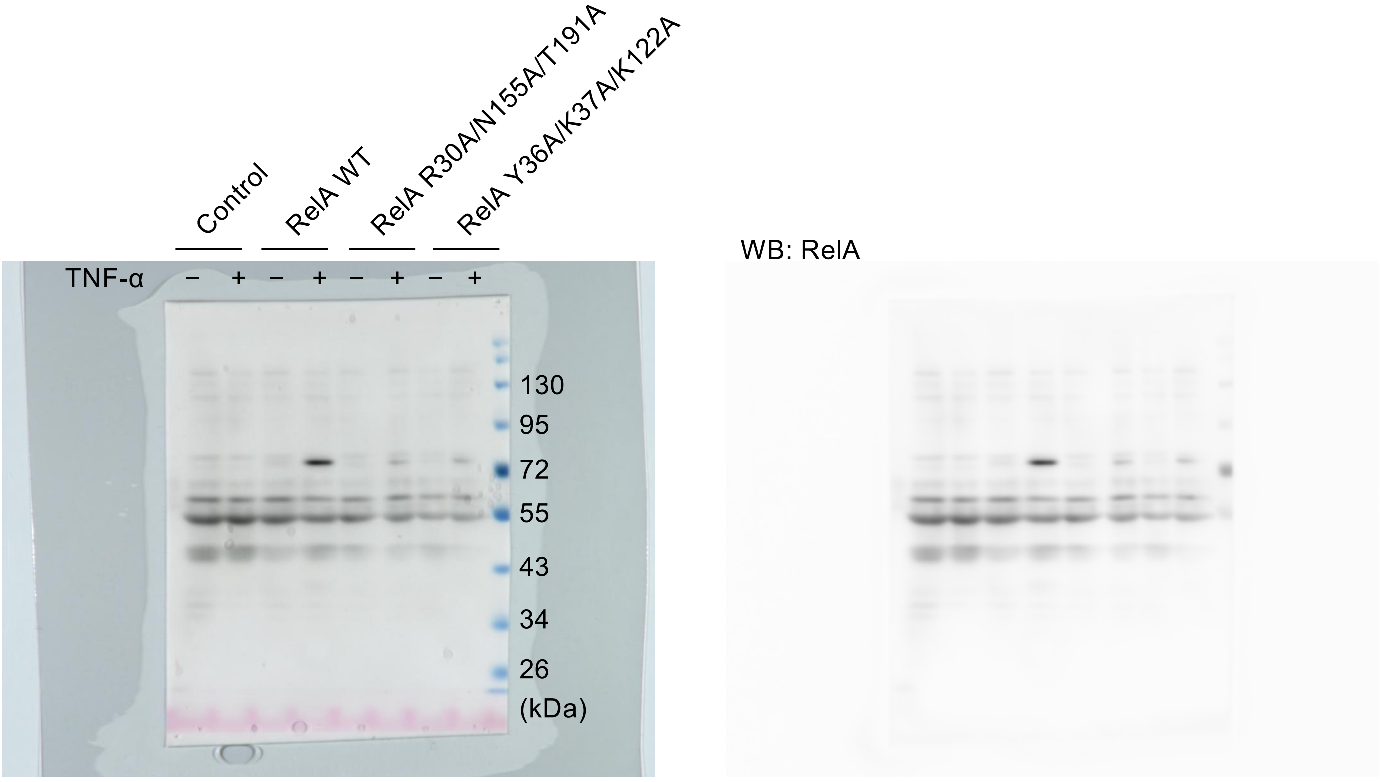

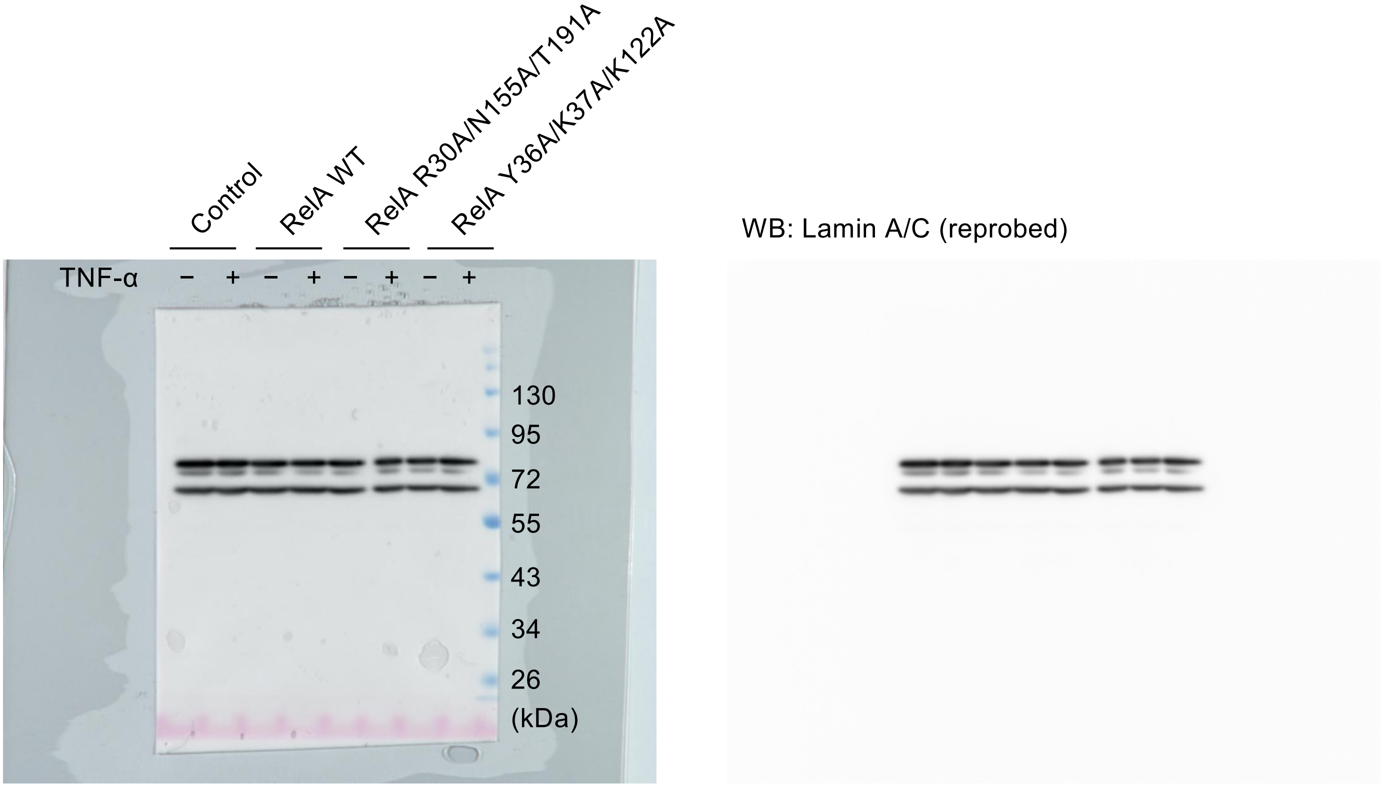

Figure S90: Original blots (3) in Figure 14C

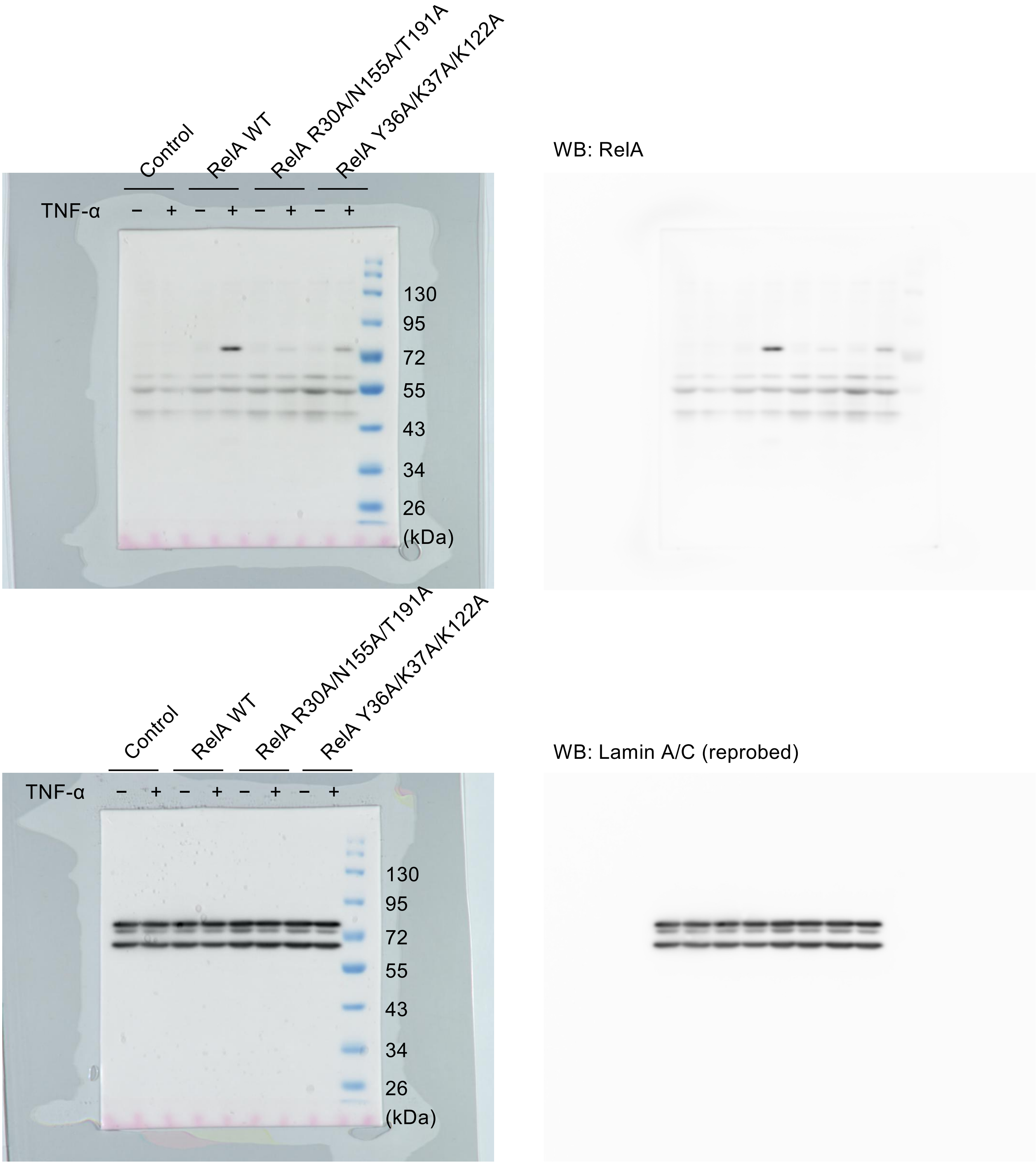

Figure S91: Original blots (1) in Figure 14D

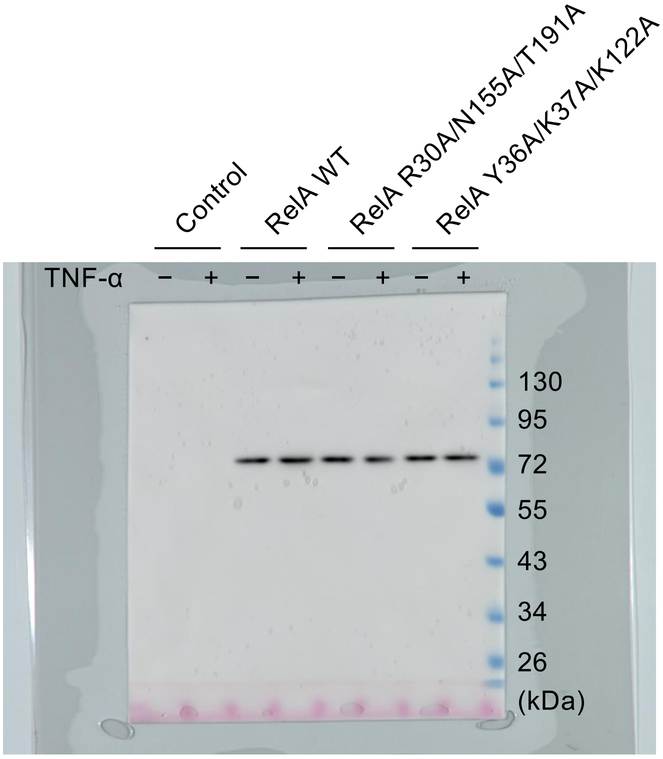

WB: RelA

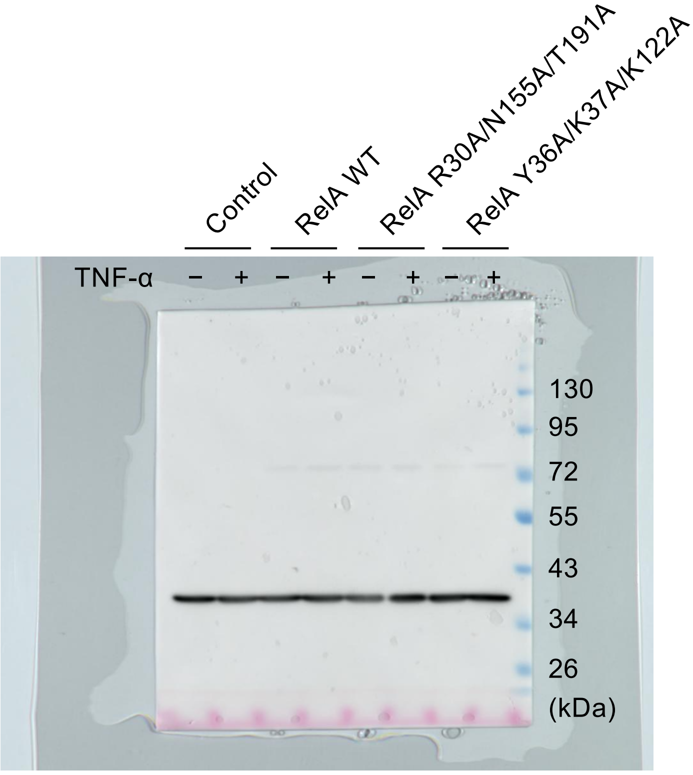

WB: GAPDH (reprobed)

Figure S92: Original blots (2) in Figure 14D

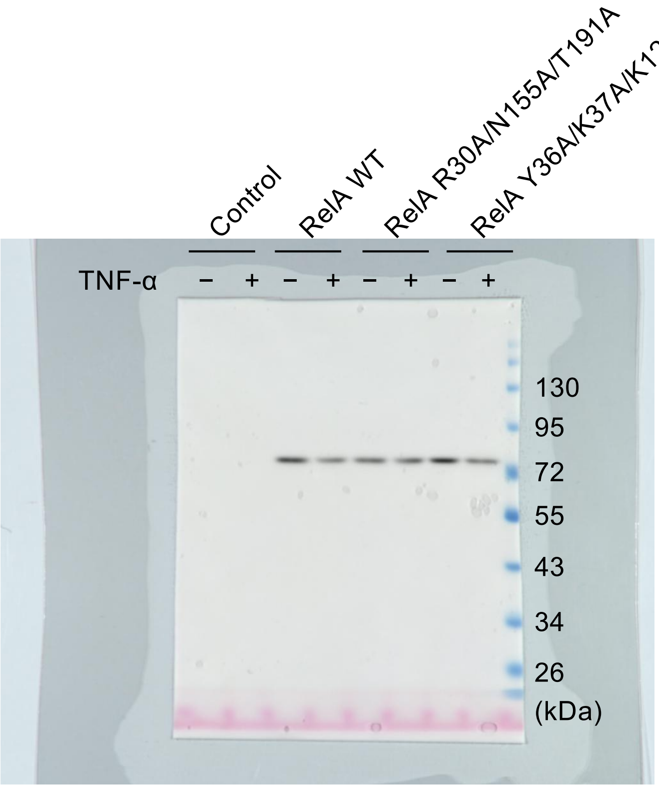

WB: RelA

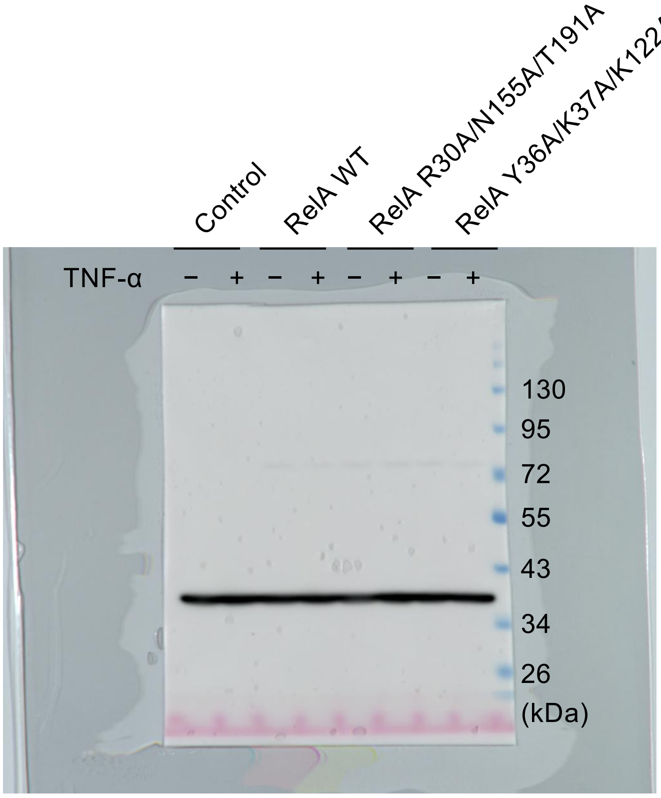

WB: GAPDH (reprobed)

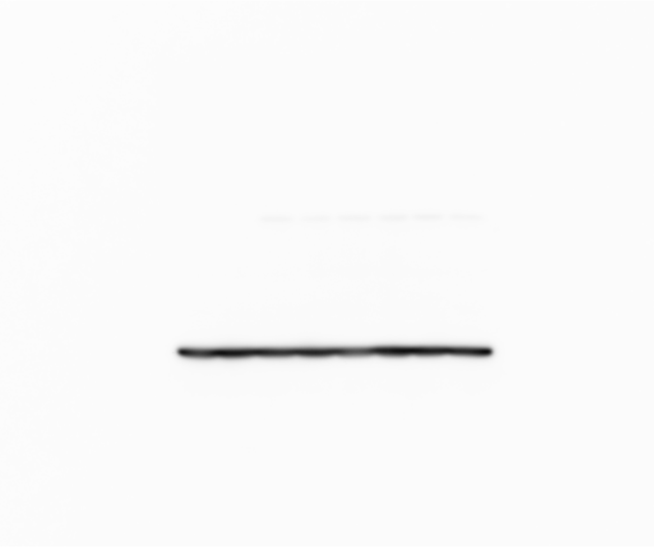

Figure S93: Original blots (3) in Figure 14D

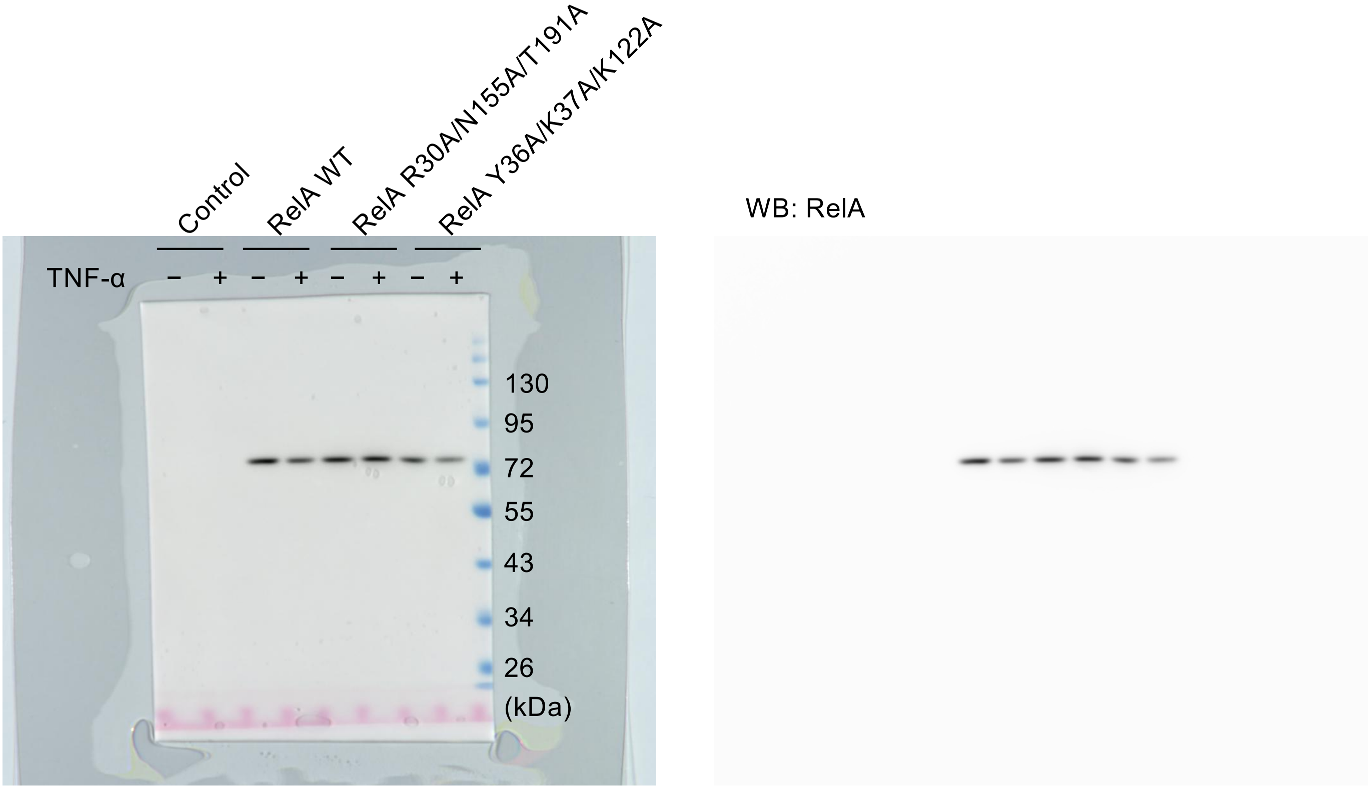

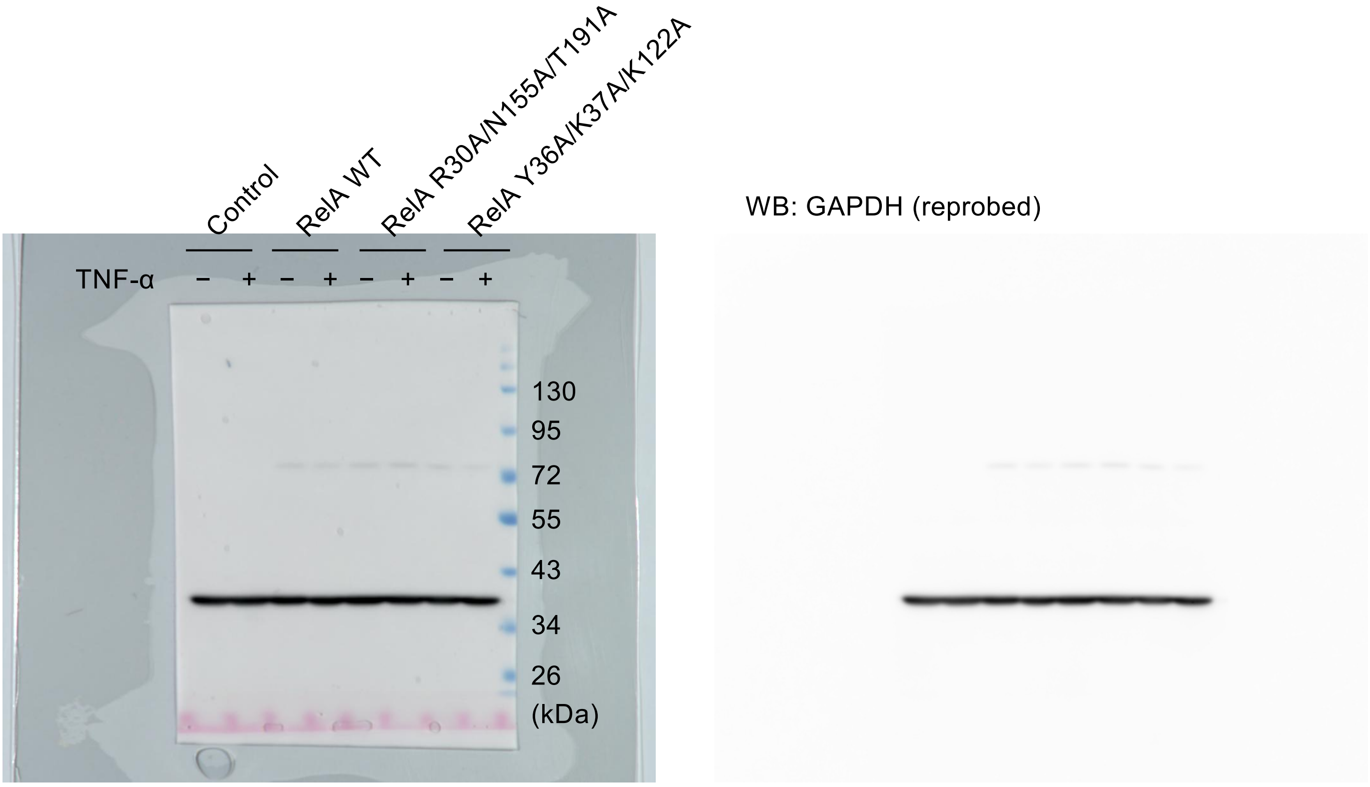

Figure S94: Original blots in Figure 14E (nucleus)

Cardamonin (μM)

0 0 25 25

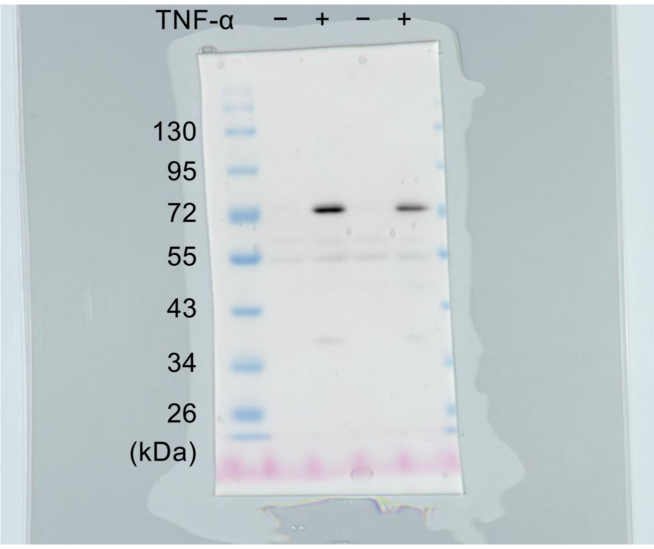

WB: RelA

Cardamonin (μM)

0 0 25 25

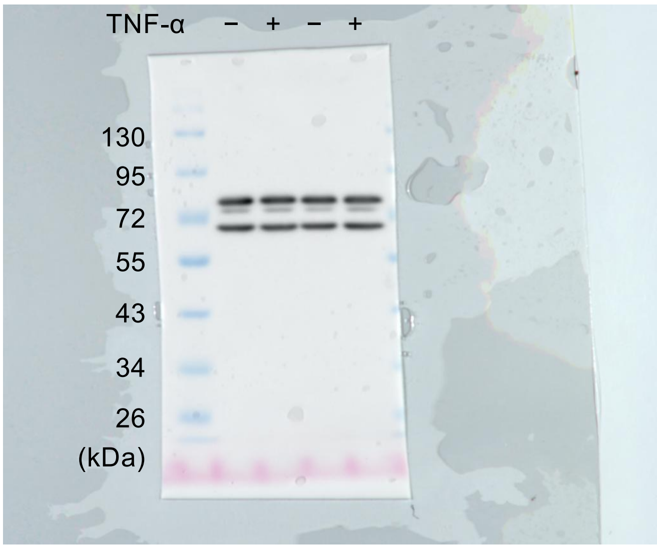

WB: LaminA/C (reprobed)

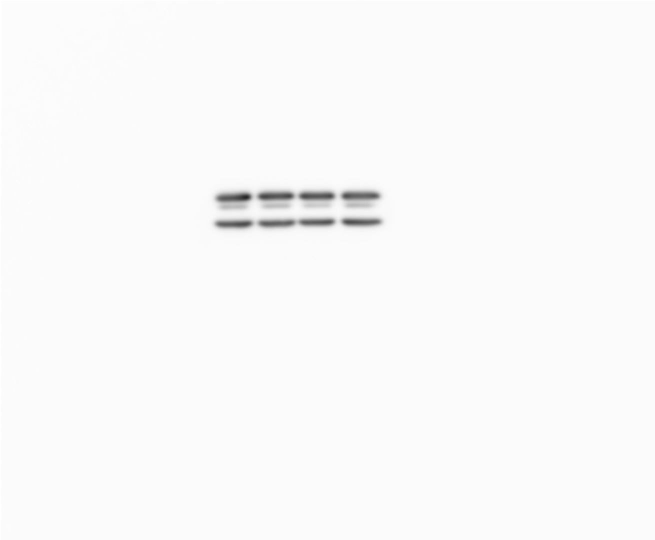

Figure S95: Original blots in Figure 14E (cytoplasm)

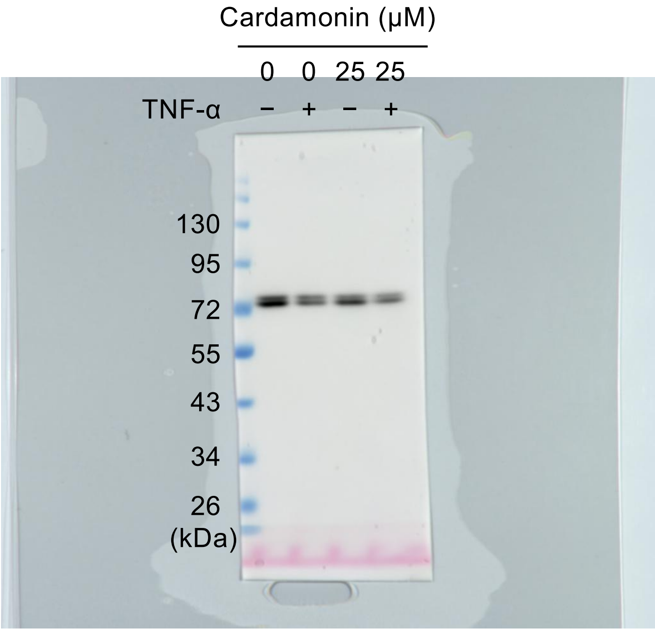

WB: RelA

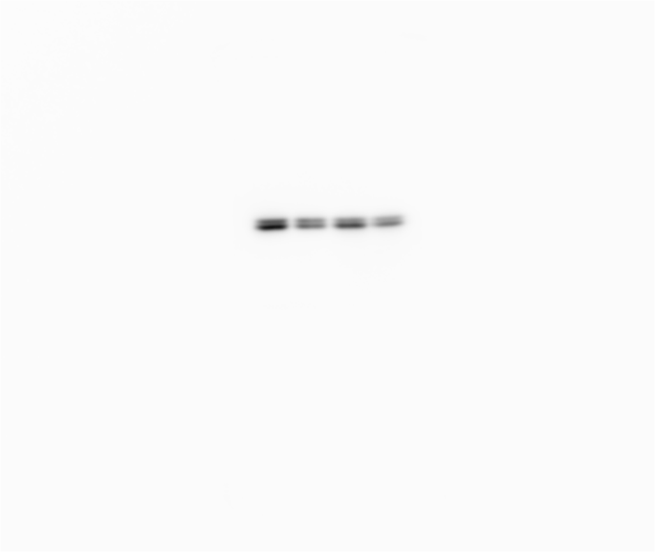

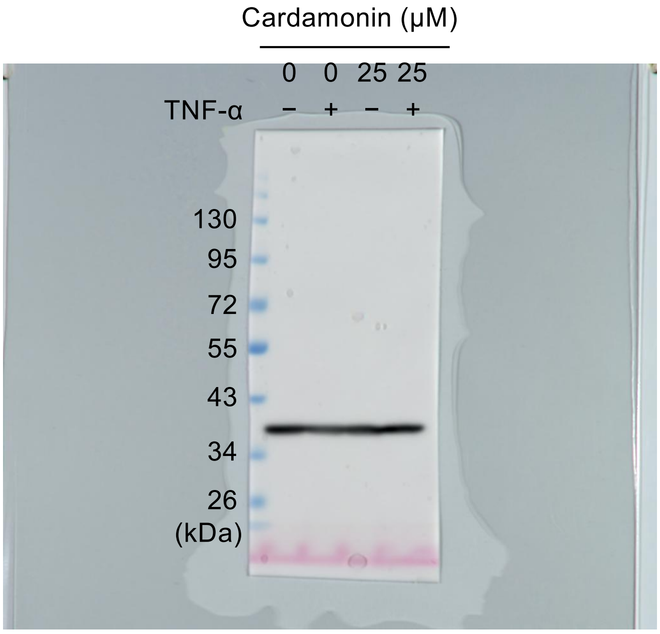

WB: GAPDH (reprobed)

Figure S96: Original blots (1) in Figure 14F

Cardamonin (μM)

0 0 25 25

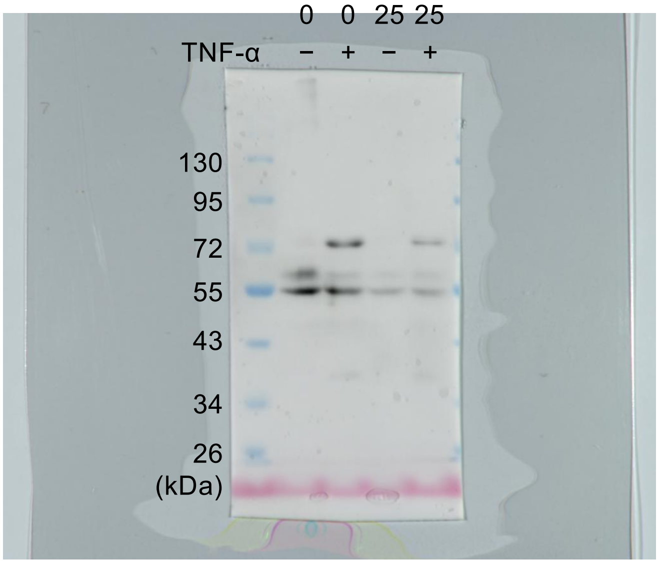

WB: RelA

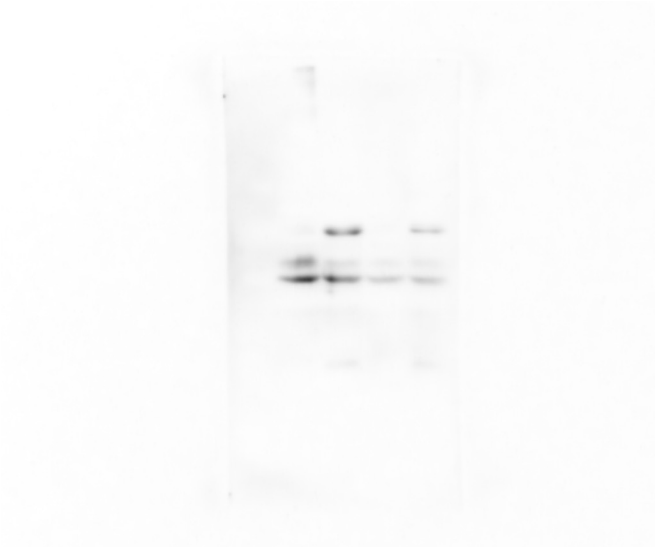

Cardamonin (μM)

0 0 25 25

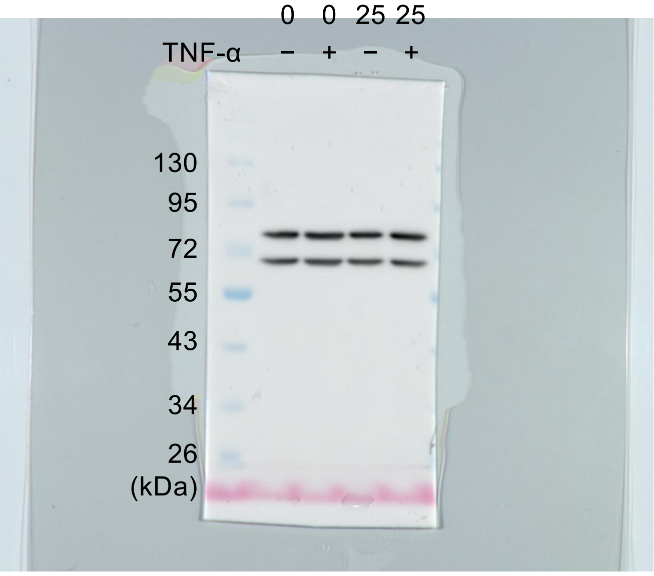

WB: LaminA/C (reprobed)

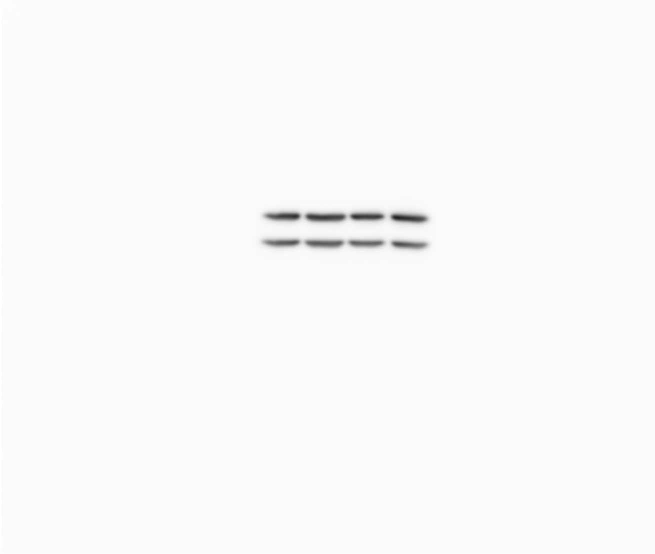

Figure S97: Original blots (2) in Figure 14F

Cardamonin (μM)

0 0 25 25

WB: RelA

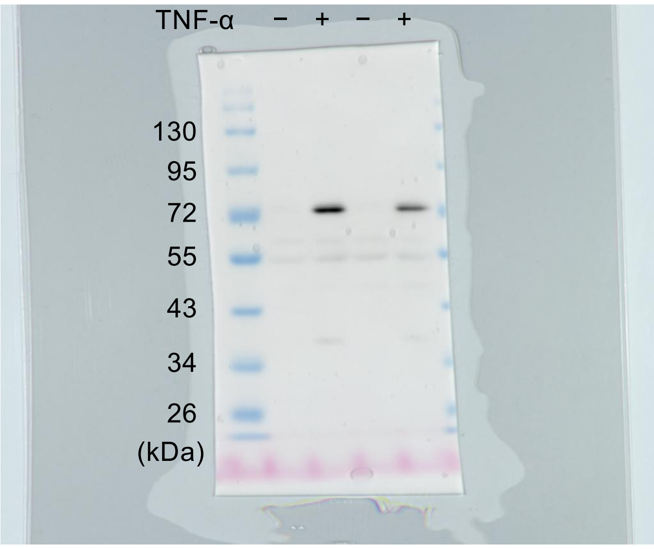

Cardamonin (μM)

0 0 25 25

WB: LaminA/C (reprobed)

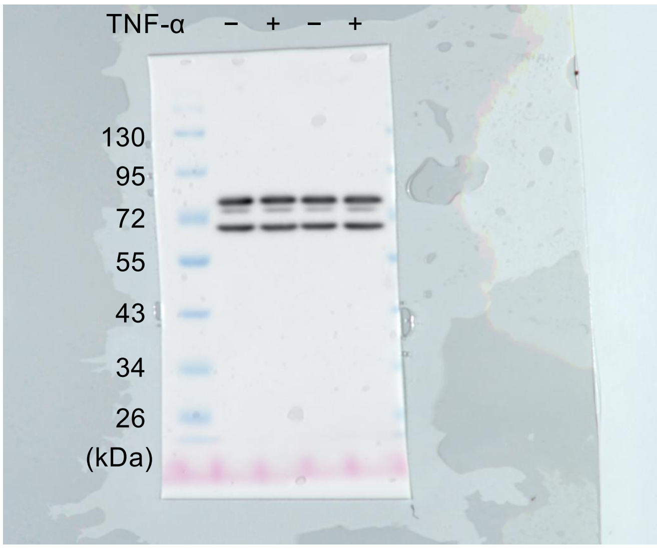

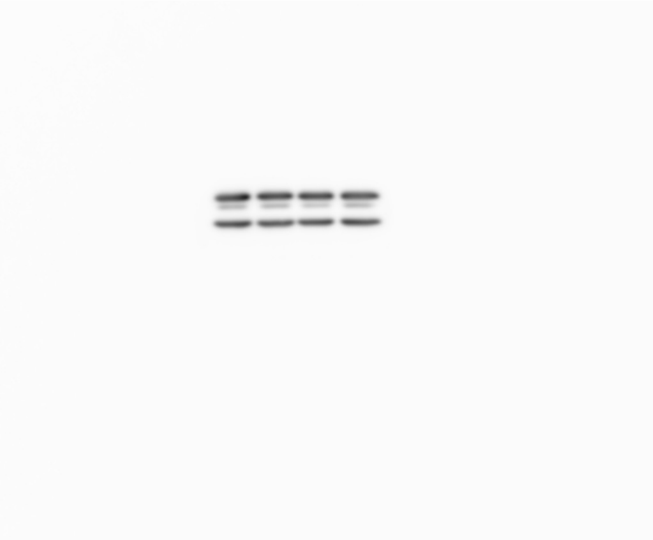

Figure S98: Original blots (3) in Figure 14F

Cardamonin (μM)

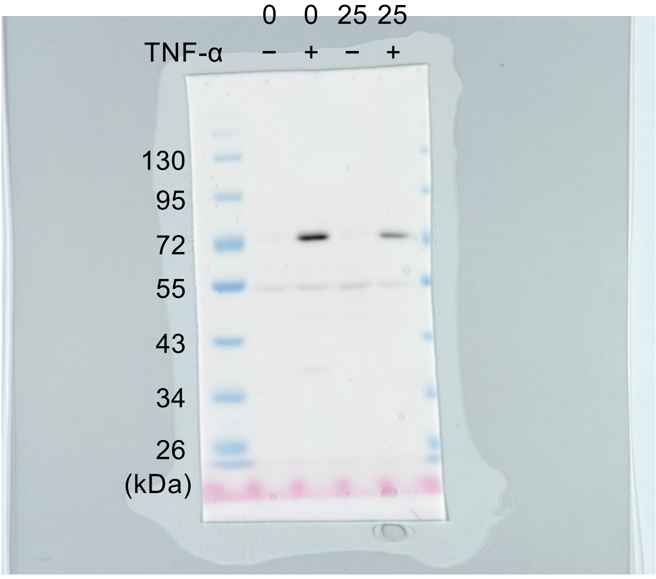

WB: RelA

Cardamonin (μM)

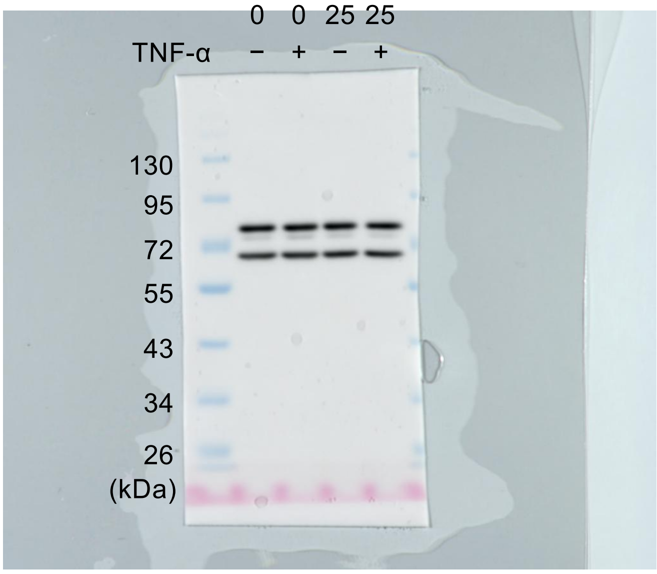

WB: LaminA/C (reprobed)

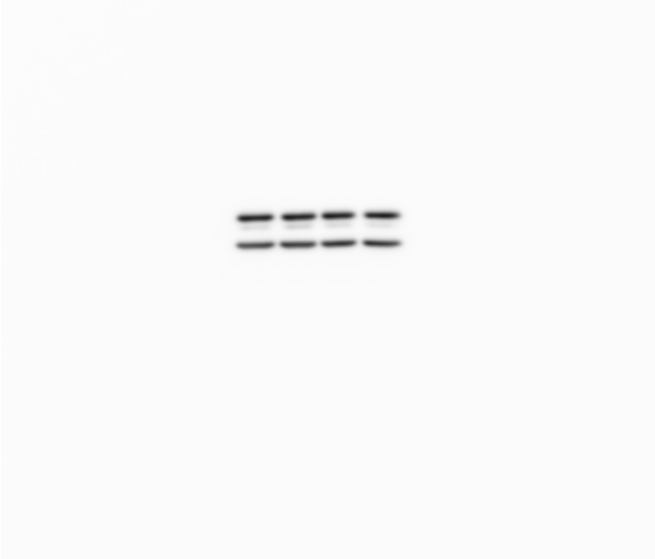

Figure S99: Original blots (1) in Figure 14G

Cardamonin (μM)

0 0 25 25

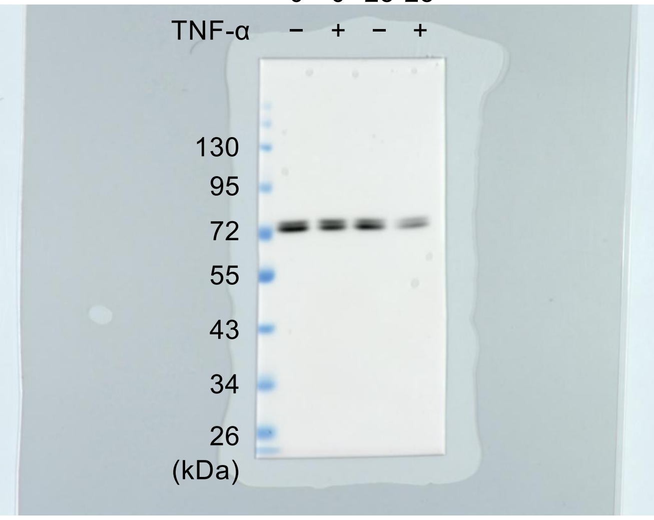

WB: RelA

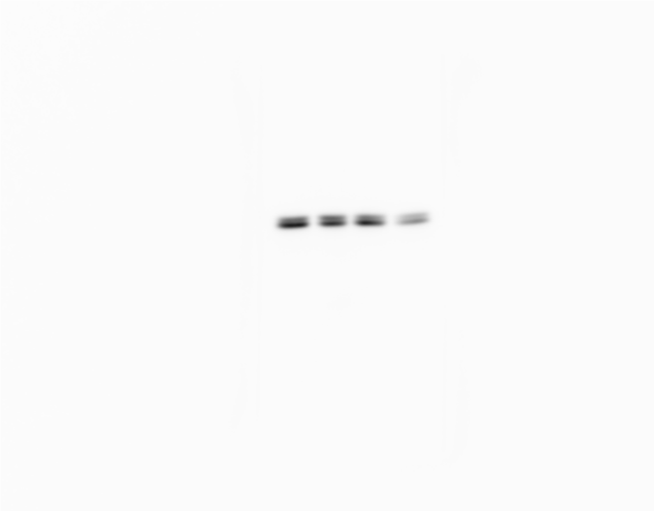

Cardamonin (μM)

0 0 25 25

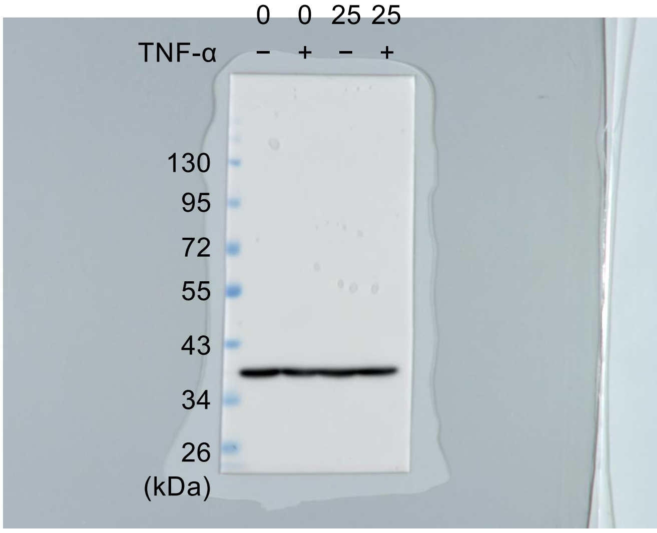

WB: GAPDH (reprobed)

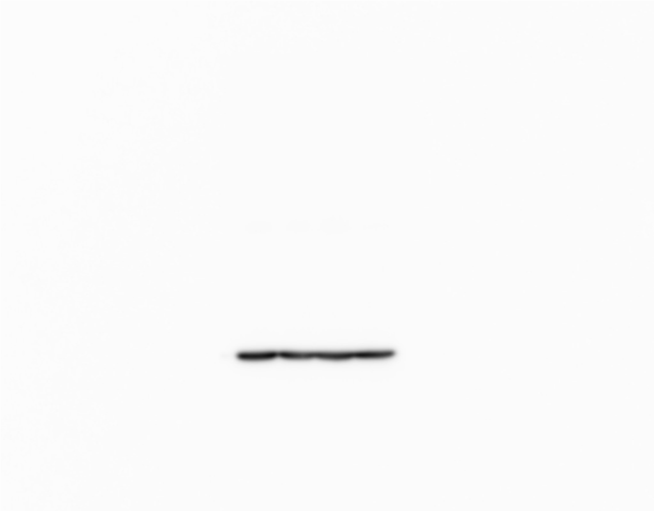

Figure S100: Original blots (2) in Figure 14G

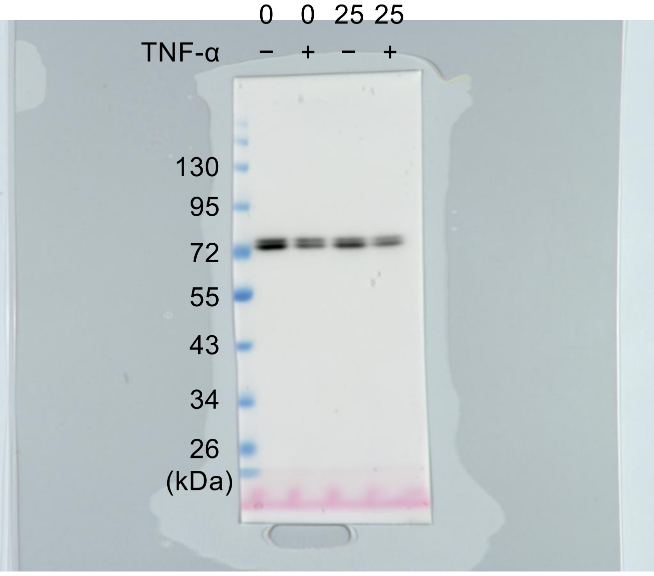

WB: RelA

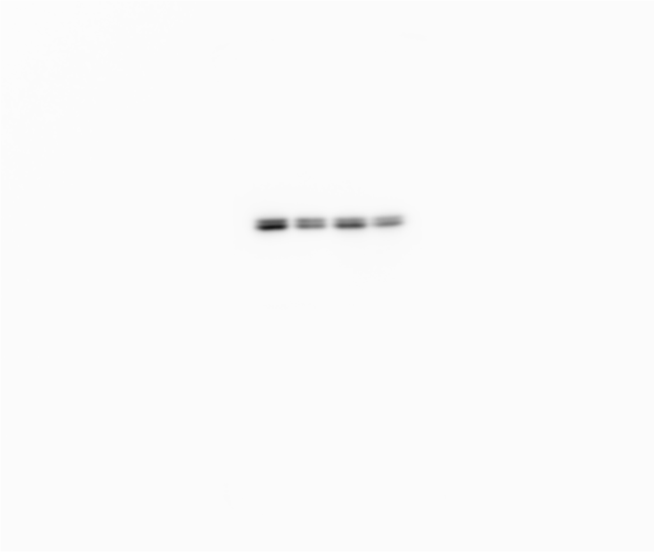

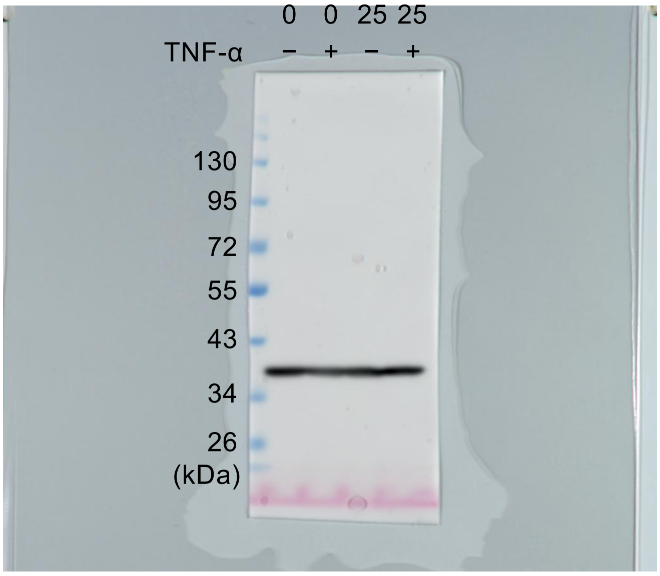

WB: GAPDH (reprobed)

Figure S101: Original blots (3) in Figure 14G

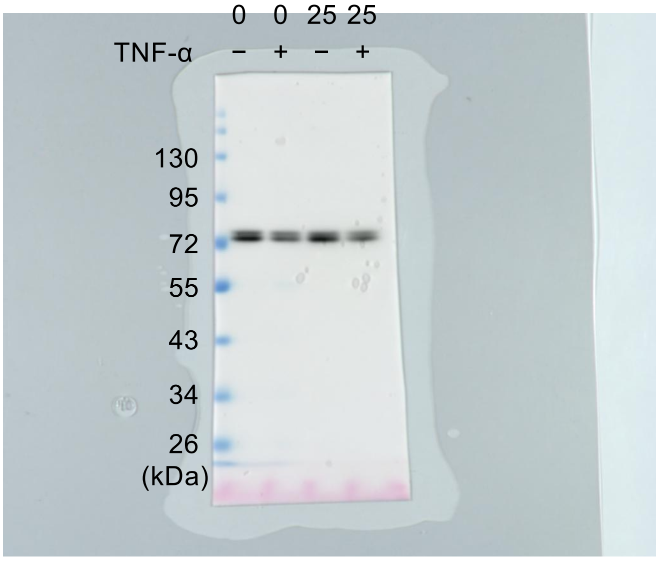

WB: RelA

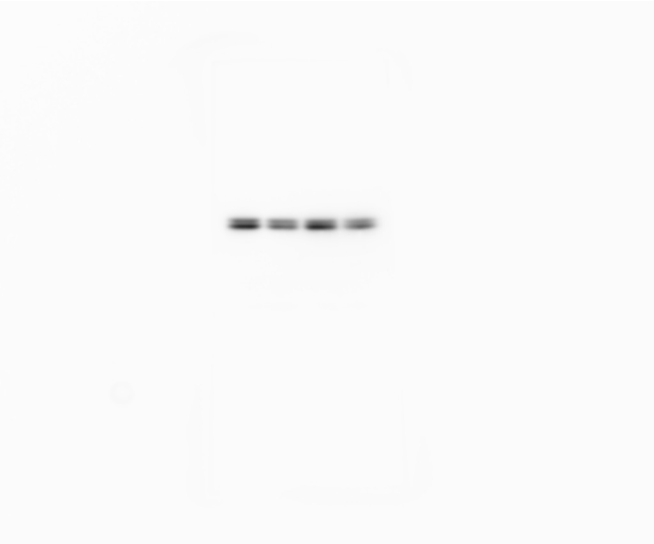

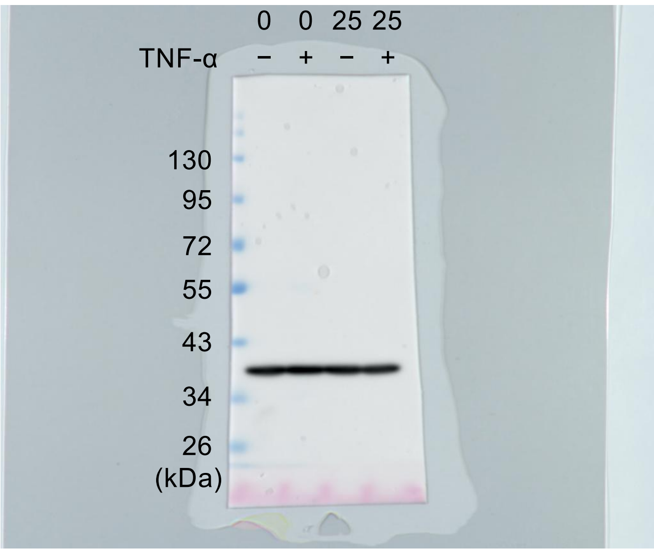

WB: GAPDH (reprobed)

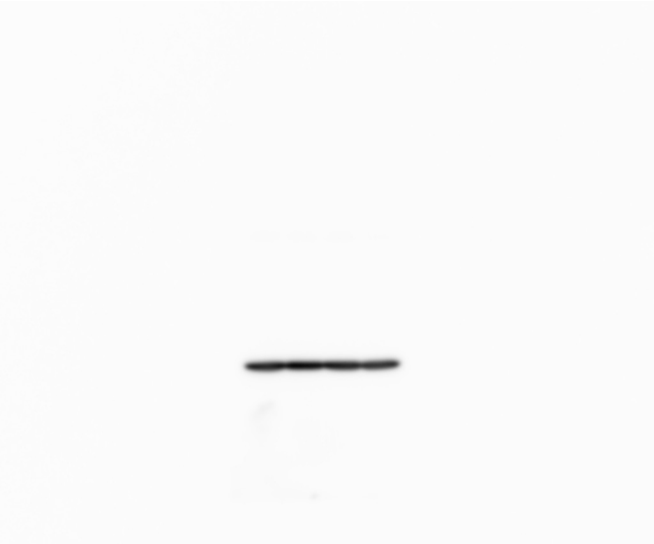

Figure S102: Original blots in Figure 14H (nucleus)

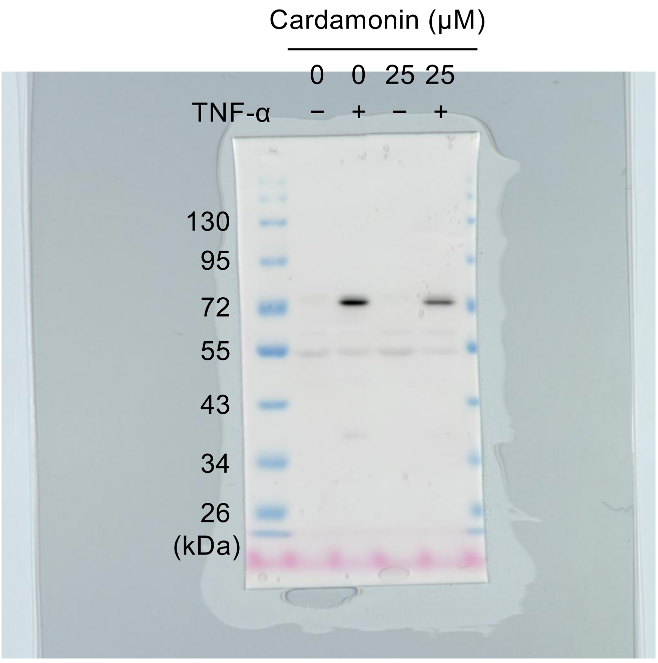

WB: RelA

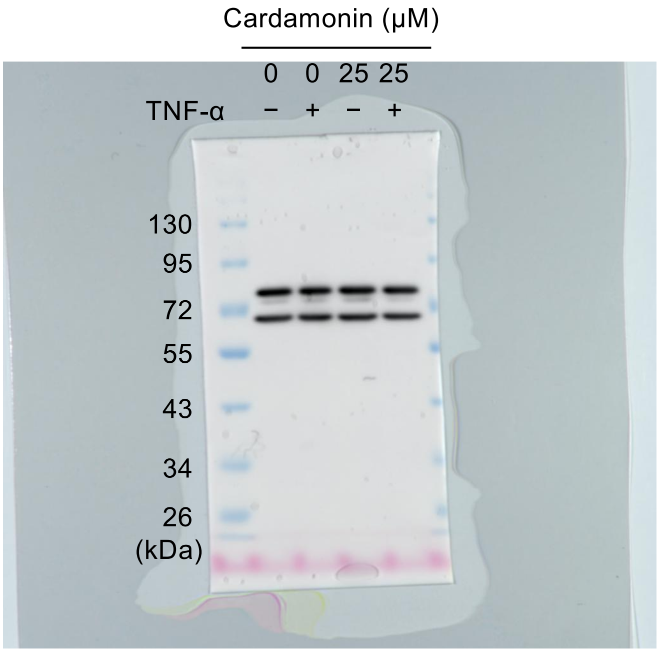

WB: LaminA/C (reprobed)

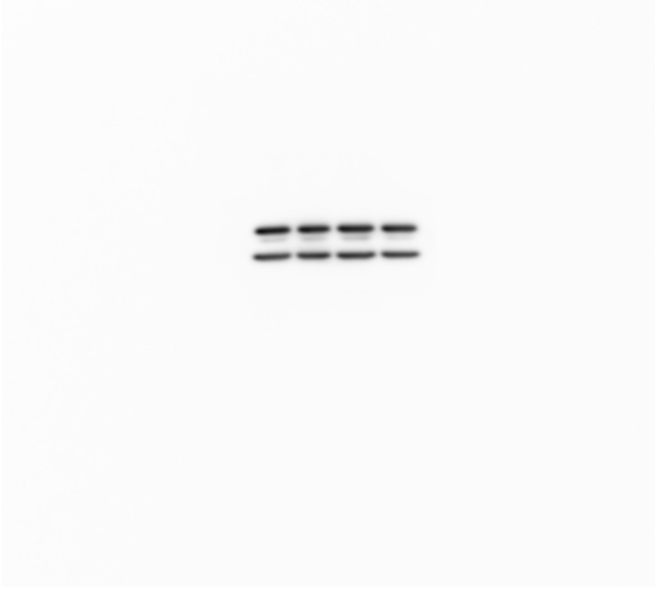

Figure S103: Original blots in Figure 14H (cytoplasm)

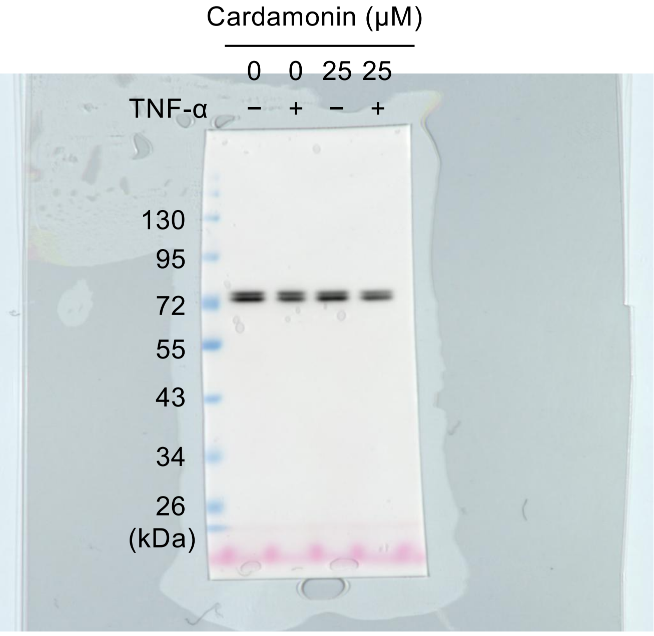

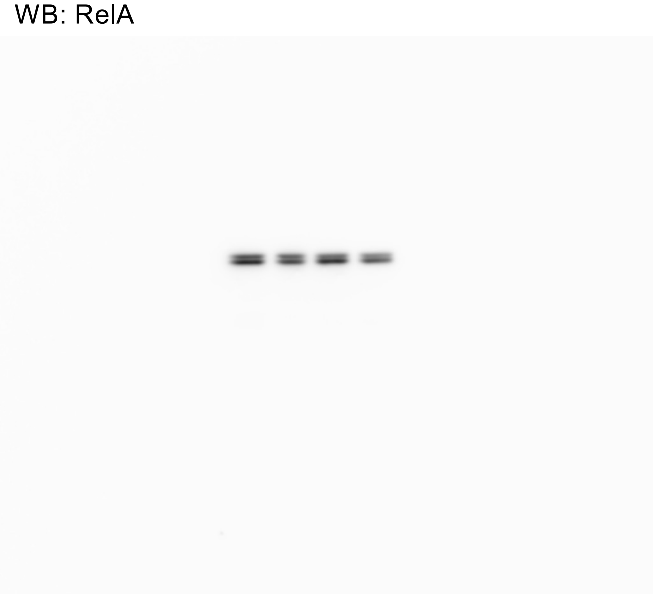

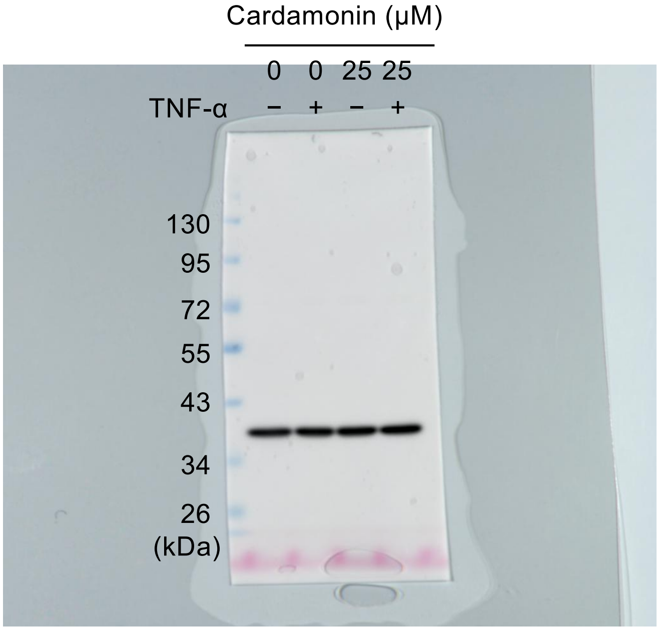

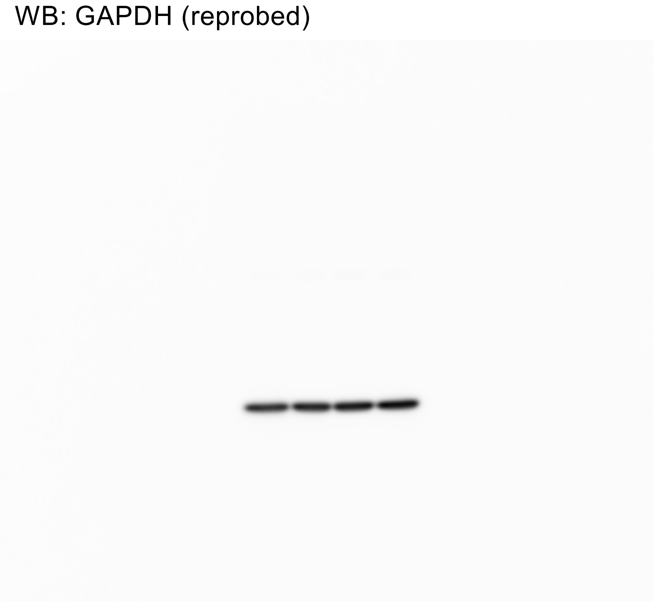

Figure S104: Original blots (1) in Figure 14I

Cardamonin (μM)

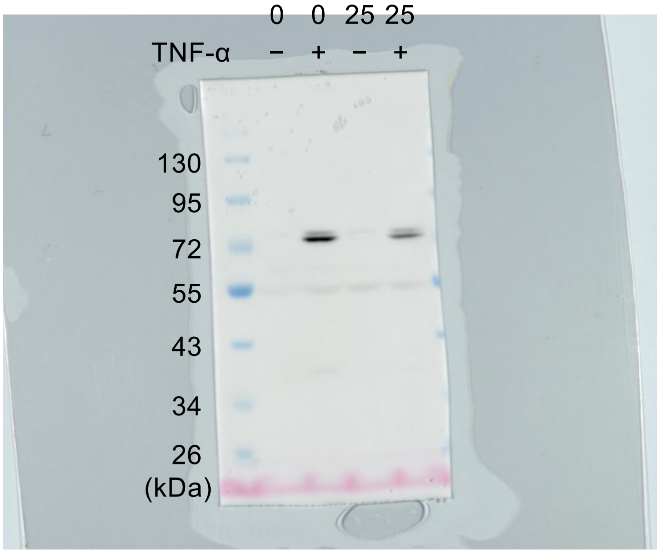

WB: RelA

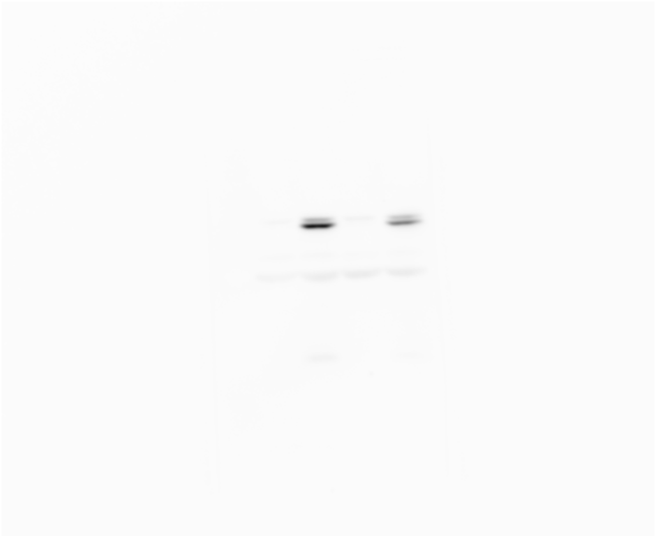

Cardamonin (μM)

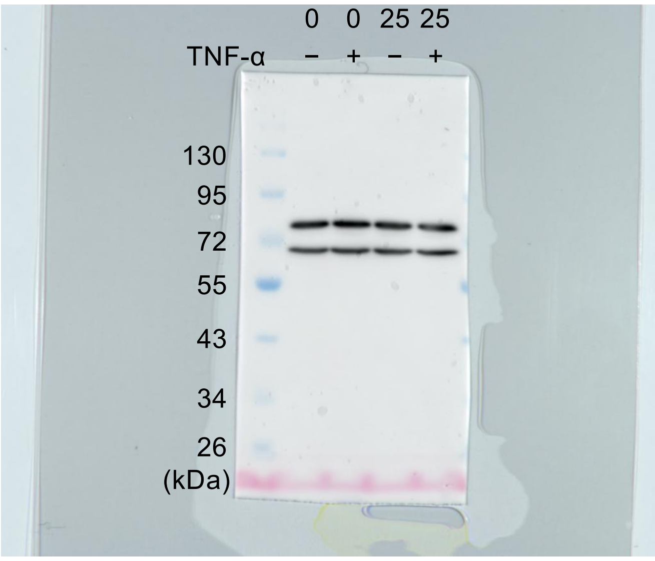

WB: LaminA/C (reprobed)

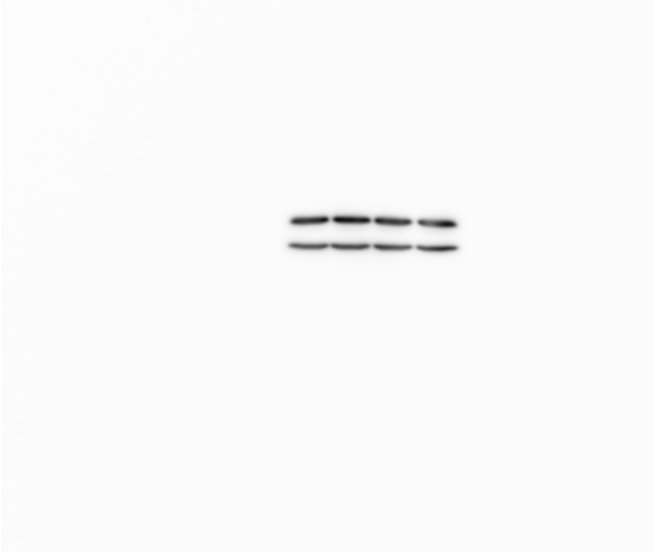

Figure S105: Original blots (2) in Figure 14I

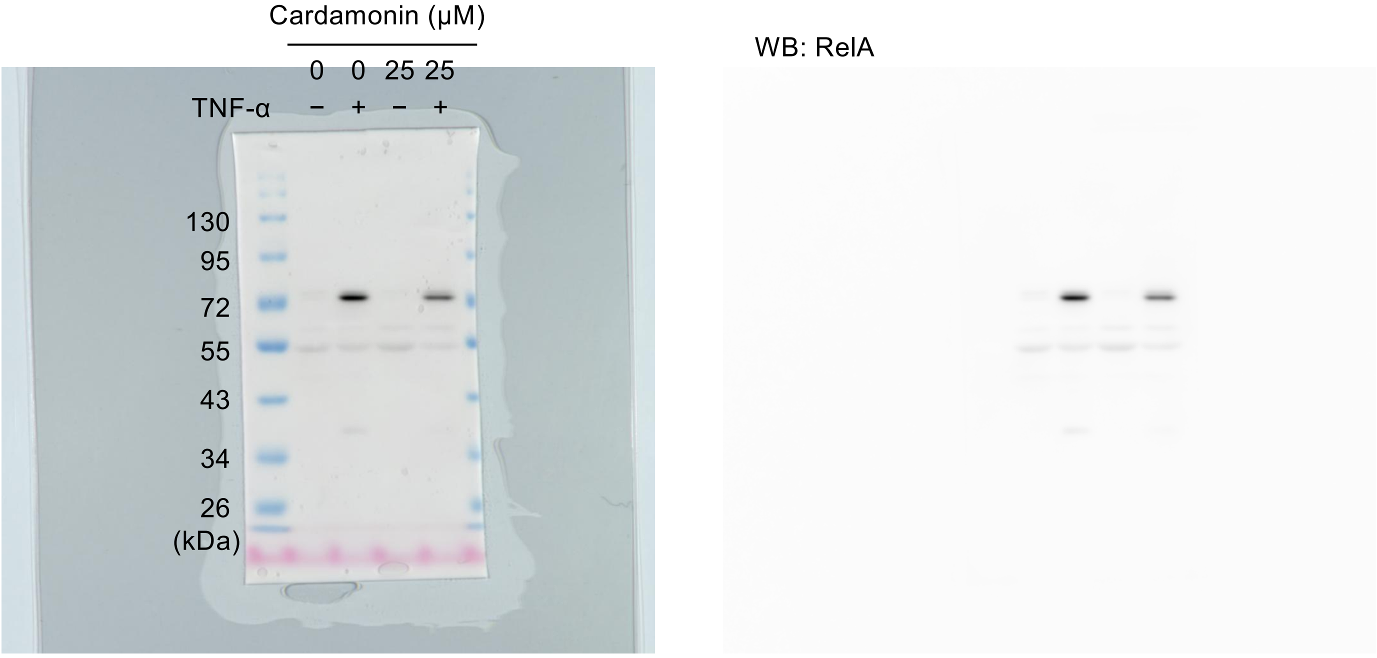

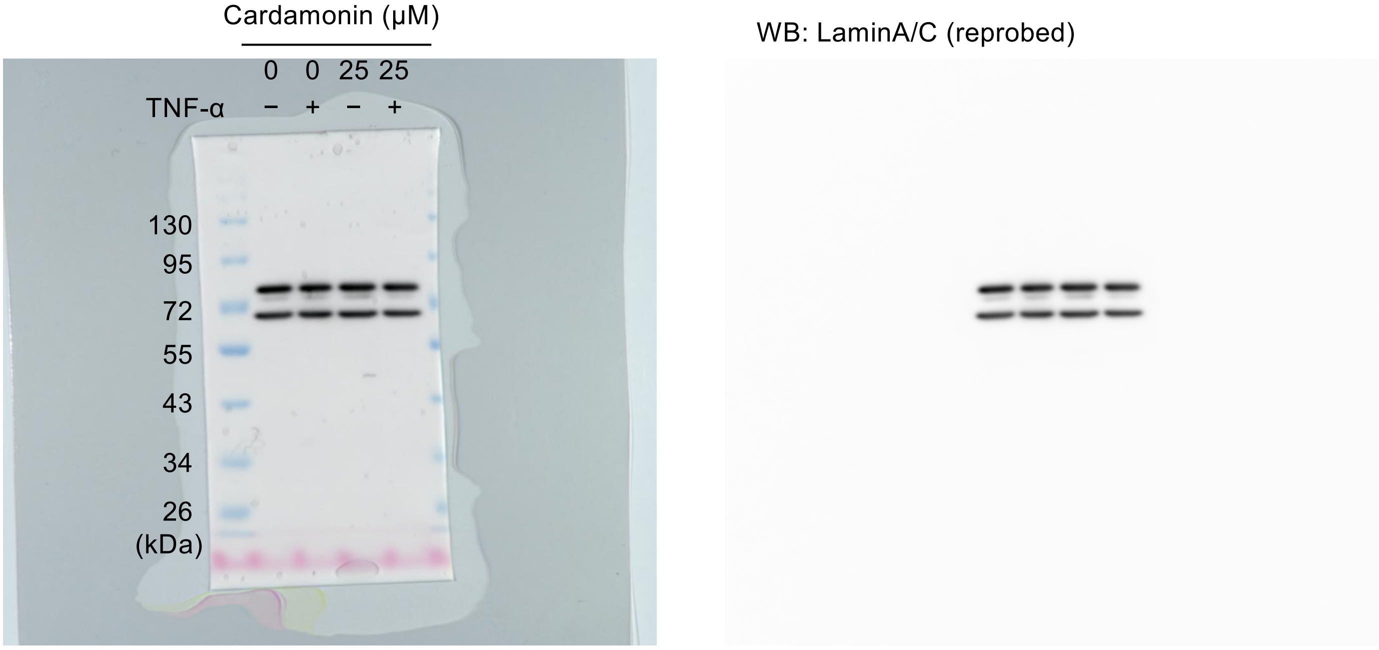

Figure S106: Original blots (3) in Figure 14I

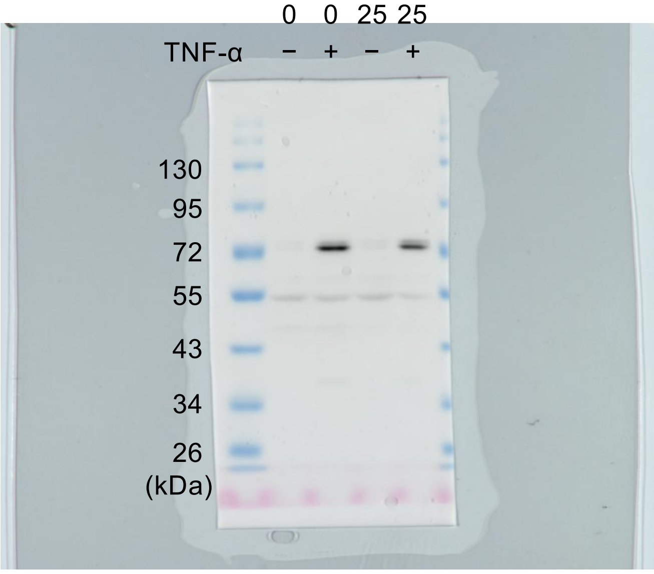

WB: RelA

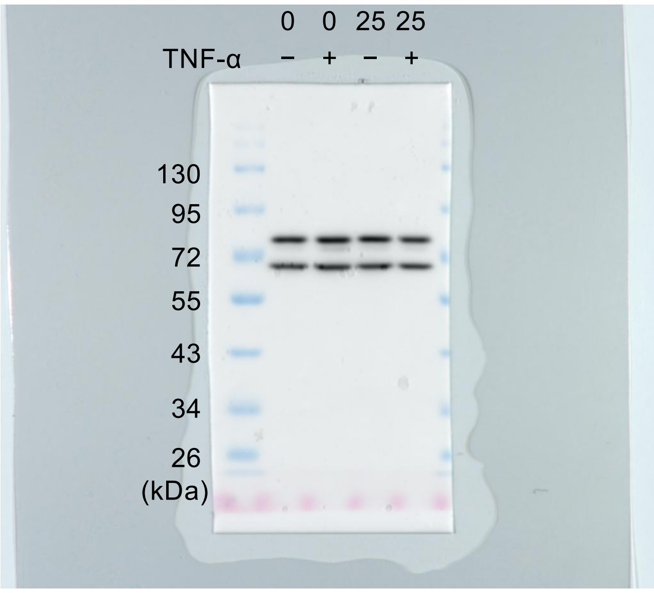

WB: LaminA/C (reprobed)

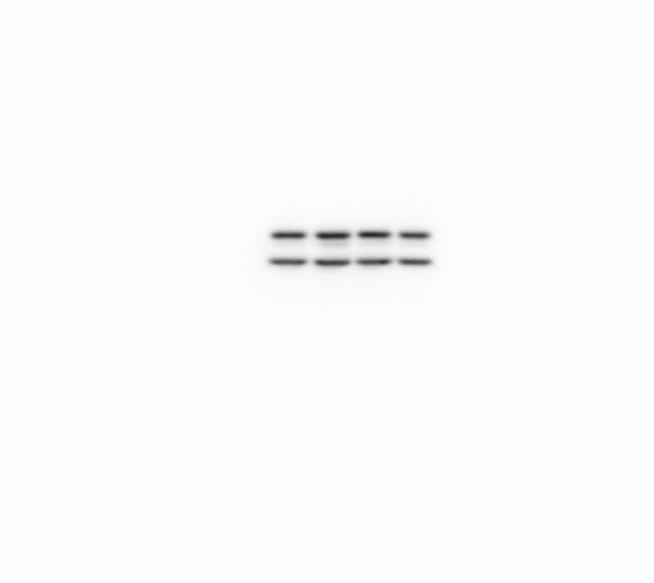

Figure S107: Original blots (1) in Figure 14J

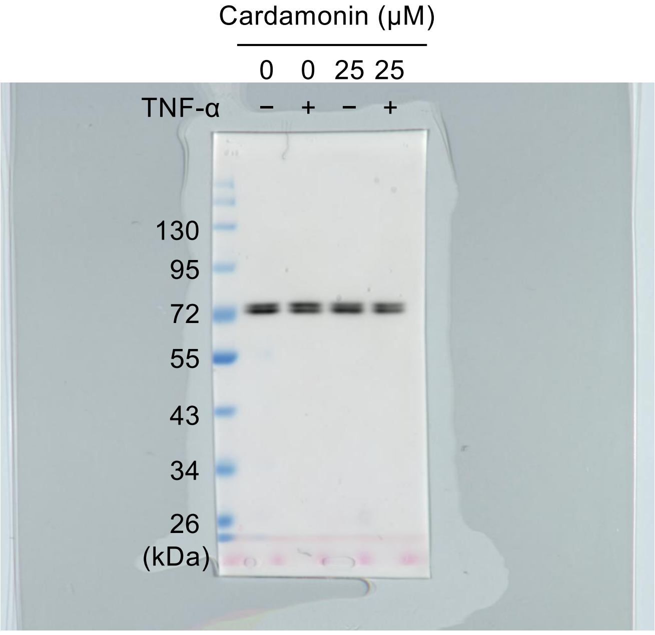

WB: RelA

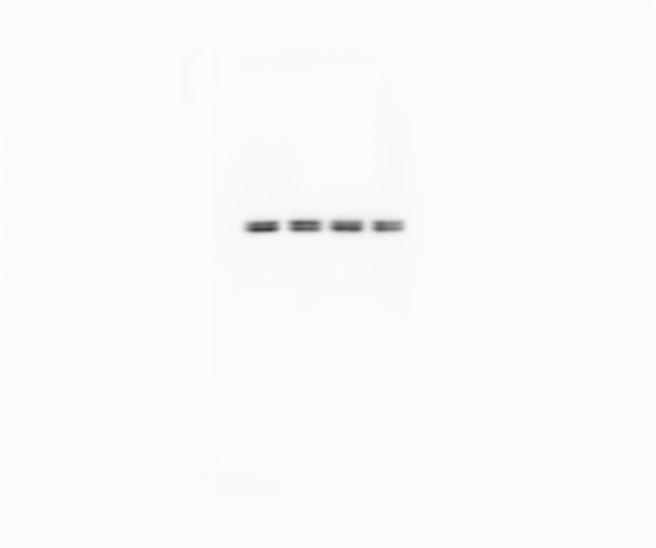

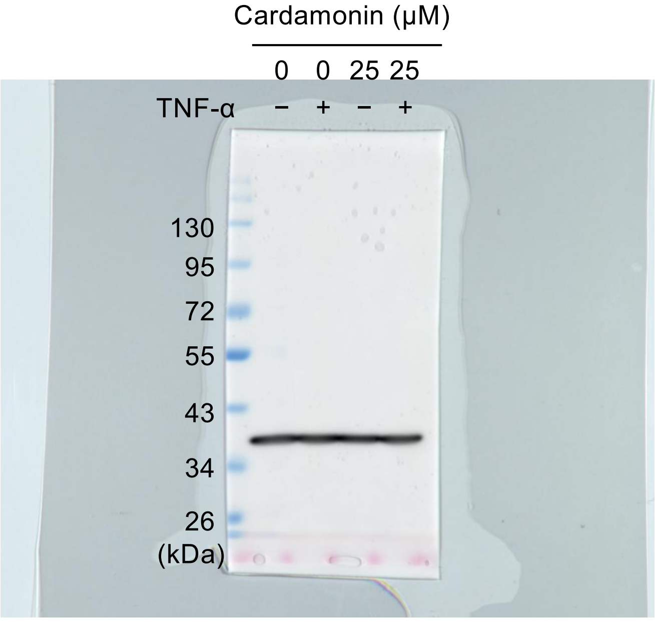

WB: GAPDH (reprobed)

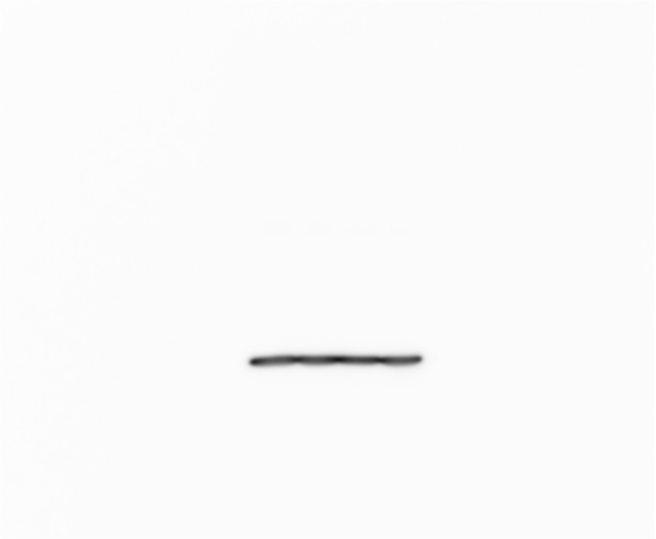

Figure S108: Original blots (2) in Figure 14J

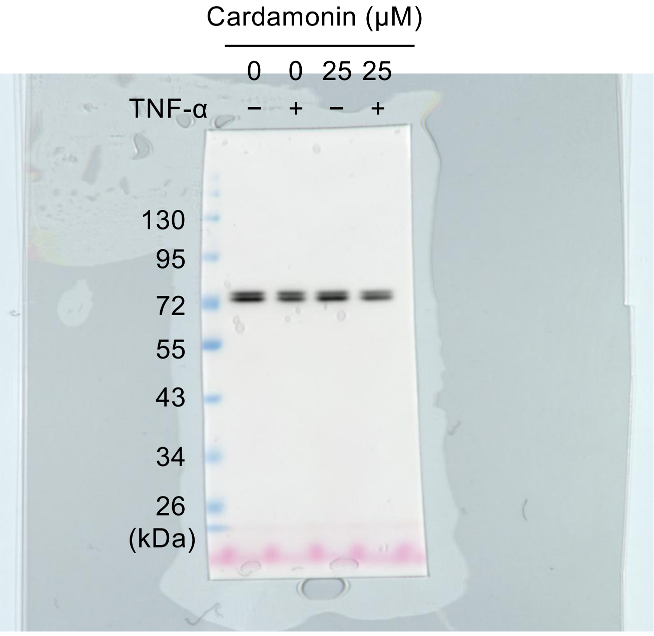

WB: RelA

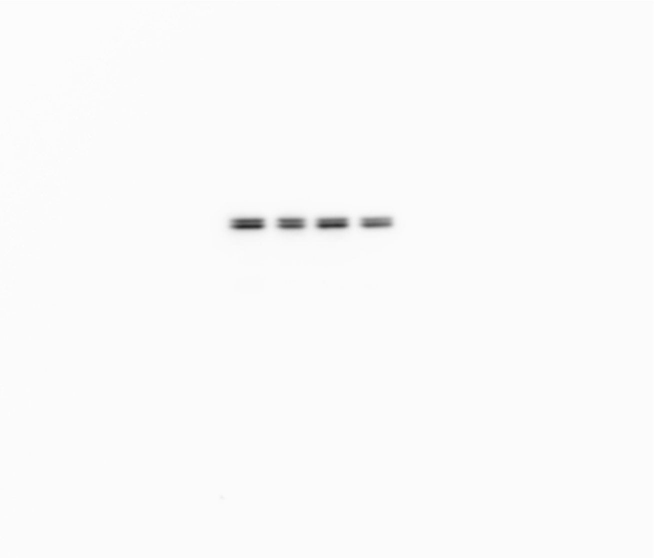

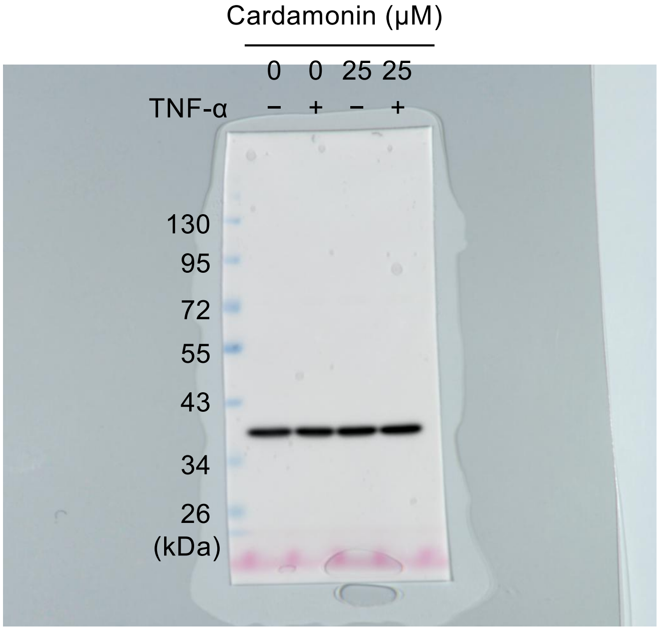

WB: GAPDH (reprobed)

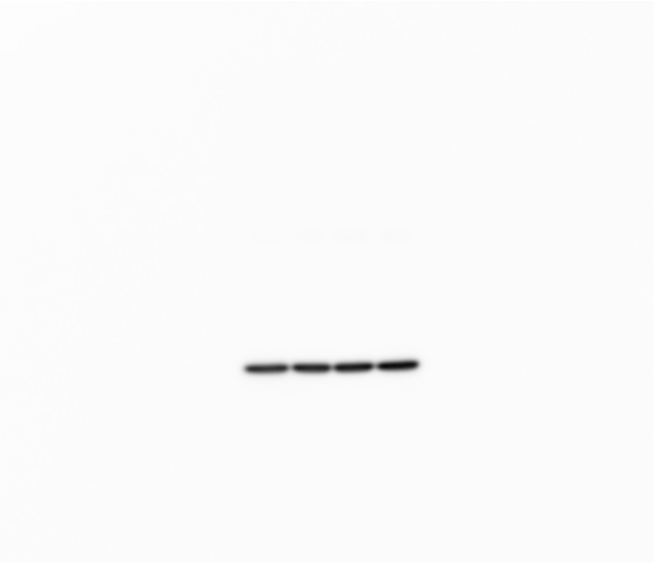

Figure S109: Original blots (3) in Figure 14J

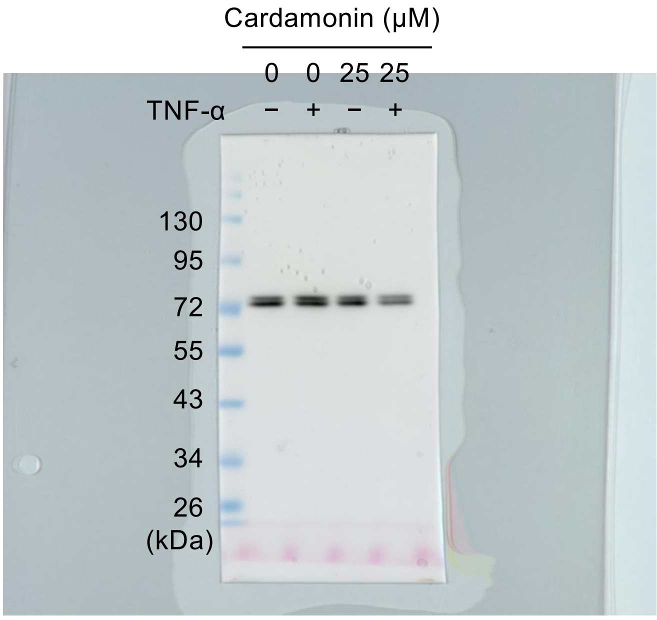

WB: RelA

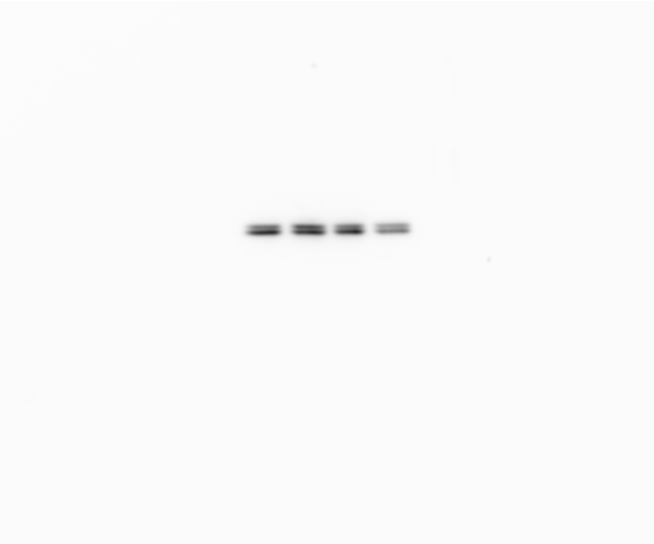

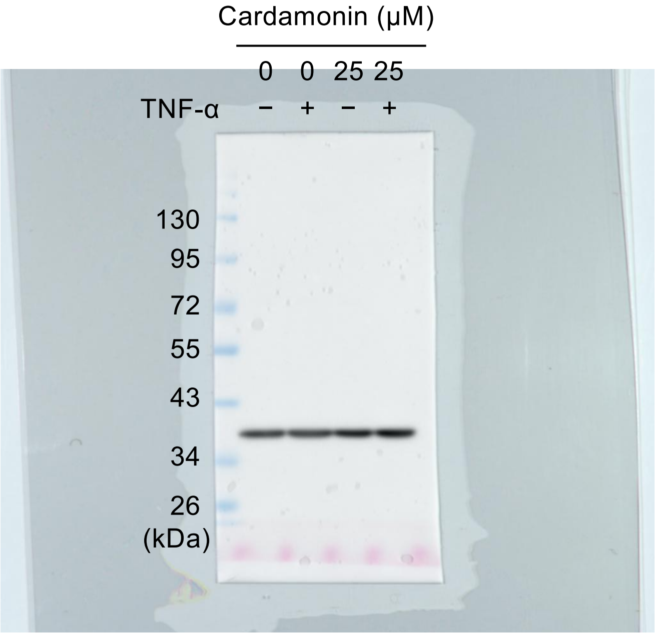

WB: GAPDH (reprobed)
